# Genomic landscape of the SARS-CoV-2 pandemic in Brazil suggests an external P.1 variant origin

**DOI:** 10.3389/fmicb.2022.1037455

**Published:** 2022-12-22

**Authors:** Camila P. Perico, Camilla R. De Pierri, Giuseppe Pasqualato Neto, Danrley R. Fernandes, Fabio O. Pedrosa, Emanuel M. de Souza, Roberto T. Raittz

**Affiliations:** ^1^Laboratory of Artificial Intelligence Applied to Bioinformatics, Professional and Technological Education Sector (SEPT), Federal University of Paraná, Curitiba, Brazil; ^2^Graduate Program in Bioinformatics, Professional and Technological Education Sector (SEPT), Federal University of Paraná, Curitiba, Brazil; ^3^Department of Biochemistry and Molecular Biology, Federal University of Paraná, Curitiba, Brazil

**Keywords:** genomics and proteomics, big data, SWeeP, machine learning, diversity, virus

## Abstract

Brazil was the epicenter of worldwide pandemics at the peak of its second wave. The genomic/proteomic perspective of the COVID-19 pandemic in Brazil could provide insights to understand the global pandemics behavior. In this study, we track SARS-CoV-2 molecular information in Brazil using real-time bioinformatics and data science strategies to provide a comparative and evolutive panorama of the lineages in the country. SWeeP vectors represented the Brazilian and worldwide genomic/proteomic data from Global Initiative on Sharing Avian Influenza Data (GISAID) between February 2020 and August 2021. Clusters were analyzed and compared with PANGO lineages. Hierarchical clustering provided phylogenetic and evolutionary analyses of the lineages, and we tracked the P.1 (Gamma) variant origin. The genomic diversity based on Chao's estimation allowed us to compare richness and coverage among Brazilian states and other representative countries. We found that epidemics in Brazil occurred in two moments with different genetic profiles. The P.1 lineages emerged in the second wave, which was more aggressive. We could not trace the origin of P.1 from the variants present in Brazil. Instead, we found evidence pointing to its external source and a possible recombinant event that may relate P.1 to a B.1.1.28 variant subset. We discussed the potential application of the pipeline for emerging variants detection and the PANGO terminology stability over time. The diversity analysis showed that the low coverage and unbalanced sequencing among states in Brazil could have allowed the silent entry and dissemination of P.1 and other dangerous variants. This study may help to understand the development and consequences of variants of concern (VOC) entry.

## 1. Introduction

The current pandemic of Severe Acute Respiratory Syndrome Coronavirus 2 (SARS-CoV-2), which causes the disease known as Corona Virus Disease 2019 (COVID-19) (Zhou et al., 2020), was first reported in Brazil in February 2020. Brazil was the pandemic's epicenter during the peak of COVID-19 second wave, around April 2021.

New variants continually emerge, and many of them are considered variants of concern (VOC), such as the British B.1.1.7 (Alpha), the South African B.1.351 (Beta), the Indian B.1.617.2 (Delta), and the P.1 (Gamma), which was first identified in Brazil in November 2020 (Faria et al., 2021). In addition, variants acquire mutations that make them more adapted, transmissible and challenging to detect by the immune system (Berger and Schaffitzel, 2020; Korber et al., 2020; Yurkovetskiy et al., 2020). Therefore, virus monitoring is essential to diagnose, improve treatment, characterize strains and sub-strains, and thus understand their dynamics and dispersion (Cella et al., 2021). It is also of utmost importance in health policy decisions. International and domestic travel without quarantine is a significant vehicle for spreading potentially dangerous variants, as occurred at the beginning of the pandemic in 2020 before air travel restrictions (Candido et al., 2020). Proper quarantine use positively impacted case reduction and neglected quarantine caused exponential growth in infected curves (Li et al., 2021).

Franceschi et al. (2021) presented the Brazilian panorama until February 2021, when it completed a year of pandemic in Brazil. The authors analyzed mutations, phylogeny, and phylogeography of the virus in the Brazilian context by exploiting conventional bioinformatics tools, with a genomic focus, analyzing 2,732 sequences. The viral sequence data is immense, reaching more than 3.4 million genomes sequenced worldwide by September 2021 in the Global Initiative on Sharing Avian Influenza Data (GISAID) Database (Shu and McCauley, 2017), where the numbers are increasing constantly.

Current methods based on sequence alignment cannot process large volumes of data due to the exponential growth of the computational cost. Conventional bioinformatics is not enough to thoroughly analyze large volumes of data. However, data mining and machine learning methods can be decisive in extensive data analysis, providing reliable and fast results. These methods are already widely in use, with several applications in different areas, including the taxonomic classification of coronavirus genomes (Alimadadi et al., 2020; Randhawa et al., 2020).

Previous studies showed that alignment-free methods, particularly vector representation of biological sequences, are fast, scalable, and effective in analyzing SARS-CoV-2 sequences and efficient in associating with machine learning methods (De Pierri et al., 2020; Randhawa et al., 2020; Rui et al., 2020; Raittz et al., 2021). Vector representation of biological sequences is an emerging method that facilitates the implementation of data science techniques and has already proven effective in applications in bioinformatics (Asgari and Mofrad, 2015; Zhang et al., 2017; Leimeister et al., 2019; De Pierri et al., 2020; Raittz et al., 2021).

This study attempted to understand how the emergence and extinction of SARS-CoV-2 lineages occur and verify if the variants in the databases are correctly defined. As suggested in the correlated studies, the terminology PANGO (or PANGOLIN) was adopted (González-Candelas et al., 2021; To et al., 2021). We constructed a pipeline in R language based on the application of vector representation, data mining, and machine learning methods to obtain the current panorama of the pandemic in real time and to understand the evolution of the virus in Brazil. To understand the virus evolution process, we tested the hypothesis of the external origin of the P.1 variant and the possibility of whether or not a recombination event was involved in its origin. Furthermore, to facilitate monitoring and adequate decision-make action, we investigated whether our pipeline is suitable for the early detection of the emergence of new strains.

## 2. Materials and methods

Supplementary Figure S1 presents the pipeline constructed in R language that is available at https://github.com/CamilaPPerico/SARS-CoV-2_Brazil_Landscape/, as well as the other results of this research. Using the available pipeline, the main results of this study can be reproduced. The sequences used in this paper, except the Wuhan reference sequence (Wu et al., 2020), were downloaded from the GISAID database and represented into vectors. Euclidean is the adopted metric for distance in this study. We ran the analysis on a Xeon server with 251Gb of RAM and 40 threads.

### 2.1. Obtaining and pre-processing of SARS-CoV-2 sequences

We downloaded the proteomes of SARS-CoV-2 and the sequences corresponding to the Brazilian genomes from GISAID (https://gisaid.org/) (Elbe and Buckland-Merrett, 2017). The PANGO nomenclature^1^ (Rambaut et al., 2020) was adopted. The sequences were obtained from GISAID in three different moments, with its corresponding PANGO designation: a) initial analysis (GISAID release 409, PANGO v.2.3.8 2021-04-20); b) principal analysis (release 609, PANGO v3.0.5 2021-06-04); and c) final update (release 829, PANGO v.3.1.11 2021-08-09). The Wuhan reference sequence (NC_045512.2) is from the NCBI^2^ database.

The addressed sequences from Brazil, Italy, India, Germany, and England correspond to the period from the pandemic onset to the end of May 2021, while other worldwide considered sequences were from 2020 only. All incomplete proteomes and the sequences with misreading were not considered. However, when only one protein was absent, it was accepted (Pereira, 2021). Table 1 shows the number of samples before and after filtering by quality (quality sequences considered were those with proteome complete and without misreadings).

**Table 1:** Relationship between the number of sequences analyzed per country and the computational time for the vectorization.

**Country**	**Total**	**Quality**	**Non-redundant**	**rSWeeP (min)**
Brazil	13,395	8,720	6,146	12.2
India	18,558	7,154	5,493	17.4
Italy	33,014	12,784	6,617	18.4
Germany	126,794	51,880	22,048	74.1
England	353,330	199,110	62,643	274.3
World (release 409)	1,000,558	493,080	312,224	22 h

Total, total number of samples present in GISAID in release 609 (except World-2020 from release 409); Quality, number of filtered vectors used for analysis; Non-redundant, number of unique vectors; rSWeeP, computational time in minutes to project the filtered sequences.

### 2.2. Mutations

We searched for Brazilian sequence mutations using the web platform Nextstrain^3^ from FASTA nucleotide files (Hadfield et al., 2018). Mutations statistics by cluster and by lineage were performed, considering only the Brazilian context. The characteristic mutations for each group were considered when present in more than 75% of the respective cluster or lineage samples.

### 2.3. Sequences vectorial representation

Protein sequences were concatenated (with border delimiters) into proteomes which were represented in vectors using the SWeeP tool (Spaced Words Projection) (De Pierri et al., 2020). The R version of the SWeeP tool, used for the proteome vectorization, is available in the Bioconductor Platform^4^ for R version 3.12 (Fernandes et al., 2020). Finally, we made the vector projection of the Brazilian genomes (coded in DNA) in the SWeeP tool in Matlab® (De Pierri et al., 2020) with its default parameters.

A total of 1,000,588 (1M) of SARS-CoV-2 proteomes from around the world were vectorized, comprising 9.97 billion amino acids, including the reference sequence of Wuhan and the spike protein of Brazilian samples separately integrated into the comparative study. The proteomes of Brazil, Germany, India, Italy, England, and World-2020 were vectorized and considered as independent sets. The same orthonormal base, with the SWeeP default parameters (length 600 and mask [1 1 0 1 1]), was employed to project all sequences into compacted vectors.

### 2.4. Cluster analysis and visualization

Brazilian proteomes were clustered using the ConsensusClusterPlus package version 1.54.0 from Bioconductor (Wilkerson and Hayes, 2010) and the K-medoids method (partitioning around medoids, PAM), in procedures with 1,000 replicates for each cycle, testing 2–given by the Equation (20) as the number of clusters. For the spike proteins, 2–10 sets were tested. As the selection criterion for the best number of clusters in both cases (proteome and protein spike), the best convergence in the consensus cumulative distribution function (CDF) associated with the smallest number of clusters was considered. We visualized and compared the clustering results using two approaches of dimensionality reduction: principal component analysis (PCA) and the t-distributed stochastic neighbor embedding (t-SNE) (Van der Maaten and Hinton, 2008). The t-SNE diagrams were constructed in the Rtsne package^5^, with its default parameters.

Information on the number of COVID-19 cases in Brazil was available at the official website https://covid.saude.gov.br/. In addition, the mapping of temporal and spatial evolutions was carried out based on information obtained from the metadata provided by the GISAID platform.

### 2.5. Diversity analysis

Coverage and richness of viral subvariants (unique and non-redundant sequences) were estimated *via* the Chao 1 richness estimator, given by the Equation (1) (Chao, 1984; Colwell and Coddington, 1994).


(1) $$\begin{array}{l} S_{1}=S_{obs}+\frac{F_{1}^{2}}{2F_{2}} \end{array}$$

In Equation (1), *S*_*obs*_ is the number of distinct vectorized proteomes observed, *F*_1_ is the number of singletons (single-occurring vectors), and *F*_2_ is the number of doubletons (two-occurring vectors). Thus, the coverage is given by


(2) $$\begin{array}{l} C_{ov}=\frac{S_{obs}}{S_{1}} \end{array}$$

### 2.6. Phylogenetic analisys

All proteomic phylogenetic trees were built using the neighbor-joining (NJ) method through the Ape version 5.5 package (Paradis and Schliep, 2019), performing bootstrap (bp) with 1,000 replicas. Only branches with bp >70% were considered. For previous studies employing bp calculation in tree construction in alignment-free analyses, see references (Wu et al., 2009; Fan et al., 2015).

We built a consensus phylogenetic tree for the 8,720 Brazilian proteomes based on the proteome's vectors distance matrix. In addition, phylogenetic trees were built for cluster and lineage centroids, selecting the sequence closest to each corresponding centroid, and taking it as a representative vector. The centroids were obtained by the average of the vectors within the cluster/lineage.

The proteomic results were compared to a phylogenetic tree with the aligned genomes of the clusters and lineages centroid. We also aligned the specific sequences and constructed genome trees to analyze the origin of the P.1 variant. For this step, the maximum likelihood method of the MEGAX 10.2.6 (Kumar et al., 2018) tool, with a 500 bp size, was performed using the Jukes-Cantor nucleotide substitution model (Jukes and Cantor, 1969). The alignment was made using the Nextclade online tool (Hadfield et al., 2018). All the phylogenetic trees were rooted using the Wuhan reference sequence (NC_045512.2) as the outgroup. Finally, we visualized the trees in the iTOL tool^6^ (Letunic and Bork, 2021) and with the ggtree package (Yu et al., 2017).

### 2.7. P.1 variant origin analysis

In order to determine the origin of the P.1 variant, whether internal or external to Brazil, we obtained the 70 closest worldwide samples to each of the 50 P.1 Brazilian samples in 2020 by distance, resulting in 91 unique vectors whose phylogeny by alignment was analyzed. We also searched for occurrences of sequences like P.1 in the world before its emergence in Brazil. Finally, we assessed the involvement of the P.1 variant in possible recombination events.

### 2.8. Machine learning for P.1 search

An ensemble of 50 feed-forward neural networks (multilayer perceptron, MLP) was trained using the vectors of the Brazilian sequences classified as P.1 and non-P.1 utilizing data from release 609 with classification PANGO v.3.0.5, data until the end of 2020. Data division was 70:30 for training and testing sets, respectively, randomly divided for each neural network training of the ensemble, aiming to avoid overfitting. Each MLP network contained input, middle, and output layers with 600,3,1 neurons, respectively. We defined the topology experimentally, and all tests showed similar performances. Only networks with an f1-score higher than 90% as a threshold compose the ensemble. A majority vote decided classification. We validated the model with the complete set of Brazilian vectors until 2021 of accuracy, f1-score, recall, and precision through cross-validation. Finally, we searched P.1 in the 2020 worldwide data.

### 2.9. Recombinant's detection

Possible recombinants were detected from aligned genomes using RAPR (Song et al., 2018) and RDP4 (Martin et al., 2015) tools. RDP4 provides the methods RDP (Martin and Rybicki, 2000), BOOTSCAN (Salminen et al., 1995), MAXCHI (Smith, 1992), CHIMAERA (Posada and Crandall, 2001), 3SEQ (Boni et al., 2007), GENECONV (Padidam et al., 1999), LARD (Holmes et al., 1999), and SISCAN (Gibbs et al., 2000) applied in this task. The confirmation test for the recombinant events was performed by analyzing the phylogenetic trees of genomes. The genomes were split into two parts at the breaking points of the aligned sequences, and we phylogenetically analyzed the relative position between supposed recombinants and their parents. Finally, the recombinants that presented a distinct relative position between the trees of each segment were validated (Zhu et al., 2020).

## 3. Results

From the 1,000,558 samples worldwide in release 409 on the GISAID platform (Elbe and Buckland-Merrett, 2017), 493,080 sequences of proteomes remained after filtering (49%), of which 260,759 sequences were from 2020. In total, 65% of the Brazilian samples were considered, a quality percentage higher than the world average and the other countries studied, as shown in Table 1. Notably, 8,720 vectorized Brazilian sequences were analyzed and discussed later. More detailed information on the results is in Section 2 of Supplementary material 1. The complete metadata of the Brazilian sequences and metadata referring to other countries and the world is available at the Github link.

### 3.1. Landscape in Brazil

The ConsensusClusterPlus analysis returned 15 clusters representing the epidemic proteomes in Brazil from 25 February 2020 to the end of May 2021. More than 15 clusters do not provide a considerable increase in the consensus value of the CDF curve (<5% is shown in Supplementary Figure S2). The main lineages identified in Brazil according to the PANGO nomenclature are as follows: P.1 (3,572–40.9%), P.4 (1,274–14.6%), P.2 (1,132–13.0%), B.1.1.33 (909–10.4%), B.1.1.28 (864–9.9%), B.1.1.7 (248–2.8%), B.1.1 (186–2.1%), P.1.2 (153–1.7%), N.9 (81–0.9%), B.1 (65–0.7%), B.1.195 (54–0.6%), and other (178–2.0%). Some variants were completely grouped in single clusters (B.1.1.7, P.2, P.1.2, B.1.1.33), while others occurred in various groups divided into subvariants (B.1.1.28 in clusters 1, 5, and 6; P.1 and P.4 in clusters 3,7,9,10,14 and 15). Rarer variants were mainly grouped in clusters 2 and 6. Cluster 2 is composed of lineages less frequent in Brazil, including the basal lineages A.1, A.2, B, and B.1, which have 1, 3, 3, and 59 samples, respectively. The Wuhan reference sequence belongs to cluster 2 and is highlighted in **Figure 2**.

**Figure 1:**
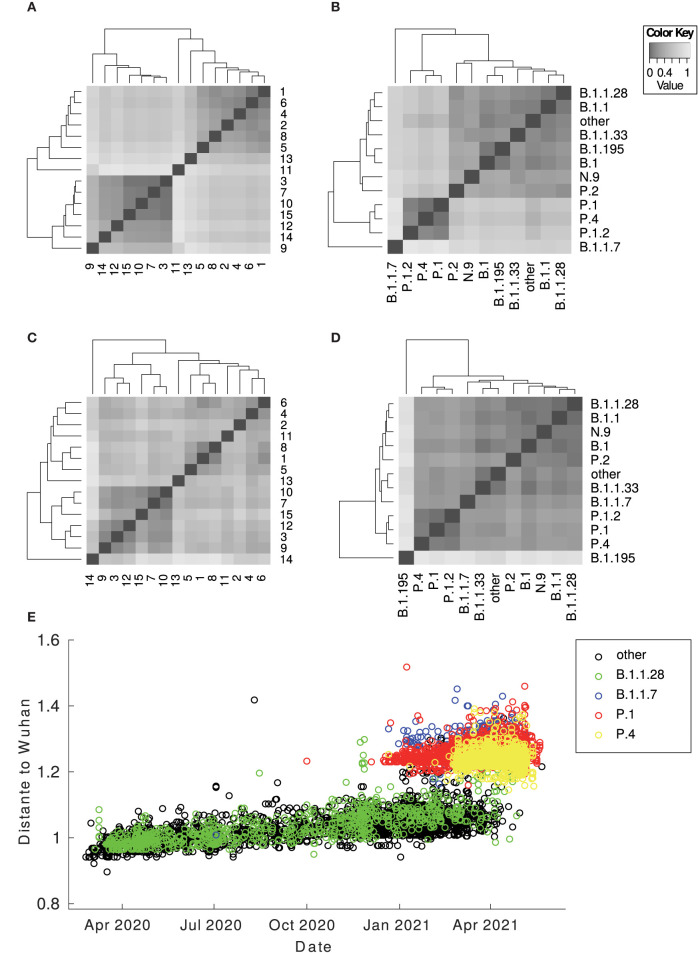
Heatmap of the centroid distance matrix. Distances regarding genomes and proteomes were analyzed and grouped by lineages and by clusters. The images below correspond to **(A)** proteomes by clusters; **(B)** proteomes by lineages; **(C)** genomes by clusters; **(D)** genomes by lineages. The image **(E)** corresponds to the Euclidean distance between the Wuhan vectorized sample and the Brazilian ones against time. There is a considerable gap between the Brazilian sequences in general (B.1.1.28 and other variants from T0 to the TP1 group) (P.1 and P.4 according to PANGO v3.0.5) and different imported sequences (B.1.1.7). The outlier sequences were removed from the visualization.

**Figure 2:**
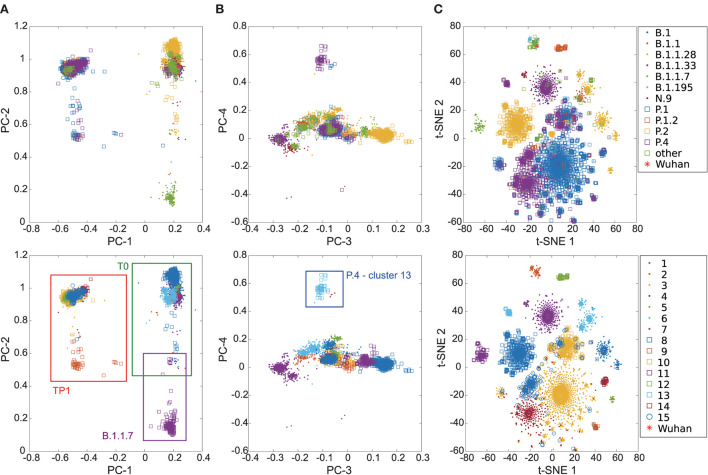
Overview of the relationship between SARS-CoV-2 proteomes of Brazilian samples. The colors represent the main lineages (above) and clusters (below) for the Brazilian sequences for each image pair. The red asterisk highlights the position of the Wuhan reference sequence. **(A)** components 1 and 2 of the PCA graph—note the cluster on the left is the TP1 group, composed of sequences from P.1, P.1.1, P.1.2, and P.4; **(B)** components 3 and 4 of the PCA graph—the cluster 13, isolated above, corresponds to the 79 samples identified as P.4 by PANGO (v3.0.5). The TP1 group is more central, and the other clusters are around it; **(C)** t-SNE graph—note clustering coincides with classification by lineages.

The analysis showed that the clustering approach respects evolutionary similarity among the sequences. Moreover, the clustering results match the PANGO division, as viewed in the t-SNE diagram, PCA, and clusters/lineage centroids heatmap (Figures 1, 2). Table 2 and Supplementary Table S2 show the relationship between the clusters and their main composition. Other results are presented in the Supplementary Table S1.

**Table 2:** Division of lineages into clusters using the complete vectorized proteome.

**Cluster**	**Predominant lineage**	**Number of samples**	**First case**	**Last case**
Groups related to the P.1 variant-TP1		5,000	2020-10-01	2021-05-20
3	P.1	3,062	2020-10-01	2021-05-20
7	P.4	754	2020-12-21	2021-05-07
9	P.1 + P.4	50	2020-12-23	2021-05-06
10	P.1 + P.4	665	2021-01-11	2021-05-10
12	P.1.2	104	2021-02-22	2021-05-17
14	P.1	81	2021-01-16	2021-05-13
15	P.4	284	2021-02-19	2021-05-19
Early group–T0		3,391	2020-02-25	2021-05-22
1	B.1.1.28	477	2020-03-09	2021-04-27
2	B.1.195 + other	179	2020-02-25	2021-05-22
4	B.1.1.33 + N.9	1,037	2020-03-01	2021-04-19
5	B.1.1.28	77	2020-07-31	2021-04-03
6	B.1.1.28 + B.1.1 + other	531	2020-02-28	2021-04-25
8	P.2	1,090	2020-04-13	2021-04-30
Others		329	2020-12-21	2021-05-14
11	B.1.1.7	250	2020-12-21	2021-05-06
13	P.4	79	2021-02-17	2021-05-14

The predominant strains in each cluster are listed with the date of their first and last sample. The complete list of observed lineages by cluster is available in Supplementary Table S2.

### 3.2. Groups analysis

The Brazilian samples are divided into two main groups: the early Brazilian group with 3,391 representatives (here named T0) and the representatives related to the P.1 variant with 5,000 samples (TP1). In addition, two specific clusters that consistently drift apart from the other samples are cluster 13, emerging P.4, within 79 pieces, and cluster 11, imported variant B.1.1.7, within 250 samples (both clusters 13 and 11 are clearly separated from other clusters, as shown in Figure 1). The t-SNE diagram (Figure 2C) shows partially overlapping clusters 3, 7, 10, and 15, composed mainly of variants P.4 and P.1, this likely occurred due to dimensionality reduction. Clusters 1, 2, 4, 5, 6, and 8 compose the T0 group (Figure 2A, right), and clusters 3, 7, 9, 10, 12, 14, and 15 compose the TP1 group (left). In particular, the 1x2 components of PCA show group B.1.1.7 below far apart, and the 3 x 4 components of PCA (Figure 2B) show cluster 13 above, far away from the others.

We also vectorized and clustered spike proteins sequences which derived eight consensus clusters (Supplementary Figure S3 and Supplementary Tables S3, S4). The clustering of the spike proteins was similar to those of the complete proteomes but with fewer divisions. Nevertheless, the division into two larger groups is maintained, and clusters 11 and 13 are still differentiable, as shown in the PCA (Supplementary Figure S3b).

**Figure 3:**
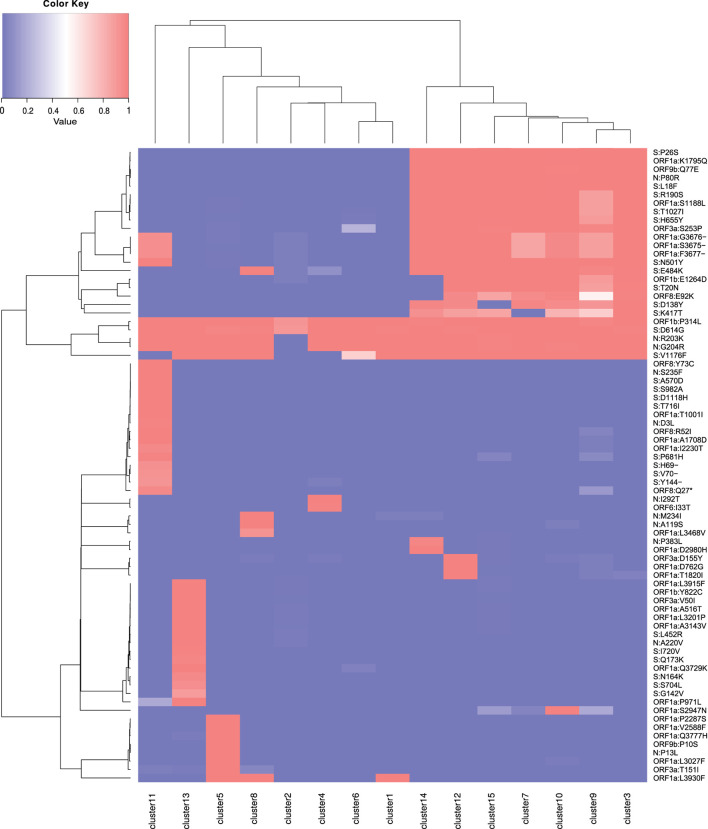
Heatmap of mutations by cluster. Mutations present in 75% of the samples from one or more clusters are listed. The value 1 (red) represents the presence of the mutation in 100% of the cluster samples, and the value 0 (blue) indicates the absence. Values are normalized by cluster.

The consensus mutations for all SARS-CoV-2 Brazilian samples, the characteristic mutations for the TP1 group, and for the other clusters are presented in Supplementary Tables S5–S7, respectively, and can be visualized in the heatmaps of Figure 3 and Supplementary Figure S5 for clusters and lineages. More detailed information is available in Section 2.2 of Supplementary material 1.

#### 3.2.1. Early group (T0)

**Figure 4:**
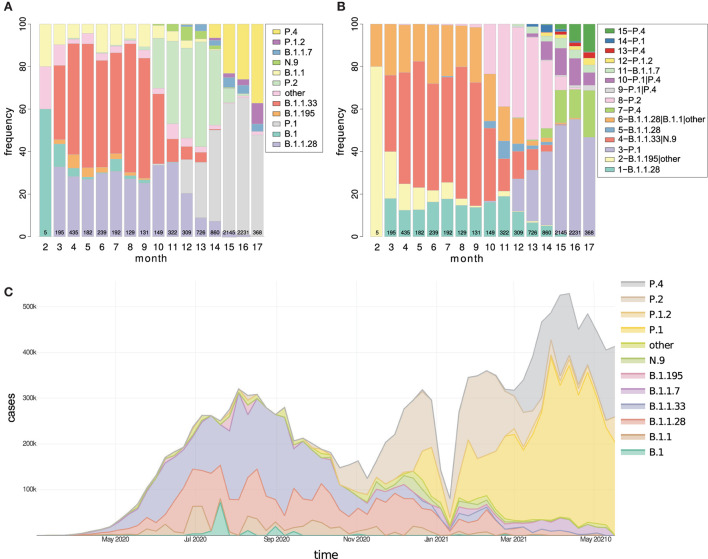
The temporal distribution of **(A)** lineages and **(B)** clusters in Brazil are represented as proportional stacked bar charts divided by months from February 2020 to May 2021. Additionally, in **(C)**, the number of COVID-19 cases registered in Brazil is proportionally divided by lineages. Currently, in Brazil, there is a considerable increase in the number of clusters possibly linked to the diversification of variants, and the strains P.1, P.4, and P.1.2 stand out. The values inside the bars indicate the number of sequencing performed each month. PANGO version 3.0.5 was used.

The T0 group (clusters 1, 2, 4, 5, 6, and 8) is composed of clusters of sequences from the early entry of the virus in Brazil at the beginning of 2020, and daughter lineages evolved locally. The group is mainly composed of B.1.1, B.1.1.28, B.1.1.33, P.2, N.9, and N.10 (Supplementary Table S2). These are the older groups that are predominantly found in Brazil in 2020, but are almost extinct, giving way to the TP1 group (Figure 4). There are no consensus mutations characteristic in T0 (Supplementary Tables S5, S7); each cluster represents an individual lineage or a group of lineages less frequent in Brazil. One example of a variant belonging to T0 is the B.1.1.33, which stood out the most in 2020 in Brazil. Franceschi et al. (2021) suggest that this variant (B.1.1.33) probably originated in Europe and later spread into America. The Brazilian B.1.1.33 sequences are closer to the B.1.1 sequences found in Switzerland (EPI_ISL_415454, EPI_ISL_524474, EPI_ISL_415700, EPI_ISL_415457, and EPI_ISL_429203), Czech Republic (EPI_ISL_416743 and EPI_ISL_895731), and Netherlands (EPI_ISL_454750), corroborating its European origin (Franceschi et al., 2021).

#### 3.2.2. Groups related to variant P.1 (TP1)

TP1 comprises clusters within variants P.1, P.4, P.1.1, and P.1.2 (clusters 3, 7, 9, 10, 12, 14, and 15). The first P.1 was notified in December 2020, though previous studies estimate that P.1 origin in Brazil occurred between early October and mid-November 2020 (Faria et al., 2021). The P.1 sample (EPI_ISL_2241496) dated 01 October 2020 from Paraíba State corroborates this hypothesis. Brazil had one of its lowest sequencing months in October 2022, which may be due to underreporting of P.1-related cases. Remarkably, this month was a period of flexibilization of international flights in Brazil (BBC News-Brazil, 2020).

Phylogenies show that P.1 and P.4 variants mix themselves among and inside clusters in TP1 (Supplementary Figure S6). In t-SNE, PCA, and heatmaps, P.1 and P.4 are hardly distinguishable, either in clusters or lineages (Figures 1, 2). Furthermore, the TP1 clusters share many non-synonymous mutations (Figure 3 and Supplementary Table S6). At least five of these mutations are in the spike protein, conferring the adaptive virus advantage (E484K, N501Y, K417T, H655Y, and L18F) (Colson et al., 2021; Gan et al., 2021; Grabowski et al., 2021; VanInsberghe et al., 2021). The sum of these characteristics suggests that the TP1 group could be seen as a single lineage, divided into sublineages. As stated before, it is also remarkable that clusters within TP1 do not correspond perfectly to the P.1 and P.4 subdivisions provided by PANGO.

#### 3.2.3. Cluster 11–variant B.1.1.7

Cluster 11 is composed of B.1.1.7, comprising 250 sequences, which had its first case identified in Brazil on 12 December 2021. The characteristic mutations of the group correspond to those found in the literature (Davies et al., 2021) (Supplementary Table S7). Furthermore, the smallest distances between cluster 11 and world-2020 samples indicate their closest similarity with sequences from England (EPI_ISL_799516, EPI_ISL_1248398, EPI_ISL_760286, EPI_ISL_797822, and EPI_ISL_799518), all belonging to the British B.1.1.7 variant. Therefore, it reinforces the possibility that the entry of the variant in Brazil occurred directly from England.

#### 3.2.4. Cluster 13–variant P.4

Cluster 13 comprises 79 sequences classified as P.4, as designated by PANGO v3.0.5 (2021-06-04); however, mutations do not correspond to the TP1 group to which the P.4 variant belongs (Figure 3). Later modifications in the PANGO nomenclature (v3.1.11 2021-08-09) changed P.4 classification which will be covered in more detail in the discussion. This cluster is an attention-grabbing group because it contains many unique mutations, three of which are of concern (Supplementary Table S7). This group of mutations was not found in other locations but only in Brazil (according to a search carried out on Outbreak.info). The 3 x 4 components of PCA (Figure 2B) placed the cluster 13 group away from the other clusters in the same way that occurred with the samples from B.1.1.7, indicating a possible late entry, but we could not track its origin.

### 3.3. Phylogenetic trees

Although most analyzes of this study were performed based on proteome samples, the complete genome DNA trees were built for comparison. The results showed that the proteome and genome-derived trees with 8,720 samples generally agree (Supplementary Figure S6), complete trees are available in Supplementary Figures S5, S6.

**Figure 5:**
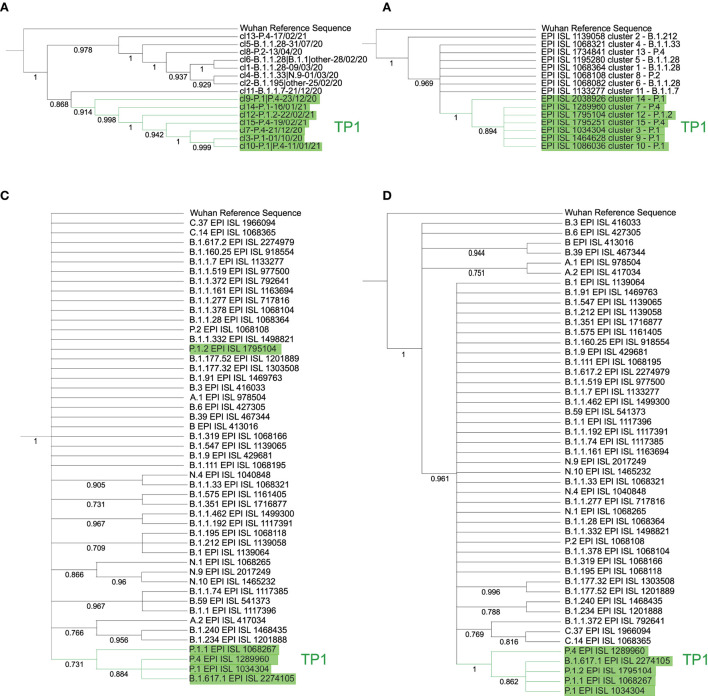
Phylogenetic trees by cluster and lineage centroids (relative to PANGO v3.0.5) with a bootstrap of 1,000 replicas. Left, the neighbor-joining method with Euclidean distance was used on the proteome centroids corresponding to each **(A)** cluster and **(B)** lineage. Right, centroid trees per cluster by DNA *via* maximum likelihood with JukesCantor method, by **(C)** cluster and **(D)** lineage. The tree **(A)** is divided into two main branches: the TP1 group, with cluster 11 of B.1.1.7 as more basal, followed by cluster 9; and the other main branch, the T0 with cluster 13 as the most basal. All branches reach bootstrap greater than 85%. The **(B)** is less consistent, but the TP1 group reaches a 73% bootstrap, except for the branch of P.1.2, which does not reach high BP. The **(C)** consistently inserts cluster 14 as basal in the TP1 group and cluster 2 as more basal in Brazil. Finally, tree **(D)** obtains BP of 100% for the branch of the TP1 group and inserts as basal the variants A.1, A.2, B, B.3, B.6, and B.39 consistently.

The consensus tree consistently grouped the monophyletic branch of the TP1 group with 100% BP. The branch containing cluster 12 (P.1.2), internal to the branch of the TP1 group, is monophyletic and obtained a 100% BP. The cluster 11 (B.1.1.7), with 87% BP, and the lineages N.9 and N.10 of cluster 4, both with 100% BP, are also monophyletic (tree available in Github as SARS_NJ_Consensus_BP.nwk). Centroids-based phylogenies provided a cleaner and more reliable evolutionary overview of the groups with high bp (Figure 5). TP1 group appears together in all tested centroid trees with high bp.

The TP1 group is cohesive and monophyletic in all approaches, and the P.4 lineage does not differ from P.1, as there is an alternation of branches in all trees, both in genome and proteome (Supplementary Figure S6). The basal cluster of the TP1 group is uncertain, varying between cluster 14 in the genomic approach and cluster 9 in the proteomic one. Cluster-based methods reached higher bp values compared to those based on the lineage (Figure 5). The clusters obtained are supported by phylogenetic analysis, obtaining a large overlap, with point divergences, as shown in Supplementary Figure S6.

Kappa variant B.1.617.1 samples appear together within the TP1 group in the DNA and the protein trees (Figures 5B,D), which probably consists of annotation errors once these samples have the characteristic mutations of the P.1 variant rather than B.1.617.1 (Supplementary Table S1).

### 3.4. P.1 variant origin analysis

We investigated three hypotheses for the P.1 variant origin: a) it evolved locally, i.e., from T0, b) it had a later entry external origin (came from abroad); and c) P.1 is derived from some recombination event.

**Figure 6:**
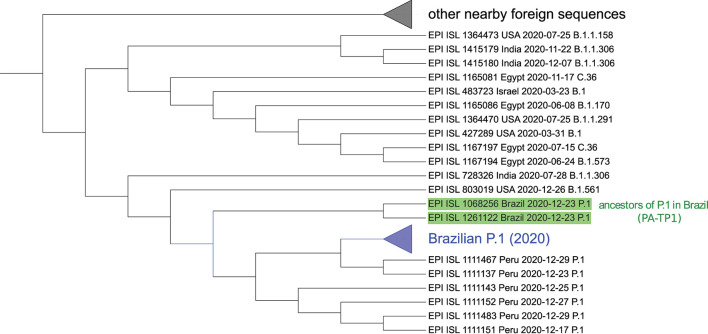
Sequences similar to P.1 detected by the neural network ensemble. The two identified ancestral sequences from the P.1 lineage in Brazil (PA-TP1) are highlighted in green. The collapsed branch in blue groups 48 of the 50 Brazilian P.1 samples and is close to Peruvian. Neither instance of B.1.1.28 was identified in the group nor other Brazilian sequences besides P.1. The collapsed branch above groups different foreign arrangements similar to P.1. The complete tree is available in Supplementary material 7. Graphic obtained with the iTOL tool—https://itol.embl.de/ (Letunic and Bork, 2021).

From each of the 50 P.1 samples (Brazilian), we take the 70 closest vectors in the set of world proteomes of 2020. This search identified 91 unique sequences, including 6 Peruvian P.1 and 17 Brazilian B.1.1.28 samples, and others from several countries, as listed in Supplementary Table S8. This isolated information would indicate that P.1 is closely related to the B.1.1.28 sequences from the Pará (PA) and São Paulo (SP) states, supporting the local ancestry hypothesis previously reported (Naveca et al., 2021). However, the two samples nominated here as PA-TP1 (EPI_ISL_1068256 and EPI_ISL_1261122) have as closest sequences only foreign samples, as shown in Table 3. These samples also appeared close to all other searched P.1 samples then we deepened the analysis. The PA-TP1 genomes have 10 of the 17 characteristic mutations of the P.1 group, and both instances belong to cluster 9. The PA-TP1 characteristic mutations of the P.1 group are S:E484K; S:N501Y; S:L18F; S:P26S; N:P80R; ORF1a:K1795Q; ORF3a:S253P; ORF9b:Q77E; S:K417T; S:D138Y. Thus, the phylogeny suggests that PA-TP1 may be the precursor of the TP1 group in Brazil (Figure 6 and Supplementary Figure S7), and cluster 9 may be ancestral to the P.1 variant. The PA-TP1 mutations have important effects, as listed in Supplementary Table S6. Of these, we highlight S:E484K and S:N501Y associated with high transmissibility and low vaccine efficiency; the S:L18F mutation which compromises the immune response; and S:K417T that promotes high affinity with ACE2 and resistance to antibodies.

**Table 3:** Worldwide sequences close to P.1 ancestors (PA-TP1) in 2020.

**epi**	**ID**	**Date**	**Variant**
EPI_ISL_831339	hCoV-19/USA/NC-UNC-0017/2020	2020-04-00	B.1.1.1
EPI_ISL_530145	hCoV-19/USA/WA-S2788/2020	2020-08-12	B.1.1
EPI_ISL_530128	hCoV-19/USA/WA-S2771/2020	2020-08-01	B.1.1
EPI_ISL_525755	hCoV-19/USA/WA-S2765/2020	2020-08-03	B.1.1
EPI_ISL_954139	hCoV-19/NorthMacedonia/29205/2020	2020-12-23	B.1.1.428
EPI_ISL_555709	hCoV-19/England/ALDP-952525/2020	2020-06-09	B.1.1
EPI_ISL_1301549	hCoV-19/Mexico/HID-InDRE-IBT-66/2020	2020-06-02	B.1.1
EPI_ISL_729470	hCoV-19/Germany/SH-ChVir8194/2020	2020-07-19	B.1.1
EPI_ISL_700185	hCoV-19/India/MH-ACTREC-539/2020	2020-08-25	B.1.1.306
EPI_ISL_745223	hCoV-19/Russia/MOS-CRIE-7182855/2020	2020-08-24	B.1.1

The list was obtained using the distance from PA-TP1 to the 2020 world samples.

In the tree based on aligned genomes (Supplementary Figure S7), we included the 50 Brazilian P.1 sequences from 2020, the 91 closest samples, including B.1.1.28 from cluster 6 and the B.1.1.28 sequences (EPI_ISL_1068137, EPI_ISL_801387, EPI_ISL_801397, EPI_ISL_801398, EPI_ISL_801389, EPI_ISL_801392, EPI_ISL_801394, EPI_ISL_801395, EPI_ISL_801399, EPI_ISL_801401) indicated by Naveca et al. (2021) as belonging to the ancestral clade of the P.1 lineage. The P.1 group achieved a BP of 100% in its branch (Supplementary Figure S7) and is a sister group of a branch divided into a consistent branch of B.1.1.198 and another branch that includes samples of B.1.1 and B.1.1.192. The P.4 variant, corresponding to cluster 7, is a descendant of the P.1 strain (cluster 3) since the first sample of P.4 (hCoV-19/Brazil/AM-CD1739/2020–EPI_ISL_2233906) is a sister group of an Amazonian strain of P.1, indicating its probable place of origin, with BP of 86%. The 100% BP corroborates that PA-TP1 are ancestors of the P.1 lineage in Brazil. However, it was impossible to confirm the ancestor of the TP1 group since the BP was low in all other basal branches, including the samples indicated by Naveca et al. (2021) as the P.1 ancestral clade. This same phylogeny suggests that the Peruvian lineage of P.1 descended from the Brazilian P.1 lineage.

We measured the distances between proteomes from Brazilian samples and that of the Wuhan reference proteome over the pandemic period (Figure 1E). The differences between P.1 and P.4 and the Wuhan reference are much higher than the distance between the T0 group to Wuhan. From the beginning of 2021, the average distance among the Brazilian samples concerning the Wuhan sample leaped with the TP1 emergence as T0 group variants became extinct. Therefore, the distancing of the TP1 group from T0 is abrupt and not gradual.

The objective to construct the neural network ensemble was to search for P.1 like sequences in worldwide samples of 2020, before the emergence of P.1 in Brazil. After all results of the cross-validation over the complete set of Brazilian proteome samples using the trained ensemble of neural networks for the P.1-true and P.1-false classes, we obtained f1-score of 99.39%, accuracy of 99.5%, precision of 99.05%, and recall of 99.72%. These results confirm the separability of the P.1 samples from other Brazilian strains. The search in world 2020 data found 129 records of P1-like organisms, including 50 Brazilian P.1 from 2020 and an additional 79 from other countries. This shows there were already, in 2020, viruses like the Brazilian P.1 variant circulated the world before its emergence in Brazil. The phylogenetic analysis of these samples presented the same PA-TP1 samples, mentioned above with 10/17 mutations, as ancestral of P.1 variant (Figure 6). The proteomes closest to the origin of P.1, from those identified by the network, are one from the USA (EPI_ISL_803019) labeled B.1.561, and one from India (EPI_ISL_728326) identified as B.1.1.306.

The proximity of the 48/50 P.1 sequences to a particular subgroup of B.1.1.28 samples in Brazil, above mentioned, led us to consider a possible recombination event involving B.1.1.28 and P.1 variants. Therefore, we provide a list with the 91 closest samples, the 50 Brazilian samples, and the ones suggested by Naveca et al. (2021) for the recombinant event search tools RDP4 and RAPR.

The RDP4 found one possible recombinant event: hCoV-19/Lithuania/MR-LUHS-Eilnr352/2020 (EPI_ISL_636871–B.1.1.280) as a recombinant sample, hCoV-19/Brazil/AM-CD1739/ 2020 (EPI_ISL_2233906–P.4) as minor parental, and hCoV-19/England/OXON-AD15D/2020 (EPI_ISL_448567–B.1.1.10) as major parental. This indicative comes with the observation that the recombinant may be a parent since the “minor parental” has not been precisely identified. The methods applied by RDP4 and their respective *p*-values are RDP (3.92E-04), GENECONV (6.26E-03), Bootscan (2.46E-03), Maxchi (1.35E-02), Chimaera (6.28E-03), and 3Seq (1.36E-05).

RAPR results (Supplementary Table S9) suggested the hypothesis that the proximity between P.1 and few samples of B.1.1.28 from cluster 6 may be due to a recombination event between a Brazilian P.1 and a foreign strain, close to hCoV-19/USA/NC-UNC-0017/2020 (EPI_ISL_831339–B.1.1.1), which originated this group of B.1.1.28. Among the samples indicated as recombinant is the Brazil/AM-FIOCRUZ-20890261MV (EPI_ISL_801402–B.1.1.28—Supplementary Table S9). This sample belongs to clade 28-AM-II (A6613G) of B.1.1.28, indicated as the ancestor of lineage P.1 according to Naveca et al. (2021) (Supplementary Figure S7). Such clade has the A6613G mutation, a characteristic mutation of the TP1 group, present in 99.9% of the samples in the group. Therefore, to reinforce the recombination possibility, we built phylogenetic trees with the sequence before the alignment breakpoint and the other after this point. However, the trees did not reach a high enough bp to confirm or rule out any recombination events suggested by the tools (Supplementary Figure S7).

### 3.5. Temporal and spatial distribution of lineages

The distribution of variants by state (Supplementary Figure S8) showed that only 4 of the 27 states had samples continuously sequenced along with pandemics till May 2021: SP, RJ, RS, and BA. In other states, there were months without sequencing or simply one-off analyses. In the first phase of the pandemic, variants B.1.1.28 and B.1.1.33 predominated until October 2020, after which time the P.2 variant predominated. Thus, from December 2020 until March 2021, the P.1 variant grew to become the primary variant in the country, followed by February 2021 variant P.4 (Figure 4). Detailed information for the Brazilian States is available in Section 2.3 of Supplementary material 1.

The second epidemic wave of SARS-CoV-2 was more significant than the first, and its beginning coincides with the emergence and rise of P.1 (Figure 4C), as already reported (Franceschi et al., 2021; Naveca et al., 2021). Refer to Supplementary material 4. Over time, the lineages and clusters graphs illustrated how the T0 group prevalence decreased and was probably extinct (or occurred in small quantity), with variants TP1 and the imported groups, B.1.1.7 and the new variant of cluster 13, becoming dominants in Brazil (Figure 4). This transition is more evident when the evolution of the pandemics in the PCA and t-SNE graphs is viewed (3D graph of Supplementary Figure S9, and the Supplementary material 3, 4). Furthermore, looking at the development of the lineages over time, we notice a pattern in the origin of new variants, characterized by the formation of new clusters (discussed later).

### 3.6. Diversity of SARS-CoV-2 proteomes in Brazil

The study of SARS-CoV-2 diversity enabled both: i) understanding the distribution of the variants in the viral population in Brazil (richness) and ii) verifying and comparing the degree of sequencing in different Brazilian states and to compare with other countries (coverage). We exploited the concepts of richness and coverage as defined in Methods, and the results are presented in Section 2.4 of Supplementary material 1.

## 4. Discussion

The COVID-19 pandemic is a catastrophic event with severe consequences, leading to losses in almost all human activities, mainly health and the economy. On the other hand, we have a rare opportunity to observe the evolution process in almost real time; since it promotes a rush for genome sequencing of a single virus species never seen before. Recent bioinformatics technology provides resources to analyze the big data provided by these efforts and allows us to draw a panoramic view of the SARS-CoV-2 evolution in Brazil and worldwide.

The pandemic in Brazil had two moments (Figure 4):

1. T0 - the early entry of SARS-CoV-2, which occurred throughout 2020 to early 2021, characterizing the T0 group in this study embodies many lineages that disappeared over time, the prevailing lineage were B.1.1.28 and B.1.1.33, and later the emergence of P.2 occurred.
2. TP1 - detected between Dec 2020 and Feb 2021, is characterized by groups related to the P.1 (Gamma) variant and other late imported foreign strains, including B.1.1.7 and P.4 of cluster 13.

We observed that strains tend to be extinct and replaced by newer and more adapted strains holding more advantageous mutations, as observed in other studies (González-Candelas et al., 2021; Naveca et al., 2021). This lineage substitution process was followed in Brazil on several occasions, as in the emergence of the P.2 variant and later of the TP1 group (Figure 4).

Our proposed pipeline^7^ (Supplementary Figure S1) allowed us to recognize the appearance of new variants. New variants emerged by moving away from the parental lineage, in a process called “exploitation of the mutational space,” which becomes graphically visible by the methods of PCA and t-SNE (Supplementary Figure S4), followed by the establishment of a new cluster. We observed a remarkable variant emergence event during the analysis, the origin of cluster 7, a sublineage of P.1 (cluster 10) composed of 126 samples from the State of São Paulo (called P.1-SP at first moment—Supplementary Figure S4). These samples had their designation updated from P.1 to P.4 between v2.3.8 and v3.0.5 of PANGO.

There is no reliable phylogenetic analysis of SARS-CoV-2 in the literature. Furthermore, the high mutation rate associated with the large volume of circulating viruses strains in the world entails frequent cases of parallel and backward mutations, resulting in inconsistencies in the determination of lineages and hindering the reconstruction of their evolutionary relationships (González-Candelas et al., 2021), and difficulties are also reported in the survey (Morel et al., 2021). Therefore, we performed a phylogenetic analysis of complete SARS-CoV-2 genomes/proteomes based on vectorial distance matrices. We compared trees based on representing lineage centroids with those based on representing cluster centroids. The cluster centroid-based phylogenetic trees showed to be consistent (bp >85% in all branches), while the lineage centroid-based tree presented a much lower BP and did not show clear differentiation in the evolutionary history of the lineages. It led us to conclude that the division of specimens by clustering is more reliable to the evolutionary mapping and that some inconsistencies may be present in SARS-CoV-2 classification by PANGO. Therefore, the clustering approach presented in this study may help revise the lineage's nomenclature process. In addition, the use of proteomes (amino acid representation) in the evolutionary analyses and the heatmaps (Figures 1, 5) showed more consistency than the use of genomes (DNA) in both cluster and lineage divisions. As a consensus across all methods, the TP1 group consistently clusters in a single branch, away from the other Brazilian variants.

Our analyses showed that the clustering method groups the sequences by evolutionary similarity, making it suitable for classification tasks even for nomenclature purposes. In addition, the results of the proteomic evolutionary analyses were more consistent than the genomic ones and, therefore, ideal for this analysis. Therefore, the proposed pipeline is based on proteomic sequences.

Based on the results, we suggest three plausible hypotheses for the P.1 variant origin: (a) origin from variant B.1.1.28 in Brazil, as reported by Naveca et al. (2021), (b) a foreign origin from a late entry strain, and (c) P.1 variant was originated by some recombinant event.

The phylogeny in Supplementary Figure S7 does not support the lineage B.1.1.28 as an ancestor of P.1. We cannot, however, conclusively rule out the possibility of a Brazilian origin for P.1 since there is a gap in the sampling in the period of the emergence of P.1 in Brazil around October 2020. However, the accumulating body of evidence consistently points to an external P.1 origin:

1. The considerable distance (Euclidean and phylogenetic) and different clustering between P.1 and the previously reported ancestor B.1.1.28 samples (Figures 1A–D, 2, 5 and Supplementary Figures S6, S7);
2. Foreign sequences are closer to PA-TP1 than any Brazilian samples of the T0 group (Table 3);
3. The distance from the Wuhan reference sample is much higher to P.1 than to the other Brazilian instances in 2020 (Figure 1E);
4. There are many accumulated mutations in P.1 without intermediate sequences detected in Brazil (Figure 3 and Supplementary Figure S5);
5. The machine learning approach found P.1-like SARS-CoV-2 samples circulating the world before the variant emergence in Brazil (Figure 6).

The external VOC P.1 entry in Brazil may have been favored by the flexibilization of measures including international flights in Brazil in October 2020 (BBC News-Brazil, 2020), which became the period of entry/emergence of P.1 in Brazil, also suggested by Faria et al. (2021). After the entry of P.1 in Brazil, the mutations S:H655Y, S:T1027I, S:R190S, S:T20N, ORF1a:S1188L, ORF8:E92K, and ORF1b:E1264D probably originated in Brazil, since they are not present in the ancestral PA-TP1, assuming these samples as reference. Among these mutations, the S:H655Y promotes immune system escape (Colson et al., 2021). However, as listed in Section 3.4, PA-TP1 already has important mutations, and according to our analysis, all of these mutations come from a foreign origin.

Recombination is a common phenomenon in the Coronaviridae family (Zhu et al., 2020); however, there are indications that recombinant events between SARS-CoV-2 strains are rarer than expected (Varabyou et al., 2021). Our results indicate no recombination event in the origin of the P.1 variant; however, such an event can relate to B.1.1.28 and P.1 variants. The RAPR tool results indicate that a subgroup of B.1.1.28, a subset of cluster 6 in our study, the same group identified as 28-AM-II (A6613G) clade by Naveca et al. (2021), was originated by recombination between a P.1 and a foreign sample close to the hCoV-19/USA/NC-UNC-0017 (EPI_ISL_831339–B.1.1.1) (Supplementary Table S9). Thus, our analysis points to the possibility that clade 28-AM-II comes from recombination, in this case, it is not an ancestor of P.1 but an ancestor of this clade.

We propose Cluster 9 as the probable ancestral cluster of the TP1 group (Figure 5). It contains the PA-TP1 samples, the sequenced strains closest to the ancestors of the P.1 lineage. Furthermore, the hypothesis is supported by the PCA (Figure 2A), which shows cluster 9 as the furthest apart among the TP1 clusters, dispersed as in the described “exploitation of the mutational space” during the origin of new variants, forming a bridge between itself and the other TP1s.

The P.1 variant arrived in Brazil from an external environment, underwent a fast local adaptation, and finally dispersed, causing the second epidemic wave. From our results, we propose that worldwide emerging waves in this pandemic may have arisen through this same process: new variant entry—local adaptation—dispersion/predomination (details see Supplementary Figure S13).

The diversity analysis revealed that coverage of viral subvariants is low in all Brazilian states (Supplementary Table S10), and 13 of the 27 Brazilian states had <100 quality samples until May 2021. São Paulo (SP) and Rio de Janeiro (RJ) states present more sequencing and had 4,386 and 1,170 sequenced samples, respectively. The state of SP is the national center of the pandemic, having the highest virus richness. In addition, SP is the main hub for national and international travel, representing more than 70% of international flights from/to Brazil (Candido et al., 2020). Therefore, it was expected a large circulation of different viral variants in this state. Candido et al. (2020) indicate that, like SP, the states MG, CE, and RJ are major international travel entry centers. For these states, the estimated richness also presented high values (Supplementary Table S10), except CE, that appears to have underestimated richness, likely due to the low sampling. The richness estimations highlight the existence of a much larger number of variants that are not yet sequenced (Supplementary Figure S12 and Supplementary Table S10). In addition, the analyses indicate that Chao's metric combined with vector representation for proteomes is a suitable method for viral diversity analysis (Chao, 1984).

Wealthier countries, such as those in Europe, also presented the highest richness estimations despite the lower number of COVID-19 cases (Table 4). Thus, we hypothesize that European countries, receiving more international inflows, have a higher chance of variants entering, increasing their viral richness, such as in the São Paulo state in Brazil.

**Table 4:** Diversity comparison in selected countries.

**Country**	**Ncases**	**Nseq**	**Nunique**	**Chao**	**Chao**
	**(millions)**			**richness**	**coverage (%)**
Brazil	16.55	8,720	6,164	26,370	23.3
India	28.18	7,154	5,493	33,505	16.4
Italy	4.22	12,784	6,617	21,946	30.1
Germany	3.69	51,880	22,048	63,263	34.8
England	4.50	199,110	62,643	164,070	38.2
World (until March 21)	83.56	493,080	312,224	2,534,900	12.3

Richness and coverage metrics were calculated by Chao 1 method (Chao, 1984). Ncases, number of cumulative cases in the country (in millions); Nseq, number of quality collected sequences (analyzed); Nunique, number of non-redundant samples.

The disproportion in sampling between states (Supplementary Table S12) makes it difficult to compare the evolutionary history of the virus among states. A considerable increase in sequencing occurred in 2021 in Brazil; however, the disproportion remains. The low coverage in regions may hide VOCs, making their tracking hard (Franceschi et al., 2021). In addition, globally, there is a concentration of sequencing. A total of 10 countries account for 85% of the GISAID samples and only 35% of the world's cases. Disproportion in sampling between different countries results in strains remaining undetectable until they become widely spread, and then it is no longer possible to effectively control their dispersion (González-Candelas et al., 2021). We assume that, similarly, the subsampling in Brazilian states corroborated the sudden spreading of the P.1 lineage.

We have observed that the lack of monitoring by sequencing in Brazil has allowed P.1 to spread silently; moreover, we could not trace the origin of its large number of accumulated mutations (17 in all), which make this VOC dangerous. The low sequencing associated with a great richness of variants, observed in countries like India, may lead to the emergence of new VOCs, such as the Indian B.1.617.2 (Delta). Therefore, sequencing should increase, and border control measures will help control the spread of dangerous variants.

Sequence vectorization in this study is a paradigm-breaker since it allows the analysis of large volumes of data where more traditional methods may be inadequate, like in the big genome data generated in the SARS-CoV-2 outbreak. However, due to this reason, the P.1 variant origin is misdefined in previous studies.

### 4.1. Comments on PANGO and GISAID database updates

In the current version of PANGO v3.1.11, all P.4 samples from all clusters have been reclassified as P.1 and subvariants, except for the samples belonging to cluster 13. The heatmaps in Figure 1 show that cluster 13, in fact, does not belong to TP1. It is in accordance with our findings and explains the overlapping observed in Figure 2C concerning the clusters of the TP1 group, based on the previous terminology (Figures 1A–D, 3). In addition, concerning the T0 group, cluster 5 has 77 samples of B.1.1.28 consistently separated from the others, which had its designation updated to variant P.7, which agrees with our analysis. Supplementary Table S13 provides more details about PANGO updates.

## Data availability statement

The datasets presented in this study can be found in online repositories. The names of the repositories and accession numbers can be found in the article.

## Author contributions

RR and CP designed and implemented the analysis. GN contributed to the search and analysis. DF coded the R version of SWeeP. CP wrote the original draft of the manuscript. CD, FP, and ES made substantial contributions, revisions, and approved the final manuscript. RR supervised the whole project. All authors contributed thoughts and advice, discussed the results, and contributed to writing the final manuscript.

## Footnotes

^1^PANGO lineages—https://cov-lineages.org/.

^2^Wuhan reference sequence NC_045512.2—https://www.ncbi.nlm.nih.gov/nuccore/1798174254.

^3^Nextclade—https://clades.nextstrain.org/.

^4^rSWeeP Bioconductor—https://bioconductor.org/packages/release/bioc/html/rSWeeP.html.

^5^Rtsne package—https://github.com/jkrijthe/Rtsne.

^6^iTOL tool—https://itol.embl.de/.

^7^Pipeline available at:https://github.com/CamilaPPerico/SARS-CoV-2_Brazil_Landscape/.
