# Supplementary material for: Genomic landscape of the SARS-CoV-2 pandemic in Brazil suggests an external P.1 variant origin
Source: Front Microbiol. 2022 Dec 22;13:1037455. doi: 10.3389/fmicb.2022.1037455 (PMC9814972; doi:10.3389/fmicb.2022.1037455)
Supplement: Supplementary file 1 [file Data_Sheet_1.PDF]

Supplementary Material 1
Genomic landscape of SARS-CoV-2 pandemic in Brazil suggests an external P.1 variant origin

Camila P. Perico[1,2], Camilla R. De Pierri[1,3], Giuseppe P. Neto[1], Danrley R. Fernandes[1,2], Fabio O. Pedrosa[2,3], Emanuel M. de Souza[2,3], and Roberto T. Raittz[1,2,*]

1. Laboratory of Artificial Intelligence Applied to Bioinformatics - SEPT, Federal University of Paraná, Curtiba, Paraná, Brazil
2. Graduate Program in Bioinformatics - SEPT, Federal University of Paraná, Curtiba, Paraná, Brazil
3. Department of Biochemistry and Molecular Biology, Federal University of Paraná, Curtiba, Paraná, Brazil

Corresponding Author:
Roberto T. Raittz: raittz@ufpr.br

This PDF file includes:

– Description of Supplementary 2-7 (section 1) and other Supplementary material available at https://github.com/CamilaPPerico/SARS-CoV-2_Brazil_Landscape/
– Results in detail (section 2)
– Supplementary Figures and Tables (section 3)
– References

1 SUPPLEMENTARY DESCRIPTION

Supplementary 2:

(Supplementary2_gisaid_hcov-19_acknowledgement_table.pdf)
Acknowledgements to all researchers and professionals who contributed to the GISAID genome database and for this study.

Supplementary 3:

(Supplementary3_tSNE_by_week.pdf)
Lineage evolution over time through visualization with t-SNE graph. Each slide adds the samples of a new week. The colors represent each cluster obtained for Brazilian sequences. Here was adopted the nomenclature PANGO v3.0.5. The key shows the main lineages that each cluster represents. The emergence, growth and extinction of the groups is visible, as well as the gradual replacement of the T0 group variants by the TP1 group.

Supplementary 4:

(Supplementary4_PCA_by_week.pdf)
Lineage evolution over time through visualization with PCA graph. Each slide adds the samples of a new week. The colors represent each cluster obtained for Brazilian sequences. Here was adopted the nomenclature PANGO v3.0.5. The key shows the main lineages that each cluster represents. The emergence, growth and extinction of the groups is visible, as well as the gradual replacement of the T0 group variants by the TP1 group. Note that the second wave was bigger and started with the emergence of P.1 lineage. The information on the number of COVID-19 cases in Brazil was obtained at the official website `https://covid.saude.gov.br/`.

Supplementary 5:

(Supplementary5_BRtree8kProt_160k.nwk)
Complete phylogenetic tree of 8720 Brazilian samples from GISAID release 609 of 2021-06-06 generated by Neighbor-Joining method from the vectorized proteomes (PANGO nomenclature v3.0.5 of 2021-06-04)

Supplementary 6:

(Supplementary6_BRtree8kDNA_65k.nwk)
Complete phylogenetic tree of 8720 Brazilian samples from GISAID release 609 of 2021-06-06 generated by Neighbor-Joining method from the vectorized genomes (PANGO nomenclature v3.0.5 of 2021-06-04)

Supplementary 7:

(Supplementary7_tree_near_P1_world_sequences_from_ANN.nwk)
Phylogenetic Tree of sequences similar to P.1 obtained through the neural network ensemble. The probable ancestral samples PA-TP1 (EPI ISL 1068256 and EPI ISL 1261122) are highlighted in green in the Figure S7. The remaining 48 samples of Brazilian P.1 are close to the Peruvian samples. No sample of B.1.1.28 was identified in the group, nor other Brazilian sequences besides P.1. The branch above groups other foreign sequences similar to P.1. The 'No' in labels indicates that they are not of the P.1 lineage.

Additional data:

Supplementary material available at `https://github.com/CamilaPPerico/SARS-CoV-2_Brazil_Landscape/`:

1. The Pipeline is divided into 9 codes, in order of use, in the "scripts" folder. In all steps, parameters and the name of the input/output files can be edited.
   script 1 Extraction of the proteomes of interest from GISAID's fasta file.
   The input sequence for this step is the file `allprot(release).fasta`, available at GISAID (`https://gisaid.org`). This file contains the proteome of each world sample of SARS-CoV-2. The input for all the following steps are produced by the scripts themselves. In this step, are extracted only the sequences of the country of interest.
   script 2 Concatenation of sequences in multifasta file.
   Concatenates each of the proteins of the country of interest and creates a unique complete proteome sequence (multifasta output). In this step, a file listing the headers and a file with the EPI codes are provided.
   script 3 Selection of quality sequences.
   From the multifasta, are selected only sequences without misreadings (presence of X) and with complete proteomes (with 26 or more proteins). In this step is create only the index list of the quality sequences.
   script 4 Filtering quality sequences.
   The quality sequences index list is used to create a second multifasta, EPI and headers lists only with filtered sequences.
   script 5 SWeeP projection of sequences in vectors.
   The proteomes from filtered multifasta are vectorized by the SWeeP tool. A matrix is obtained in which each row corresponds to a sample. Each sample vector has a length of 600 dimension.
   script 6 Consensus Clustering step.
   The vectorized sequences are grouping by similarity using the clustering method. Parameters of resampling number, maximum number of clusters, clustering algorithm, among others, can be adjusted as needed.
   script 7 Visualization of clusters step.
   The visualization of the sequences by PCA and t-SNE is performed using the vectorized data (SWeeP) based on the division by clusters (Consensus Clustering).
   script 8 Consensus Phylogenetic tree step.
   Obtaining of the consensus phylogenetic tree, with bootstrap values, by the Neighbor-Joining method.
   script 9 Consensus Phylogenetic tree visualization step.
   The visualization of the consensus phylogenetic tree, obtained in step 8. The division by clusters (Consensus Clustering) is highlighted by the colors of the branches. The bootstrap values are shown at the base of each branch.
2. A sample of GISAID release 609 to test the scripts: Sample_GISAID_Release0609.fasta
3. The metadata of the analyzed sequences of Brazil, Germany, India, Italy, UK and World-2020 are available in the "metadata" folder: Metadata_Brazil.csv, Metadata_Germany.csv, Metadata_India.csv, Metadata_Italy.csv, Metadata_UK.csv (in 7 parts), and Metadata_World_2020.csv.
4. Consensus tree of vectorized proteomes, with bootstrap of 1'000 replicas, 8'720 samples, Neighbor-Joining method: SARS_NJ_Consensus_BP.nwk

2 DETAILED RESULTS

We obtained 1'000'558 samples worldwide in release 409 on the GISAID platform Hadfield et al. (2018), and 49% of proteomes remained after filtering (493'080 sequences, of these 260'759 from 2020). Corresponding to the release of 609, Brazil had 65% of quality sequences, a percentage higher than the world average and other countries studied (India with 38%, Italy with 39%, Germany with 41% and

England with 56% of the sequences with quality) . The rSWeeP projection process ran in about 0.08s per sequence. For the 1 million sequences worldwide it took 22h using 21Gb of RAM, as shown in Table **1** (paper).

2.1 Landscape in Brazil

Based on the established consensus criteria (Figure S2), we identified 15 clusters representing the epidemic in Brazil from 25 February 2020 to the end of May 2021. All 15 clusters obtained around 80% consensus of each point in its respective cluster, for all sequences. More than 15 clusters do not provide considerable increase (less than 5%) in the consensus value of the CDF curve.

According to the PANGO v3.0.5 nomenclature of 2021-06-04, 11 main lineages were identified in Brazil: P.1(3572 – 40.9%), P.4(1274 – 14.6%), P.2(1132 – 13.0%), B.1.1.33(909 – 10.4%), B.1.1.28(864 – 9.9%), B.1.1.7(248 – 2.8%), B.1.1(186 – 2.1%), P.1.2(153 – 1.7%), N.9(81 – 0.9%), B.1(65 – 0.7%), B.1.195(54 – 0.6%), other (178 – 2.0%).

As mentioned in the main text, the clustering results match with the division of the observed groups by t-SNE, PCA, distance matrix and PANGO nomenclature (Figures **1** and **2**), except for particular cases (Table S1).

2.2 Mutation profile of the groups

We found the most common mutations in Brazil, present in almost all 8720 samples, and listed them in Table S5. We determined the characteristic mutations for the TP1 group in Table S6, and for the other clusters in Table S7. Mutations are visually presented in the heatmap of Figure **3**.

The spike mutation D614G is a globally dominant allele González-Candelas et al. (2021), present in 98.9% of the samples already collected in Brazil. It is already established that such mutation generates an increase in infectivity due to a large conformational change of the spike protein and due a higher viral load in relation to the ancestral Yurkovetskiy et al. (2020); Korber et al. (2020).

As previously verified, mutations in the Spike protein have a strong impact on the definition of lineages and variants of SARS-CoV-2, being responsible for much of the division between the clusters. Mutations and indels that occur in the Spike protein change the protein's conformation, affecting its stability and functionality, as well as its ability to infect the host Berger and Schaffitzel (2020).

Non-characteristic samples from each group were analyzed in greater detail. The Table S1 presents samples that have probable lineage annotation error by PANGO (v3.0.5) or possible unidentified recombination events, as already reported Varabyou et al. (2021).

2.2.1 cluster 11 - variant B.1.1.7

B.1.1.7 of cluster 11 has 3 deletions and 1 mutation shared with TP1 group (ORF1a:G3676–, S3675–, F3677– and S:N501Y), 4 mutations that are consensus in Brazil (ORF1b:P314L, S:D614G, N:R203K and N:G204R), and 13 mutations and 3 unique deletions from the group (ORF8:Y73C, N:S235F, S:A570D, S:S982A, S:D1118H, S:T716I , ORF1a:T1001I, N:D3L, ORF8:R52I, ORF1a:A1708D, ORF1a:I2230T, ORF8:Q27*, S:P681H, S:H69–, S:V70–, S:Y144–). These mutations correspond to those found in the literature Davies et al. (2021).

2.2.2 cluster 13 - variant P.4

Cluster 13 has 5 mutations that are consensus in Brazil (ORF1b:P314L, S:D614G, N:R203K, N:G204R, S:V1176F) and has 15 unique mutations (ORF1a:L3915F, ORF1b:Y822C, ORF3a:V50I, ORF1a:A516T, ORF1a:L3201P, ORF1a:A3143V, S:L452R, N:A220V, S:I720V, S:Q173K, ORF1a:Q3729K, S:N164K, S:S704L, S:G142V, ORF1a:P971L). As detailed in Table S7, the S:L452R mutation makes the virus 20% more transmissible, increases its infectivity (*in vitro*), and reduces antibody neutralization Deng et al. (2021), giving low vaccine efficiency VanInsberghe et al. (2021). This mutation occurs in more than 103'000 samples worldwide, but it is rare in Brazil.

The N:A220V mutation alters the conformation of the protein and potentially causes changes in pathogenicity and/or response to vaccination, affecting the activity of the protein's binding site. It is a growing mutation, with 163'000 occurrences in the world, one of the top 10 most frequent in the world, but rare in Brazil Fang et al. (2021); Vilar and Isom (2021). Additionally, the S:Q173K mutation has the potential to increase the fitness of the Pater et al. (2021) virus, but there is still no concrete evidence.

2.2.3 Early group (T0) - clusters 1,2,4,5,6 e 8

The group does not have consensus mutations characteristic of the group, as they are not a coherent group like the TP1 group. Each cluster represents an individual lineage, or a group of less frequent lineages in Brazil. The mutations shared by this group are the Brazilian consensus mutations (e.g. ORF1b:P314L, S:D614G), as detailed in Table S5.

2.2.4 Groups related to variant P.1 (TP1) - clusters 3,7,9,10,12,14 e 15

The TP1 group are divided into 7 clusters: the oldest are cluster 3 (composed of sequences from P.1) and cluster 7 (composed of P.4), the only clusters with samples in 2020. In total, there are two clusters of variant P.1 (clusters 3 and 14), two of variant P.4 (clusters 7 and 15), two containing variants P.1 and P.4 (clusters 9 and 10), and one composed of the P.1.2 variant (cluster 12).

These clusters have a particular set of non-synonymous mutations shared by the entire group, in addition to the deletions of nucleotides 11288-11296, and the insertion 28263:AACA (Table S6). Most of these consensus mutations are located in the spike protein and confer an adaptive advantage: mutations H655Y and L18F promote immune response escape Colson et al. (2021); Grabowski et al. (2021); mutations E484K and N501Y strengthen the binding with hACE2, providing high transmissibility and low vaccine efficiency VanInsberghe et al. (2021); the association K417T, E484K and N501Y promotes high affinity with ACE2 (40-50% greater than wild type) and resistance to antibodies Gan et al. (2021). It is believed that some mutations such as E484K and N501Y in the spike protein were positively selected for conferring such adaptive advantages Franceschi et al. (2021).

2.3 Temporal and spatial distribution of strains detailing

About the distribution of variants by state (Figure S8), in more detail, we observed:

- No conclusions can be drawn from the distribution of variants in the following states: Acre (Ac), Ceará (CE), Distrito Federal (DF), Mato Grosso (MT), Piauí (PI), Rondônia (RO), Roraima (RR), Tocantins (TO).
- The states of Mato Grosso do Sul (MS), Pará (PA), Rio de Janeiro (RJ), Paraná (PR), Rio Grande do Norte (RN) and Rio Grande do Sul (RS) behaved as the Brazilian average described .

- The states of Alagoas (AL), Amapá (AP), Maranhão (MA), Minas Gerais (MG) and Santa Catarina (SC) do not provide enough data to infer about the variants in the state in 2020. In late 2020 (November and December) and early 2021 there was a predominance of variant P.2 which was gradually replaced by P.1. Additionally, in MA there was a predominance of N.9 in December 2020; and in MG there was a growth of the P.4 variant from March 2021 onwards..
- Amazonas (AM): until april/2020 the B.1.195 variant predominated. Between May and August/2020 there were variants B.1.195, B.1.1.28 and B.1.1.33 representatively. The B.1.1.378 variant was present from the beginning in a smaller amount until the end of 2020. From December 2020 the P.1 variant became predominant in the state.
- Bahia (BA): In November the N.9 became significant but its frequency dropped until February/2021, when the P.2 variant became representative.
- Espírito Santo (ES): as of February/2021, variants B.1.1.7 and P.1 become predominant.
- Goiás (GO) and São Paulo (SP): behave as the Brazilian average described, additionally, from February 2021, P.4 and P.1.2 are growing.
- Paraíba (PB): The year 2020 is mainly composed of variants B.1.1 and B.1.1.33.
- Pernambuco (PE): the beginning of 2020, between April and July 2020, the most representative variant was B.1.1.
- Sergipe (SE): in the first months of 2020 (until April 2020) there was a varied distribution of variants (B.1, B.1.1, B.1.1.28, B.1.1.33 and others). In early 2021 there was also a diversity of variants which was quickly replaced by P.1 from February 2021 onwards. In the other months there was no sampling.

2.4 Diversity of SARS-CoV-2 proteomes in Brazil

São Paulo State (SP) presents the higher diversity (richness value), followed by RJ and RS with 10,221, 4,073, and 3,011 estimated distinct proteomes, respectively. The lower richness is in AC, with 50, followed by DF with 99 estimated proteomes (Table S10).

Regarding coverage, the states of SP, RO, and AM had the highest coverage (30.3%, 28.9%, and 26.9% respectively), while MA, AP, AL, MG, and SC had the lowest coverage (4.4%, 7.2%, 7.2%, 7.8%, and 8.9% respectively). Unfortunately, we could not analyze the states of RR, PI, RN, and MS due to low sampling.

The TP1 group presented almost half of the richness in Brazil, with 13,194 estimated distinct proteomes representing about 47.6% of the total local diversity in Brazil (Table S11).The TP1 also presented a high coverage (27.10% on average), which may be corresponding to the increase in sequencing in 2021; clusters 11 and 13 also had high coverages of 33.67% and 25.56%, respectively. T0 had lower coverages with 19.20% on average due to less sequencing at the beginning of 2020. We observed an increase in sampling between 2020 to 2021 and an increase in proteome quality (proteome complete and without misreadings). In 2020, 4,402 samples were sequenced, of which 52% (2,293 sequences) with quality, while in 2021, 8,993, of which 71% have good quality (6,427 instances).

Brazilian coverage (23.3%) is low compared to Italy, Germany, and England, but it is superior to India. However, the estimated richness of Brazil (26,370) is lower than the other countries in this study except for Italy (Table **3**,Figure S10).

3 SUPPLEMENTARY FIGURES AND TABLES

Figure S1: Pipeline. The method starts by concatenating viral proteome sequences (with border delimiters) and filtering out the quality sequences (removing incomplete proteomes and sequences with misreading). Then, the sequences are projected in numerical vectors by the rSWeeP tool resulting in a numerical matrix that is used for the following analyses: phylogenetic tree construction, clustering, PCA, t-SNE, and other analyses.

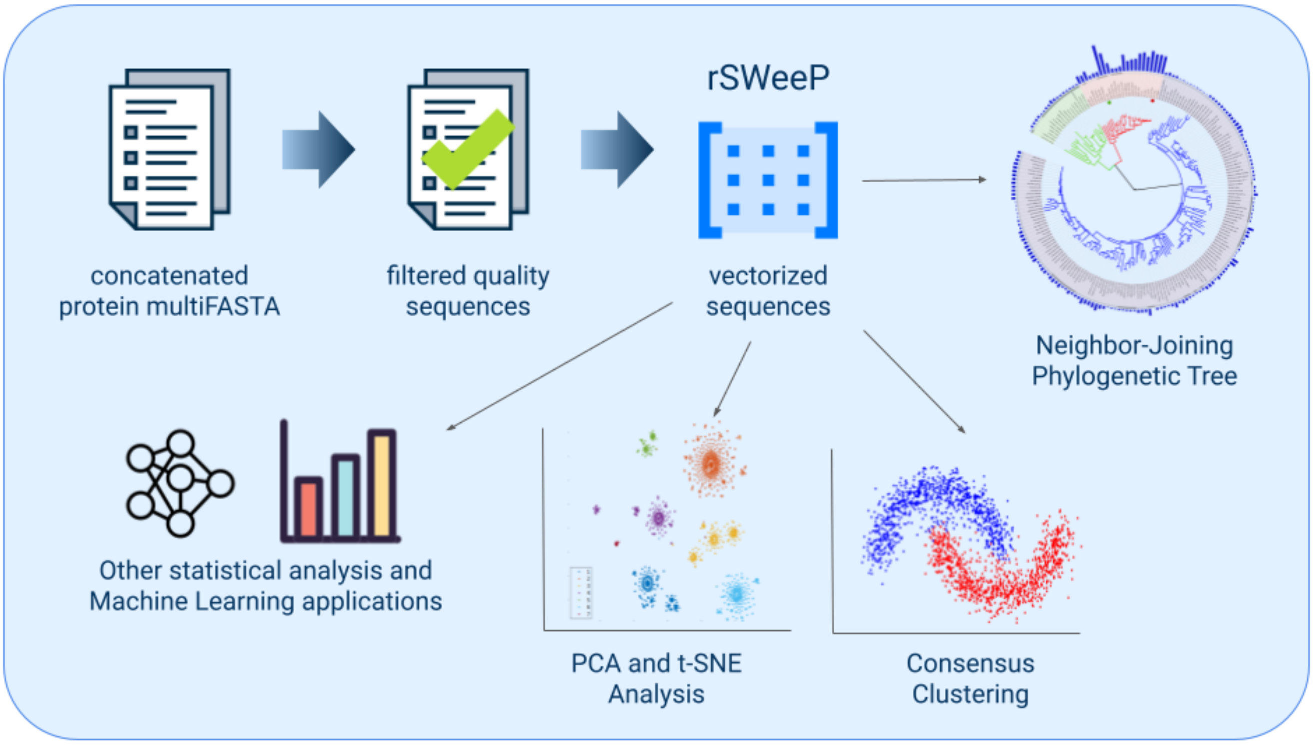

Figure S2: Consensus Cumulative Distribution Function. It is observed that above 15 clusters there is no appreciable increase in the value of the consensus between the clusters (less than 5%). With 15 clusters, a value of about 80% of consensus is obtained for all samples in their respective clusters.

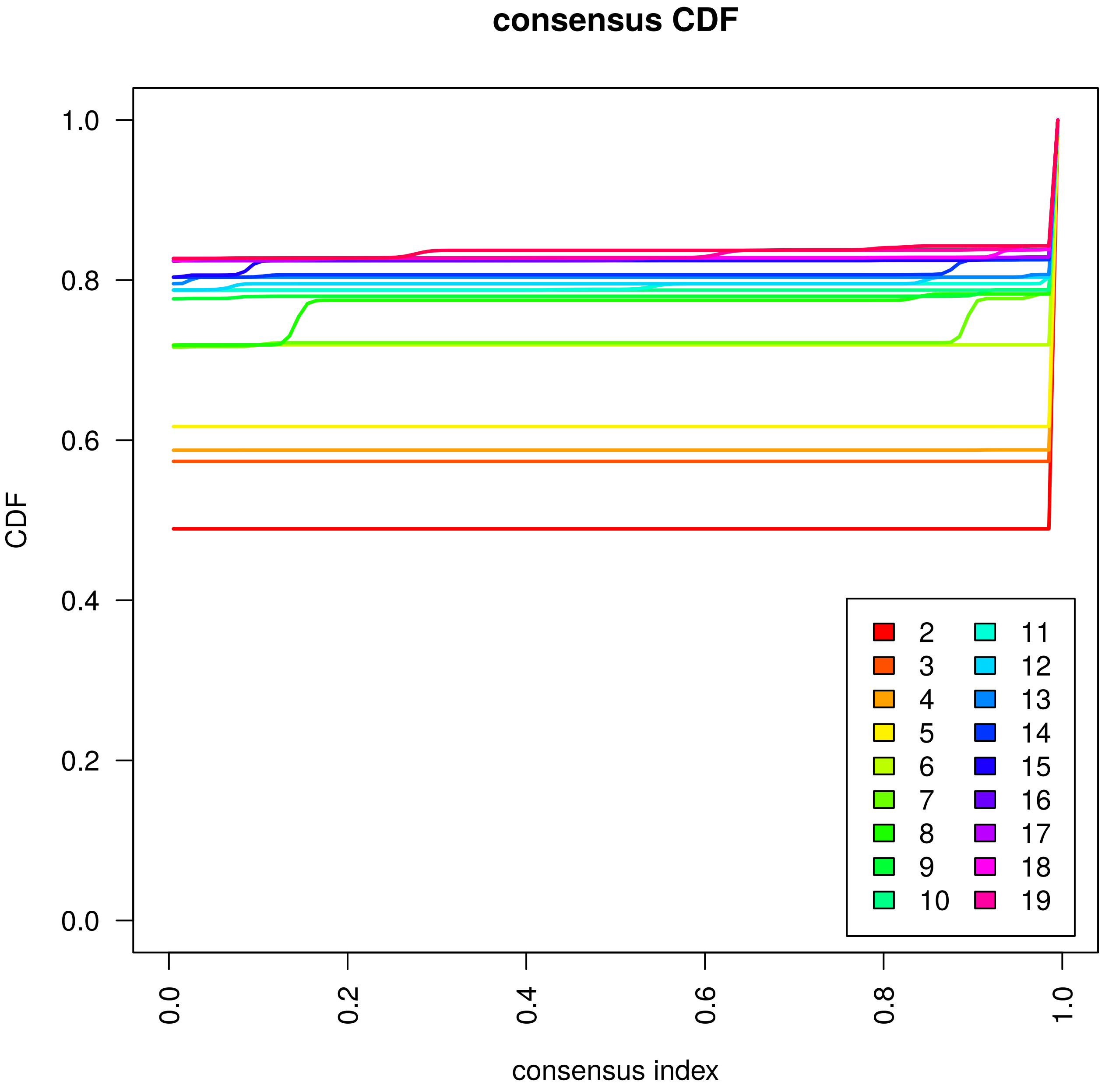

Figure S3: Clustering of vectorized spike proteins. We obtained 8 well-defined clusters, however there is a greater degree of mixing of the strains within the clusters due to the lower resolution provided by the protein in relation to the complete proteome. **a)** t-SNE and **b)** PCA of spike sequences. Divides the samples similarly to the complete proteome: TP1 group on the left, B.1.1.7 below on the right, and the remaining sequences on the right.

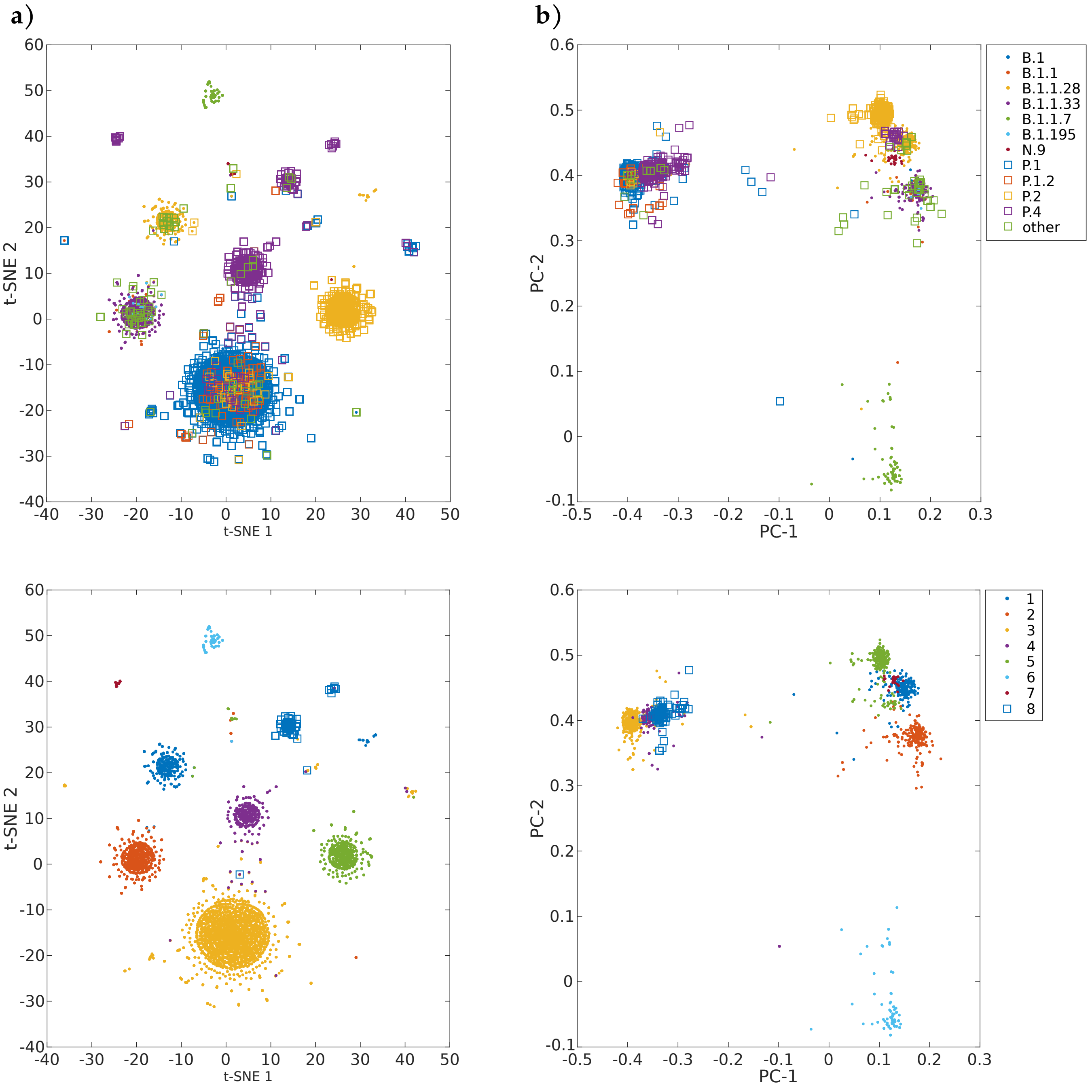

Figure S4: Detection of the emerging variant P.1-SP. A subset of variant P.1 (data from GISAID release 409 using PANGO v.2.3.8) was later identified as new variant P.4 (GISAID release 609 using PANGO v.3.0.5). **a)** t-SNE with data from GISAID release 409, until 2021-03-19. Clusters correspond to: (1) P.1; (2) B.1.1.33; (3) B.1.1/B.1.1.28; (4)B.1.1.28; (5)B.1/B.1.195; (6) P.2; (7) B.1.1.7 (PANGO nomenclature v2.3.8 of 2021-04-02). Circled in red is P.1-SP group, emerging variant group P.4, according to PANGO v.3.0.5. **b)** t-SNE with data from GISAID release 609, until 2021-05-29. Highlighted in red triangles the emerging variant P.4, as circled in **a)**.

a)

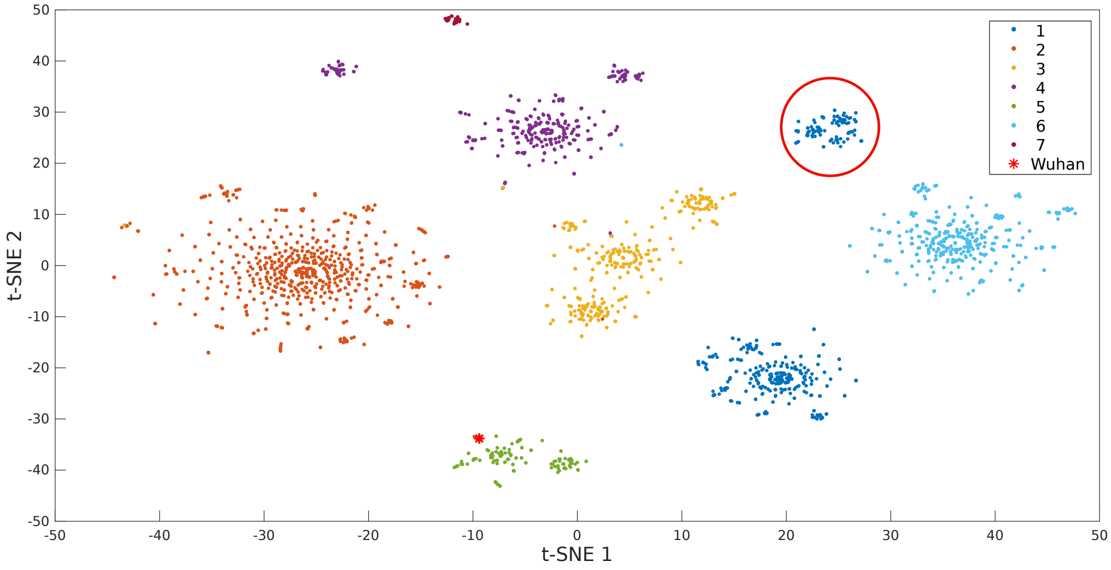

b)

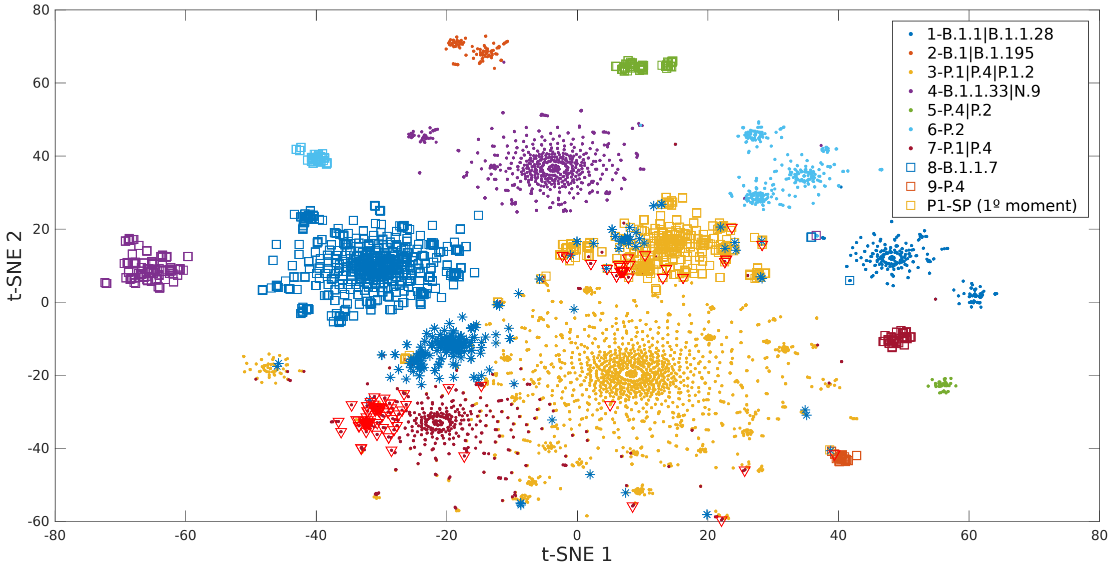

Figure S5: Heatmap of mutations by lineage (relative to PANGO v3.0.5). Mutations present in 75% samples from one or more lineages are listed. The value 1 (red) represents the presence of the mutation in 100% of the lineage samples, and the value 0 (blue) indicates the absence. Values are normalized by lineage.

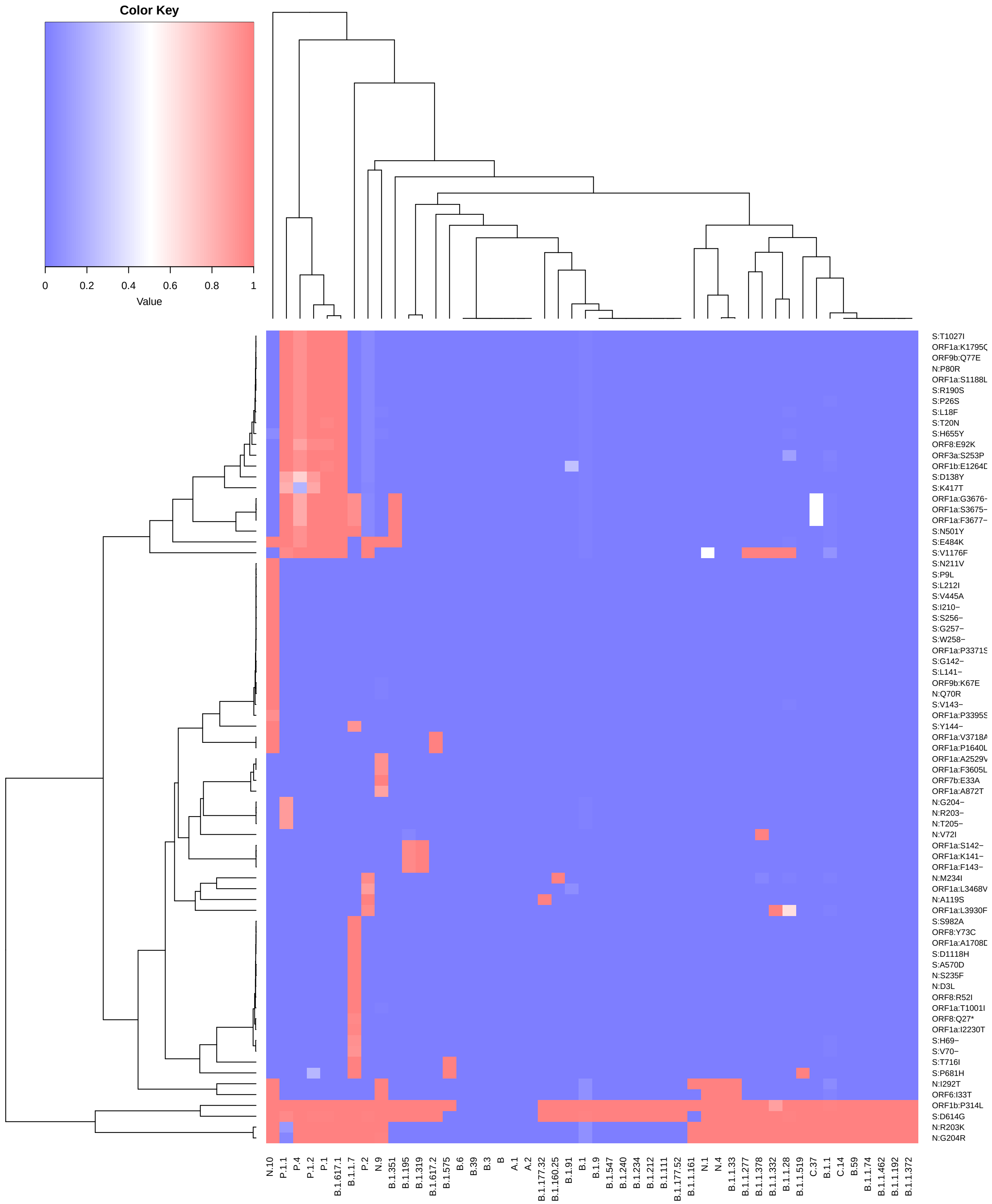

Figure S6: One of the possible phylogenetic trees for SARS-CoV-2 in Brazil, generated by the Neighbor-Joining method from the **a)** proteomes and **b)** genomes. Total of 8720 samples, data from release 609 of GISAID, using PANGO nomenclature v3.0.5. Complete trees in newick format available in the **Suplementaries 5** and **6**

a) Proteome **b)** Genome

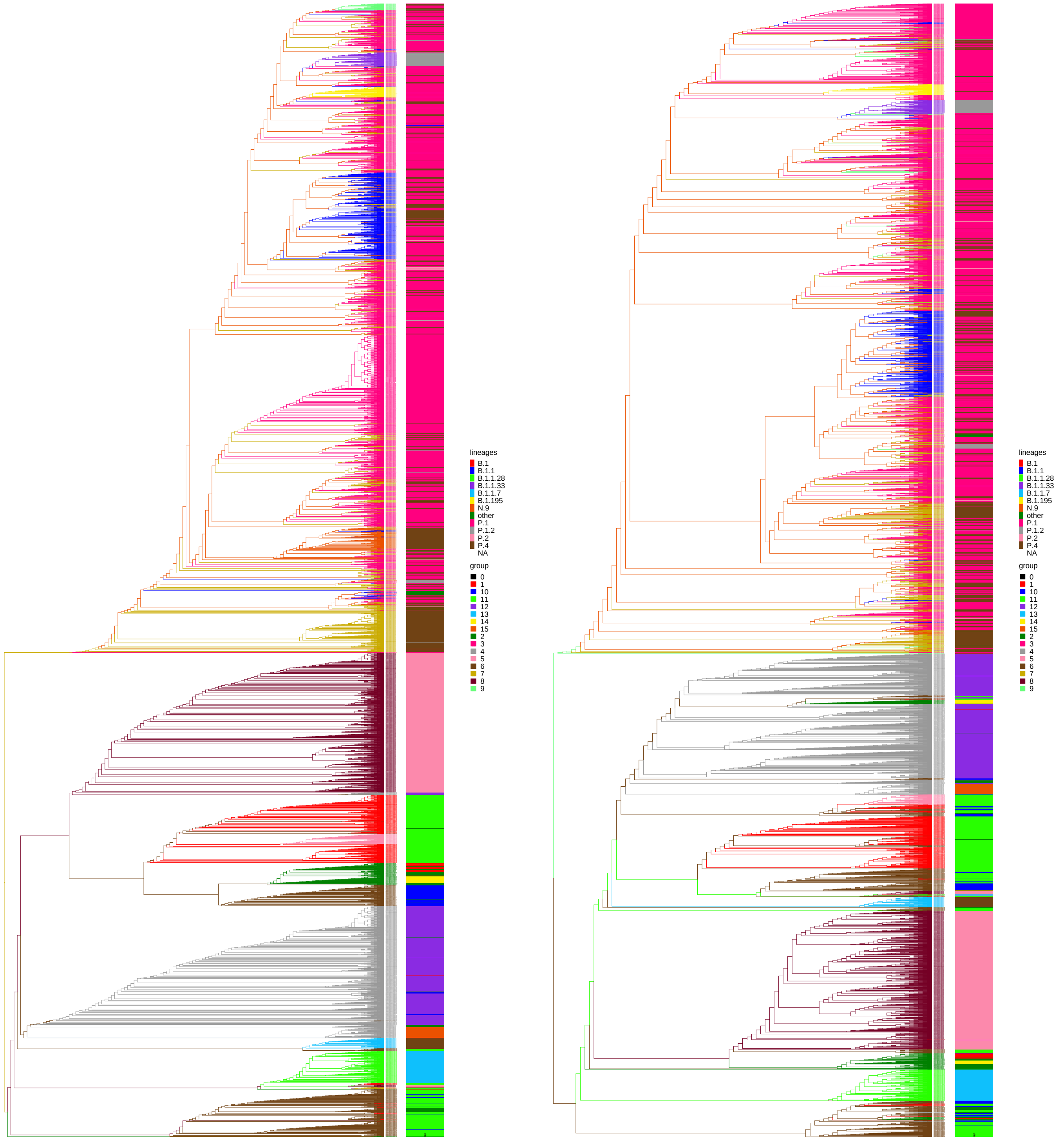

Figure S7: Phylogenetic tree of the origin of P.1 lineage in 2020. Phylogeny composed of the samples closest to P.1 samples from 2020 selected by distance matrix. In blue are the 50 Brazilian and 6 Peruvian P.1 samples from 2020, in green the two ancestral samples of the P.1 variant. In pink the samples indicated by Naveca *et al* Naveca et al. (2021) as ancestors of P.1. In red the Wuhan reference sample. Complete tree in newick format available in the **Suplementary 7**.

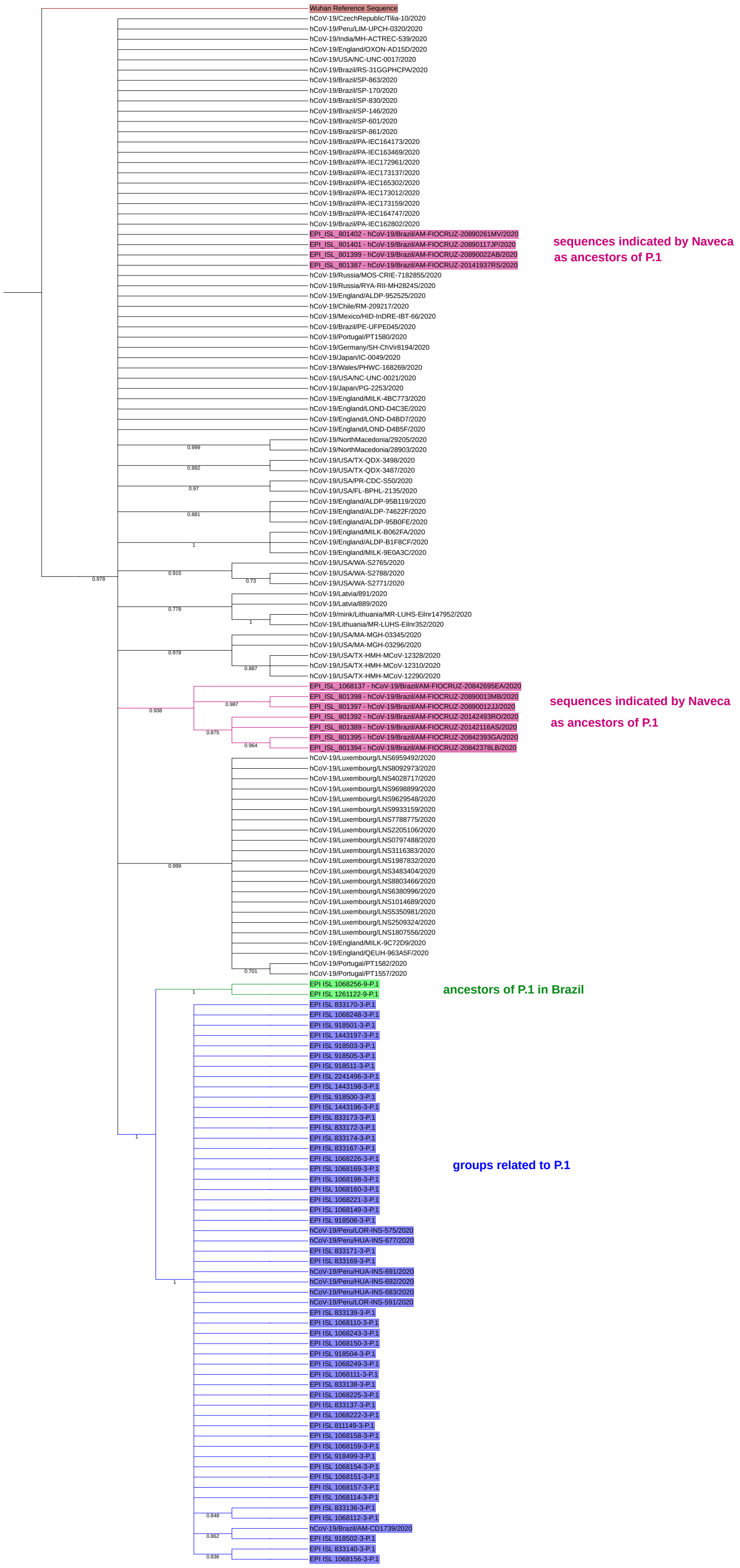

Figure S8: Proportional stacked bar chart representing proportional abundance of variants by month for each Brazilian state. Month 2 corresponds to February 2020, and 13 to January 2021.

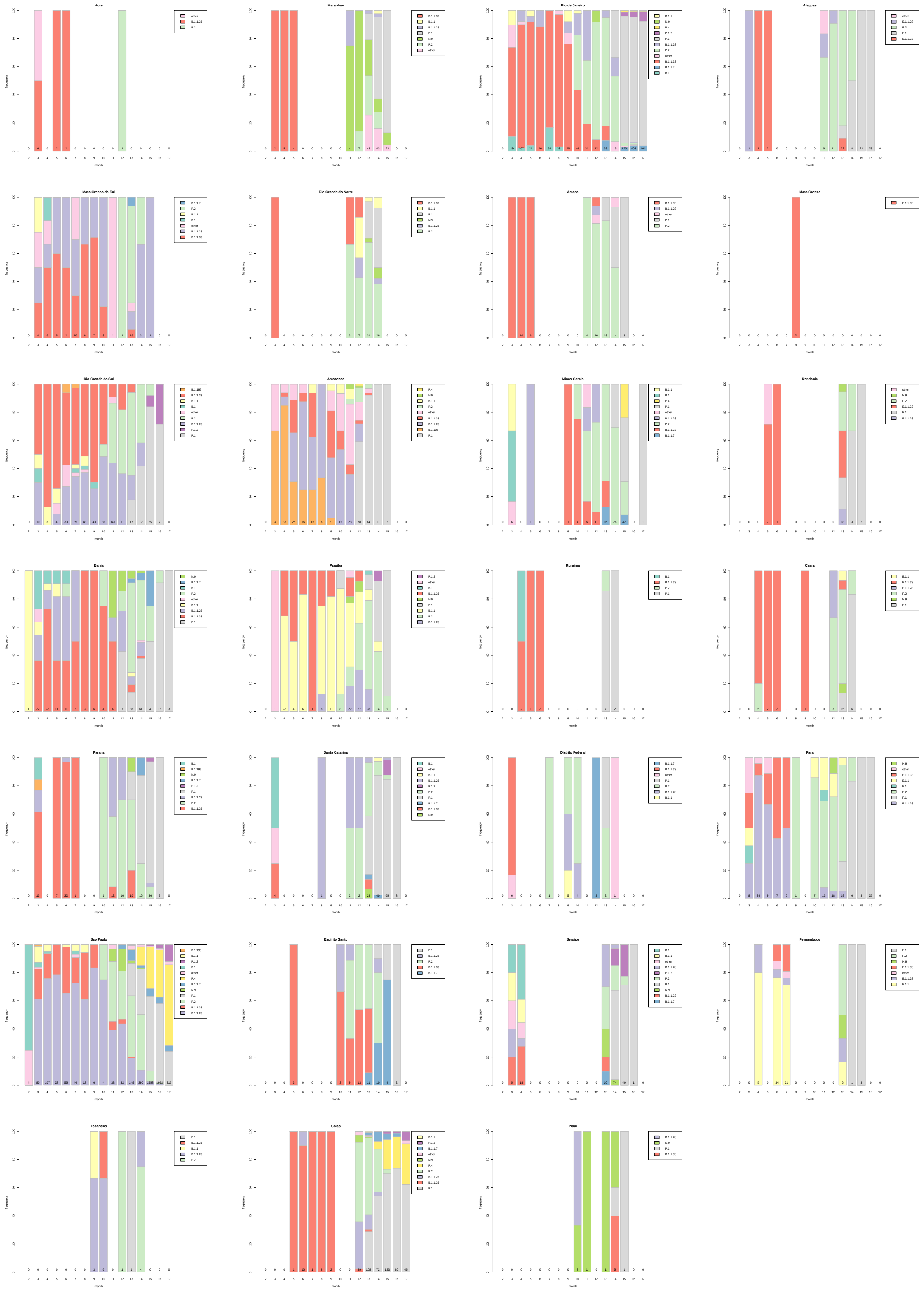

Figure S9: t-SNE in time. Time scale in weeks, since the first week of 2020. The TP1 group currently predominates and the older clusters are almost extinct.

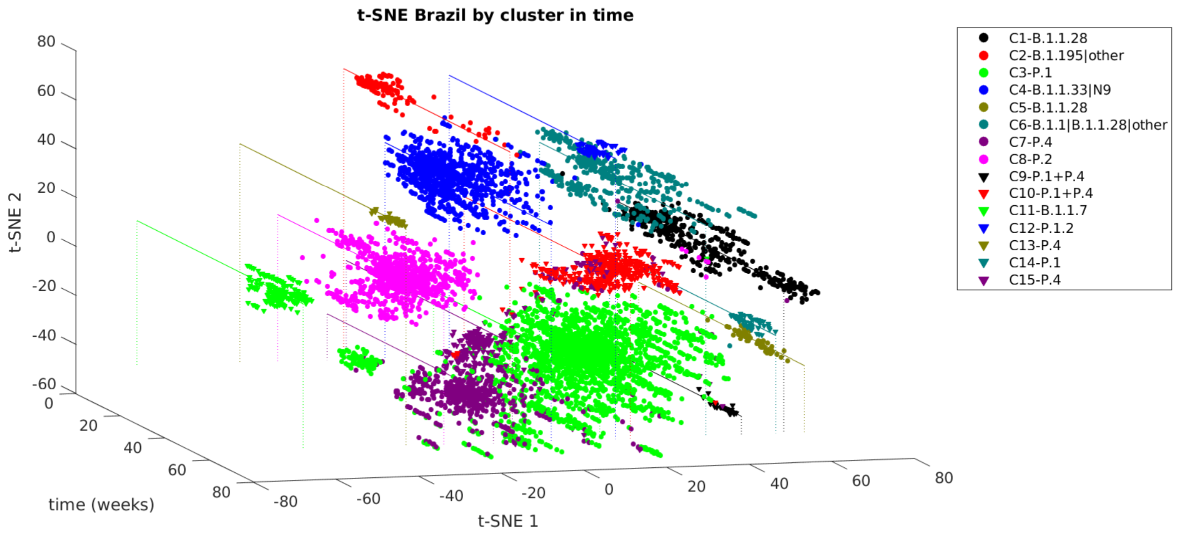

Figure S10: Richness by country until the end of May 2021. Despite having a much higher number of cases than European countries, Brazil and India have a much smaller sample.

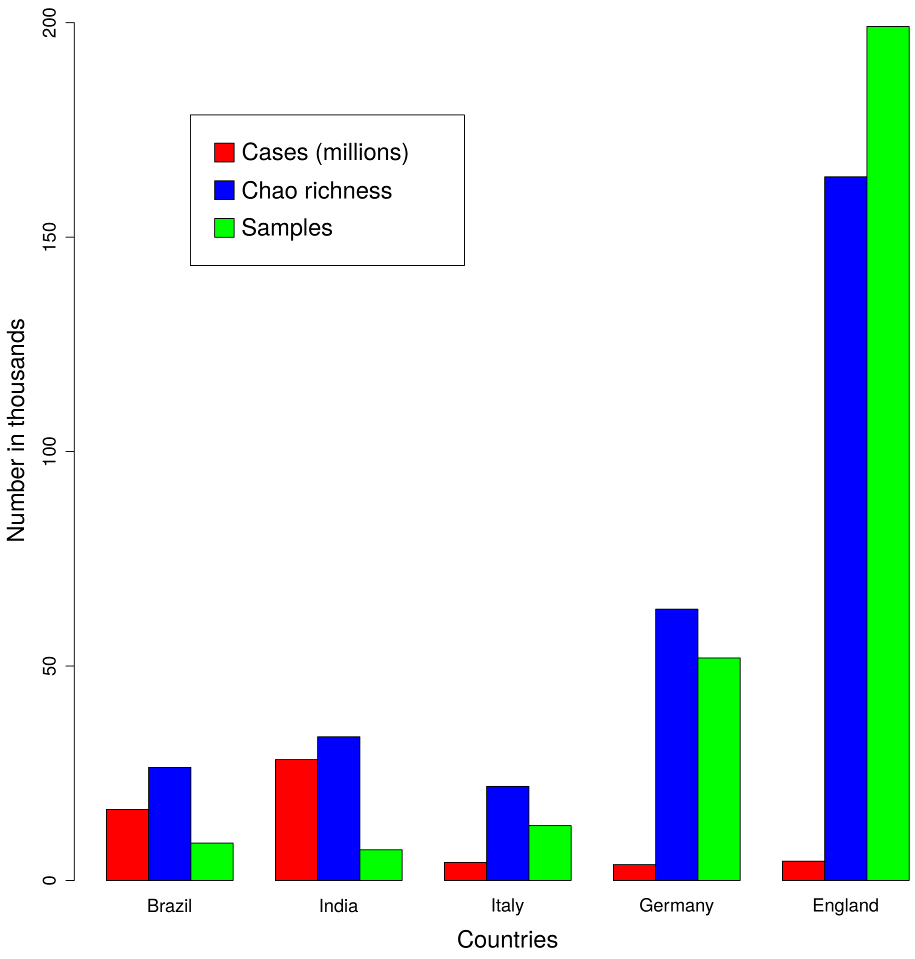

Figure S11: Proportional stacked bar chart representing proportional abundance of lineage by months for the year 2021. We use the PANGO nomenclature version 3.1.11 (2021-08-09). Histogram normalized by months, from January to August 2021. The numbers inside the bars indicate the number of sequencing performed each month.

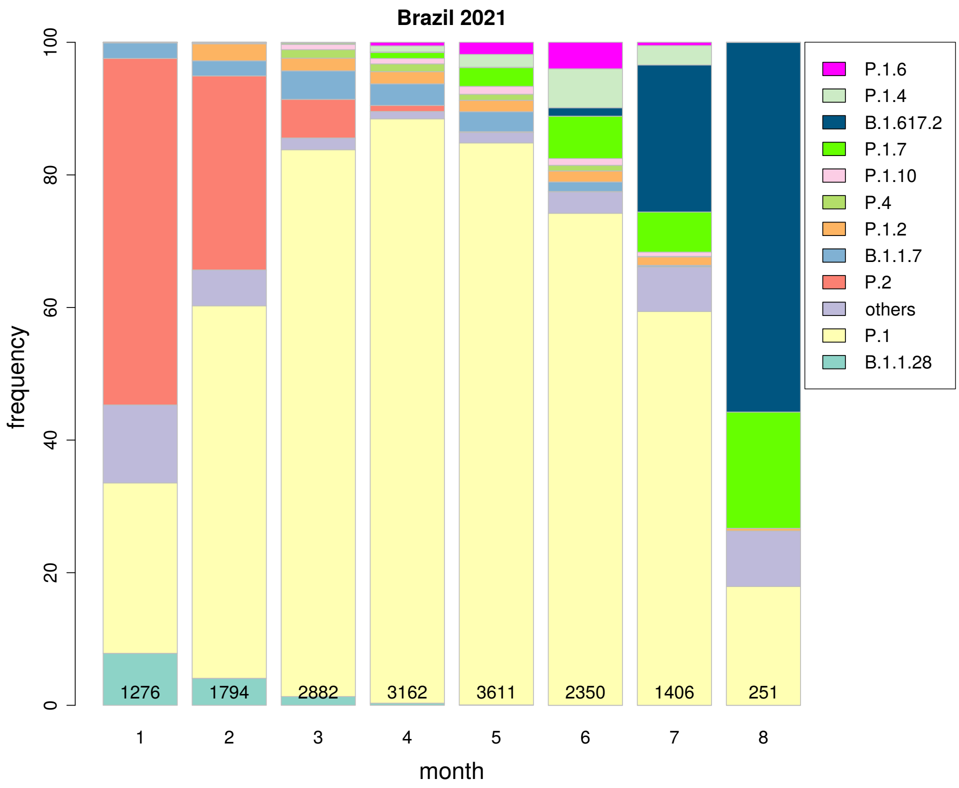

Figure S12: Coverage and Richness by state by the Chao metric. The coverage of the states of RR, RN, PI and MS could not be calculated due to low sampling.

a) Coverage **b)** Richness

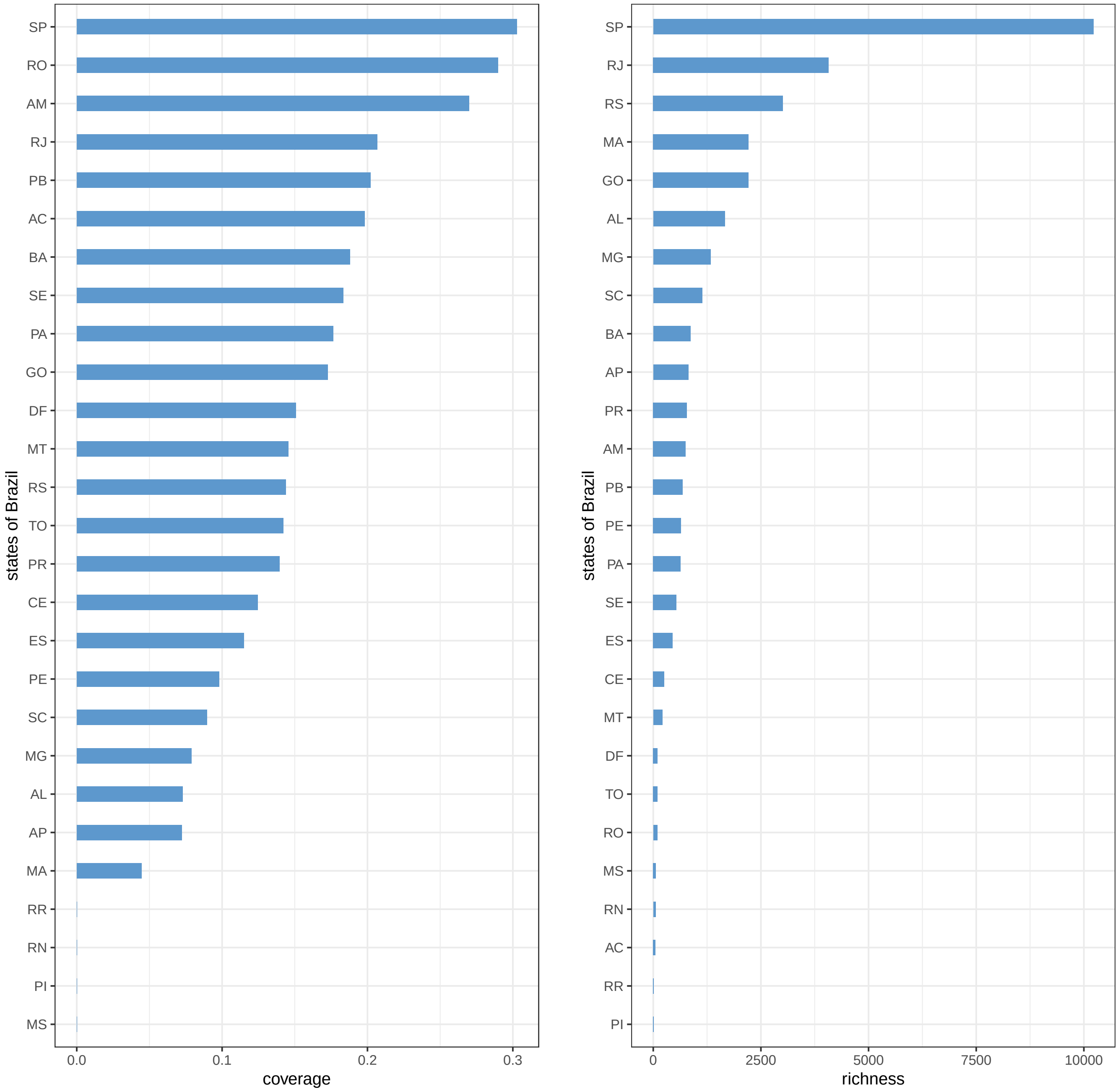

Figure S13: A viral variant is contained in an environment (population of a country) and might be adapted to that environment or not. Considering that the population of a country constitutes a certain fitness function (for a given human population, certain mutations of the virus are advantageous while others are not) in a mutational space of the virus (among the possibilities of mutation of a virus, where each point on the curve represents a certain combination of alleles), a certain viral variant can have a fitness value that represents its degree of adaptation to the population in which it is found. Over time a virus can undergo mutations that promote higher or lower adaptation/fitness to the human population in which it is found. In the fitness curve there are regions of maximum that correspond to regions of adaptation. Random mutations undergo evolutionary pressure tending to generate viral populations that move down the gradient toward the local maximum. Each mutation allows the virus to walk in mutation space in small steps, moving toward a region of maximum.
Using this analogy, we hypothesize that **(a)** a virus coming from a foreign country (adapted or not to its environment of origin), **(b)** enters a new environment having a bad initial fitness, but potential to adapt, i.e., possibility to reach a maximum. **(c)** Over time the variant acquires advantageous mutations, allowing it to reach a local maximum in the new country, adapting, and dispersing. As an example, **(d)** the P1 variant entered Brazil in mid-October 2020, initially with few cases, **(e)** and as it gained new mutations (7 mutations in Brazil), it became adapted to the new environment, reaching a minimal region. **(f)** Thus it dispersed and became prevalent and representing the largest number of cases in the country (2nd epidemic wave).

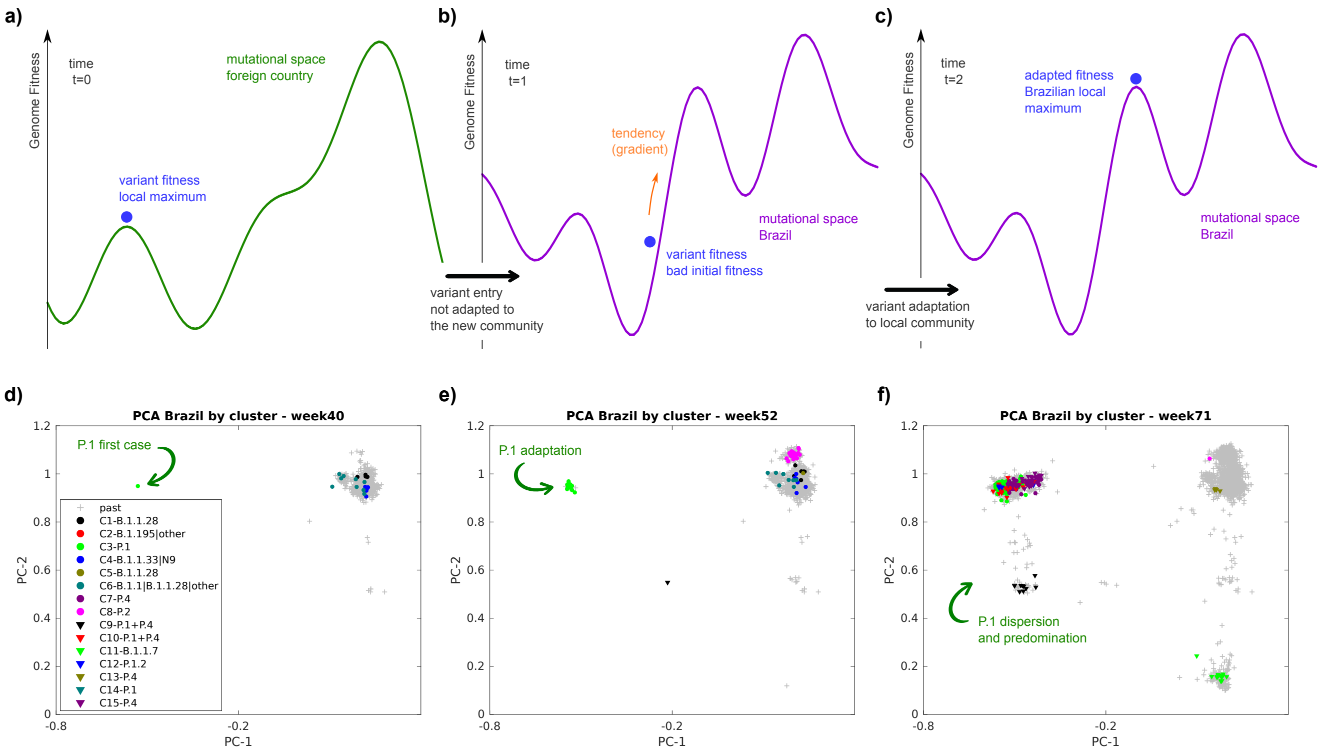

Table S1. Probable annotation errors of variants. Possible recombination events have not been discarded for the listed samples.

EPI code	cluster	PANGO variant	rectification	justification
EPI_ISL_2188014, EPI_ISL_2274105, EPI_ISL_1628358	3	B.1.617.1	P.1	samples have characteristic mutations of the P.1 variant of the cluster 3, and does not have the characteristics of lineage B.1.617.1. Is inserted next to the branch of P.1 in the genomic and proteomic trees.
EPI_ISL_2344990	3	B.1	P.1.1	the sample is inserted in the tree of P.1 in the branch of P.1.1 and has mutations characteristic of P.1.1.
EPI_ISL_2209844	7	P.1	P.4	the sample is inserted in the tree of P.4 and has mutations characteristic of P.4 of the cluster 7.
EPI_ISL_1445131, EPI_ISL_1795191, EPI_ISL_1966175, EPI_ISL_1966818, EPI_ISL_1966819, EPI_ISL_1966821	7	P.2	P.4	the sample is inserted in the branch of P.4 (100 % bootstrap in the TP1 group) and has mutations characteristic of P.4 of cluster 7. But they have an N:A119S mutation characteristic of P.2.
EPI_ISL_2308409, EPI_ISL_2308456, EPI_ISL_2308471, EPI_ISL_2308473	12	P.1	P.1.2	have the non-synonymous mutations characteristic of P.1.2 (ORF3a:D155Y,ORF1a:D762G and ORF1a:T1820I) and are inserted within the branch of P.1.2 (with bootstrap of 100%)

Table S2: Distribution of PANGO lineages (v 3.0.5) in each cluster.

clusters	pangolin lineage	frequency
1	B.1.1	2
1	B.1.1.28	467
1	B.1.1.332	8
2	A.1	1
2	A.2	3
2	B	3
2	B.1	59
2	B.1.111	5
2	B.1.160.25	1
2	B.1.177.32	1
2	B.1.177.52	1
2	B.1.195	54
2	B.1.212	15
2	B.1.234	2
2	B.1.240	1
2	B.1.319	1
2	B.1.351	5
2	B.1.547	1
2	B.1.575	1
2	B.1.617.2	1
2	B.1.9	1
2	B.1.91	15
2	B.3	2
2	B.39	5
2	B.6	1
3	B.1	1
3	B.1.1	1
3	B.1.617.1	2
3	P.1	2966
3	P.1.1	18
3	P.1.2	26
3	P.2	27
3	P.4	21
4	B.1	4
4	B.1.1	11
4	B.1.1.161	7
4	B.1.1.33	909
4	N.1	2
4	N.10	19
4	N.4	4

4	N.9	81
5	B.1.1.28	77
6	B.1	1
6	B.1.1	171
6	B.1.1.192	1
6	B.1.1.277	3
6	B.1.1.28	317
6	B.1.1.372	1
6	B.1.1.378	29
6	B.1.1.462	1
6	B.1.1.519	2
6	B.1.1.74	1
6	B.59	1
6	C.14	1
6	C.37	2
7	P.1	4
7	P.1.1	4
7	P.1.2	8
7	P.2	6
7	P.4	732
8	B.1.1.28	1
8	P.2	1089
9	P.1	31
9	P.1.2	4
9	P.4	15
10	P.1	491
10	P.1.1	2
10	P.1.2	3
10	P.2	14
10	P.4	155
11	B.1.1	1
11	B.1.1.7	248
11	P.1	1
12	P.1	4
12	P.1.2	100
13	P.4	79
14	B.1.1.28	2
14	P.1	74
14	P.4	5
15	P.1	1

15	P.1.1	4
15	P.1.2	12
15	P.4	267

Table S3. Division of lineages into clusters using only the vectorized spike protein. The predominant lineages in each cluster are listed. The complete listing of observed lineages by cluster is available in Table S4.

cluster	predominant lineage	number of samples	first case
groups related to the P.1 variant – TP1		4999	2020-10-01
3	P.1 + P.1.2	3767	2020-10-01
4	P.4	902	2020-12-21
8	P.4	330	2021-02-19
early group – T0		3391	2020-02-25
1	B.1.1.28	895	2020-03-05
2	B.1.1.33 + B.1.1 + B.1.195	1307	2020-02-25
5	P.2+N.9	1189	2020-04-13
others		330	2020-12-21
6	B.1.1.7	251	2020-12-21
7	P.4	79	2021-02-17

Table S4: Distribution of PANGO lineages (v 3.0.5) in each cluster, using only spike proteins.

cluster	pangolin lineage	frequency
1	B.1.1	15
1	B.1.1.277	3
1	B.1.1.28	837
1	B.1.1.33	1
1	B.1.1.332	8
1	B.1.1.378	29
1	N.1	1
1	P.1	1
2	A.1	1
2	A.2	3
2	B	3
2	B.1	63
2	B.1.1	169
2	B.1.1.161	7
2	B.1.1.192	1
2	B.1.1.33	908
2	B.1.1.372	1
2	B.1.1.462	1
2	B.1.1.519	2
2	B.1.1.74	1
2	B.1.111	5
2	B.1.160.25	1
2	B.1.177.32	1
2	B.1.177.52	1
2	B.1.195	54
2	B.1.212	15
2	B.1.234	2
2	B.1.240	1
2	B.1.319	1
2	B.1.351	5
2	B.1.547	1
2	B.1.575	1
2	B.1.617.2	1
2	B.1.9	1
2	B.1.91	15
2	B.3	2
2	B.39	5
2	B.59	1
2	B.6	1
2	C.14	1
2	C.37	2

2	N.1	1
2	N.10	19
2	N.4	4
2	N.9	6
3	B.1	1
3	B.1.1	1
3	B.1.617.1	2
3	P.1	3563
3	P.1.1	19
3	P.1.2	116
3	P.2	41
3	P.4	24
4	P.1	7
4	P.1.1	5
4	P.1.2	21
4	P.2	6
4	P.4	863
5	B.1.1.28	24
5	N.9	75
5	P.2	1089
5	P.4	1
6	B.1	1
6	B.1.1	1
6	B.1.1.28	1
6	B.1.1.7	248
7	P.4	79
8	B.1.1.28	2
8	P.1	1
8	P.1.1	4
8	P.1.2	16
8	P.4	307

Table S5. Consensus mutations in the Brazilian context. The frequency is relative to the 8720 quality sequences analyzed.

mutation	frequence (seq.)	remark
ORF1b:P314L	8660	present in all clusters
S:D614G	8629	present in all clusters – provides high transmissibility, low vaccine efficiency (VanInsberghe et al., 2021) and increases the infectivity rate due to a large conformational change of the protein (Timmers et al., 2021; Yurkovetskiy et al., 2020). Presented high levels of viral load compared to the original Wuhan strain (Korber et al., 2020)
N:R203K	8512	present in all clusters, except in cluster 2 – suggested as a critical region for virus maturation and assembly; highly observed in the world (Timmers et al., 2021)
N:G204R	8501	present in all clusters, except in cluster 2 – suggested as a critical region for virus maturation and assembly; highly observed in the world (Timmers et al., 2021)
S:V1176F	7080	present in all clusters significantly, except in clusters 2, 4 e 11

Table S6. Consensus mutations of TP1 group in the Brazilian context. The frequency is relative to the 5000 quality sequences analyzed belonging to the group, for general mutations. For specific clusters, the frequency is relative to their own number of sequences.

mutation	frequence (seq.)	remark
S:E484K	4992	present in all clusters of the TP1 group – the mutation strengthens the link with hACE2, providing high transmissibility and low vaccine efficiency (VanInsberghe et al., 2021). The association S:K417T, S:E484K and S:N501Y promotes high affinity with ACE2 (40-50% greater than the wild type) and resistance to antibodies (Gan et al., 2021)
S:N501Y	4990	present in all clusters of the TP1 group – the mutation strengthens the link with hACE2, providing high transmissibility and low vaccine efficiency (VanInsberghe et al., 2021). The association S:K417T, S:E484K and S:N501Y promotes high affinity with ACE2 (40-50% greater than the wild type) and resistance to antibodies (Gan et al., 2021)
S:H655Y	4988	present in all clusters of the TP1 group; promotes escape from the immune system (Colson et al., 2021)
S:L18F	4991	present in all clusters of the TP1 group; compromises the binding of neutralizing antibodies and the immune response (Grabowski et al., 2021)
S:T1027I	4984	present in all clusters of the TP1 group
S:P26S	4987	present in all clusters of the TP1 group
S:R190S	4964	present in all clusters of the TP1 group
N:P80R	4977	present in all clusters of the TP1 group
ORF1a:K1795Q	4992	present in all clusters of the TP1 group
ORF1a:S1188L	4960	present in all clusters of the TP1 group
ORF3a:S253P	4973	present in all clusters of the TP1 group
ORF9b:Q77E	4977	present in all clusters of the TP1 group
S:K417T	4035	absent in the cluster 7; the association S:K417T, S:E484K and S:N501Y promotes high affinity with ACE2 (40-50% greater than the wild type) and resistance to antibodies (Gan et al., 2021)
S:D138Y	4656	absent in the cluster 15
ORF8:E92K	4763	absent in the cluster14
ORF1b:E1264D	4904	absent in the cluster14
S:T20N	4908	absent in the cluster14
ORF1a:S3675-	4840	present in all clusters of the TP1 group
ORF1a:G3676-	4840	present in all clusters of the TP1 group
ORF1a:F3677-	4839	present in all clusters of the TP1 group
**cluster 10 (665 samples)**		
ORF1a:S2947N	665	
**cluster 12 (104 samples)**		
ORF3a:D155Y	104	
ORF1a:D762G	104	
ORF1a:T1820I	103	
ORF3a:L83F	49	
**cluster 14 (81 samples)**		
ORF1a:D2980H	81	
N:P383L	79	

Table S7: Characteristic mutations of each cluster of T0 group.

**cluster 1 (477 samples)**		
**mutation**	**frequence (seq.)**	**remark**
ORF1a:L3930F	476	shared with clusters 5 and 8
S:S689I	103	
ORF3a:G224V	103	
S:M153T	97	
**cluster 2 (179 samples)**		
ORF1a:S142-	53	present in the B.1.195 samples of the cluster
ORF1a:K141-	53	present in the B.1.195 samples of the cluster
ORF1a:F143-	53	present in the B.1.195 samples of the cluster
**cluster 4 (1037 samples)**		
N:I292T	1033	possible highly stabilizing mutation (Timmers et al., 2021)
ORF6:I33T	1017	
S:E484K	101	shared with cluster 8 and TP1 group – the mutation strengthens the link with hACE2, providing high transmissibility and low vaccine efficiency (VanInsberghe et al., 2021). The association S:K417T, S:E484K and S:N501Y promotes high affinity with ACE2 (40-50% greater than the wild type) and resistance to antibodies (Gan et al., 2021)
**cluster 5 (77 samples)**		
ORF9b:P10S	77	
ORF3a:T151I	77	
ORF1a:V2588F	77	
ORF1a:Q3777H	77	
ORF1a:P2287S	77	
ORF1a:L3930F	77	shared with clusters 1 and 8
ORF1a:L3027F	77	
N:P13L	77	provides stabilization (Periwal et al., 2021), highly observed in the world (Timmers et al., 2021), potentially increases transmissibility and death rate (Oulas et al., 2021)
**cluster 6 (531 samples)**		
ORF3a:S253P	118	characteristic of TP1 group
**cluster 8 (1090 samples)**		
ORF1a:L3930F	1089	shared with clusters 1 and 5
N:A119S	1089	
S:E484K	1088	characteristic of TP1 group, shared with cluster 4 – the mutation strengthens the link with hACE2, providing high transmissibility and low vaccine efficiency (VanInsberghe et al., 2021). The association S:K417T, S:E484K and S:N501Y promotes high affinity with ACE2 (40-50% greater than the wild type) and resistance to antibodies (Gan et al., 2021)
N:M234I	1085	
ORF1a:L3468V	1012	
**cluster 11 (250 samples)**		
S:N501Y	250	characteristic of TP1 group – the mutation strengthens the link with hACE2, providing high transmissibility and low vaccine efficiency (VanInsberghe et al., 2021). The association S:K417T, S:E484K and S:N501Y promotes high affinity with ACE2 (40-50% greater than the wild type) and resistance to antibodies (Gan et al., 2021)
ORF1a:T1001I	249	
S:T716I	248	
S:S982A	248	in association of S:S982A and S:D614G is related to the conformational variation of the spike, promoting high binding affinity with ACE2 and increased replication (Ostrov, 2021)
S:D1118H	248	
S:A570D	248	in association of S:S982A and S:D614G is related to the conformational variation of the spike, promoting high binding affinity with ACE2 and increased replication (Ostrov, 2021)
ORF8:Y73C	248	
ORF8:R52I	248	

ORF1a:A1708D	248	
N:S235F	248	S:P681H together with N:S235F potentially characterizes immune escape (Voss et al., 2021)
S:P681H	247	causes greater infectivity due to changes in protein conformation that increase affinity with the furin enzyme (Mohammad,etal,2021). S:P681H together with N:S235F potentially characterizes immune escape (Voss et al., 2021)
N:D3L	246	
ORF8:Q27*	241	
ORF1a:I2230T	241	
ORF1a:S3675-	237	characteristic of TP1 group
ORF1a:G3676-	237	characteristic of TP1 group
ORF1a:F3677-	237	characteristic of TP1 group
S:H69-	233	the pair of H69/V70 inserts increases infectivity (Meng et al., 2021)
S:V70-	231	the pair of H69/V70 inserts increases infectivity (Meng et al., 2021)
S:Y144-	230	
**cluster 13 (79 samples)**		
S:L452R	79	mutation makes the virus 20% more transmissible, increases *in vitro* infectivity, and reduces antibody neutralization (Deng et al., 2021), conferring low vaccine efficacy (VanInsberghe et al., 2021)
ORF3a:V50I	79	
ORF1b:Y822C	79	
ORF1a:Q3729K	79	
ORF1a:P971L	79	
ORF1a:L3915F	79	
ORF1a:L3201P	79	
ORF1a:A516T	79	
ORF1a:A3143V	79	
N:A220V	79	changes the conformation of proteins with the potential to alter pathogenicity and/or response to vaccination, affecting the activity of the protein binding site. A mutation with 163k cases worldwide and increasing, one of the 10 most frequent in the world, but rare in Brazil (Fang et al., 2021; Vilar and Isom, 2021).
S:I720V	78	
S:Q173K	77	the mutation potentially increases the fitness of the virus (Pater et al., 2021)
S:N164K	76	
S:S704L	75	
S:G142V	70	

Table S8: Worldwide sequences near to P.1 in 2020, defined by Euclidean distance. There are listed the 91 unique sequences closest to the 50 Brazilian samples of P.1 from 2020.

ID	variant
hCoV-19/Peru/HUA-INS-677/2020	P.1
hCoV-19/Peru/LOR-INS-575/2020	P.1
hCoV-19/Peru/HUA-INS-691/2020	P.1
hCoV-19/Peru/LOR-INS-591/2020	P.1
hCoV-19/Peru/HUA-INS-692/2020	P.1
hCoV-19/Brazil/AM-CD1739/2020	P.4
hCoV-19/Peru/HUA-INS-683/2020	P.1
hCoV-19/Brazil/PA-IEC173137/2020	B.1.1.28
hCoV-19/Brazil/PA-IEC164747/2020	B.1.1.28
hCoV-19/Brazil/SP-146/2020	B.1.1.28
hCoV-19/Brazil/SP-601/2020	B.1.1.28
hCoV-19/Brazil/SP-861/2020	B.1.1.28
hCoV-19/Brazil/SP-863/2020	B.1.1.28
hCoV-19/Brazil/SP-830/2020	B.1.1.28
hCoV-19/Brazil/PA-IEC164173/2020	B.1.1.28
hCoV-19/Brazil/PA-IEC172961/2020	B.1.1.28
hCoV-19/Brazil/PA-IEC173012/2020	B.1.1.28
hCoV-19/Brazil/SP-170/2020	B.1.1.28
hCoV-19/Brazil/PA-IEC162802/2020	B.1.1.28
hCoV-19/Brazil/AM-FIOCRUZ-20890261MV/2020	B.1.1.28
hCoV-19/Brazil/PA-IEC173159/2020	B.1.1.28
hCoV-19/Brazil/PA-IEC163469/2020	B.1.1.28
hCoV-19/Brazil/PA-IEC165302/2020	B.1.1.28
hCoV-19/England/MILK-4BC773/2020	B.1.1
hCoV-19/England/LOND-D4B5F/2020	B.1.1
hCoV-19/England/LOND-D4C3E/2020	B.1.1
hCoV-19/England/LOND-D4BD7/2020	B.1.1
hCoV-19/USA/WA-S2765/2020	B.1.1
hCoV-19/Japan/PG-2253/2020	B.1.1.48
hCoV-19/India/MH-ACTREC-539/2020	B.1.1.306
hCoV-19/USA/NC-UNC-0021/2020	B.1.1
hCoV-19/England/ALDP-952525/2020	B.1.1
hCoV-19/Mexico/HID-InDRE-IBT-66/2020	B.1.1
hCoV-19/England/OXON-AD15D/2020	B.1.1.10
hCoV-19/Portugal/PT1580/2020	B.1.1.421
hCoV-19/USA/WA-S2788/2020	B.1.1
hCoV-19/USA/WA-S2771/2020	B.1.1
hCoV-19/Russia/RYA-RII-MH2824S/2020	B.1.1
hCoV-19/Peru/LIM-UPCH-0320/2020	B.1.1.485
hCoV-19/USA/MA-MGH-03345/2020	B.1.1.192
hCoV-19/USA/MA-MGH-03296/2020	B.1.1.192
hCoV-19/CzechRepublic/Tilia-10/2020	B.1.1
hCoV-19/USA/TX-HMH-MCoV-12328/2020	B.1.1.192
hCoV-19/USA/TX-HMH-MCoV-12310/2020	B.1.1.192
hCoV-19/USA/TX-HMH-MCoV-12290/2020	B.1.1.192
hCoV-19/Russia/MOS-CRIE-7182855/2020	B.1.1
hCoV-19/England/ALDP-95B0FE/2020	B.1.1
hCoV-19/England/ALDP-74622F/2020	B.1.1
hCoV-19/England/ALDP-95B119/2020	B.1.1
hCoV-19/Brazil/PE-UFPE045/2020	B.1.1
hCoV-19/USA/NC-UNC-0017/2020	B.1.1.1
hCoV-19/England/MILK-9C72D9/2020	B.1.1.198
hCoV-19/Luxembourg/LNS1014689/2020	B.1.1.198
hCoV-19/Luxembourg/LNS8803466/2020	B.1.1.198

hCoV-19/Luxembourg/LNS8092973/2020	B.1.1.198
hCoV-19/Luxembourg/LNS9698899/2020	B.1.1.198
hCoV-19/Luxembourg/LNS7788775/2020	B.1.1.198
hCoV-19/Luxembourg/LNS6380996/2020	B.1.1.198
hCoV-19/Luxembourg/LNS3116383/2020	B.1.1.198
hCoV-19/Luxembourg/LNS1807556/2020	B.1.1.198
hCoV-19/Luxembourg/LNS9933159/2020	B.1.1.198
hCoV-19/Portugal/PT1557/2020	B.1.1.198
hCoV-19/Portugal/PT1582/2020	B.1.1.198
hCoV-19/Luxembourg/LNS6959492/2020	B.1.1.198
hCoV-19/Luxembourg/LNS9629548/2020	B.1.1.198
hCoV-19/Luxembourg/LNS0797488/2020	B.1.1.198
hCoV-19/Luxembourg/LNS5350981/2020	B.1.1.198
hCoV-19/Luxembourg/LNS1987832/2020	B.1.1.198
hCoV-19/Luxembourg/LNS2205106/2020	B.1.1.198
hCoV-19/Luxembourg/LNS2509324/2020	B.1.1.198
hCoV-19/Luxembourg/LNS4028717/2020	B.1.1.198
hCoV-19/England/QEUH-963A5F/2020	B.1.1.198
hCoV-19/Luxembourg/LNS3483404/2020	B.1.1.198
hCoV-19/NorthMacedonia/29205/2020	B.1.1.428
hCoV-19/Japan/IC-0049/2020	B.1.1.1
hCoV-19/Germany/SH-ChVir8194/2020	B.1.1
hCoV-19/NorthMacedonia/28903/2020	B.1.1.428
hCoV-19/Wales/PHWC-168269/2020	B.1.1
hCoV-19/USA/TX-QDX-3487/2020	B.1.1
hCoV-19/Chile/RM-209217/2020	N.4
hCoV-19/England/MILK-9E0A3C/2020	B.1.1.198
hCoV-19/England/MILK-B062FA/2020	B.1.1.46
hCoV-19/USA/TX-QDX-3498/2020	B.1.1
hCoV-19/mink/Lithuania/MR-LUHS-Eilnr147952/2020	B.1.1.464
hCoV-19/Brazil/RS-31GGPHCPA/2020	B.1.1.28
hCoV-19/England/ALDP-B1F8CF/2020	B.1.1.46
hCoV-19/Lithuania/MR-LUHS-Eilnr352/2020	B.1.1.280
hCoV-19/USA/FL-BPHL-2135/2020	B.1
hCoV-19/USA/PR-CDC-S50/2020	B.1
hCoV-19/Latvia/889/2020	B.1.1.280
hCoV-19/Latvia/891/2020	B.1.1.280

Table S9. List of recombinants indicated by the RAPR tool for the Brazilian P.1 sequences in 2020. The asterisk indicates the events that potentially generated B.1.1 and B.1.1.28 cluster 6 recombinants from P.1 and the USA sequence of B.1.1.1.

recombinant sample	parental sequence 1	parental sequence 2	p-value
*Brazil/PA-IEC162802 (EPI_ISL_458140 - B.1.1.28)	Brazil/AM-FIOCRUZ-20143088ER (EPI_ISL_1068110 - P.1)	USA/NC-UNC-0017 (EPI_ISL_831339 - B.1.1.1)	$5{,}12 \times 10^{-6}$
*Brazil/AM-FIOCRUZ-20890261MV (EPI_ISL_801402 - B.1.1.28)	Brazil/AM-FIOCRUZ-20143088ER (EPI_ISL_1068110 - P.1)	USA/NC-UNC-0017 (EPI_ISL_831339 - B.1.1.1)	$3{,}01 \times 10^{-6}$
Brazil/RS-31GGPHCPA (EPI_ISL_1163736 - B.1.1.28)	Brazil/PB-BA1200-250009713 (EPI_ISL_2241496 - P.1)	Lithuania/MR-LUHS-Eilnr352 (EPI_ISL_636871 - B.1.1.280)	$4{,}805 \times 10^{-4}$
Brazil/PE-UFPE045 (EPI_ISL_1117381 - B.1.1)	USA/NC-UNC-0017 (EPI_ISL_831339 - B.1.1.1)	Brazil/PA-IEC173159 (EPI_ISL_848562 - B.1.1.28)	$3{,}996 \times 10^{-4}$
Wales/PHWC-168269 (EPI_ISL_494217 - B.1.1)	Brazil/AM-FIOCRUZ-20143088ER (EPI_ISL_1068110 - P.1)	USA/NC-UNC-0017 (EPI_ISL_831339 - B.1.1.1)	$4{,}96 \times 10^{-7}$
Russia/RYA-RII-MH2824S (EPI_ISL_639968 - B.1.1)	Brazil/AM-FIOCRUZ-20143088ER (EPI_ISL_1068110 - P.1)	USA/NC-UNC-0017 (EPI_ISL_831339 - B.1.1.1)	$2{,}08 \times 10^{-5}$
Chile/RM-209217 (EPI_ISL_1167679 - N.4)	Brazil/PB-BA1200-250009713 (EPI_ISL_2241496 - P.1)	USA/NC-UNC-0017 (EPI_ISL_831339 - B.1.1.1)	$1{,}46 \times 10^{-7}$
Russia/MOS-CRIE-7182855 (EPI_ISL_745223 - B.1.1)	Brazil/AM-FIOCRUZ-20143088ER (EPI_ISL_1068110 - P.1)	USA/NC-UNC-0017 (EPI_ISL_831339 - B.1.1.1)	$2{,}08 \times 10^{-5}$
Peru/LIM-UPCH-0320 (EPI_ISL_812477 - B.1.1.485)	Brazil/AM-FIOCRUZ-20143103JT (EPI_ISL_833136 - P.1)	USA/NC-UNC-0017 (EPI_ISL_831339 - B.1.1.1)	$3{,}82 \times 10^{-7}$
CzechRepublic/Tilia-10 (EPI_ISL_906062 - B.1.1)	Peru/HUA-INS-683 (EPI_ISL_1111143 - P.1)	USA/NC-UNC-0017 (EPI_ISL_831339 - B.1.1.1)	$2{,}33 \times 10^{-7}$
Mexico/HID-InDRE-IBT-66 (EPI_ISL_1301549 - B.1.1)	Japan/IC-0049 (EPI_ISL_591391 - B.1.1.1)	USA/NC-UNC-0017 (EPI_ISL_831339 - B.1.1.1)	$1{,}748 \times 10^{-4}$
England/MILK-9E0A3C (EPI_ISL_581366 - B.1.1.46)	Russia/RYA-RII-MH2824S (EPI_ISL_639968 - B.1.1)	USA/NC-UNC-0017 (EPI_ISL_831339 - B.1.1.1)	$2{,}057 \times 10^{-4}$
Lithuania/MR-LUHS-Eilnr352 (EPI_ISL_636871 - B.1.1.280)	Luxembourg/LNS5350981 (EPI_ISL_744221 - B.1.1.98)	USA/NC-UNC-0017 (EPI_ISL_831339 - B.1.1.1)	$3{,}702 \times 10^{-4}$
USA/FL-BPHL-2135 (EPI_ISL_653288 - B.1)	Brazil/AM-FIOCRUZ-20890261MV (EPI_ISL_801402 - B.1.1.28)	USA/NC-UNC-0017 (EPI_ISL_831339 - B.1.1.1)	$9{,}991 \times 10^{-4}$
Portugal/PT1580 (EPI_ISL_693533 - B.1.1.421)	USA/NC-UNC-0017 (EPI_ISL_831339 - B.1.1.1)	Brazil/PA-IEC173159 (EPI_ISL_848562 - B.1.1.28)	$3{,}996 \times 10^{-4}$
Germany/SH-ChVir8194 (EPI_ISL_729470 - B.1.1)	Brazil/AM-FIOCRUZ-20890261MV (EPI_ISL_801402 - B.1.1.28)	USA/NC-UNC-0017 (EPI_ISL_831339 - B.1.1.1)	$9{,}99 \times 10^{-4}$
Luxembourg/LNS1014689 (EPI_ISL_739870 - B.1.1.198)	England/OXON-AD15D (EPI_ISL_448567 - B.1.1.10)	USA/NC-UNC-0017 (EPI_ISL_831339 - B.1.1.1)	$9{,}99 \times 10^{-4}$
USA/TX-HMH-MCoV-12290 (EPI_ISL_789934 - B.1.1.192)	England/MILK-9E0A3C (EPI_ISL_581366 - B.1.1.46)	USA/NC-UNC-0017 (EPI_ISL_831339 - B.1.1.1)	$2{,}057 \times 10^{-4}$
mink/Lithuania/MR-LUHS-Eilnr147952 (EPI_ISL_851056 - B.1.1.464)	England/ALDP-B1F8CF (EPI_ISL_646057 - B.1.1.46)	USA/NC-UNC-0017 (EPI_ISL_831339 - B.1.1.1)	$7{,}14 \times 10^{-5}$
USA/TX-QDX-3498 (EPI_ISL_877125 - B.1.1)	Russia/RYA-RII-MH2824S (EPI_ISL_639968 - B.1.1)	USA/NC-UNC-0017 (EPI_ISL_831339 - B.1.1.1)	$2{,}057 \times 10^{-4}$
Luxembourg/LNS6380996 (EPI_ISL_744406 - B.1.1.198)	England/OXON-AD15D (EPI_ISL_448567 - B.1.1.10)	USA/NC-UNC-0017 (EPI_ISL_831339 - B.1.1.1)	$9{,}990 \times 10^{-4}$
Latvia/889 (EPI_ISL_1312400 - B.1.1.280)	England/MILK-9E0A3C (EPI_ISL_581366 - B.1.1.46)	Lithuania/MR-LUHS-Eilnr352 (EPI_ISL_636871 - B.1.1.280)	$5{,}97 \times 10^{-5}$

Table S10. Richness and coverage by states in Brazil, using Chao 1 (Chao, 1984). Number of different refers to the number of non-redundant sequences. The states of RR, PI, RN and MS could not be analyzed due to low sampling.

State	Number of different	Coverage	Richness
RO	27	0,2899	93,13
AC	10	0,198	50,50
AM	203	0,2699	752,13
RR	12	–	–
PA	112	0,1765	634,56
AP	59	0,072 37	815,25
TO	14	0,1421	98,52
MA	99	0,0447	2214,76
PI	11	–	–
CE	32	0,1245	257,03
RN	54	–	–
PB	138	0,2022	682,49
PE	63	0,097 95	643,18
AL	121	0,072 84	1661,17
SE	99	0,1833	540,10
BA	162	0,1879	862,16
MG	105	0,078 94	1330,12
ES	52	0,115	452,17
RJ	842	0,2067	4073,54
SP	3095	0,3028	10 221,27
PR	109	0,1396	780,80
SC	102	0,0897	1137,12
RS	433	0,1438	3011,13
MS	62	–	–
MT	31	0,1454	213,20
GO	382	0,1726	2213,21
DF	15	0,1508	99,47

States: Acre (AC), Alagoas (AL), Amapá (AP), Amazonas (AM), Bahia (BA), Ceará (CE), Distrito Federal (DF), Espírito Santo (ES), Goiás (GO), Maranhão (MA), Mato Grosso (MT), Mato Grosso do Sul (MS), Minas Gerais (MG), Pará (PA), Paraíba (PB), Paraná (PR), Pernambuco (PE), Piauí (PI), Rio de Janeiro (RJ), Rio Grande do Norte (RN), Rio Grande do Sul (RS), Rondônia (RO), Roraima (RR), Santa Catarina (SC), São Paulo (SP), Sergipe (SE), Tocantins (TO).

Table S11. Coverage and Richness estimated for each cluster using Chao 1 (Chao, 1984). Number of different refers to the number of non-redundant sequences.

Cluster	number of different	Coverage	Richness
CL15-1	376	0,1501	2504,0
CL15-2	115	0,2713	423,9
CL15-3	1950	0,2785	7001,0
CL15-4	736	0,1870	3936,0
CL15-5	56	0,1686	332,1
CL15-6	386	0,1850	2086,0
CL15-7	575	0,1981	2903,0
CL15-8	861	0,1903	4525,0
CL15-9	42	0,2915	144,1
CL15-10	470	0,3006	1563,0
CL15-11	179	0,3367	531,7
CL15-12	68	0,2124	320,1
CL15-13	47	0,2556	183,9
CL15-14	47	0,4085	115,1
CL15-15	238	0,2073	1148,0
Total	6146		27 716,9

Table S12. Total samples taken by state in the years 2020 and 2021 (GISAID release 609), compared to the total number of quality samples. The second column is the total population of the state in millions, according to the IBGE – Instituto Brasileiro de Geografia e Estatística `https://www.ibge.gov.br/cidades-e-estados`. There is a marked imbalance in the sampling between Brazilian states.

	population (millions)	2020	2021	with quality (2020/21)
AC	0,894	26	2	11
AL	3,351	23	80	100
AP	0,861	39	45	72
AM	4,207	417	132	309
BA	14,930	126	152	211
CE	9,187	50	29	34
ES	4,064	32	38	55
GO	7,113	86	757	489
MA	7,114	43	121	131
MT	3,526	19	4	2
MS	2,809	62	31	71
MG	21,292	161	152	114
PA	8,690	110	66	147
PB	4,039	125	69	171
PR	11,516	162	79	142
PE	9,616	190	17	70
PI	3,281	9	10	11
RJ	17,366	704	1354	1170
RN	3,534	29	69	68
RS	11,422	691	263	556
RO	1,796	8	26	31
RR	0,631	6	23	14
SC	7,252	26	161	151
SP	46,289	1176	5143	4386
SE	2,318	28	142	157
TO	1,590	15	12	15
DF	3,055	27	4	21

Table S13. Comparison of clusters composition between the PANGO nomenclature v3.0.5 from 2021-06-04 and v3.1.11 from 2021-08-09.

	v3.0.5 (2021-06-04)					v3.1.11 (2021-08-09)				
cluster 1	B.1.1	B.1.1.28	B.1.1.332			B.1.1	B.1.1.28	B.1.1.332	B.1.1.448	B.1.1.464
	2	467	8			6	453	8	1	1
						P.1	P.2	P.7		
						1	3	4		
cluster 2	A.1	A.2	B	B.1	B.1.111	A.1	A.2	B	B.1	B.1.111
	1	3	3	59	5	1	3	3	58	5
	B.1.160.25	B.1.177.32	B.1.177.52	B.1.195	B.1.212	B.1.160.25	B.1.177.32	B.1.177.52	B.1.195	B.1.212
	1	1	1	54	15	1	1	1	54	15
	B.1.234	B.1.240	B.1.319	B.1.351	B.1.547	B.1.234	B.1.240	B.1.351	B.1.479	B.1.547
	2	1	1	5	1	3	1	5	1	1
	B.1.575	B.1.617.2	B.1.9	B.1.91	B.3	B.1.617.2	B.1.623	B.1.9	B.1.91	B.3
	1	1	1	15	2	1	1	1	15	2
	B.39	B.6				B.39	B.6			
	5	1				5	1			
cluster 3	B.1	B.1.1	B.1.617.1	P.1	P.1.1	P.1	P.1.1	P.1.2	P.1.3	P.1.7
	1	1	2	2966	18	3013	19	12	1	17
	P.1.2	P.2	P.4							
	26	27	21							
cluster 4	B.1	B.1.1	B.1.1.161	B.1.1.33	N.1	B.1	B.1.1	B.1.1.161	B.1.1.277	B.1.1.33
	4	11	7	909	2	5	11	7	1	907
	N.10	N.4	N.9			N.1	N.10	N.4	N.9	
	19	4	81			1	24	4	77	
cluster 5	B.1.1.28					P.7				
	77					77				
cluster 6	B.1	B.1.1	B.1.1.192	B.1.1.277	B.1.1.28	B.1	B.1.1	B.1.1.192	B.1.1.277	B.1.1.28
	1	171	1	3	317	1	158	1	3	300
	B.1.1.372	B.1.1.378	B.1.1.462	B.1.1.519	B.1.1.74	B.1.1.371	B.1.1.372	B.1.1.378	B.1.1.462	B.1.1.515
	1	29	1	2	1	3	1	29	1	1
	B.59	C.14	C.37			B.1.1.519	B.1.1.74	B.59	B.60	C.14
	1	1	2			2	1	1	7	1
						C.37	P.5			
						2	19			
cluster 7	P.1	P.1.1	P.1.2	P.2	P.4	P.1	P.1.1	P.1.2	P.1.7	
	4	4	8	6	732	742	4	3	5	
cluster 8	B.1.1.28	P.2				B.1.1	B.1.1.28	P.2		
	1	1089				2	14	1074		
cluster 9	P.1	P.1.2	P.4			P.1	P.1.2	P.1.7		
	31	4	15			46	1	3		
cluster 10	P.1	P.1.1	P.1.2	P.2	P.4	P.1	P.1.1	P.1.2	P.1.3	
	491	2	3	14	155	659	2	3	1	
cluster 11	B.1.1	B.1.1.7	P.1			B.1.1	B.1.1.7	None		
	1	248	1			2	242	6		
cluster 12	P.1	P.1.2				P.1	P.1.2			
	4	100				4	100			
cluster 13	P.4					P.4				
	79					79				
cluster 14	B.1.1.28	P.1	P.4			P.1				
	2	74	5			81				
cluster 15	P.1	P.1.1	P.1.2	P.4		P.1	P.1.1	P.1.7		
	1	4	12	267		268	4	12		

REFERENCES

Berger, I. and Schaffitzel, C. (2020). The SARS-CoV-2 spike protein: balancing stability and infectivity. *Cell research* 30, 1059–1060. doi:10.1038/s41422-020-00430-4

Chao, A. (1984). Nonparametric estimation of the number of classes in a population. *Scandinavian Journal of statistics* , 265–270

Colson, P., Levasseur, A., Delerce, J., Pinault, L., Dudouet, P., Devaux, C., et al. (2021). Spreading of a new SARS-CoV-2 N501Y spike variant in a new lineage. *Clinical Microbiology and Infection* doi:10.1016/j.cmi.2021.05.006

Davies, N. G., Abbott, S., Barnard, R. C., Jarvis, C. I., Kucharski, A. J., Munday, J. D., et al. (2021). Estimated transmissibility and impact of sars-cov-2 lineage b.1.1.7 in england. *Science* 372. doi:10.1126/science.abg3055

Deng, X., Garcia-Knight, M. A., Khalid, M. M., Servellita, V., Wang, C., Morris, M. K., et al. (2021). Transmission, infectivity, and neutralization of a spike l452r sars-cov-2 variant. *Cell* doi:10.1016/j.cell.2021.04.025

Fang, S., Liu, S., Shen, J., Lu, A. Z., Zhang, Y., Li, K., et al. (2021). Updated SARS-CoV-2 Single Nucleotide Variants and Mortality Association. *preprint* doi:10.1101/2021.01.29.21250757

Franceschi, V. B., Ferrareze, P. A. G., Zimerman, R. A., Cybis, G. B., and Thompson, C. E. (2021). Mutation hotspots, geographical and temporal distribution of SARS-CoV-2 lineages in Brazil, February 2020 to February 2021: insights and limitations from uneven sequencing efforts. *medRxiv* doi:10.1101/2021.03.08.21253152

Gan, H. H., Twaddle, A., Marchand, B., and Gunsalus, K. C. (2021). Structural modeling of the SARS-CoV-2 Spike/human ACE2 complex interface can identify high-affinity variants associated with increased transmissibility. *Journal of Molecular Biology* 433, 167051. doi:10.1016/j.jmb.2021.167051

González-Candelas, F., Shaw, M.-A., Phan, T., Kulkarni-Kale, U., Paraskevis, D., Luciani, F., et al. (2021). One year into the pandemic: Short-term evolution of SARS-CoV-2 and emergence of new lineages. *Infection, Genetics and Evolution* , 104869doi:10.1016/j.meegid.2021.104869

Grabowski, F., Preibisch, G., Giziński, S., Kochańczyk, M., and Lipniacki, T. (2021). SARS-CoV-2 variant of concern 202012/01 has about twofold replicative advantage and acquires concerning mutations. *Viruses* 13, 392. doi:10.3390/v13030392

Hadfield, J., Megill, C., Bell, S. M., Huddleston, J., Potter, B., Callender, C., et al. (2018). Nextstrain: real-time tracking of pathogen evolution. *Bioinformatics* 34, 4121–4123. doi:10.1093/bioinformatics/bty407

Korber, B., Fischer, W. M., Gnanakaran, S., Yoon, H., Theiler, J., Abfalterer, W., et al. (2020). Tracking changes in SARS-CoV-2 spike: evidence that D614G increases infectivity of the COVID-19 virus. *Cell* 182, 812–827. doi:10.1016/j.cell.2020.06.043

Meng, B., Kemp, S. A., Papa, G., Datir, R., Ferreira, I. A., Marelli, S., et al. (2021). Recurrent emergence of sars-cov-2 spike deletion h69/v70 and its role in the alpha variant b.1.1.7. *Cell reports* 35, 109292. doi:10.1016/j.celrep.2021.109292

Naveca, F., Nascimento, V., Souza, V., Corado, A., Nascimento, F., Silva, G., et al. (2021). COVID-19 epidemic in the Brazilian state of Amazonas was driven by long-term persistence of endemic SARS-CoV-2 lineages and the recent emergence of the new Variant of Concern P.1. *preprint* doi:10.21203/rs.3.rs-275494/v1

Ostrov, D. A. (2021). Structural consequences of variation in sars-cov-2 b.1.1.7. *Journal of cellular immunology* 3, 103. doi:10.33696/immunology.3.085

Oulas, A., Zanti, M., Tomazou, M., Zachariou, M., Minadakis, G., Bourdakou, M. M., et al. (2021). Generalized linear models provide a measure of virulence for specific mutations in sars-cov-2 strains. *PloS one* 16, e0238665. doi:10.1371/journal.pone.0238665

Pater, A. A., Bosmeny, M. S., Barkau, C. L., Ovington, K. N., Chilamkurthy, R., Parasrampuria, M., et al. (2021). Emergence and evolution of a prevalent new SARS-CoV-2 variant in the United States. *bioRxiv* doi:10.1101/2021.01.11.426287

Periwal, N., Rathod, S. B., Pal, R., Sharma, P., Nebhnani, L., Barnwal, R. P., et al. (2021). In silico characterization of mutations circulating in sars-cov-2 structural proteins. *Journal of Biomolecular Structure and Dynamics* , 1–16doi:10.1080/07391102.2021.1908170

Timmers, L. F. S. M., Peixoto, J. V., Ducati, R. G., Bachega, J. F. R., de Mattos Pereira, L., Caceres, R. A., et al. (2021). Sars-cov-2 mutations in brazil: from genomics to putative clinical conditions. *Scientific reports* 11, 1–14. doi:10.1038/s41598-021-91585-6

VanInsberghe, D., Neish, A. S., Lowen, A. C., and Koelle, K. (2021). Recombinant SARS-CoV-2 genomes are currently circulating at low levels. *bioRxiv* , 2020–08doi:10.1101/2020.08.05.238386

Varabyou, A., Pockrandt, C., Salzberg, S. L., and Pertea, M. (2021). Rapid detection of inter-clade recombination in SARS-CoV-2 with Bolotie. *Genetics* 218, iyab074. doi:10.1093/genetics/iyab074

Vilar, S. and Isom, D. G. (2021). One year of SARS-CoV-2: How much has the virus changed? *Biology* 10, 91. doi:10.3390/biology10020091

Voss, C., Esmail, S., Liu, X., Knauer, M. J., Ackloo, S., Kaneko, T., et al. (2021). Epitope-specific antibody responses differentiate covid-19 outcome and variants of concern. *JCI insight* doi:10.1172/jci.insight.148855

Yurkovetskiy, L., Wang, X., Pascal, K. E., Tomkins-Tinch, C., Nyalile, T. P., Wang, Y., et al. (2020). Structural and functional analysis of the D614G SARS-CoV-2 spike protein variant. *Cell* 183, 739–751. doi:10.1016/j.cell.2020.09.032
