# Supplementary material for: Genomic landscape of the SARS-CoV-2 pandemic in Brazil suggests an external P.1 variant origin
Source: Front Microbiol. 2022 Dec 22;13:1037455. doi: 10.3389/fmicb.2022.1037455 (PMC9814972; doi:10.3389/fmicb.2022.1037455)
Supplement: Supplementary file 3 [file Data_Sheet_3.PDF]

Supplementary Materials 3 – Genomic landscape of SARS-CoV-2 pandemic in Brazil suggests an external P.1 variant origin

Camila P. Perico[1,2], Camilla R. De Pierri[1,3], Giuseppe P. Neto[1], Danrley R. Fernandes[1,2], Fabio O. Pedrosa[2,3], Emanuel M. de Souza[2,3], and Roberto T. Raittz[1,2,*]

November 10, 2021

1. Laboratory of Artificial Intelligence Applied to Bioinformatics - SEPT, Federal University of Paraná, Curtiba, Paraná, Brazil
2. Graduate Program in Bioinformatics - SEPT, Federal University of Paraná, Curtiba, Paraná, Brazil
3. Department of Biochemistry and Molecular Biology, Federal University of Paraná, Curtiba, Paraná, Brazil

Corresponding Author:
Roberto T. Raittz: raittz@ufpr.br

Lineage evolution over time through visualization with t-SNE graph. Each slide adds the samples of a new week. The colors represent each cluster obtained for Brazilian sequences. Here was adopted the nomenclature PANGO v3.0.5. The key shows the main lineages that each cluster represents. The emergence, growth and extinction of the groups is visible, as well as the gradual replacement of the T0 group variants by the TP1 group.

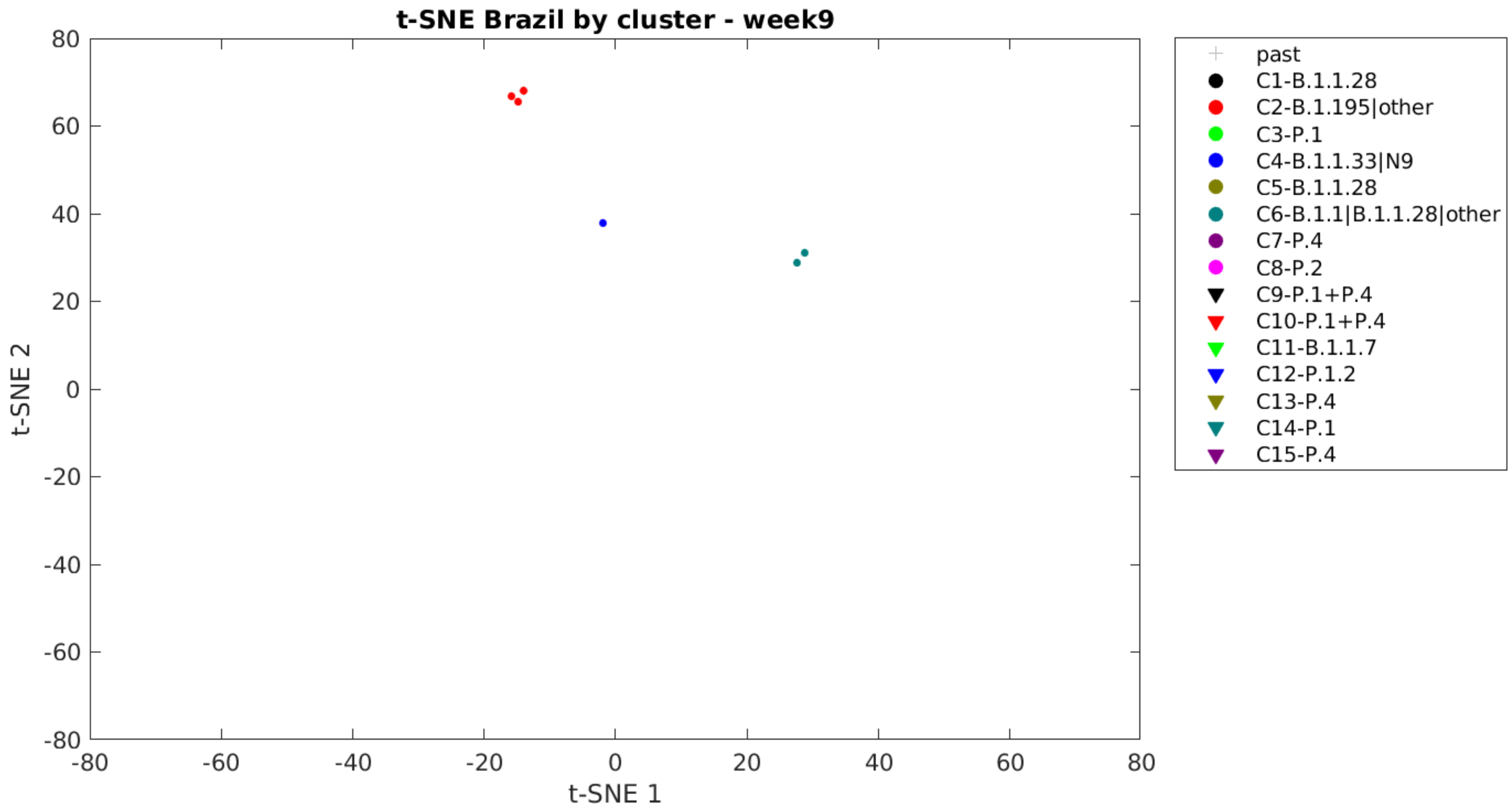
t-SNE Brazil by cluster - week9
t-SNE 2
t-SNE 1
80
60
40
20
0
-20
-40
-60
-80
-80
-60
-40
-20
0
20
40
60
80
past
C1-B.1.1.28
C2-B.1.195|other
C3-P.1
C4-B.1.1.33|N9
C5-B.1.1.28
C6-B.1.1|B.1.1.28|other
C7-P.4
C8-P.2
C9-P.1+P.4
C10-P.1+P.4
C11-B.1.1.7
C12-P.1.2
C13-P.4
C14-P.1
C15-P.4

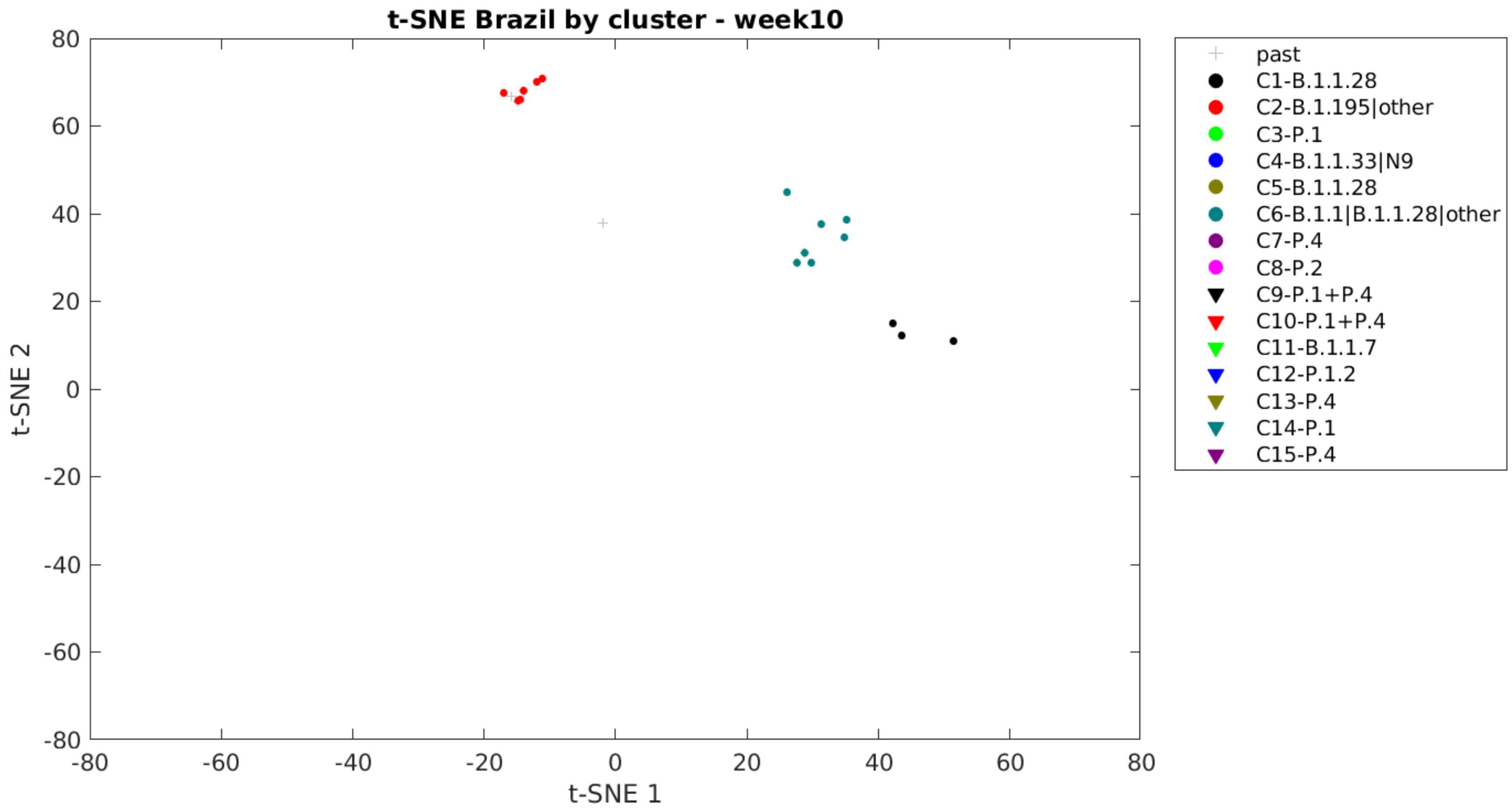
t-SNE Brazil by cluster - week10
t-SNE 1
t-SNE 2
80
60
40
20
0
-20
-40
-60
-80
past
C1-B.1.1.28
C2-B.1.195|other
C3-P.1
C4-B.1.1.33|N9
C5-B.1.1.28
C6-B.1.1|B.1.1.28|other
C7-P.4
C8-P.2
C9-P.1+P.4
C10-P.1+P.4
C11-B.1.1.7
C12-P.1.2
C13-P.4
C14-P.1
C15-P.4

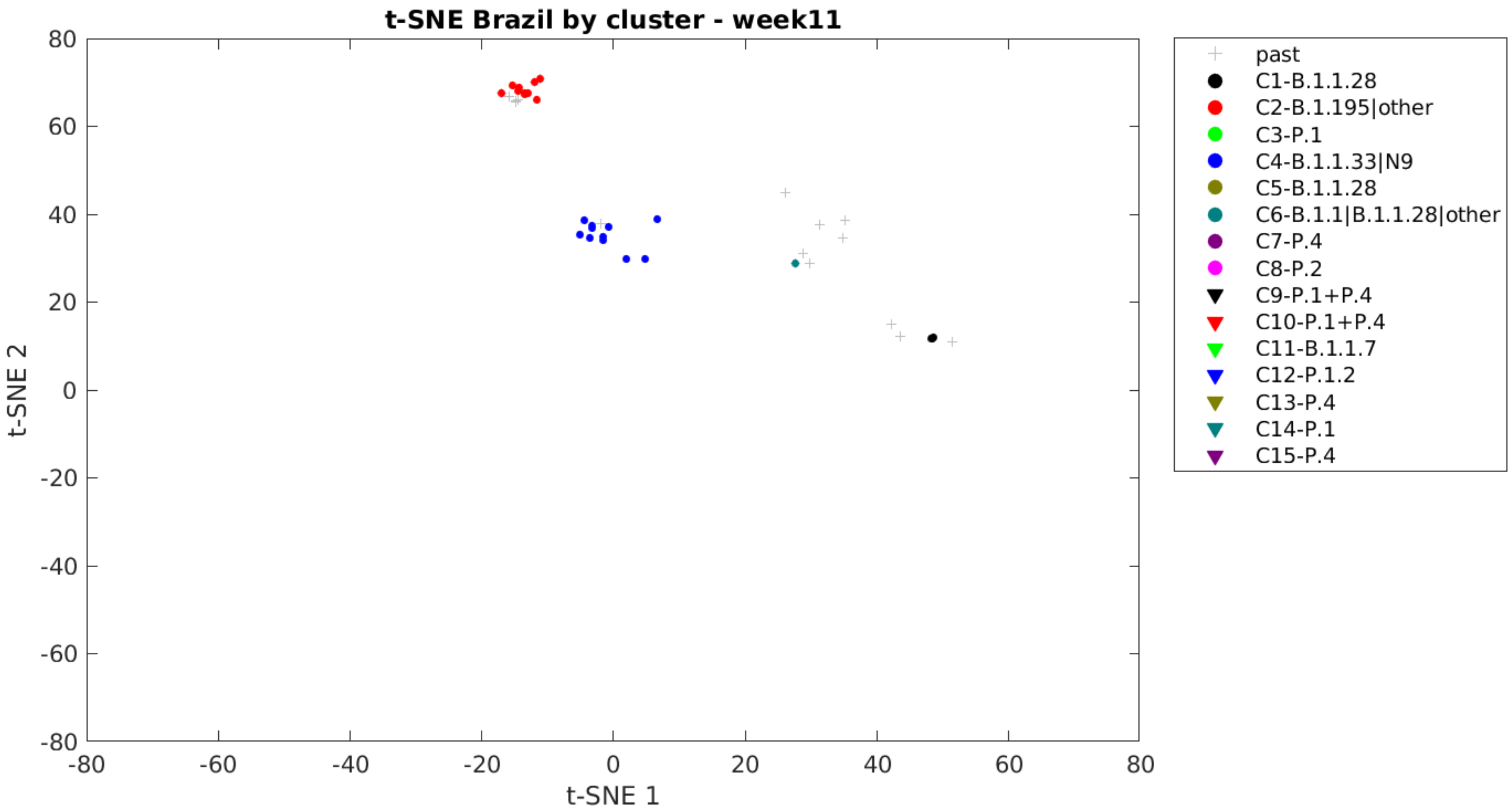

t-SNE Brazil by cluster - week11
80
60
40
20
0
-20
-40
-60
-80
t-SNE 2
-80
-60
-40
-20
0
20
40
60
80
t-SNE 1
past
C1-B.1.1.28
C2-B.1.195|other
C3-P.1
C4-B.1.1.33|N9
C5-B.1.1.28
C6-B.1.1|B.1.1.28|other
C7-P.4
C8-P.2
C9-P.1+P.4
C10-P.1+P.4
C11-B.1.1.7
C12-P.1.2
C13-P.4
C14-P.1
C15-P.4

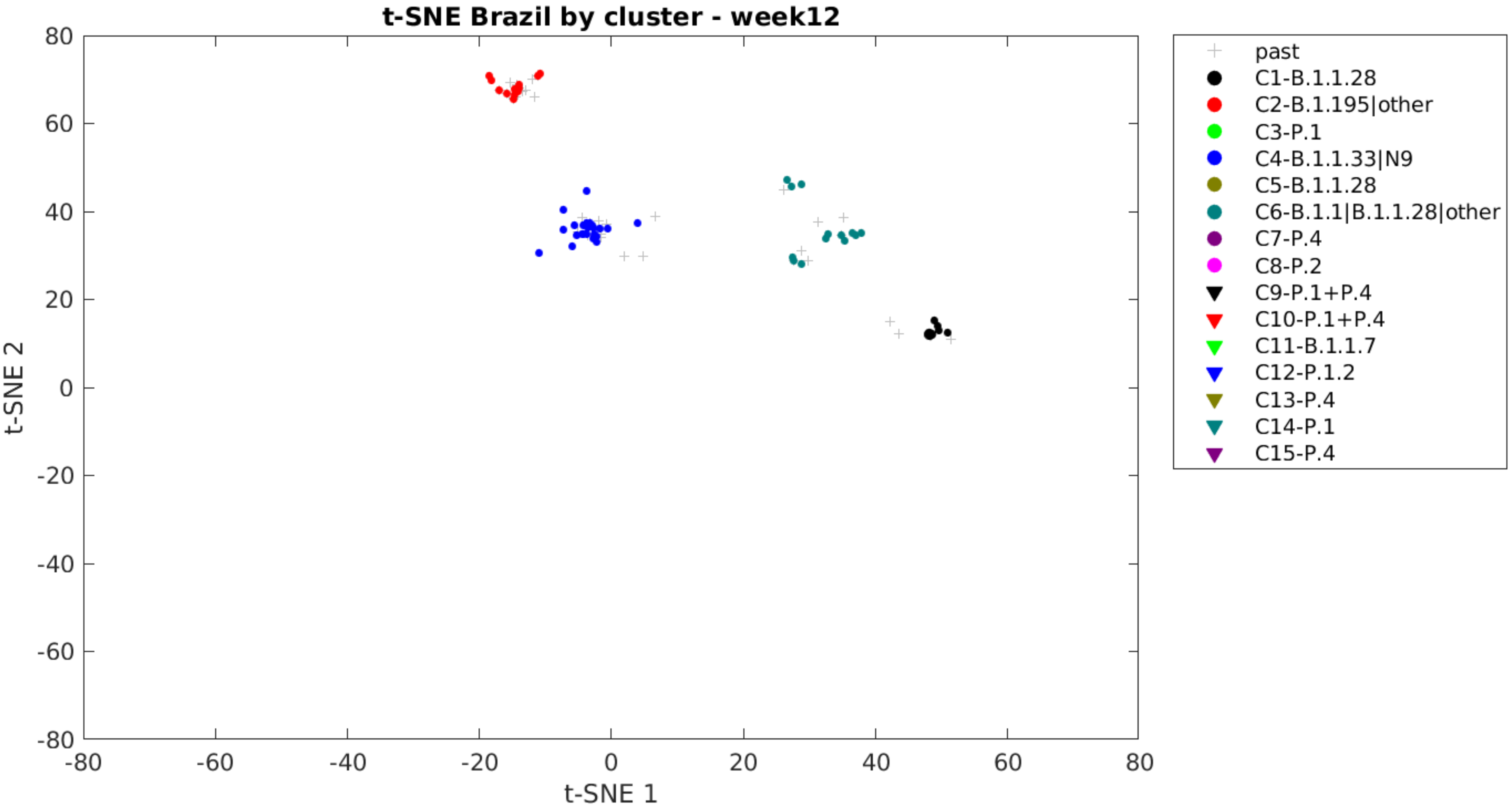
t-SNE Brazil by cluster - week12
t-SNE 2
t-SNE 1
80
60
40
20
0
-20
-40
-60
-80
-80
-60
-40
-20
0
20
40
60
80
past
C1-B.1.1.28
C2-B.1.195|other
C3-P.1
C4-B.1.1.33|N9
C5-B.1.1.28
C6-B.1.1|B.1.1.28|other
C7-P.4
C8-P.2
C9-P.1+P.4
C10-P.1+P.4
C11-B.1.1.7
C12-P.1.2
C13-P.4
C14-P.1
C15-P.4

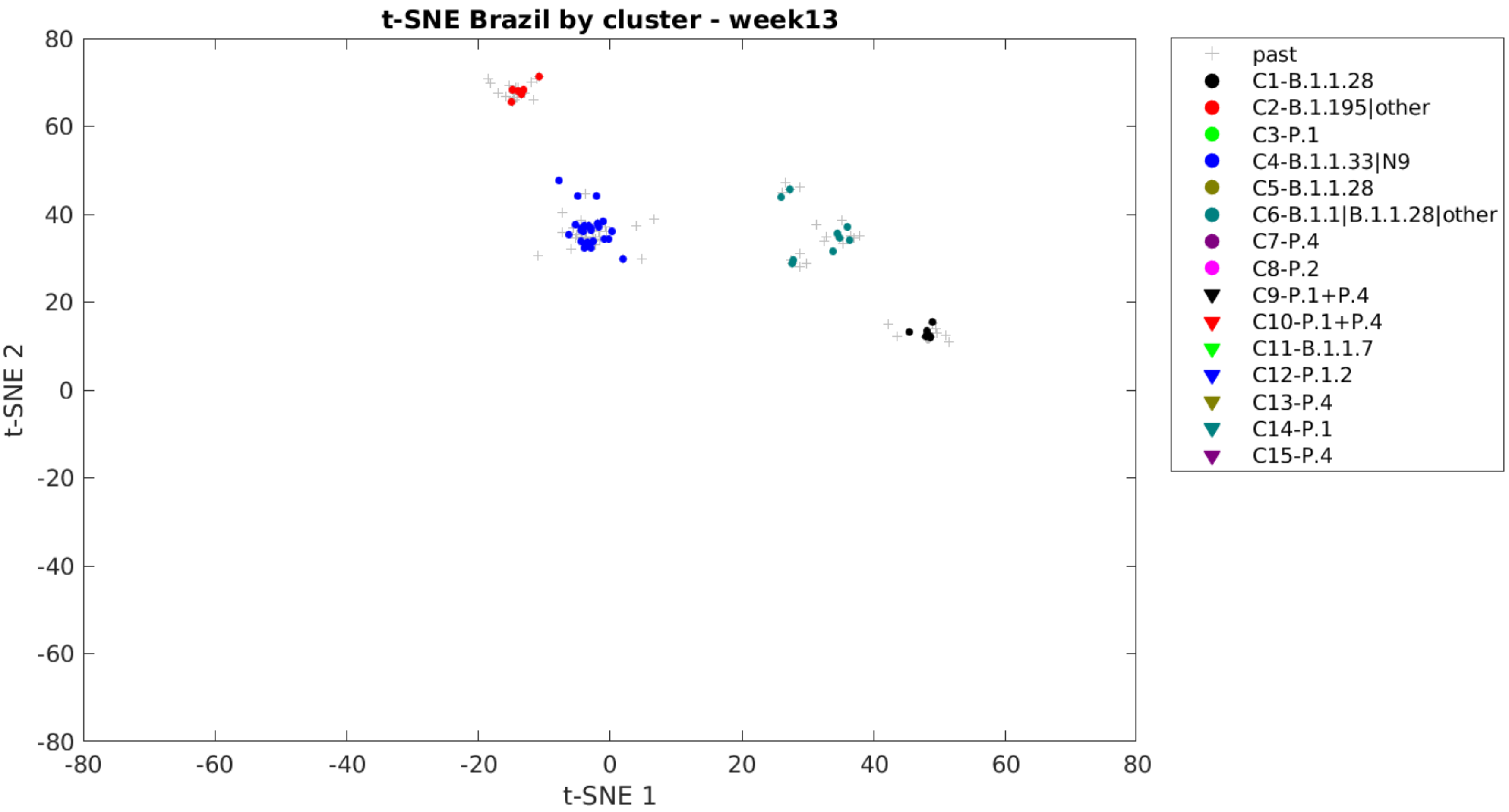

t-SNE Brazil by cluster - week13
80
60
40
20
0
-20
-40
-60
-80
-80
-60
-40
-20
0
20
40
60
80
t-SNE 1
t-SNE 2
past
C1-B.1.1.28
C2-B.1.195|other
C3-P.1
C4-B.1.1.33|N9
C5-B.1.1.28
C6-B.1.1|B.1.1.28|other
C7-P.4
C8-P.2
C9-P.1+P.4
C10-P.1+P.4
C11-B.1.1.7
C12-P.1.2
C13-P.4
C14-P.1
C15-P.4

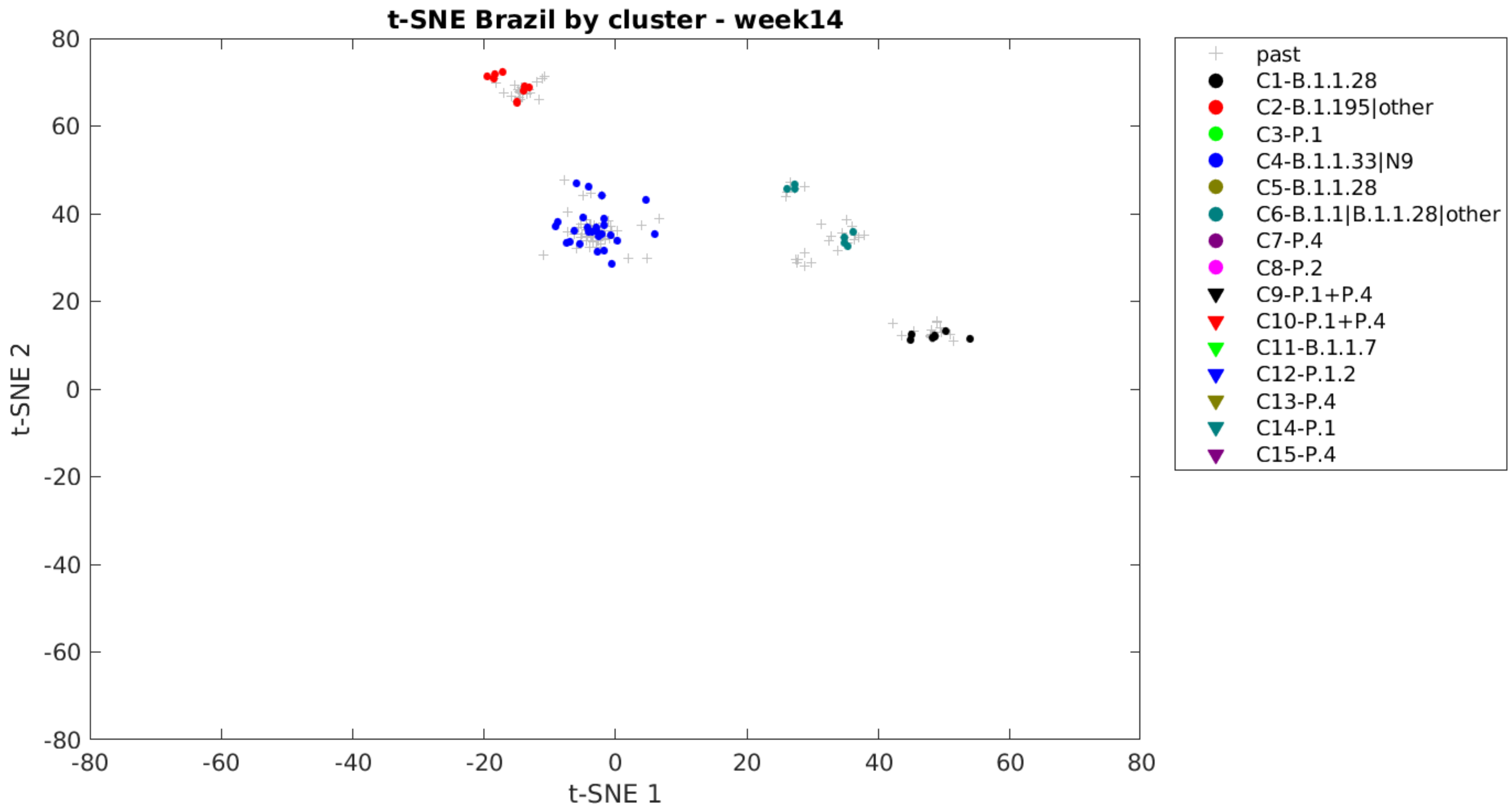

t-SNE Brazil by cluster - week14
t-SNE 2
t-SNE 1
80
60
40
20
0
-20
-40
-60
-80
-80
-60
-40
-20
0
20
40
60
80
past
C1-B.1.1.28
C2-B.1.195|other
C3-P.1
C4-B.1.1.33|N9
C5-B.1.1.28
C6-B.1.1|B.1.1.28|other
C7-P.4
C8-P.2
C9-P.1+P.4
C10-P.1+P.4
C11-B.1.1.7
C12-P.1.2
C13-P.4
C14-P.1
C15-P.4

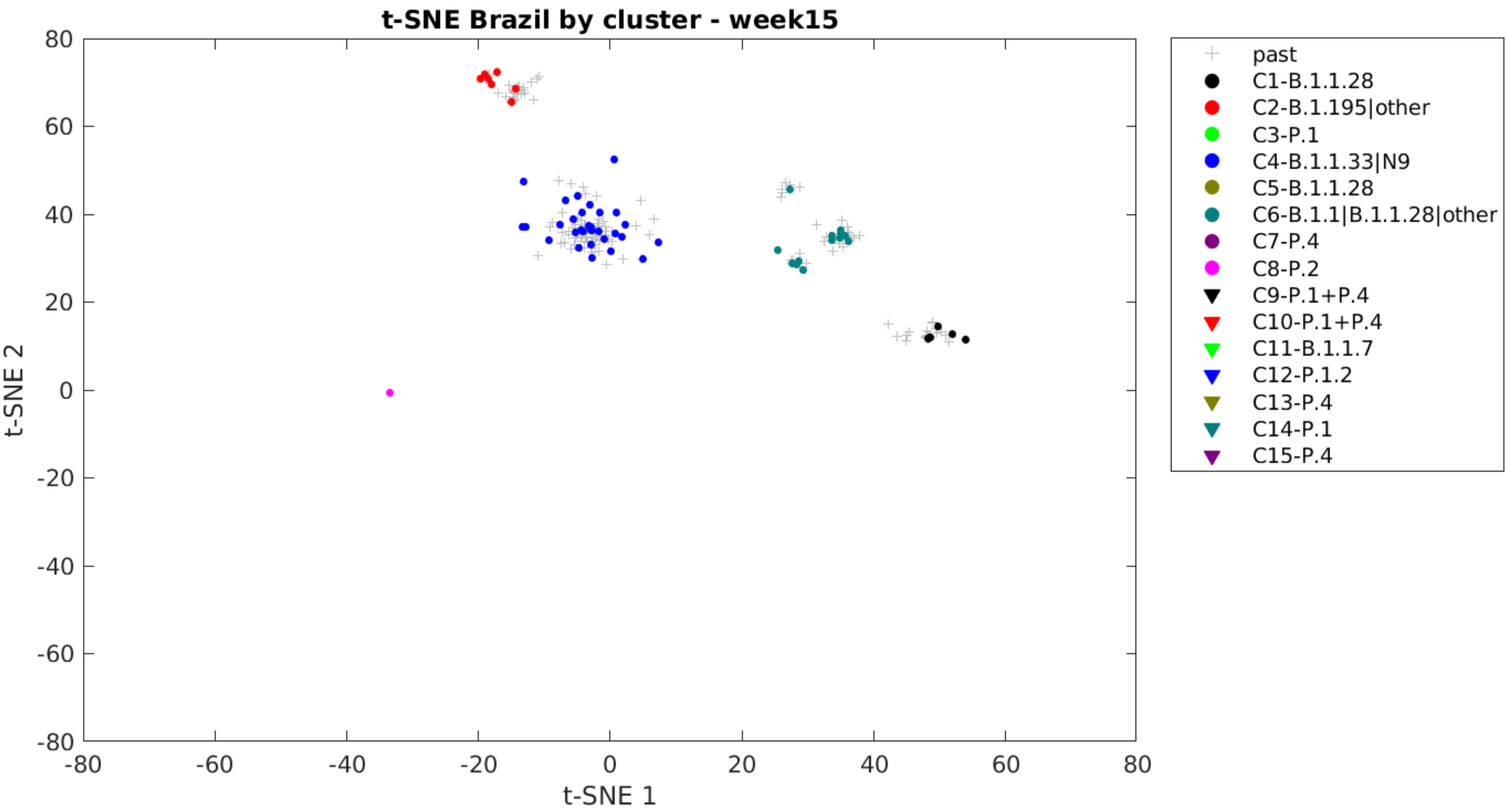
t-SNE Brazil by cluster - week15
t-SNE 2
t-SNE 1
80
60
40
20
0
-20
-40
-60
-80
-80
-60
-40
-20
0
20
40
60
80
past
C1-B.1.1.28
C2-B.1.195|other
C3-P.1
C4-B.1.1.33|N9
C5-B.1.1.28
C6-B.1.1|B.1.1.28|other
C7-P.4
C8-P.2
C9-P.1+P.4
C10-P.1+P.4
C11-B.1.1.7
C12-P.1.2
C13-P.4
C14-P.1
C15-P.4

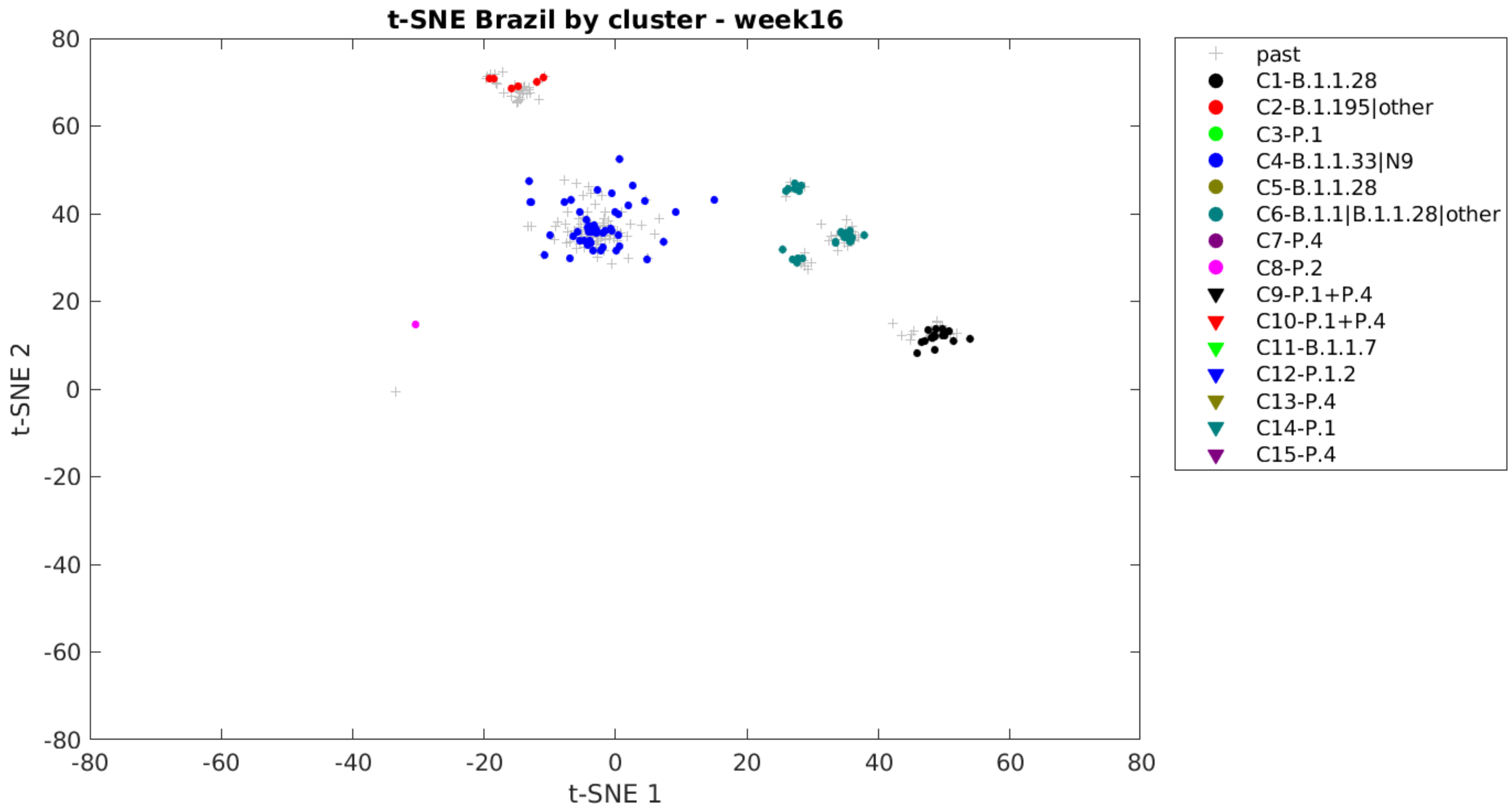
t-SNE Brazil by cluster - week16
t-SNE 2
t-SNE 1
80
60
40
20
0
-20
-40
-60
-80
-80
-60
-40
-20
0
20
40
60
80
past
C1-B.1.1.28
C2-B.1.195|other
C3-P.1
C4-B.1.1.33|N9
C5-B.1.1.28
C6-B.1.1|B.1.1.28|other
C7-P.4
C8-P.2
C9-P.1+P.4
C10-P.1+P.4
C11-B.1.1.7
C12-P.1.2
C13-P.4
C14-P.1
C15-P.4

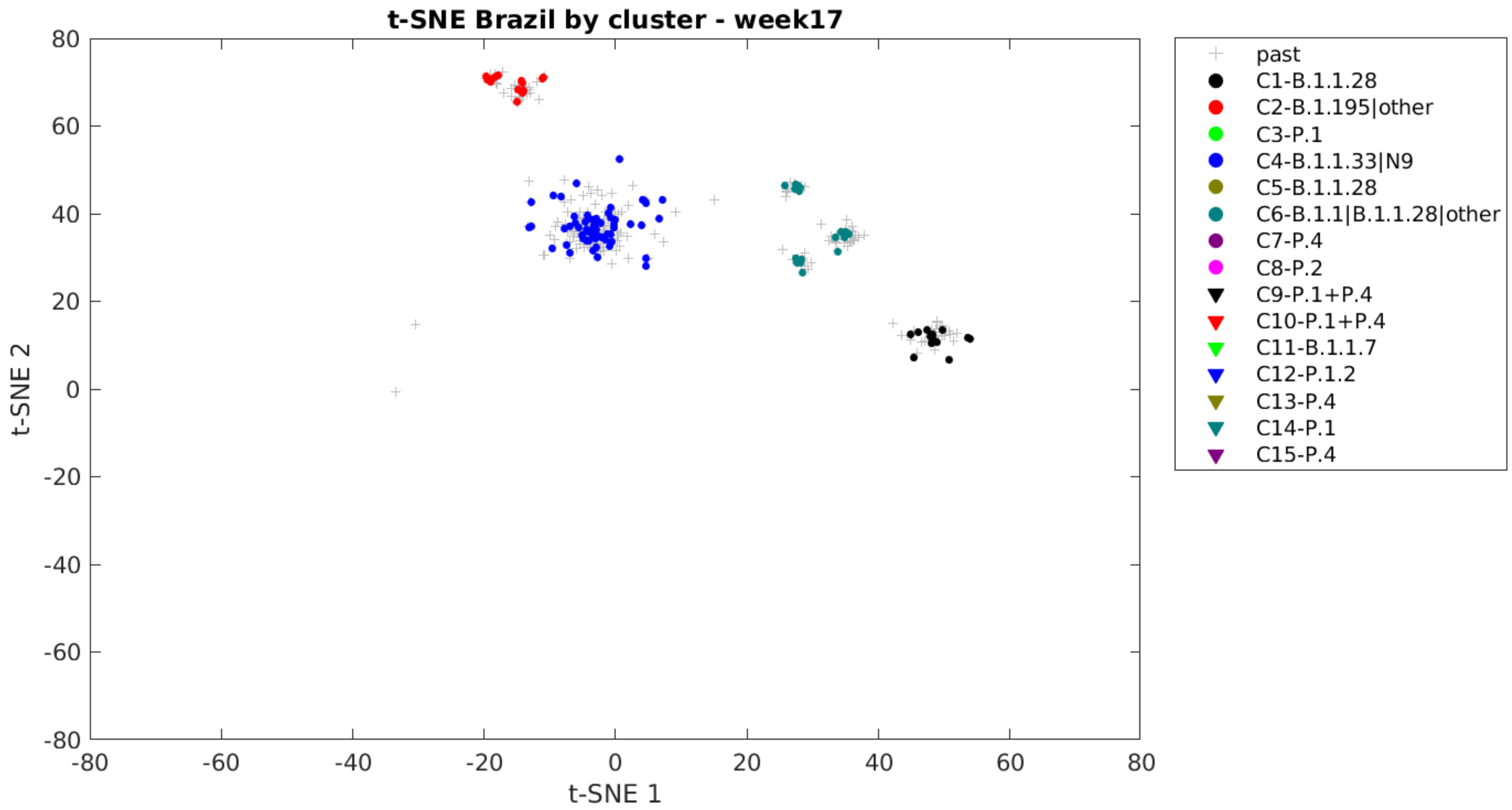
t-SNE Brazil by cluster - week17
t-SNE 1
t-SNE 2
80
60
40
20
0
-20
-40
-60
-80
-80
-60
-40
-20
0
20
40
60
80
past
C1-B.1.1.28
C2-B.1.195|other
C3-P.1
C4-B.1.1.33|N9
C5-B.1.1.28
C6-B.1.1|B.1.1.28|other
C7-P.4
C8-P.2
C9-P.1+P.4
C10-P.1+P.4
C11-B.1.1.7
C12-P.1.2
C13-P.4
C14-P.1
C15-P.4

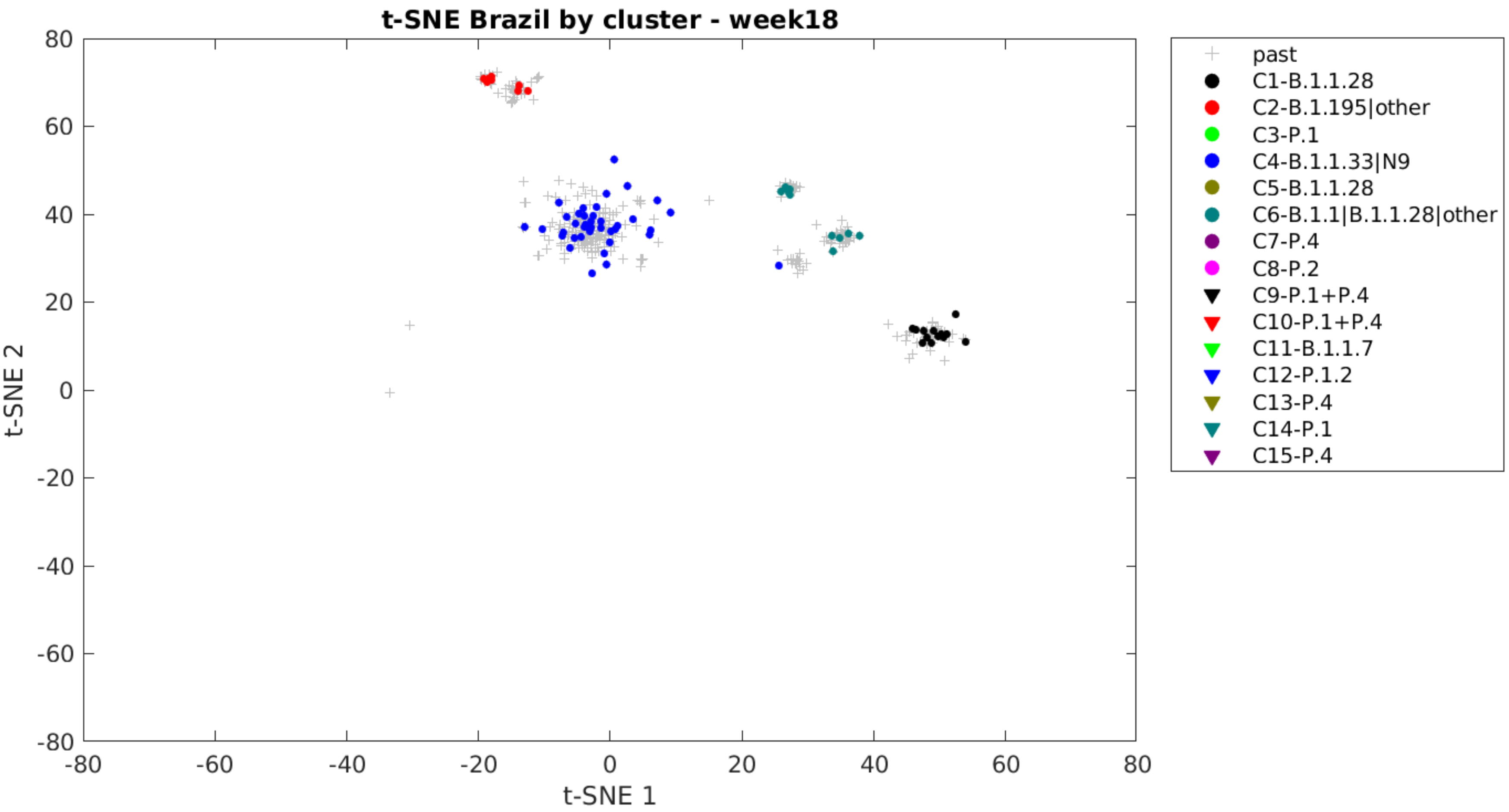

t-SNE Brazil by cluster - week18
t-SNE 2
t-SNE 1
80
60
40
20
0
-20
-40
-60
-80
-80
-60
-40
-20
0
20
40
60
80
past
C1-B.1.1.28
C2-B.1.195|other
C3-P.1
C4-B.1.1.33|N9
C5-B.1.1.28
C6-B.1.1|B.1.1.28|other
C7-P.4
C8-P.2
C9-P.1+P.4
C10-P.1+P.4
C11-B.1.1.7
C12-P.1.2
C13-P.4
C14-P.1
C15-P.4

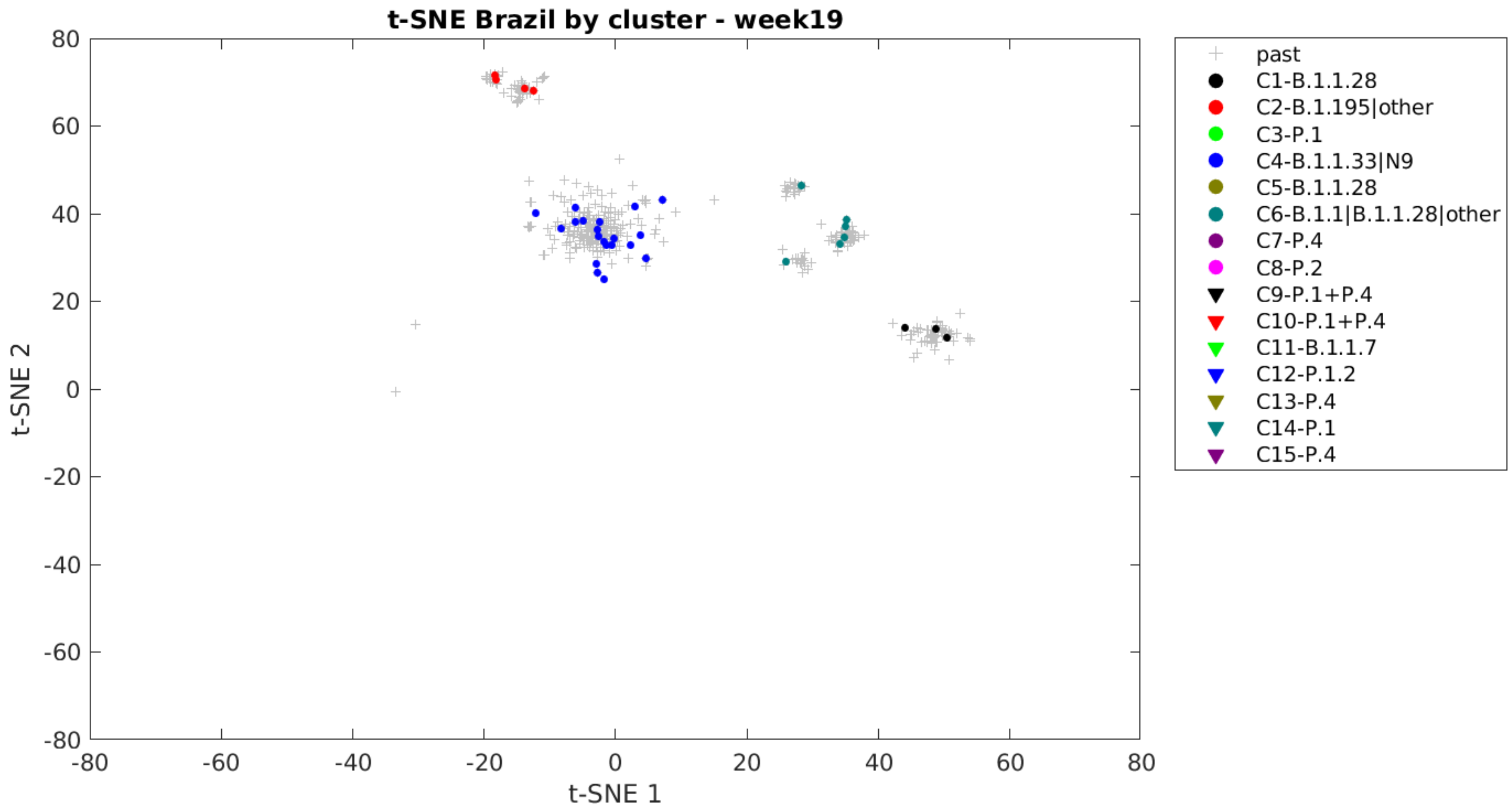
t-SNE Brazil by cluster - week19
t-SNE 1
t-SNE 2
80
60
40
20
0
-20
-40
-60
-80
-80
-60
-40
-20
0
20
40
60
80
past
C1-B.1.1.28
C2-B.1.195|other
C3-P.1
C4-B.1.1.33|N9
C5-B.1.1.28
C6-B.1.1|B.1.1.28|other
C7-P.4
C8-P.2
C9-P.1+P.4
C10-P.1+P.4
C11-B.1.1.7
C12-P.1.2
C13-P.4
C14-P.1
C15-P.4

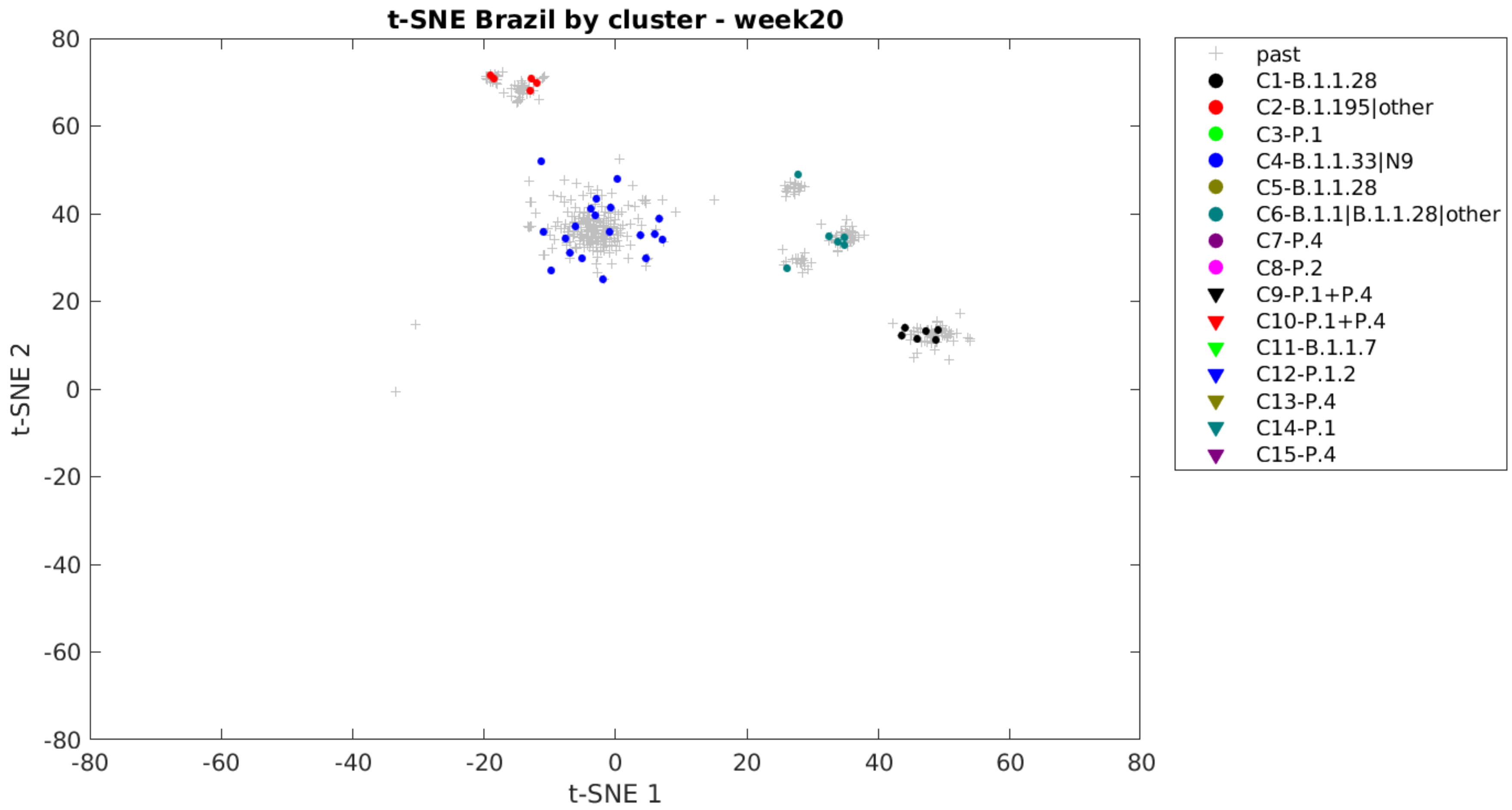
t-SNE Brazil by cluster - week20
t-SNE 1
t-SNE 2
80
60
40
20
0
-20
-40
-60
-80
-80
-60
-40
-20
0
20
40
60
80
past
C1-B.1.1.28
C2-B.1.195|other
C3-P.1
C4-B.1.1.33|N9
C5-B.1.1.28
C6-B.1.1|B.1.1.28|other
C7-P.4
C8-P.2
C9-P.1+P.4
C10-P.1+P.4
C11-B.1.1.7
C12-P.1.2
C13-P.4
C14-P.1
C15-P.4

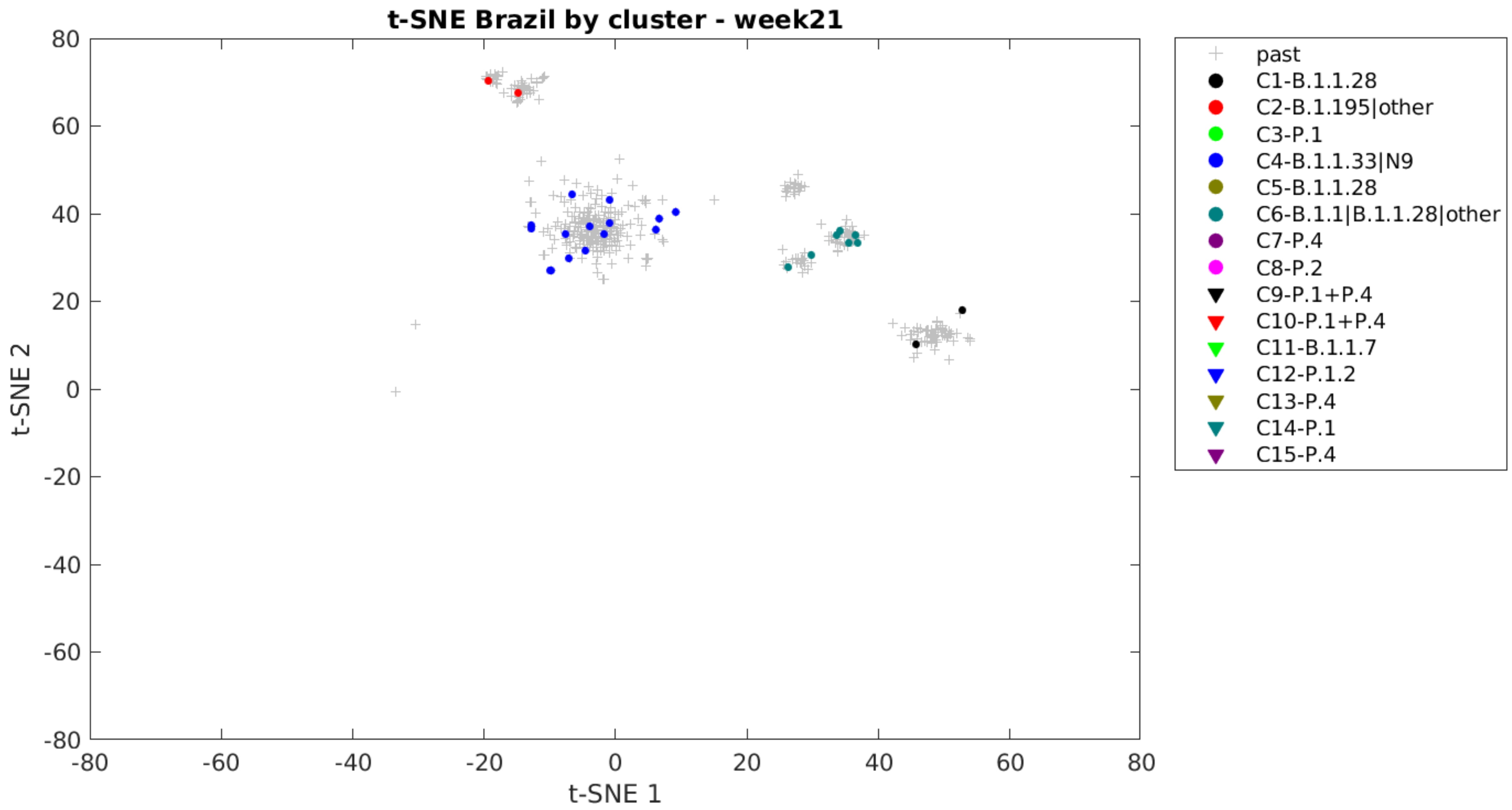

t-SNE Brazil by cluster - week21
t-SNE 1
t-SNE 2
80
60
40
20
0
-20
-40
-60
-80
past
C1-B.1.1.28
C2-B.1.195|other
C3-P.1
C4-B.1.1.33|N9
C5-B.1.1.28
C6-B.1.1|B.1.1.28|other
C7-P.4
C8-P.2
C9-P.1+P.4
C10-P.1+P.4
C11-B.1.1.7
C12-P.1.2
C13-P.4
C14-P.1
C15-P.4

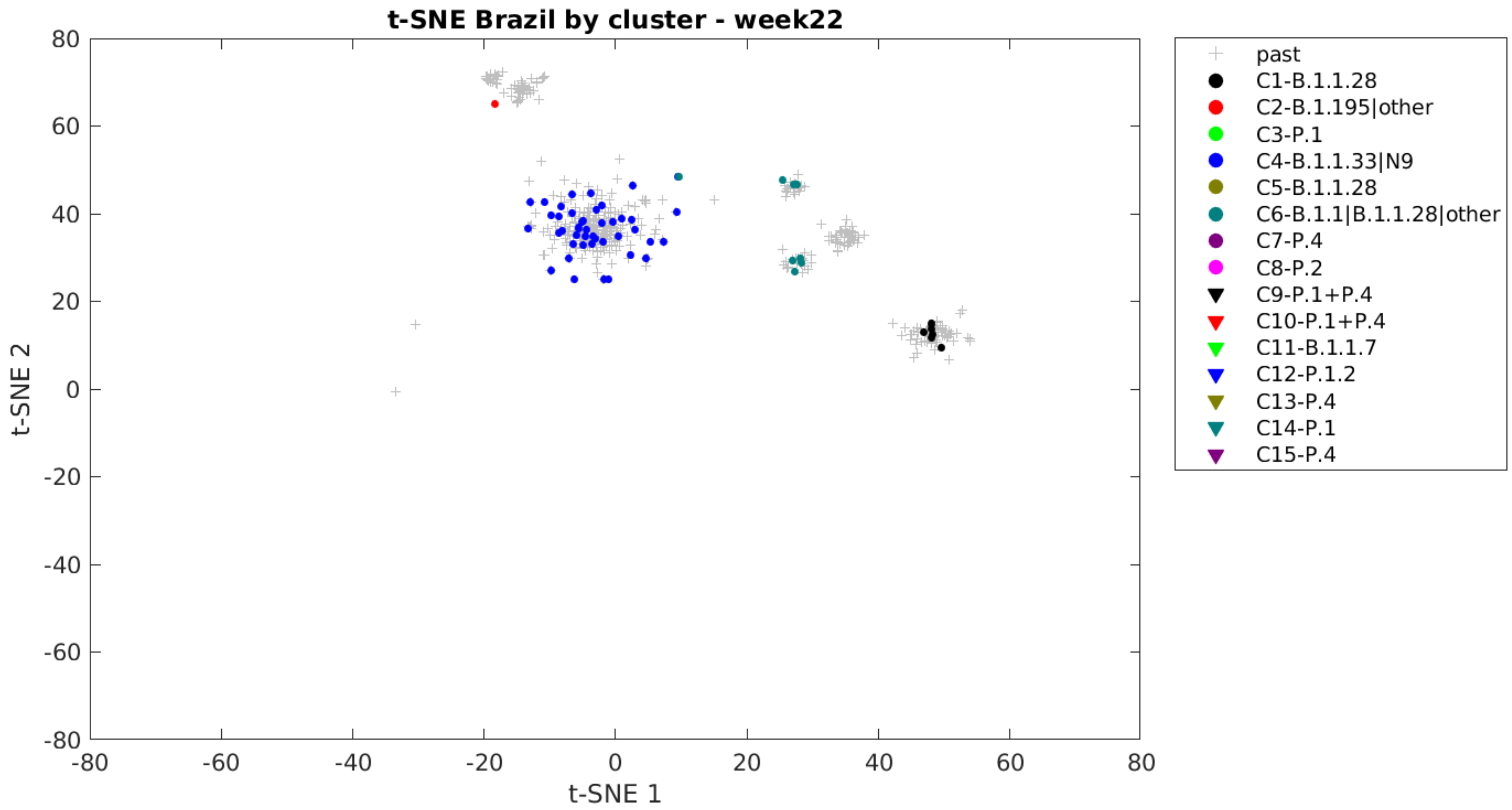
t-SNE Brazil by cluster - week22
t-SNE 1
t-SNE 2
80
60
40
20
0
-20
-40
-60
-80
-80
-60
-40
-20
0
20
40
60
80
past
C1-B.1.1.28
C2-B.1.195|other
C3-P.1
C4-B.1.1.33|N9
C5-B.1.1.28
C6-B.1.1|B.1.1.28|other
C7-P.4
C8-P.2
C9-P.1+P.4
C10-P.1+P.4
C11-B.1.1.7
C12-P.1.2
C13-P.4
C14-P.1
C15-P.4

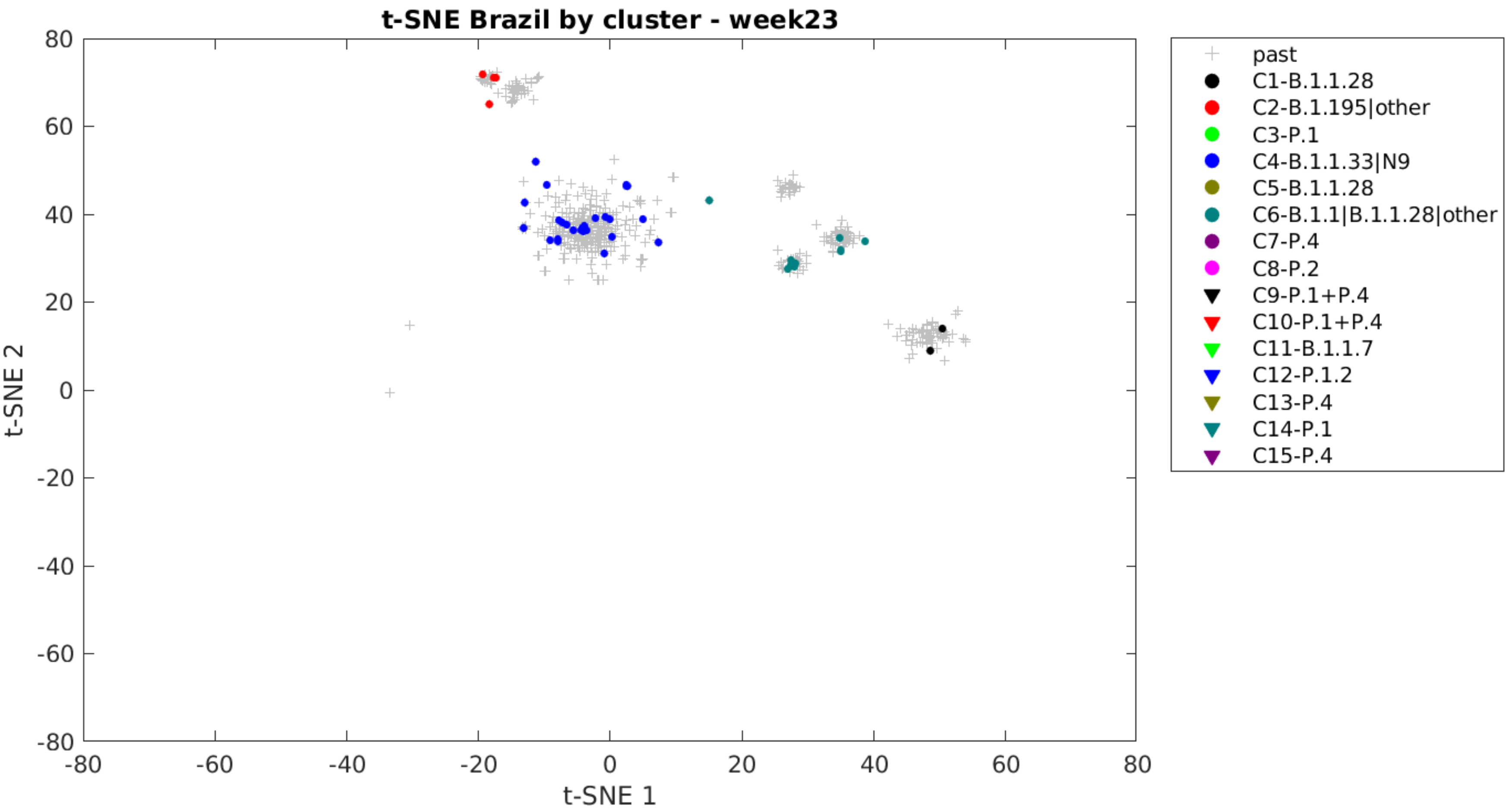
t-SNE Brazil by cluster - week23
80
60
40
20
0
-20
-40
-60
-80
-80
-60
-40
-20
0
20
40
60
80
t-SNE 1
t-SNE 2
past
C1-B.1.1.28
C2-B.1.195|other
C3-P.1
C4-B.1.1.33|N9
C5-B.1.1.28
C6-B.1.1|B.1.1.28|other
C7-P.4
C8-P.2
C9-P.1+P.4
C10-P.1+P.4
C11-B.1.1.7
C12-P.1.2
C13-P.4
C14-P.1
C15-P.4

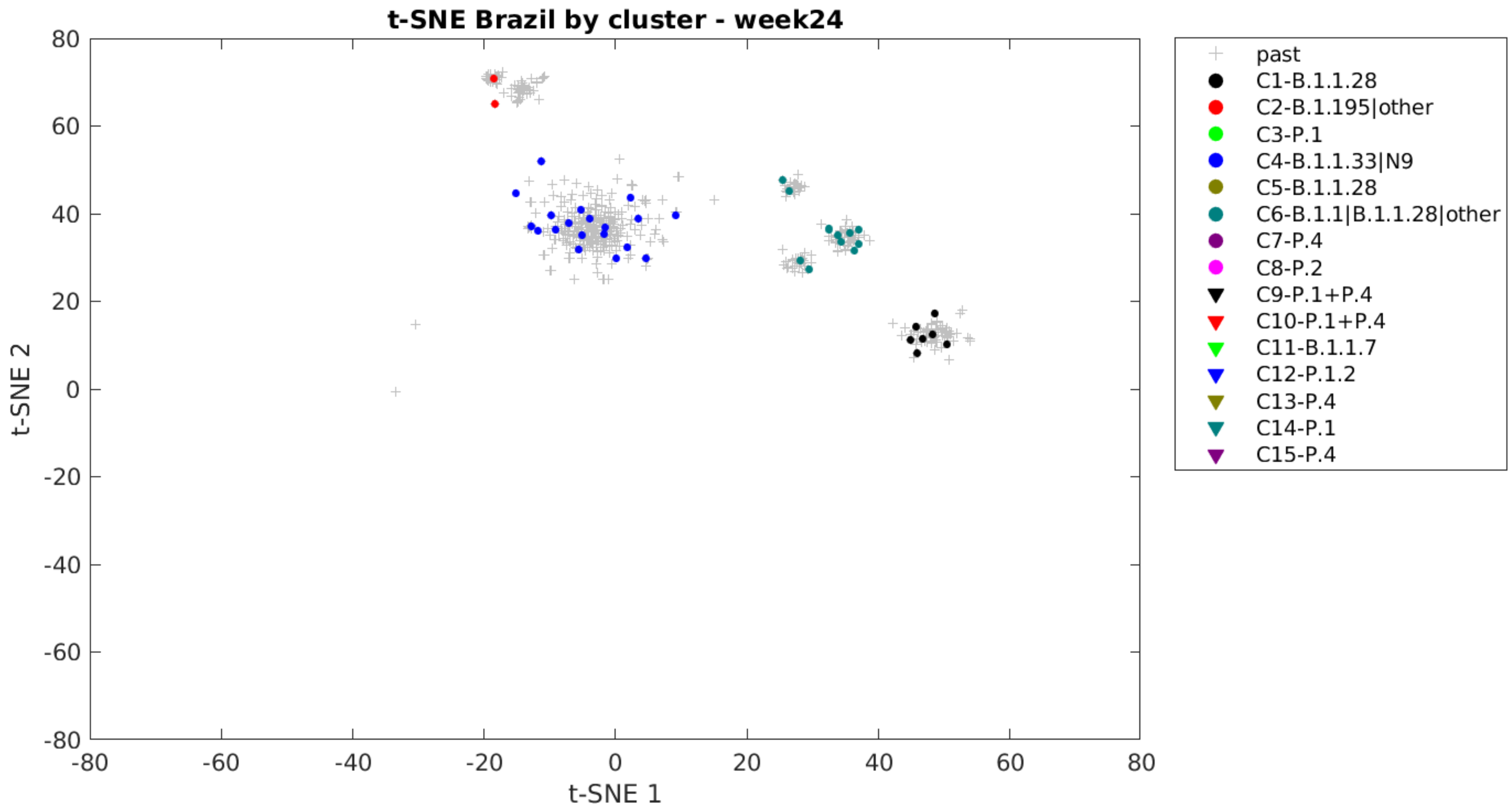
t-SNE Brazil by cluster - week24
t-SNE 2
t-SNE 1
80
60
40
20
0
-20
-40
-60
-80
-80
-60
-40
-20
0
20
40
60
80
past
C1-B.1.1.28
C2-B.1.195|other
C3-P.1
C4-B.1.1.33|N9
C5-B.1.1.28
C6-B.1.1|B.1.1.28|other
C7-P.4
C8-P.2
C9-P.1+P.4
C10-P.1+P.4
C11-B.1.1.7
C12-P.1.2
C13-P.4
C14-P.1
C15-P.4

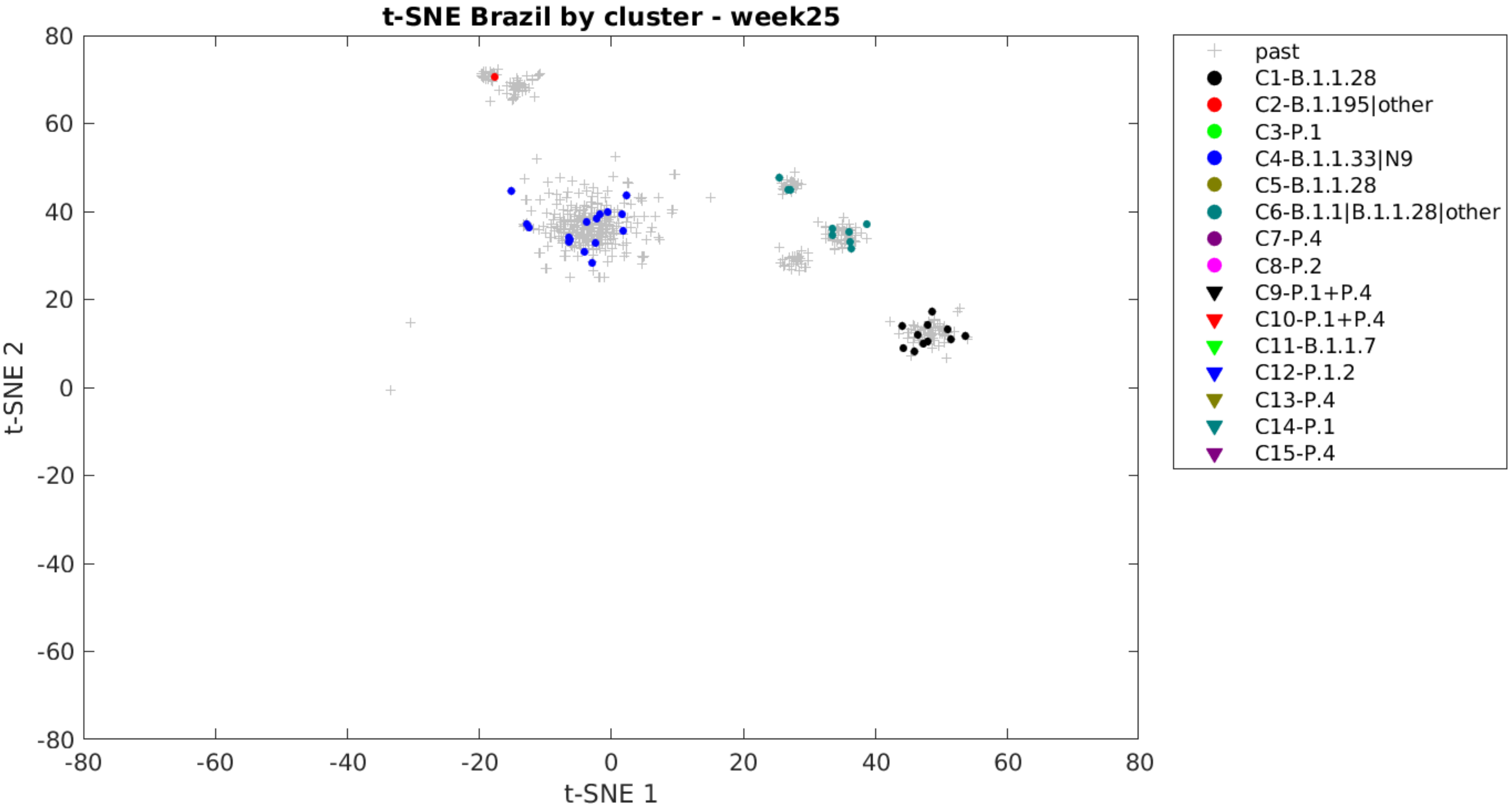

t-SNE Brazil by cluster - week25
80
60
40
20
0
-20
-40
-60
-80
-80
-60
-40
-20
0
20
40
60
80
t-SNE 2
t-SNE 1
past
C1-B.1.1.28
C2-B.1.195|other
C3-P.1
C4-B.1.1.33|N9
C5-B.1.1.28
C6-B.1.1|B.1.1.28|other
C7-P.4
C8-P.2
C9-P.1+P.4
C10-P.1+P.4
C11-B.1.1.7
C12-P.1.2
C13-P.4
C14-P.1
C15-P.4

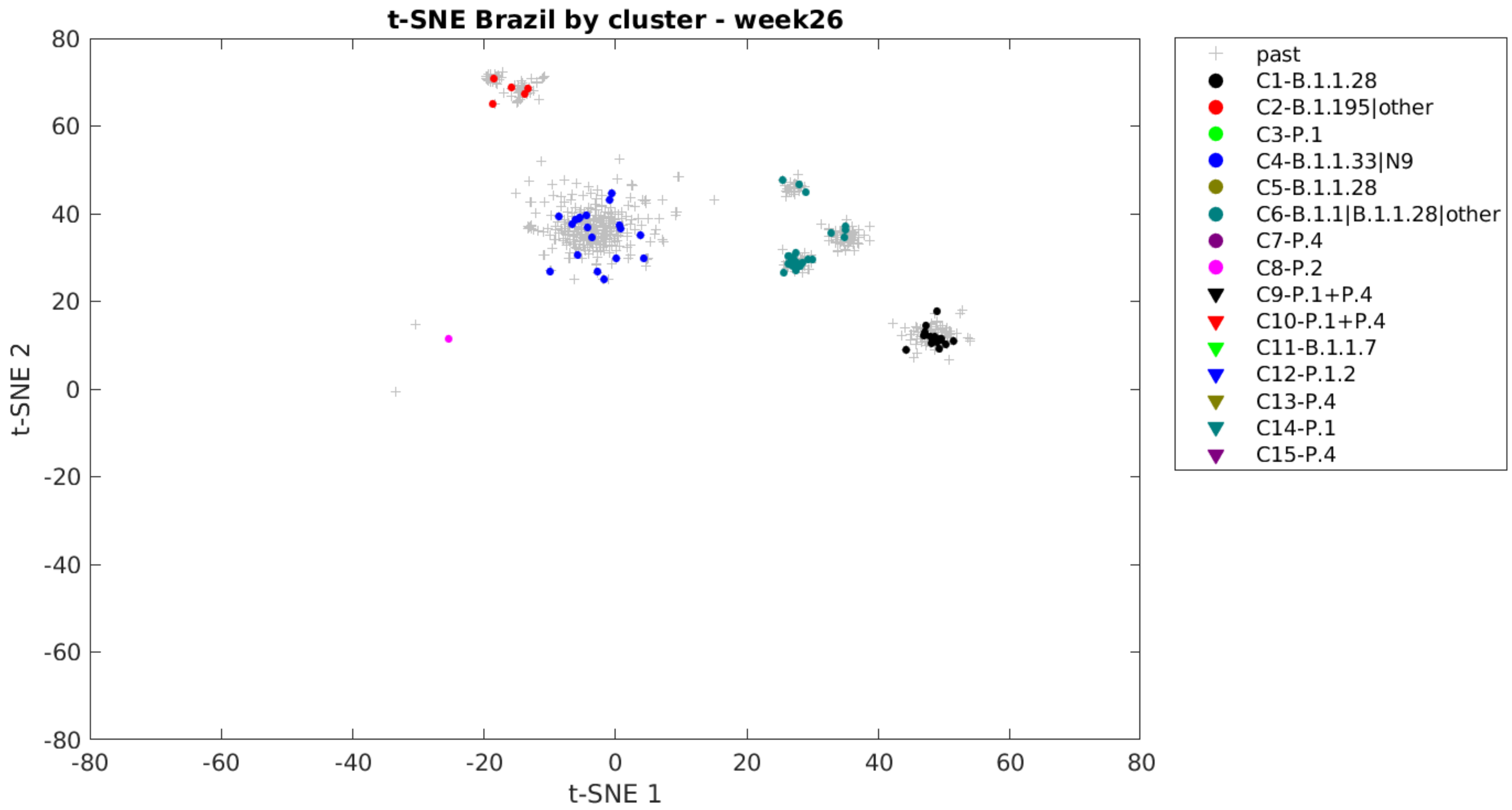

t-SNE Brazil by cluster - week26
t-SNE 2
t-SNE 1
80
60
40
20
0
-20
-40
-60
-80
past
C1-B.1.1.28
C2-B.1.195|other
C3-P.1
C4-B.1.1.33|N9
C5-B.1.1.28
C6-B.1.1|B.1.1.28|other
C7-P.4
C8-P.2
C9-P.1+P.4
C10-P.1+P.4
C11-B.1.1.7
C12-P.1.2
C13-P.4
C14-P.1
C15-P.4

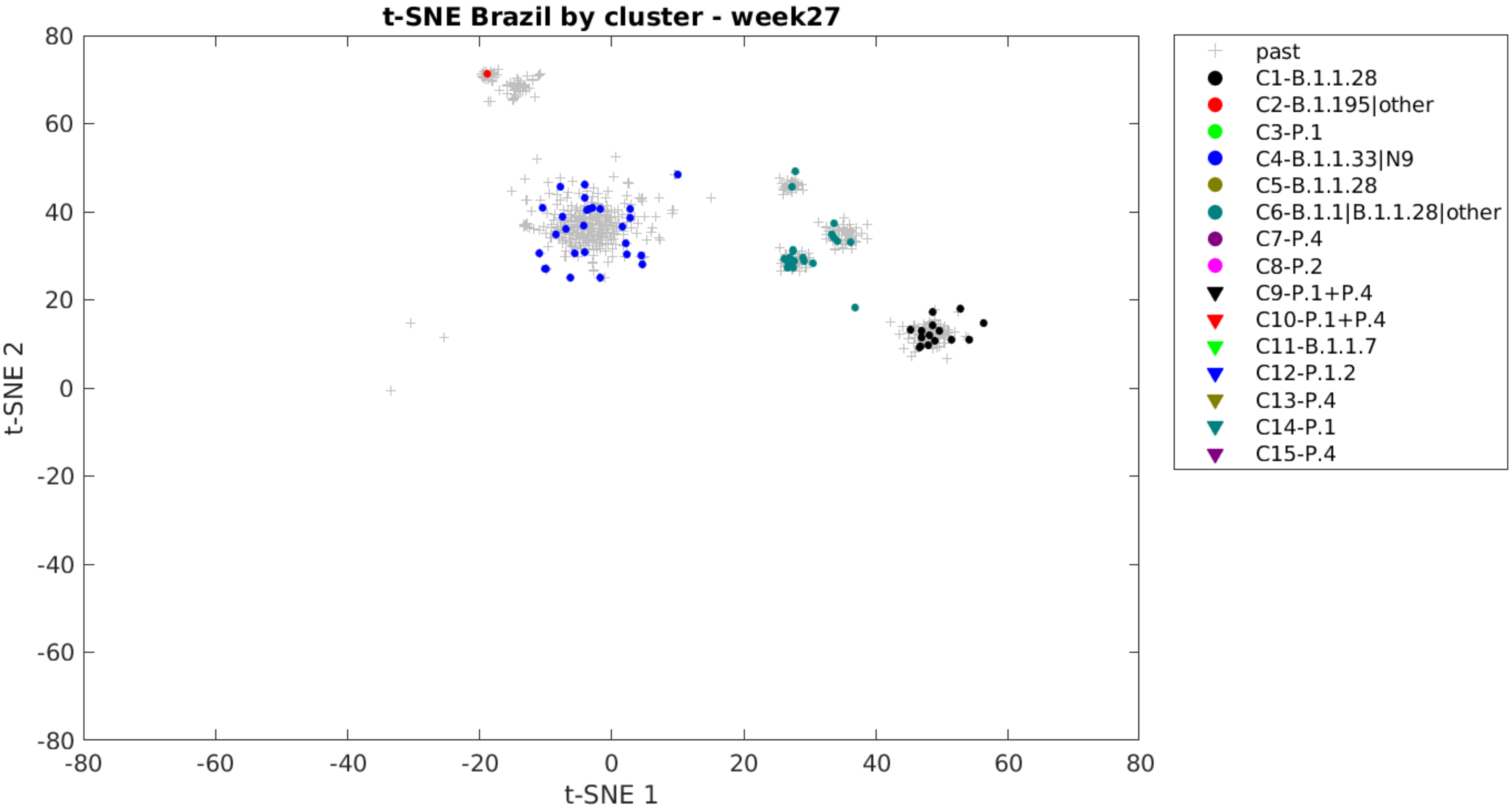
t-SNE Brazil by cluster - week27
t-SNE 2
t-SNE 1
80
60
40
20
0
-20
-40
-60
-80
-80
-60
-40
-20
0
20
40
60
80
past
C1-B.1.1.28
C2-B.1.195|other
C3-P.1
C4-B.1.1.33|N9
C5-B.1.1.28
C6-B.1.1|B.1.1.28|other
C7-P.4
C8-P.2
C9-P.1+P.4
C10-P.1+P.4
C11-B.1.1.7
C12-P.1.2
C13-P.4
C14-P.1
C15-P.4

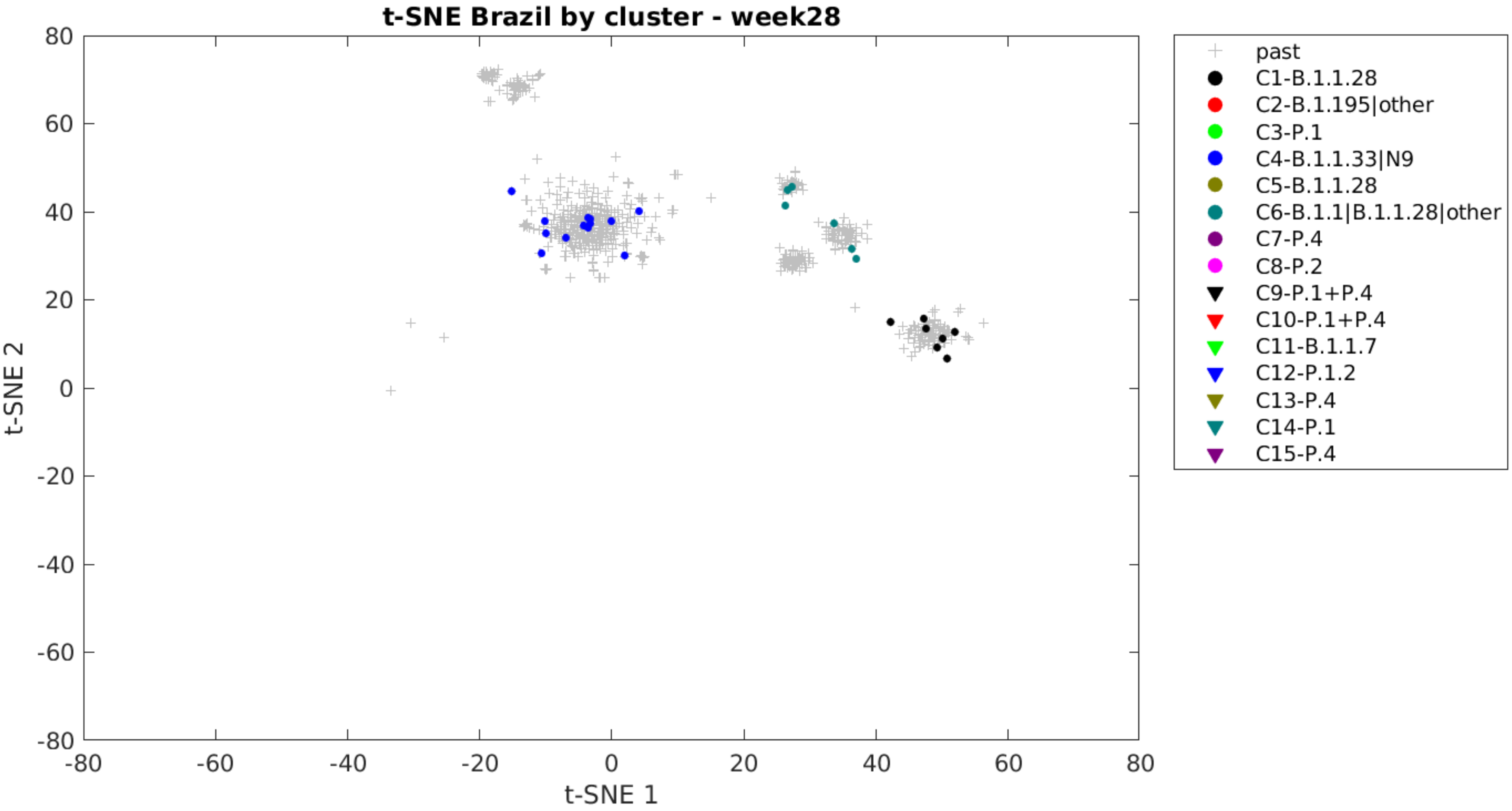

t-SNE Brazil by cluster - week28
80
60
40
20
0
-20
-40
-60
-80
t-SNE 2
-80
-60
-40
-20
0
20
40
60
80
t-SNE 1
past
C1-B.1.1.28
C2-B.1.195|other
C3-P.1
C4-B.1.1.33|N9
C5-B.1.1.28
C6-B.1.1|B.1.1.28|other
C7-P.4
C8-P.2
C9-P.1+P.4
C10-P.1+P.4
C11-B.1.1.7
C12-P.1.2
C13-P.4
C14-P.1
C15-P.4

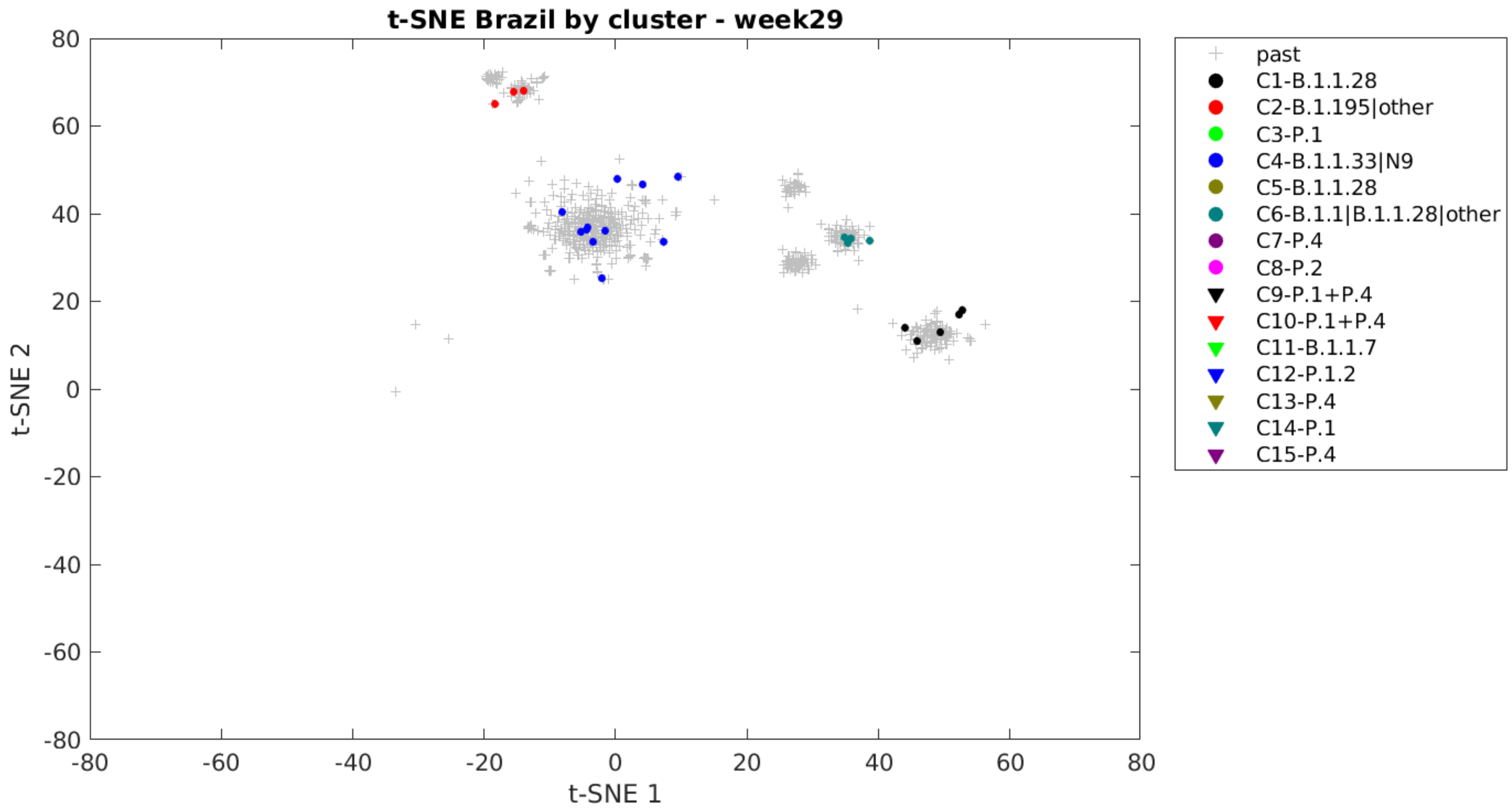
t-SNE Brazil by cluster - week29
t-SNE 2
t-SNE 1
80
60
40
20
0
-20
-40
-60
-80
-80
-60
-40
-20
0
20
40
60
80
past
C1-B.1.1.28
C2-B.1.195|other
C3-P.1
C4-B.1.1.33|N9
C5-B.1.1.28
C6-B.1.1|B.1.1.28|other
C7-P.4
C8-P.2
C9-P.1+P.4
C10-P.1+P.4
C11-B.1.1.7
C12-P.1.2
C13-P.4
C14-P.1
C15-P.4

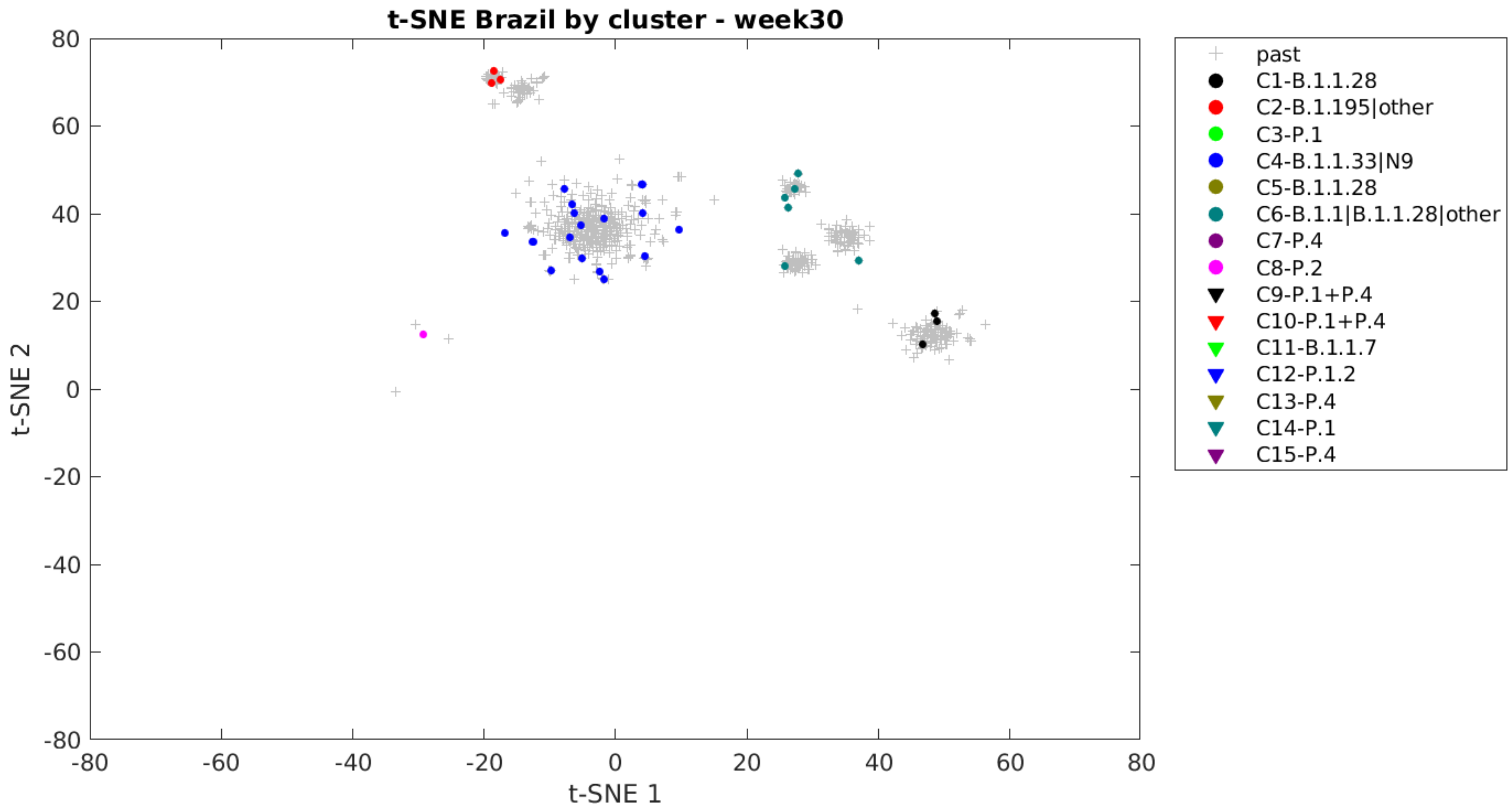

t-SNE Brazil by cluster - week30
t-SNE 2
t-SNE 1
80
60
40
20
0
-20
-40
-60
-80
-80
-60
-40
-20
0
20
40
60
80
past
C1-B.1.1.28
C2-B.1.195|other
C3-P.1
C4-B.1.1.33|N9
C5-B.1.1.28
C6-B.1.1|B.1.1.28|other
C7-P.4
C8-P.2
C9-P.1+P.4
C10-P.1+P.4
C11-B.1.1.7
C12-P.1.2
C13-P.4
C14-P.1
C15-P.4

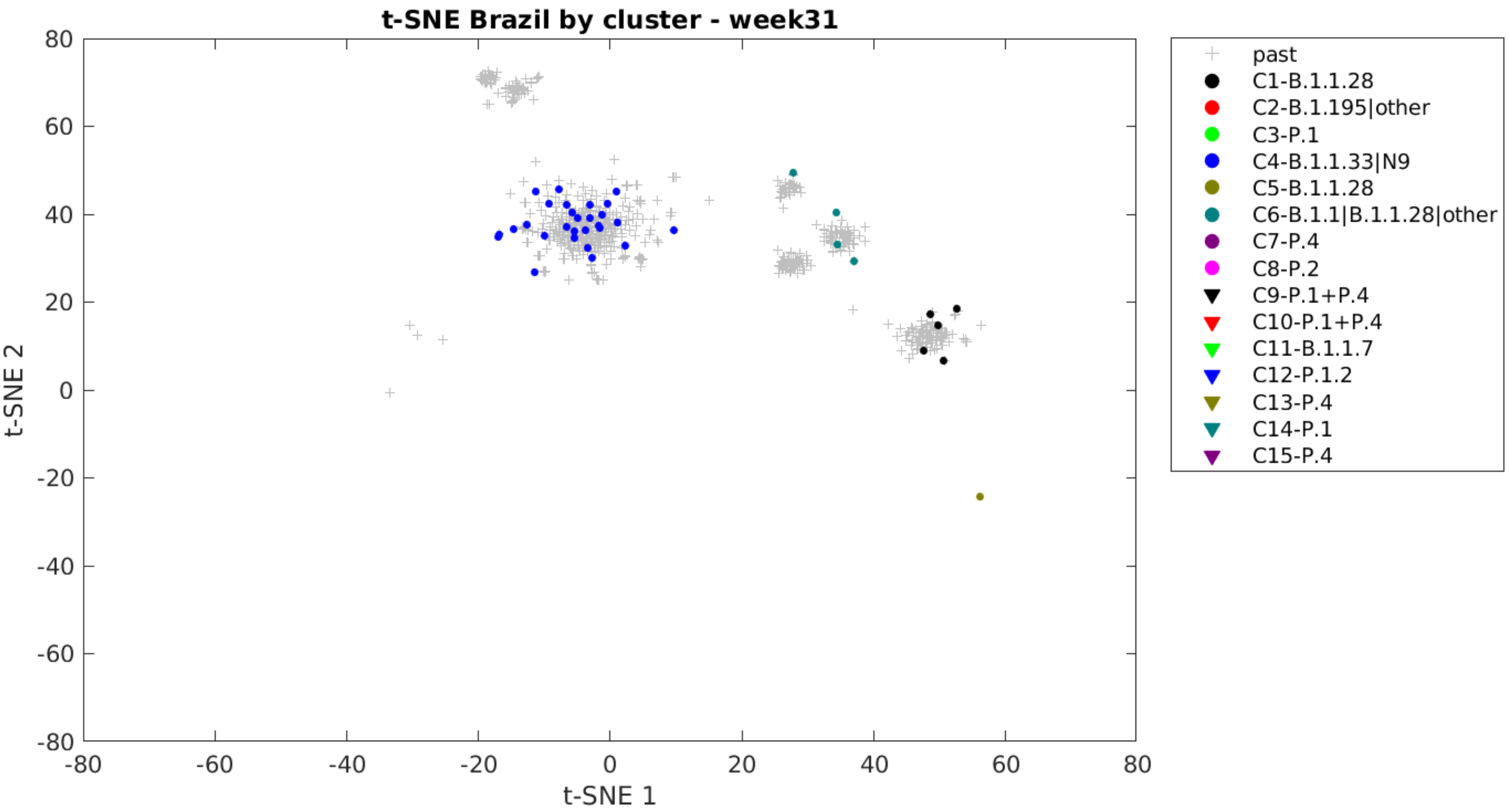
t-SNE Brazil by cluster - week31
t-SNE 1
t-SNE 2
80
60
40
20
0
-20
-40
-60
-80
past
C1-B.1.1.28
C2-B.1.195|other
C3-P.1
C4-B.1.1.33|N9
C5-B.1.1.28
C6-B.1.1|B.1.1.28|other
C7-P.4
C8-P.2
C9-P.1+P.4
C10-P.1+P.4
C11-B.1.1.7
C12-P.1.2
C13-P.4
C14-P.1
C15-P.4

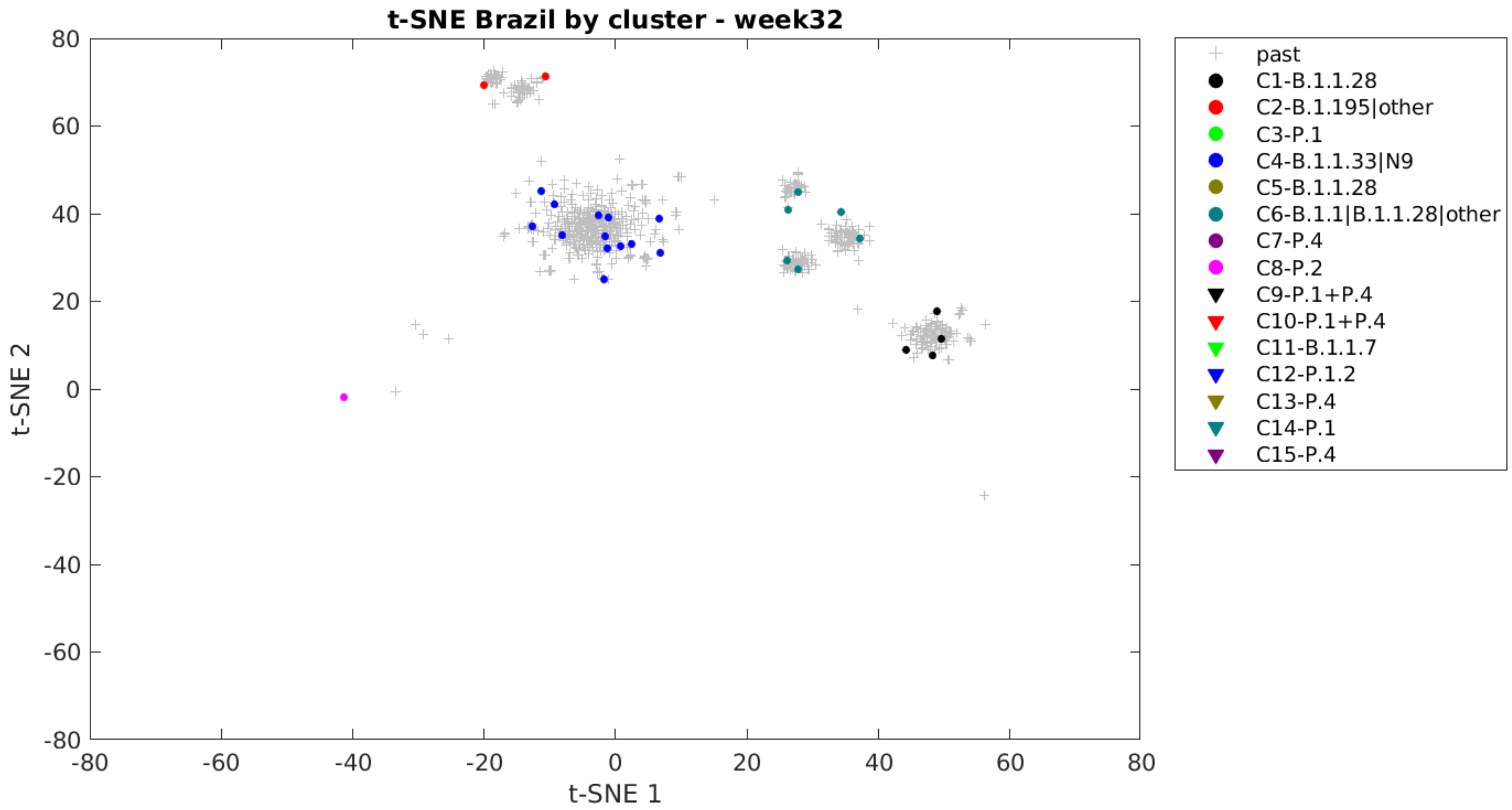

t-SNE Brazil by cluster - week32
80
60
40
20
0
-20
-40
-60
-80
t-SNE 2
-80
-60
-40
-20
0
20
40
60
80
t-SNE 1
past
C1-B.1.1.28
C2-B.1.195|other
C3-P.1
C4-B.1.1.33|N9
C5-B.1.1.28
C6-B.1.1|B.1.1.28|other
C7-P.4
C8-P.2
C9-P.1+P.4
C10-P.1+P.4
C11-B.1.1.7
C12-P.1.2
C13-P.4
C14-P.1
C15-P.4

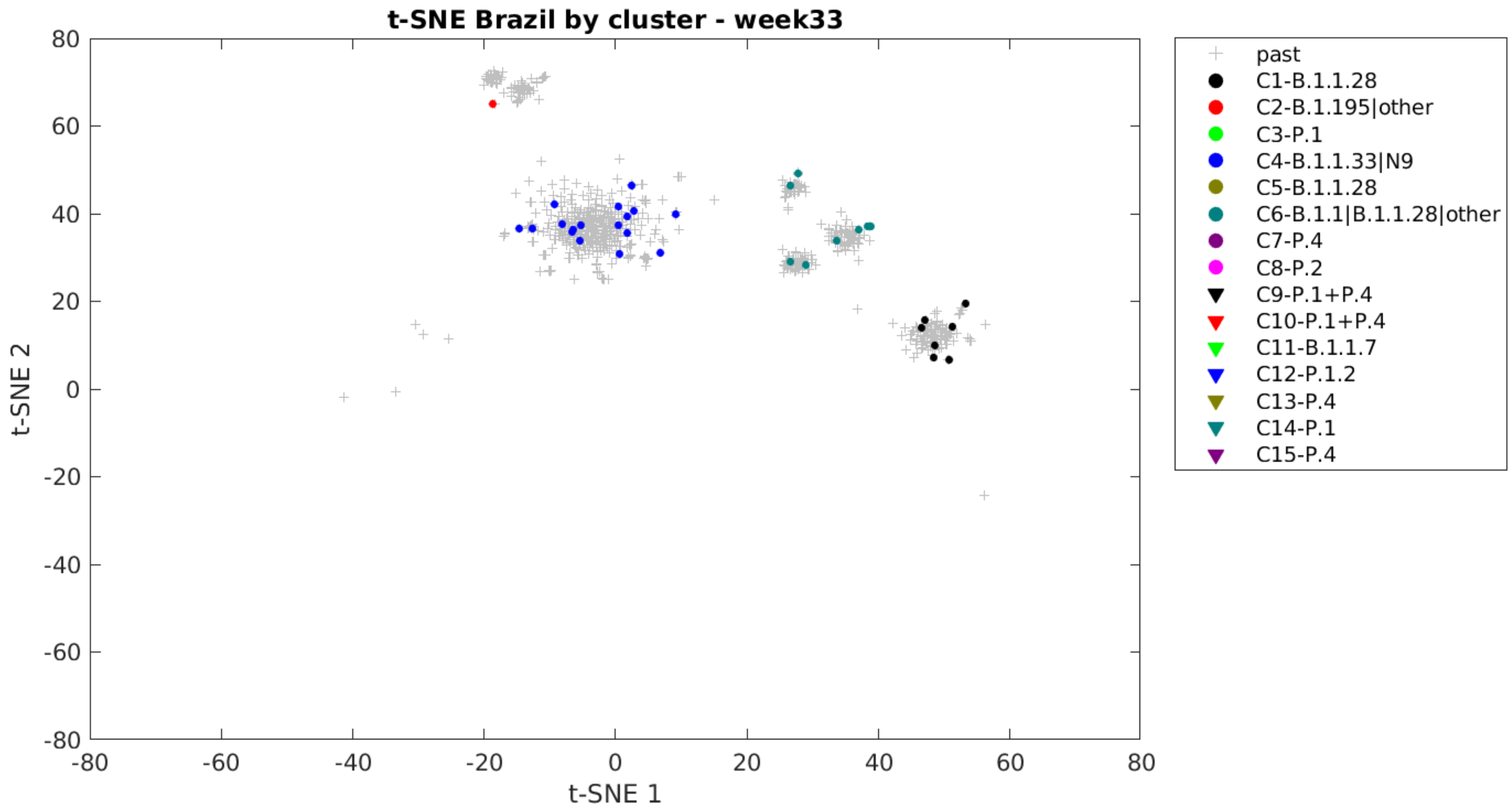
t-SNE Brazil by cluster - week33
t-SNE 1
t-SNE 2
past
C1-B.1.1.28
C2-B.1.195|other
C3-P.1
C4-B.1.1.33|N9
C5-B.1.1.28
C6-B.1.1|B.1.1.28|other
C7-P.4
C8-P.2
C9-P.1+P.4
C10-P.1+P.4
C11-B.1.1.7
C12-P.1.2
C13-P.4
C14-P.1
C15-P.4

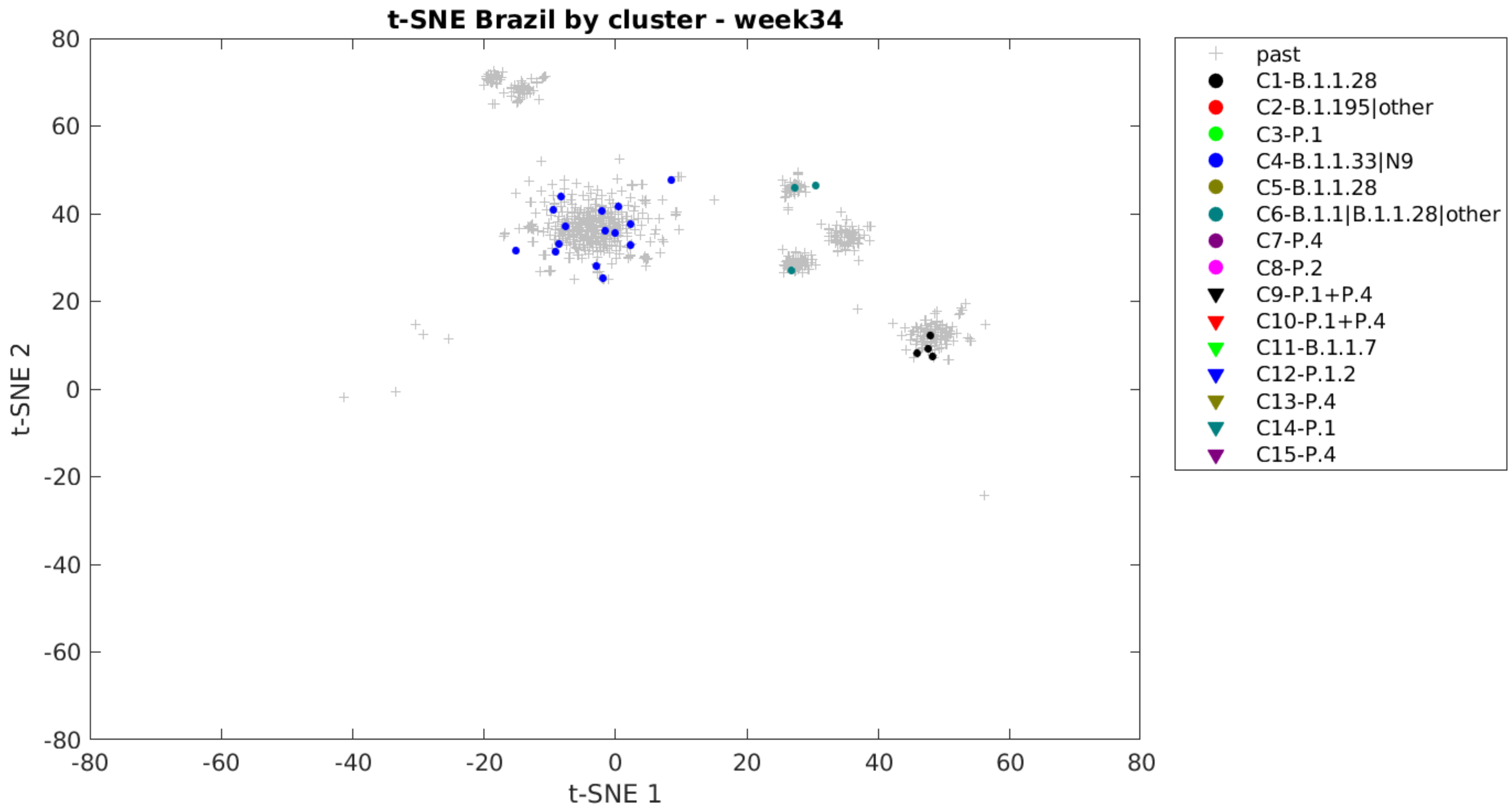

t-SNE Brazil by cluster - week34
80
60
40
20
0
-20
-40
-60
-80
t-SNE 2
-80
-60
-40
-20
0
20
40
60
80
t-SNE 1
past
C1-B.1.1.28
C2-B.1.195|other
C3-P.1
C4-B.1.1.33|N9
C5-B.1.1.28
C6-B.1.1|B.1.1.28|other
C7-P.4
C8-P.2
C9-P.1+P.4
C10-P.1+P.4
C11-B.1.1.7
C12-P.1.2
C13-P.4
C14-P.1
C15-P.4

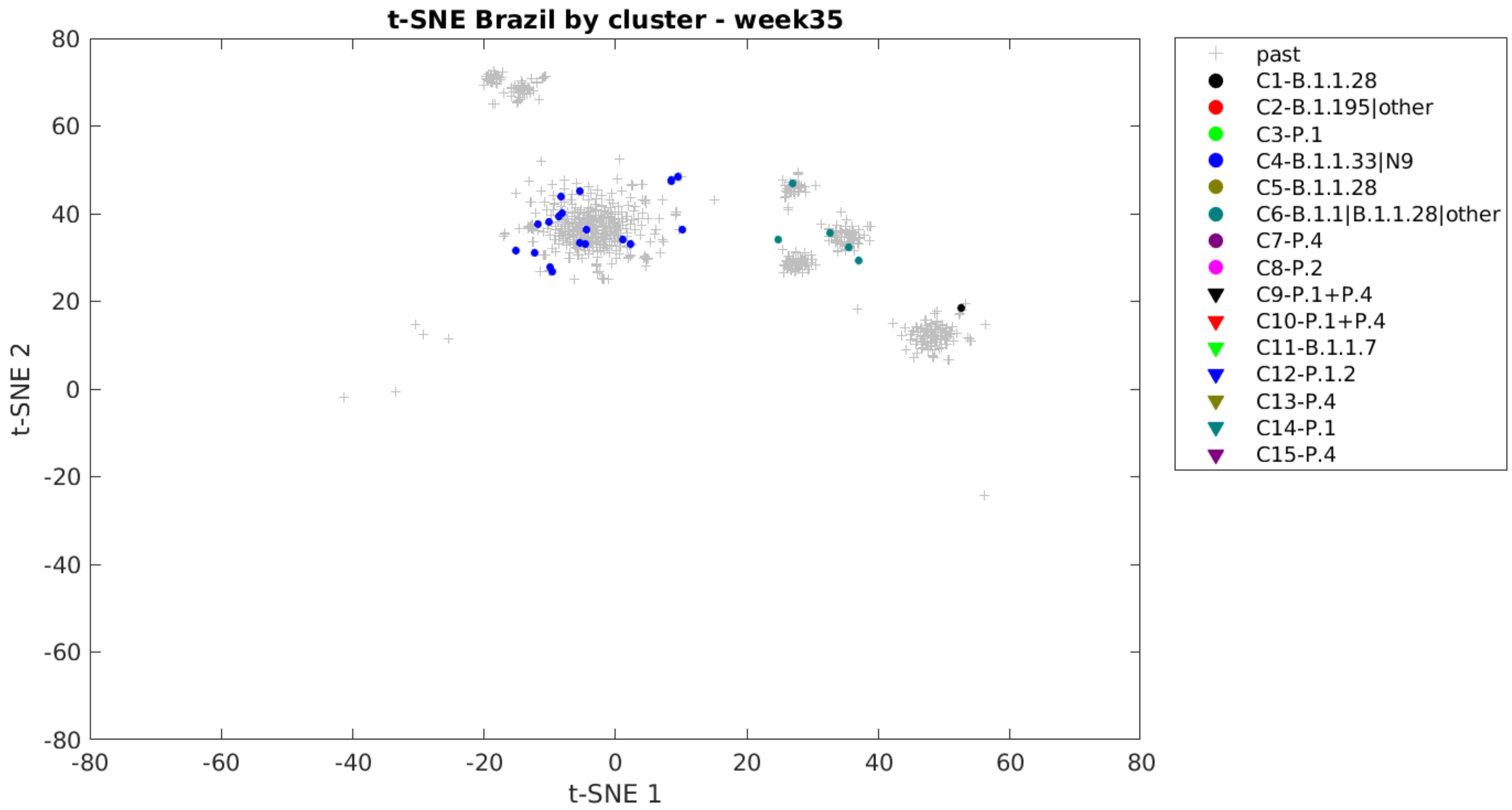
t-SNE Brazil by cluster - week35
t-SNE 2
t-SNE 1
80
60
40
20
0
-20
-40
-60
-80
-80
-60
-40
-20
0
20
40
60
80
past
C1-B.1.1.28
C2-B.1.195|other
C3-P.1
C4-B.1.1.33|N9
C5-B.1.1.28
C6-B.1.1|B.1.1.28|other
C7-P.4
C8-P.2
C9-P.1+P.4
C10-P.1+P.4
C11-B.1.1.7
C12-P.1.2
C13-P.4
C14-P.1
C15-P.4

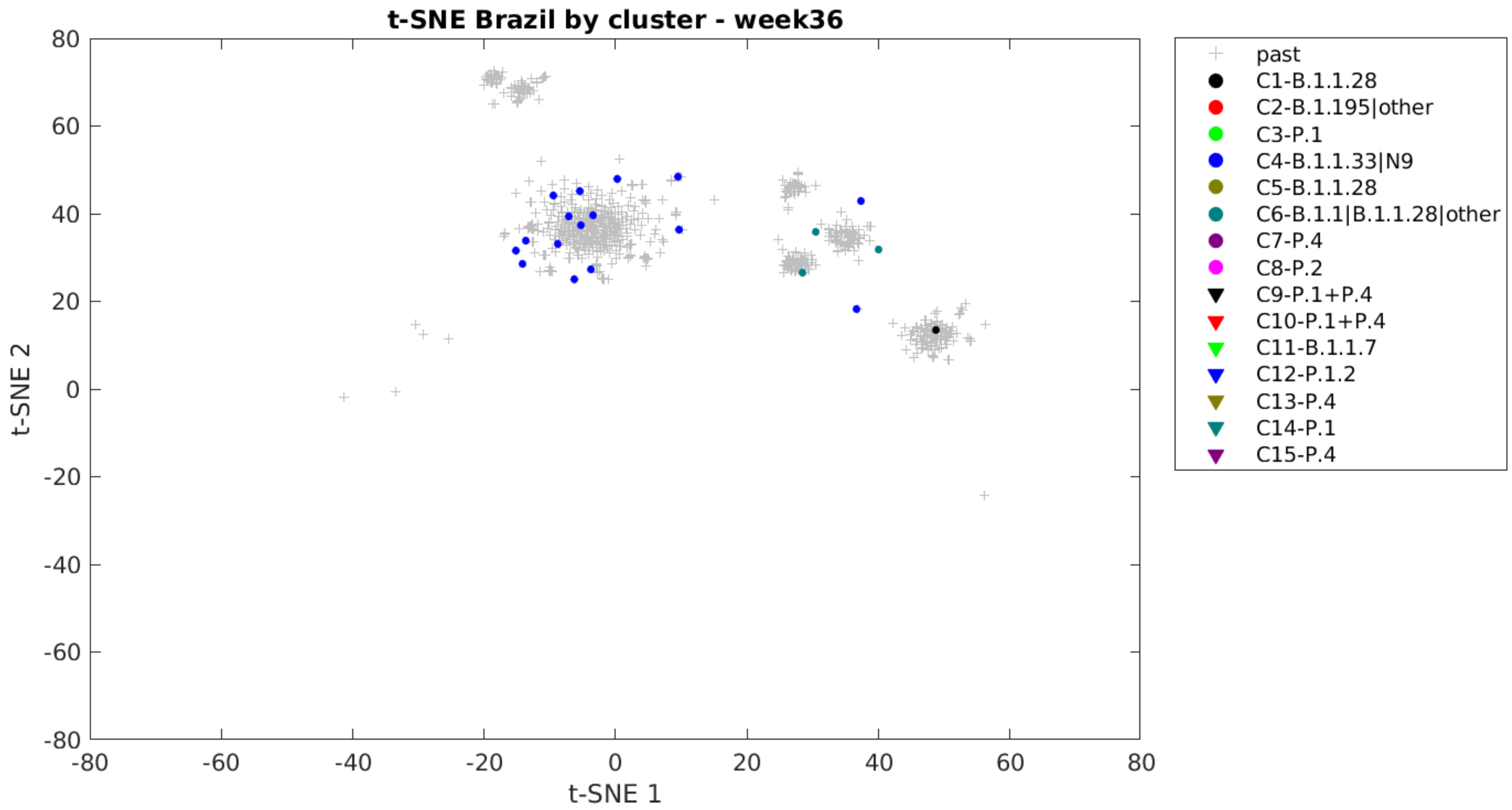
t-SNE Brazil by cluster - week36
t-SNE 1
t-SNE 2
80
60
40
20
0
-20
-40
-60
-80
-80
-60
-40
-20
0
20
40
60
80
past
C1-B.1.1.28
C2-B.1.195|other
C3-P.1
C4-B.1.1.33|N9
C5-B.1.1.28
C6-B.1.1|B.1.1.28|other
C7-P.4
C8-P.2
C9-P.1+P.4
C10-P.1+P.4
C11-B.1.1.7
C12-P.1.2
C13-P.4
C14-P.1
C15-P.4

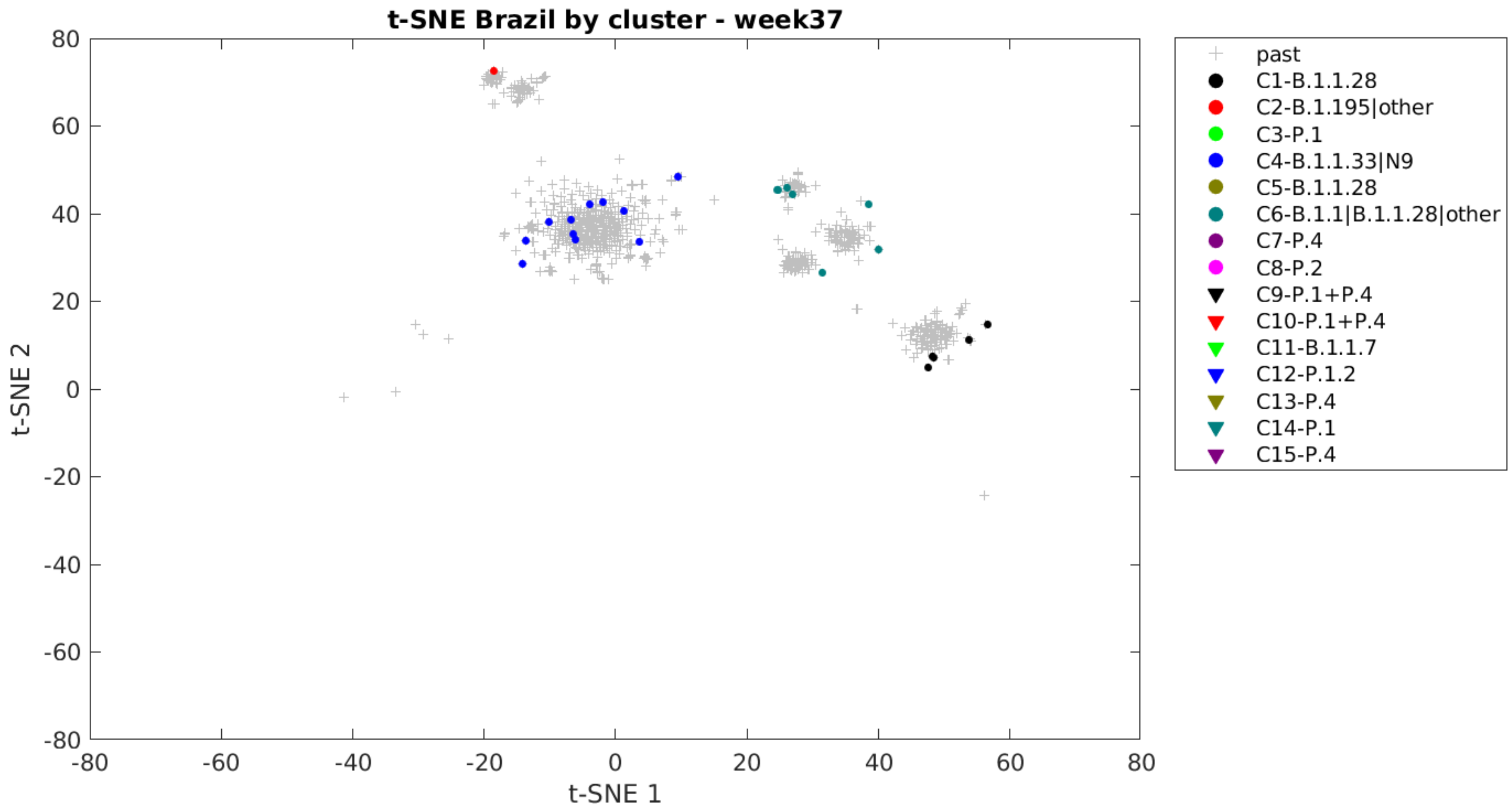

t-SNE Brazil by cluster - week37
t-SNE 2
t-SNE 1
80
60
40
20
0
-20
-40
-60
-80
-80
-60
-40
-20
0
20
40
60
80
past
C1-B.1.1.28
C2-B.1.195|other
C3-P.1
C4-B.1.1.33|N9
C5-B.1.1.28
C6-B.1.1|B.1.1.28|other
C7-P.4
C8-P.2
C9-P.1+P.4
C10-P.1+P.4
C11-B.1.1.7
C12-P.1.2
C13-P.4
C14-P.1
C15-P.4

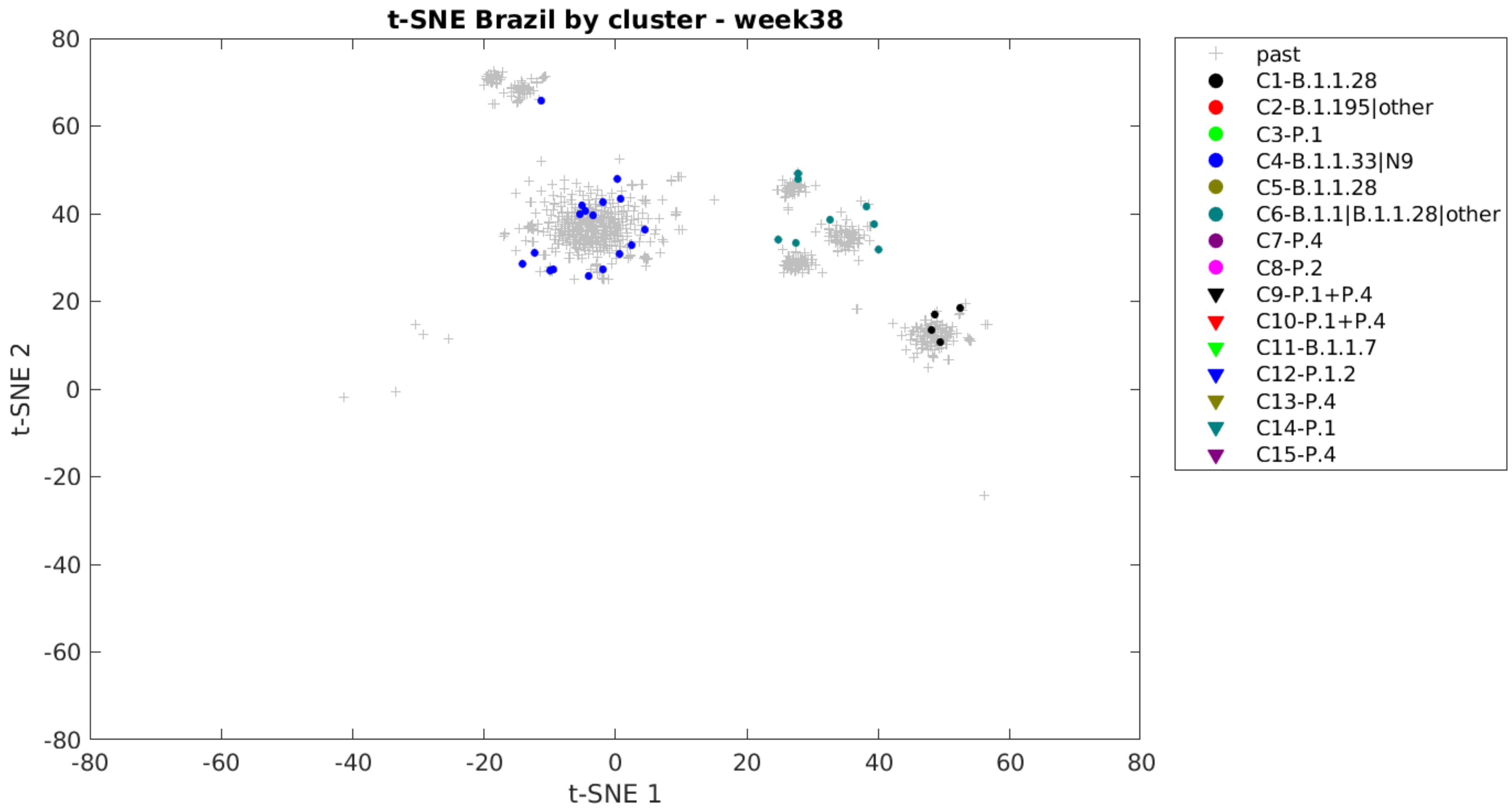
t-SNE Brazil by cluster - week38
t-SNE 1
t-SNE 2
-80
-60
-40
-20
0
20
40
60
80
past
C1-B.1.1.28
C2-B.1.195|other
C3-P.1
C4-B.1.1.33|N9
C5-B.1.1.28
C6-B.1.1|B.1.1.28|other
C7-P.4
C8-P.2
C9-P.1+P.4
C10-P.1+P.4
C11-B.1.1.7
C12-P.1.2
C13-P.4
C14-P.1
C15-P.4

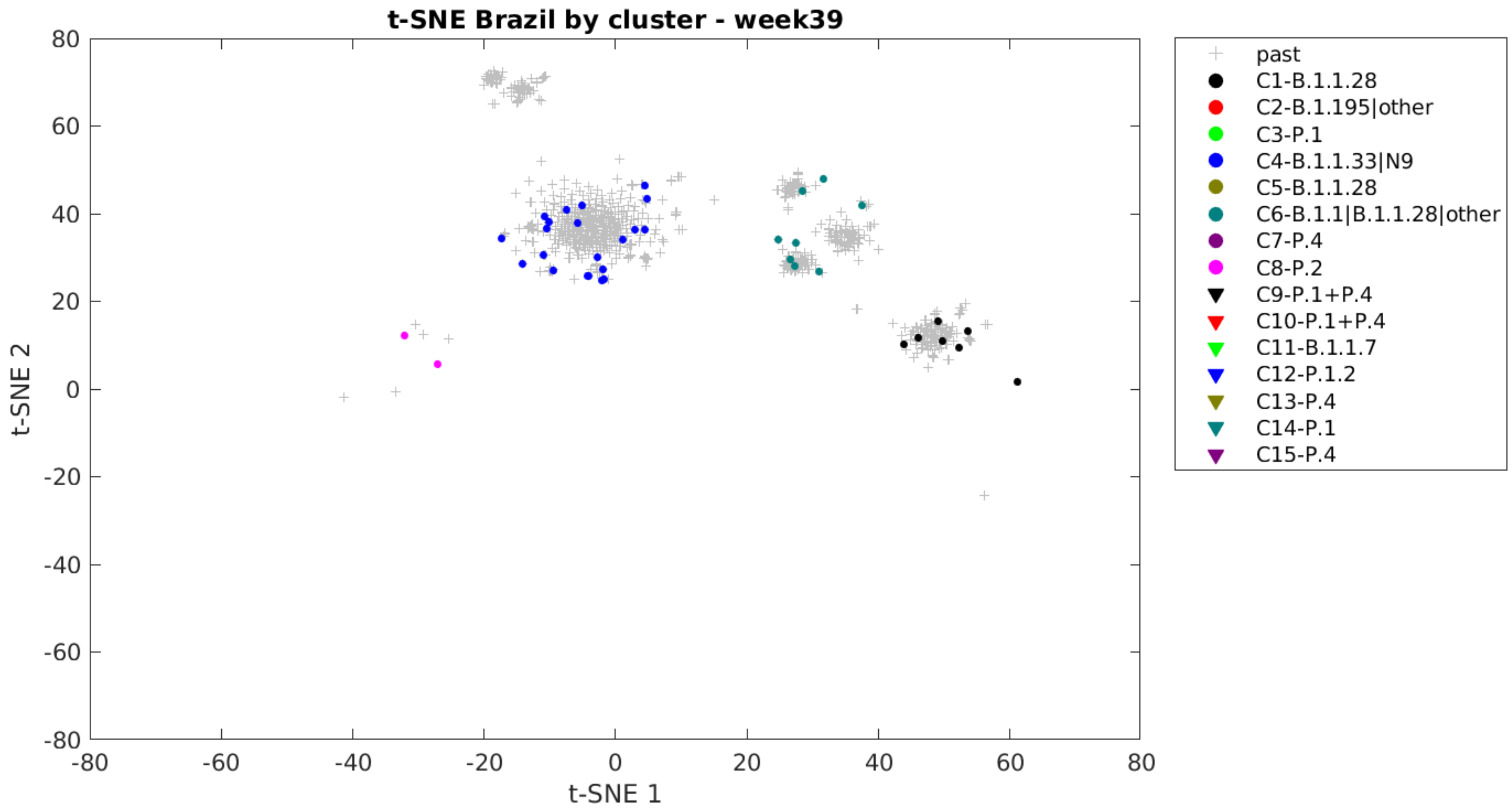

t-SNE Brazil by cluster - week39
80
60
40
20
0
-20
-40
-60
-80
-80
-60
-40
-20
0
20
40
60
80
t-SNE 1
t-SNE 2
past
C1-B.1.1.28
C2-B.1.195|other
C3-P.1
C4-B.1.1.33|N9
C5-B.1.1.28
C6-B.1.1|B.1.1.28|other
C7-P.4
C8-P.2
C9-P.1+P.4
C10-P.1+P.4
C11-B.1.1.7
C12-P.1.2
C13-P.4
C14-P.1
C15-P.4

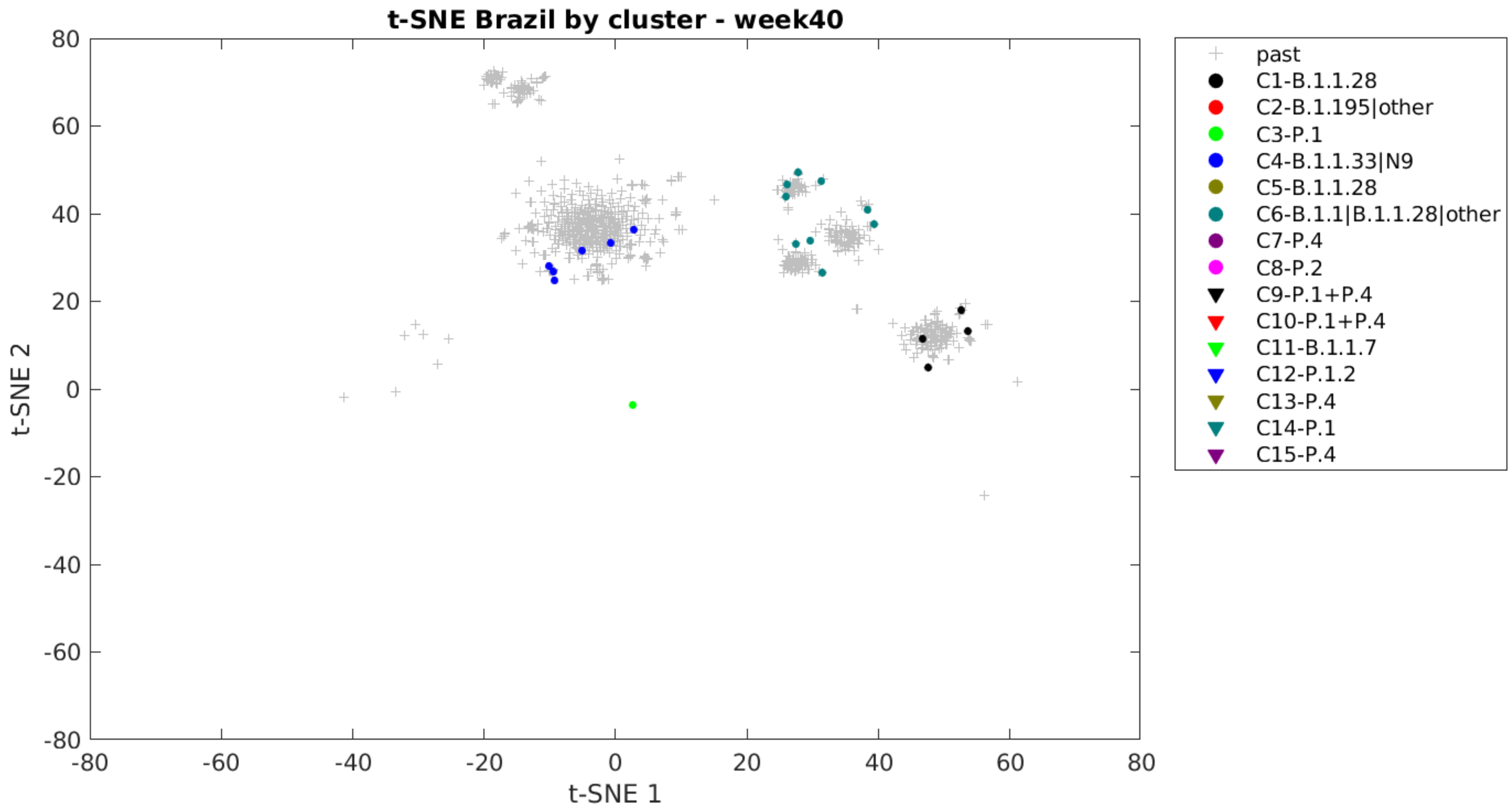

t-SNE Brazil by cluster - week40
t-SNE 1
t-SNE 2
80
60
40
20
0
-20
-40
-60
-80
-80
-60
-40
-20
0
20
40
60
80
past
C1-B.1.1.28
C2-B.1.195|other
C3-P.1
C4-B.1.1.33|N9
C5-B.1.1.28
C6-B.1.1|B.1.1.28|other
C7-P.4
C8-P.2
C9-P.1+P.4
C10-P.1+P.4
C11-B.1.1.7
C12-P.1.2
C13-P.4
C14-P.1
C15-P.4

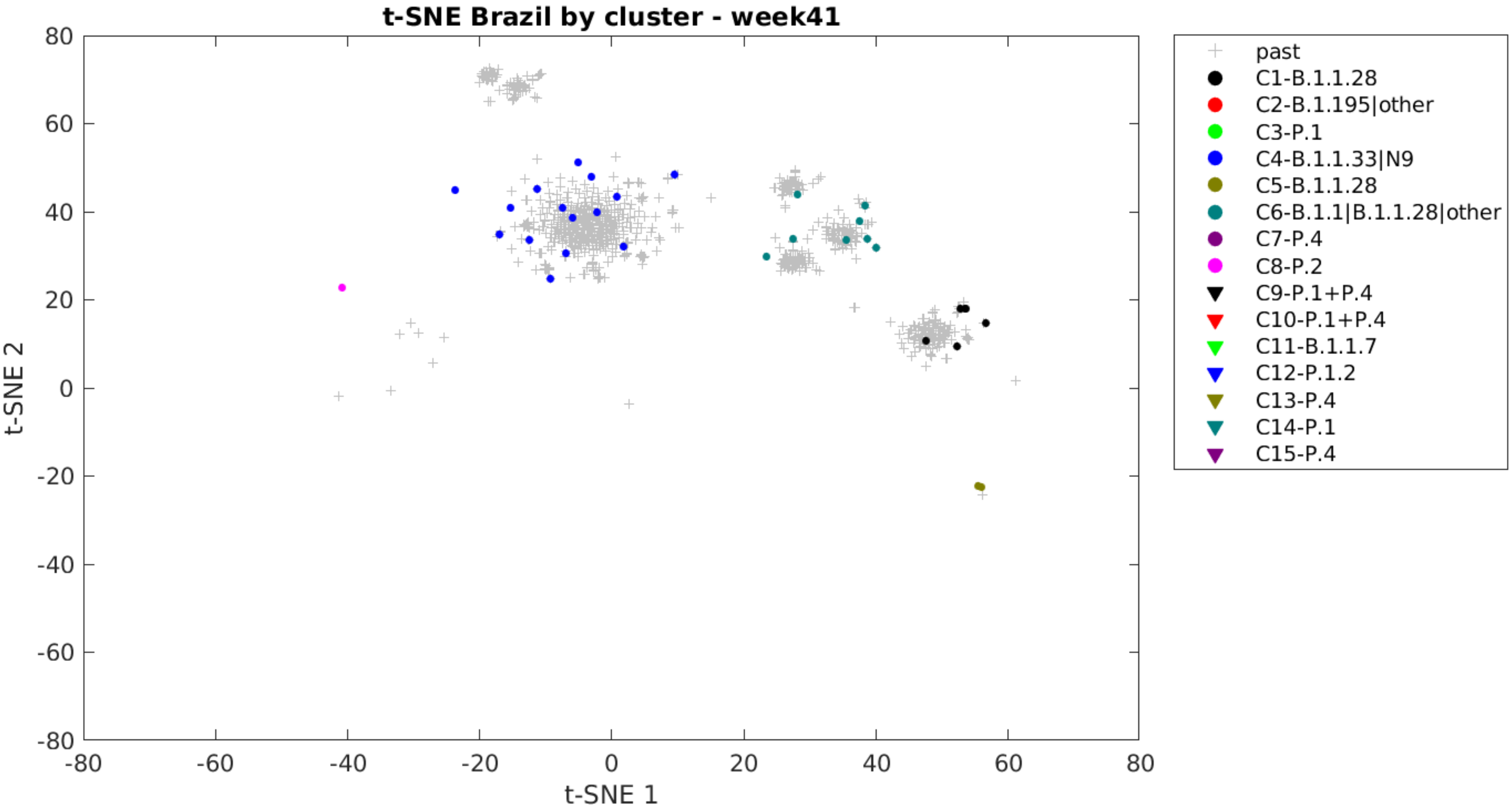
t-SNE Brazil by cluster - week41
t-SNE 1
t-SNE 2
80
60
40
20
0
-20
-40
-60
-80
-80
-60
-40
-20
0
20
40
60
80
past
C1-B.1.1.28
C2-B.1.195|other
C3-P.1
C4-B.1.1.33|N9
C5-B.1.1.28
C6-B.1.1|B.1.1.28|other
C7-P.4
C8-P.2
C9-P.1+P.4
C10-P.1+P.4
C11-B.1.1.7
C12-P.1.2
C13-P.4
C14-P.1
C15-P.4

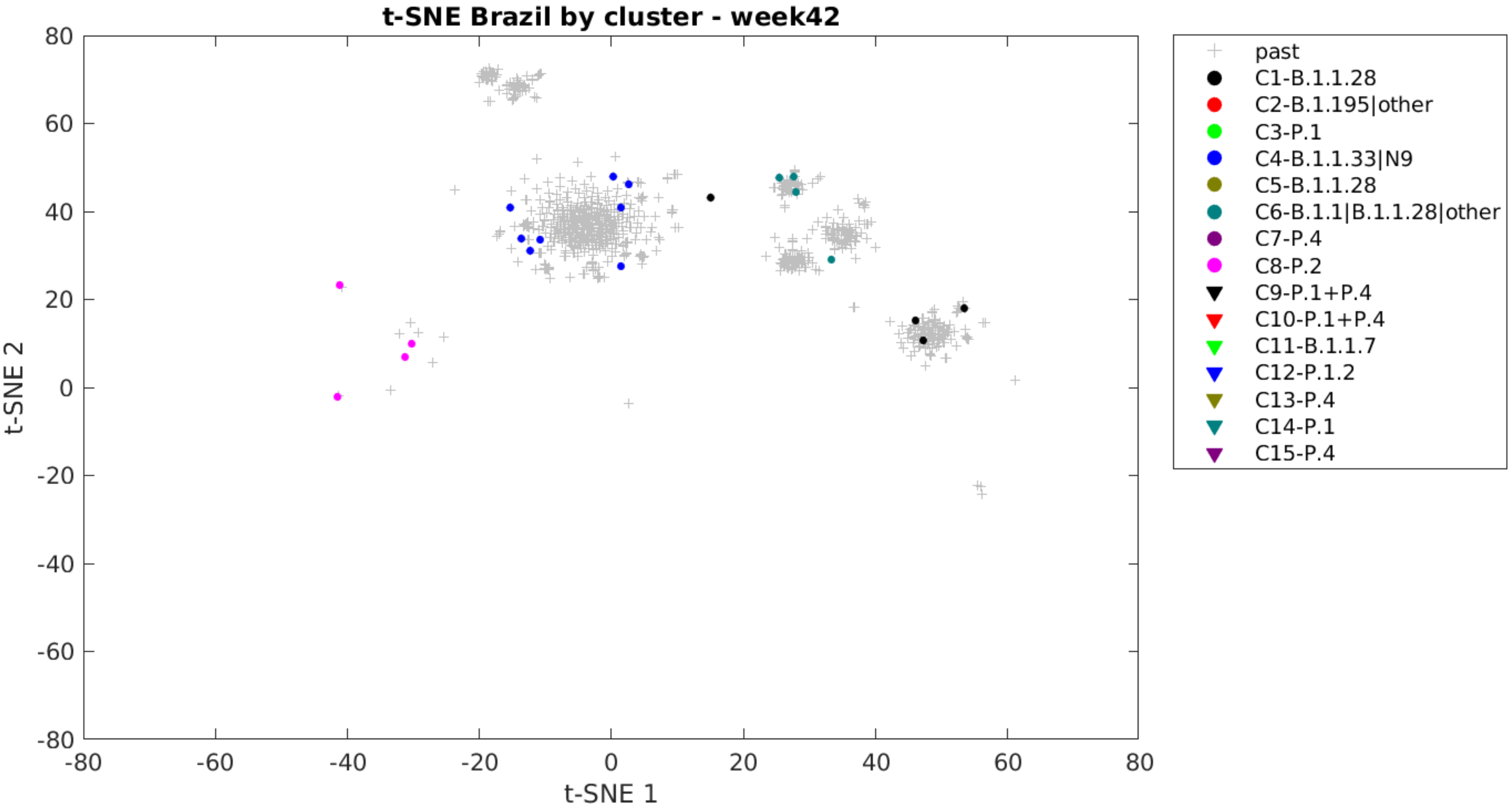
t-SNE Brazil by cluster - week42
80
60
40
20
0
-20
-40
-60
-80
t-SNE 2
-80
-60
-40
-20
0
20
40
60
80
t-SNE 1
past
C1-B.1.1.28
C2-B.1.195|other
C3-P.1
C4-B.1.1.33|N9
C5-B.1.1.28
C6-B.1.1|B.1.1.28|other
C7-P.4
C8-P.2
C9-P.1+P.4
C10-P.1+P.4
C11-B.1.1.7
C12-P.1.2
C13-P.4
C14-P.1
C15-P.4

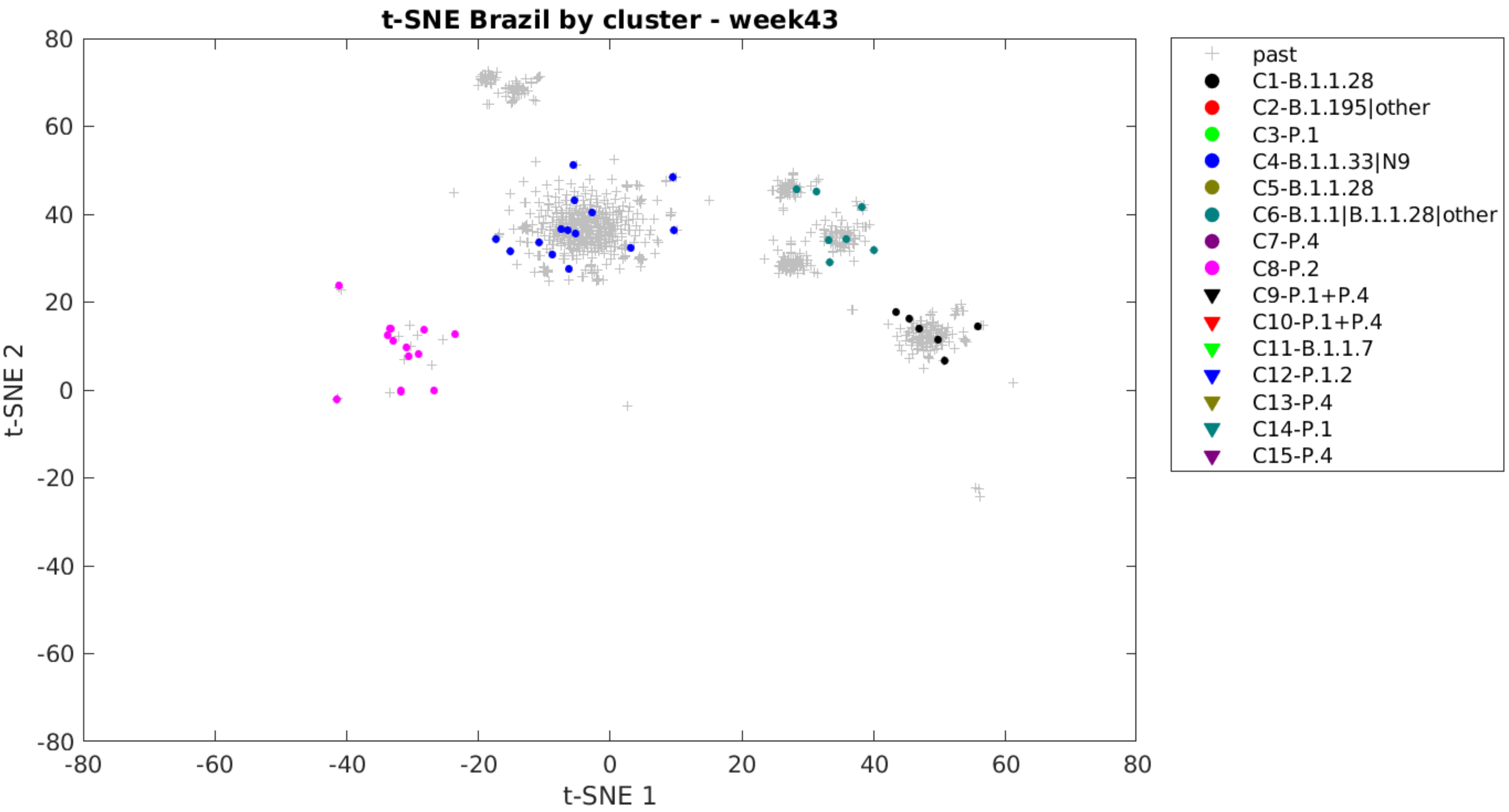
t-SNE Brazil by cluster - week43
t-SNE 1
t-SNE 2
past
C1-B.1.1.28
C2-B.1.195|other
C3-P.1
C4-B.1.1.33|N9
C5-B.1.1.28
C6-B.1.1|B.1.1.28|other
C7-P.4
C8-P.2
C9-P.1+P.4
C10-P.1+P.4
C11-B.1.1.7
C12-P.1.2
C13-P.4
C14-P.1
C15-P.4

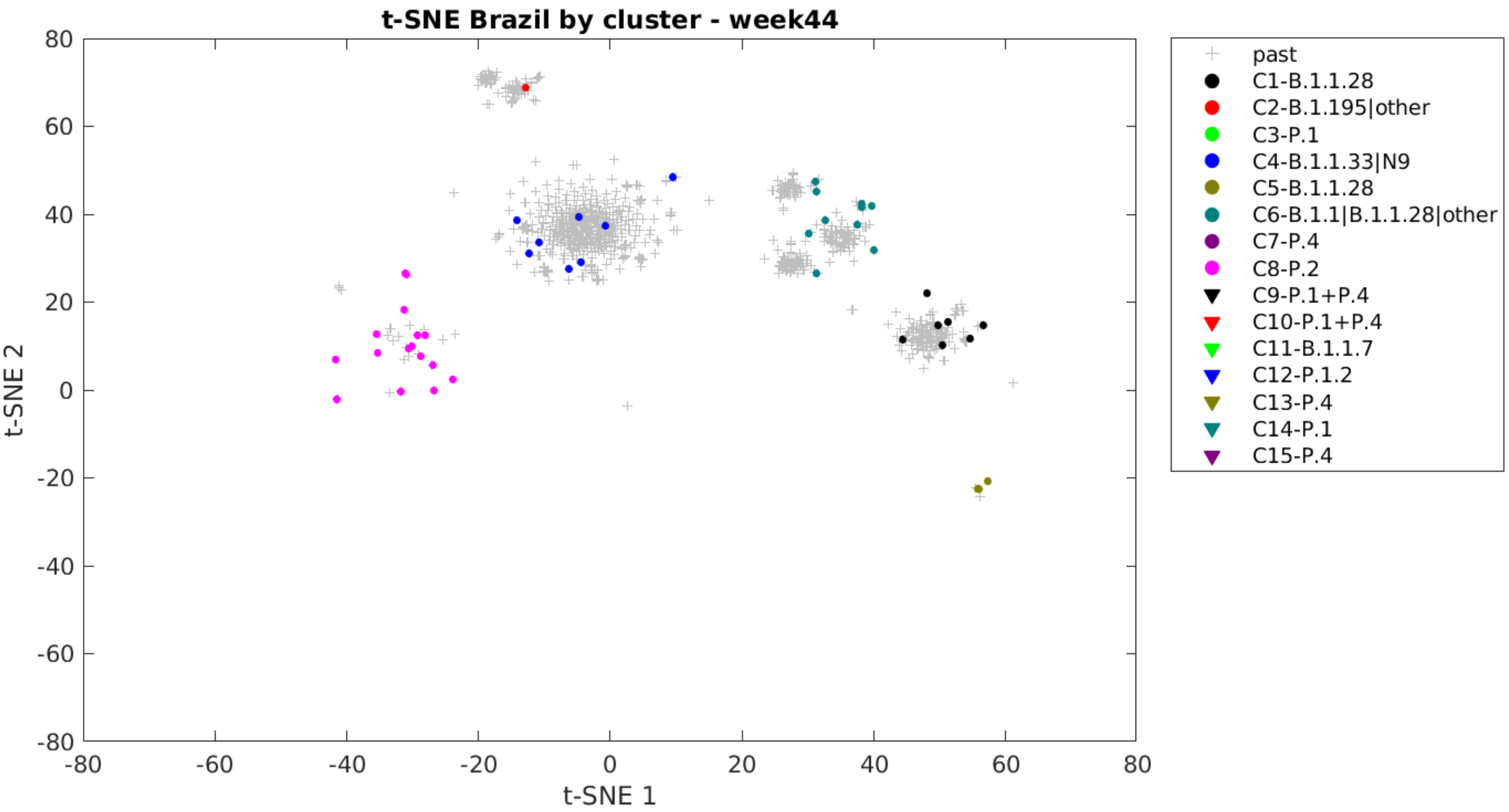

t-SNE Brazil by cluster - week44
t-SNE 2
t-SNE 1
80
60
40
20
0
-20
-40
-60
-80
-80
-60
-40
-20
0
20
40
60
80
past
C1-B.1.1.28
C2-B.1.195|other
C3-P.1
C4-B.1.1.33|N9
C5-B.1.1.28
C6-B.1.1|B.1.1.28|other
C7-P.4
C8-P.2
C9-P.1+P.4
C10-P.1+P.4
C11-B.1.1.7
C12-P.1.2
C13-P.4
C14-P.1
C15-P.4

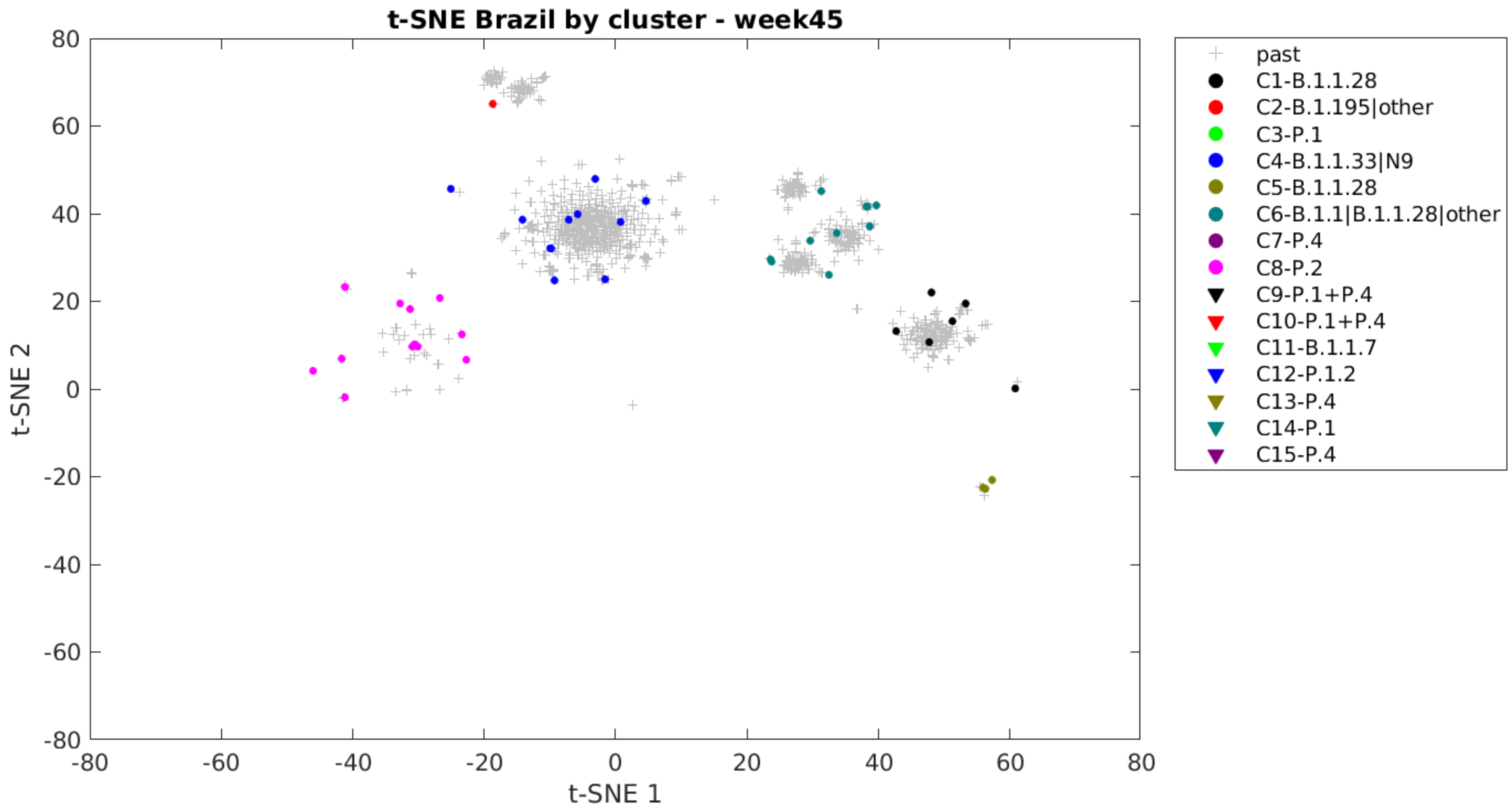

t-SNE Brazil by cluster - week45
t-SNE 2
t-SNE 1
80
60
40
20
0
-20
-40
-60
-80
-80
-60
-40
-20
0
20
40
60
80
past
C1-B.1.1.28
C2-B.1.195|other
C3-P.1
C4-B.1.1.33|N9
C5-B.1.1.28
C6-B.1.1|B.1.1.28|other
C7-P.4
C8-P.2
C9-P.1+P.4
C10-P.1+P.4
C11-B.1.1.7
C12-P.1.2
C13-P.4
C14-P.1
C15-P.4

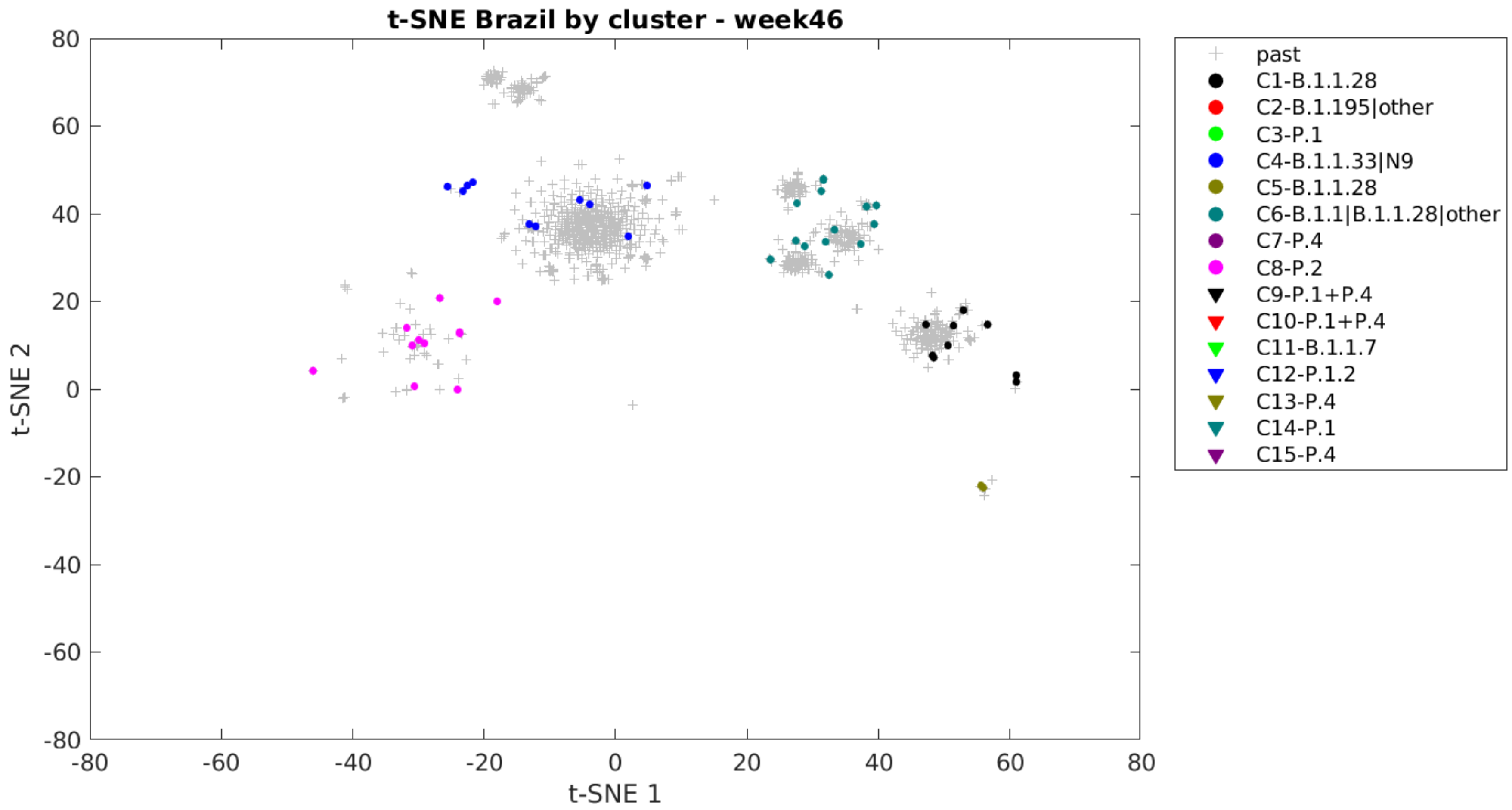
t-SNE Brazil by cluster - week46
t-SNE 1
t-SNE 2
80
60
40
20
0
-20
-40
-60
-80
-80
-60
-40
-20
0
20
40
60
80
past
C1-B.1.1.28
C2-B.1.195|other
C3-P.1
C4-B.1.1.33|N9
C5-B.1.1.28
C6-B.1.1|B.1.1.28|other
C7-P.4
C8-P.2
C9-P.1+P.4
C10-P.1+P.4
C11-B.1.1.7
C12-P.1.2
C13-P.4
C14-P.1
C15-P.4

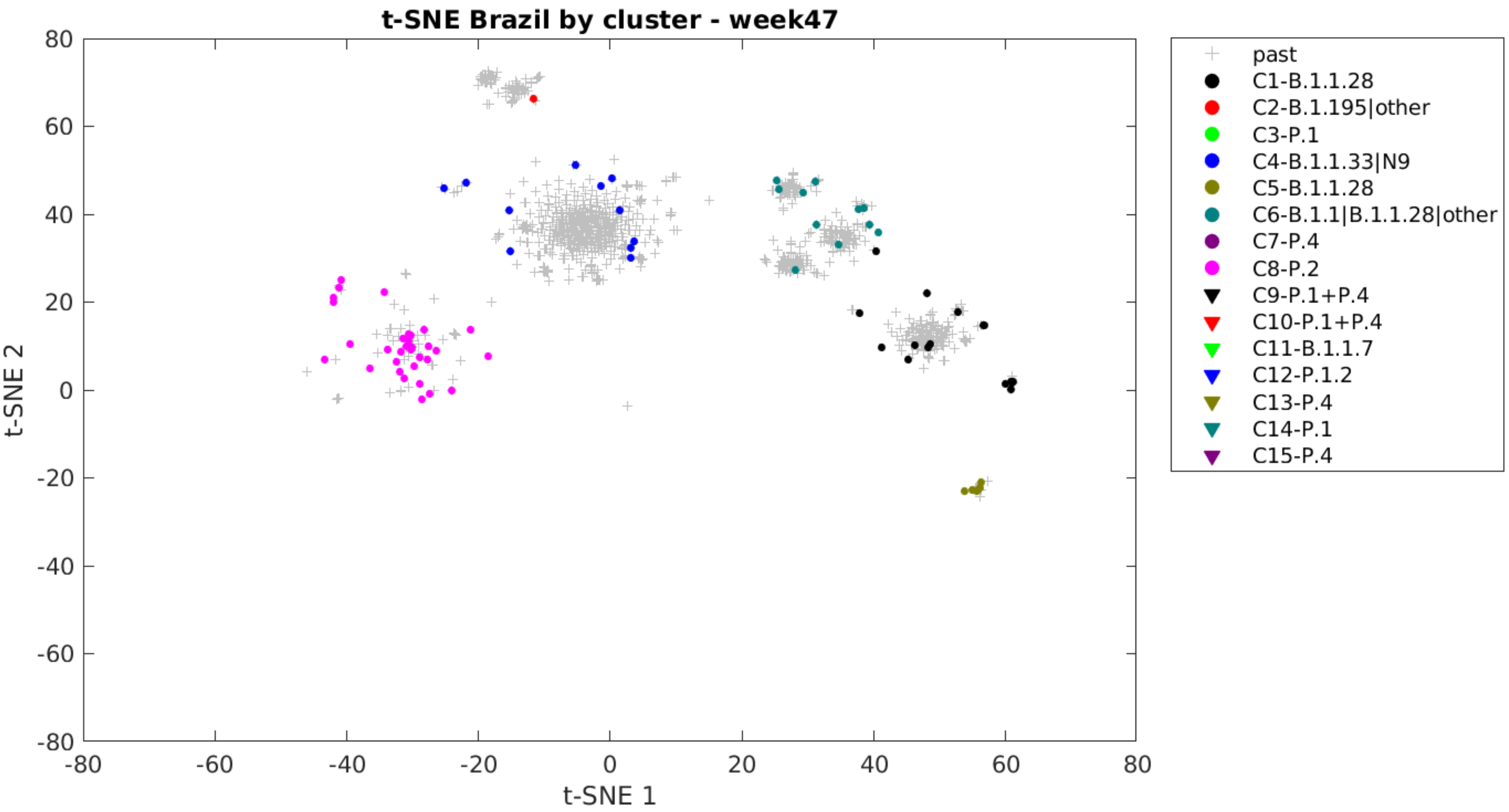
t-SNE Brazil by cluster - week47
t-SNE 1
t-SNE 2
80
60
40
20
0
-20
-40
-60
-80
-80
-60
-40
-20
0
20
40
60
80
past
C1-B.1.1.28
C2-B.1.195|other
C3-P.1
C4-B.1.1.33|N9
C5-B.1.1.28
C6-B.1.1|B.1.1.28|other
C7-P.4
C8-P.2
C9-P.1+P.4
C10-P.1+P.4
C11-B.1.1.7
C12-P.1.2
C13-P.4
C14-P.1
C15-P.4

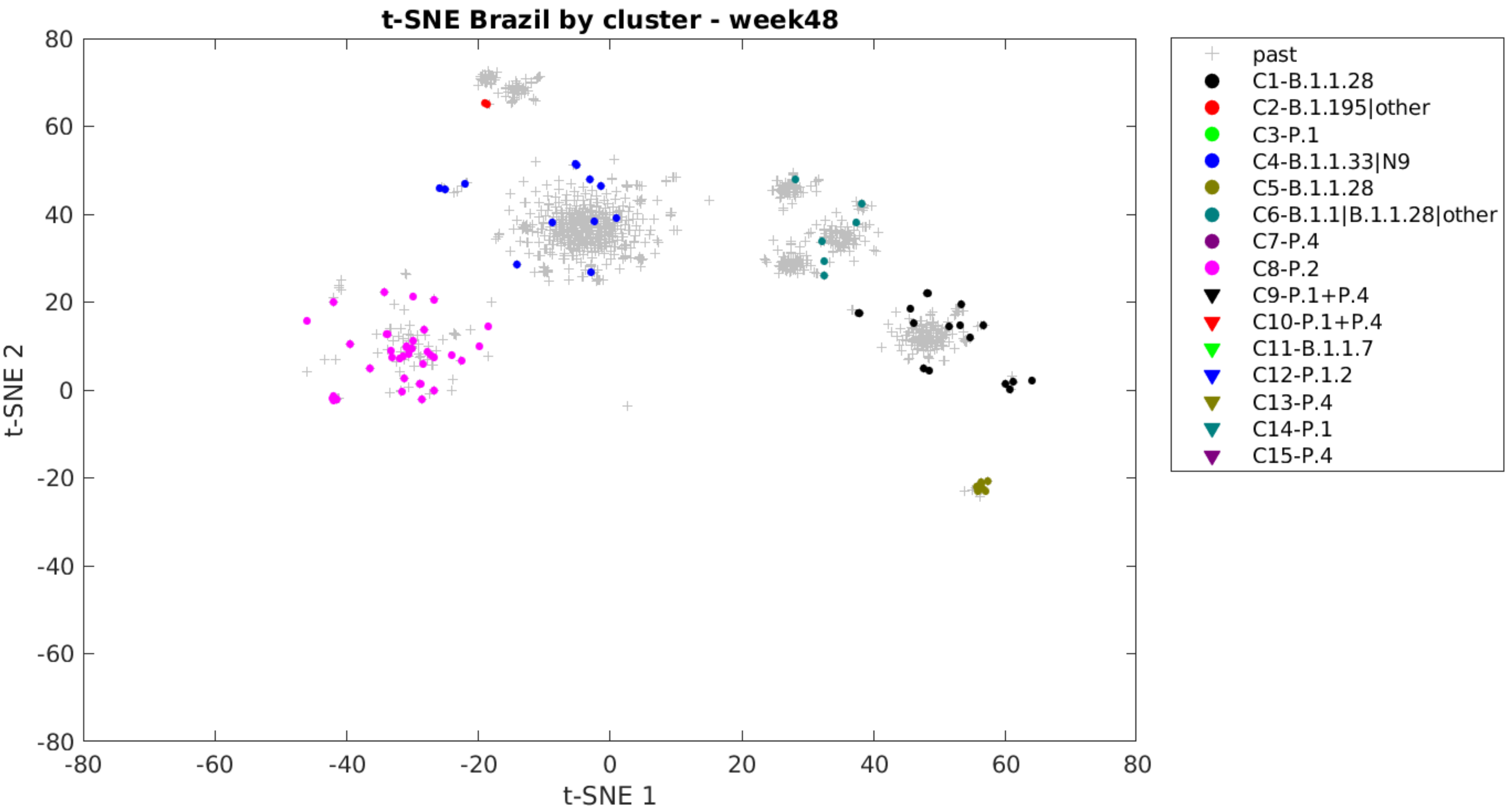
t-SNE Brazil by cluster - week48
t-SNE 1
t-SNE 2
past
C1-B.1.1.28
C2-B.1.195|other
C3-P.1
C4-B.1.1.33|N9
C5-B.1.1.28
C6-B.1.1|B.1.1.28|other
C7-P.4
C8-P.2
C9-P.1+P.4
C10-P.1+P.4
C11-B.1.1.7
C12-P.1.2
C13-P.4
C14-P.1
C15-P.4

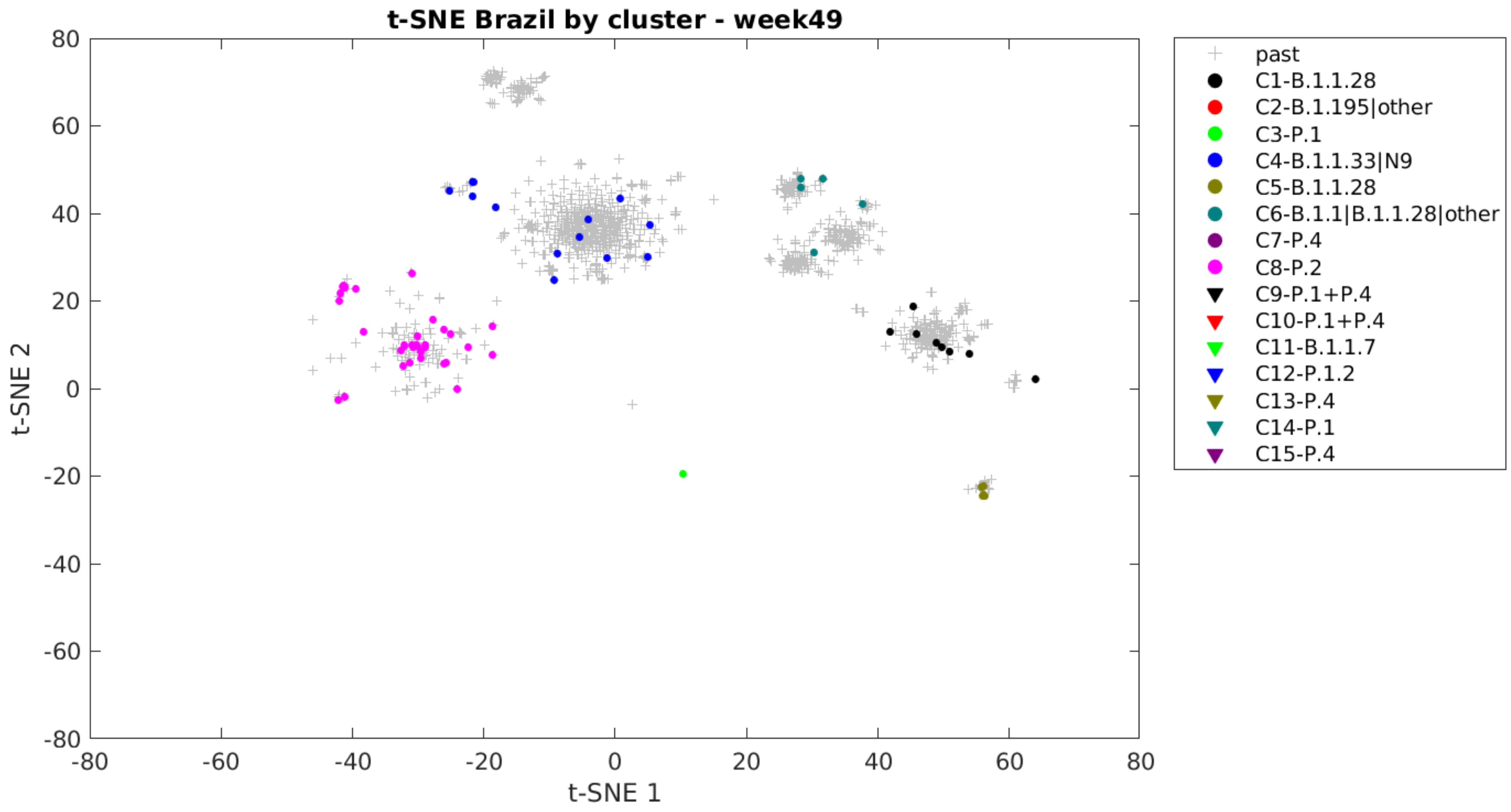
t-SNE Brazil by cluster - week49
t-SNE 1
t-SNE 2
80
60
40
20
0
-20
-40
-60
-80
-80
-60
-40
-20
0
20
40
60
80
past
C1-B.1.1.28
C2-B.1.195|other
C3-P.1
C4-B.1.1.33|N9
C5-B.1.1.28
C6-B.1.1|B.1.1.28|other
C7-P.4
C8-P.2
C9-P.1+P.4
C10-P.1+P.4
C11-B.1.1.7
C12-P.1.2
C13-P.4
C14-P.1
C15-P.4

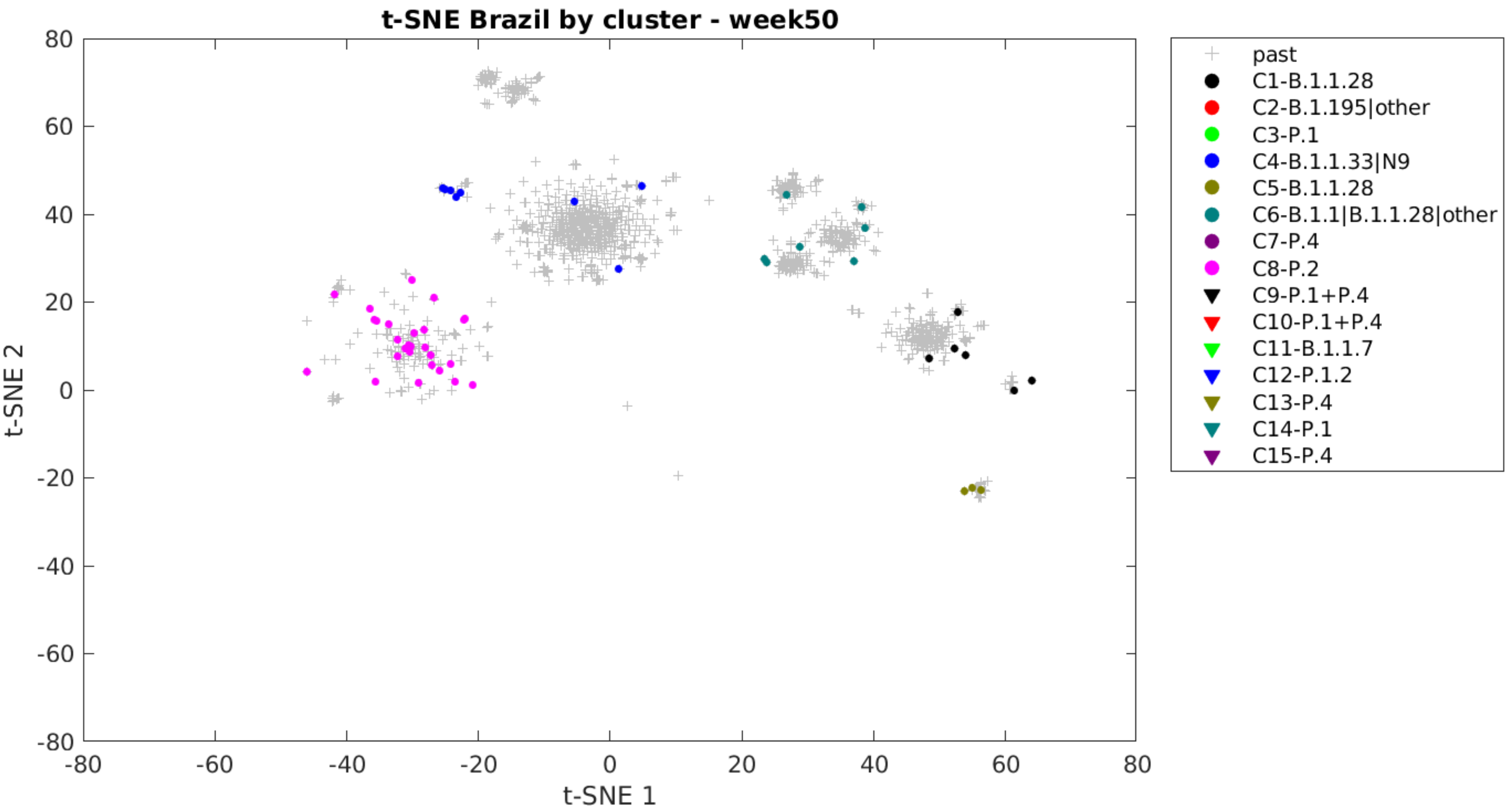
t-SNE Brazil by cluster - week50
t-SNE 1
t-SNE 2
80
60
40
20
0
-20
-40
-60
-80
-80
-60
-40
-20
0
20
40
60
80
past
C1-B.1.1.28
C2-B.1.195|other
C3-P.1
C4-B.1.1.33|N9
C5-B.1.1.28
C6-B.1.1|B.1.1.28|other
C7-P.4
C8-P.2
C9-P.1+P.4
C10-P.1+P.4
C11-B.1.1.7
C12-P.1.2
C13-P.4
C14-P.1
C15-P.4

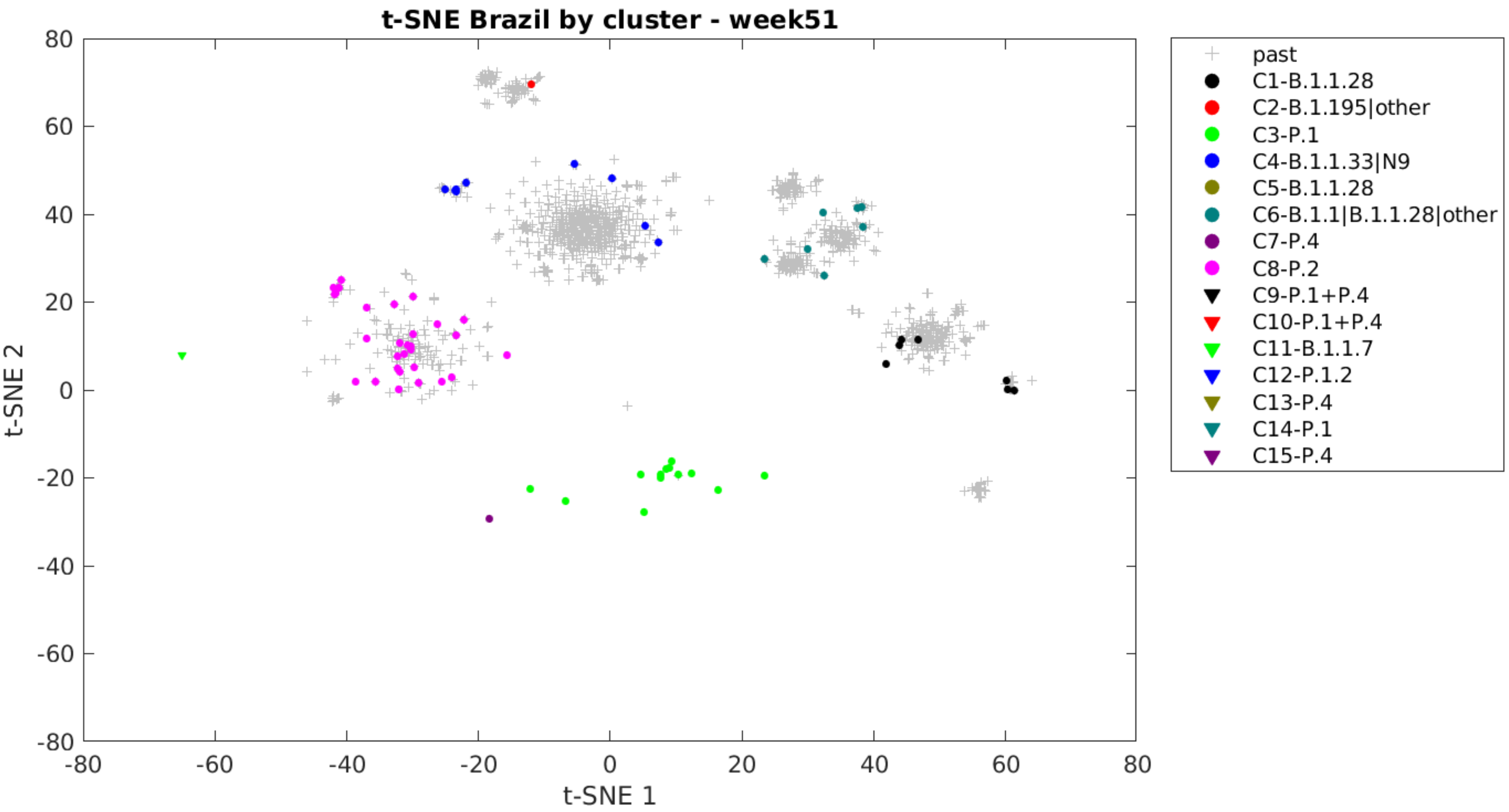
t-SNE Brazil by cluster - week51
t-SNE 2
t-SNE 1
80
60
40
20
0
-20
-40
-60
-80
-80
-60
-40
-20
0
20
40
60
80
past
C1-B.1.1.28
C2-B.1.195|other
C3-P.1
C4-B.1.1.33|N9
C5-B.1.1.28
C6-B.1.1|B.1.1.28|other
C7-P.4
C8-P.2
C9-P.1+P.4
C10-P.1+P.4
C11-B.1.1.7
C12-P.1.2
C13-P.4
C14-P.1
C15-P.4

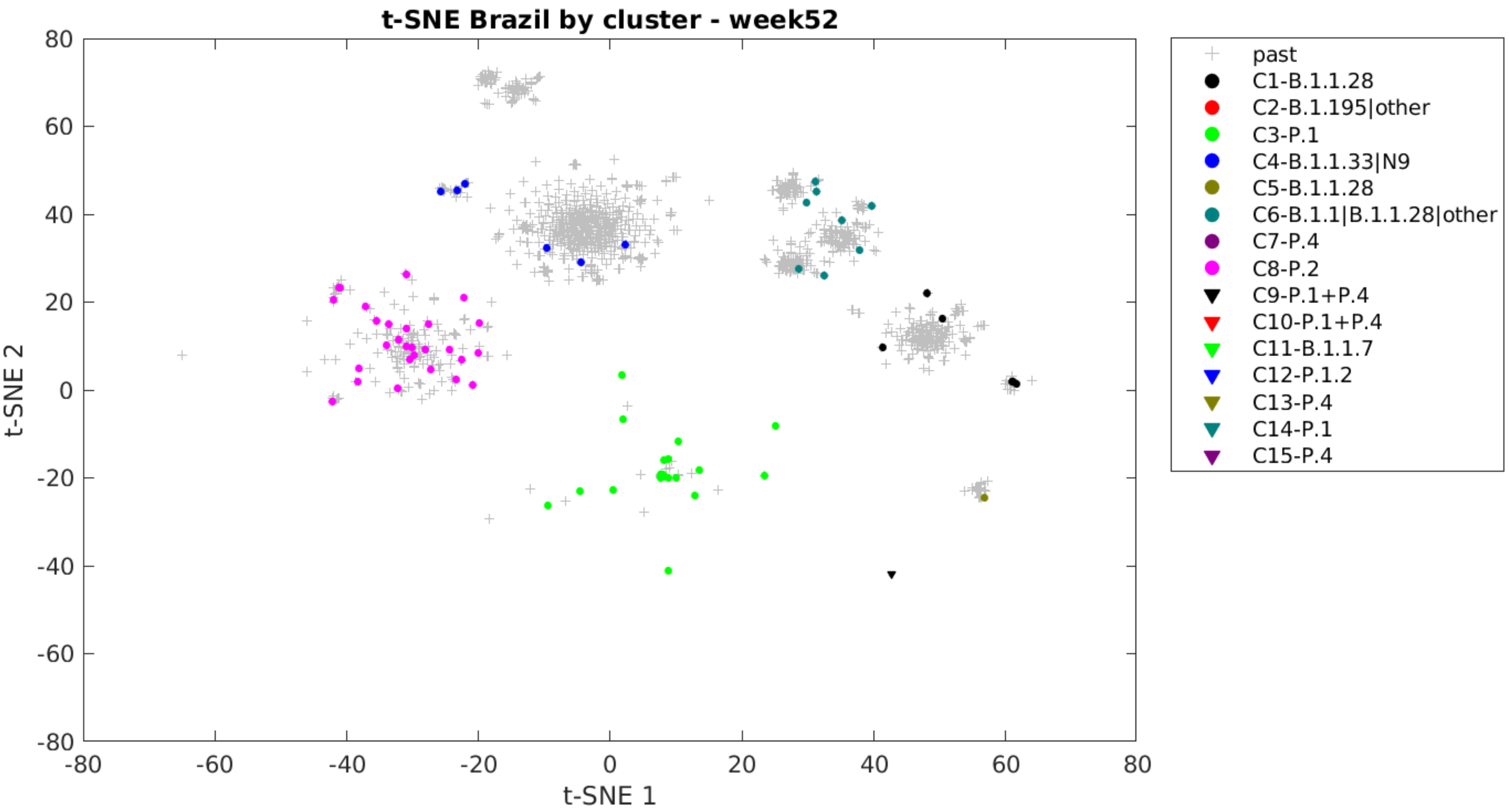

t-SNE Brazil by cluster - week52
t-SNE 2
t-SNE 1
80
60
40
20
0
-20
-40
-60
-80
-80
-60
-40
-20
0
20
40
60
80
past
C1-B.1.1.28
C2-B.1.195|other
C3-P.1
C4-B.1.1.33|N9
C5-B.1.1.28
C6-B.1.1|B.1.1.28|other
C7-P.4
C8-P.2
C9-P.1+P.4
C10-P.1+P.4
C11-B.1.1.7
C12-P.1.2
C13-P.4
C14-P.1
C15-P.4

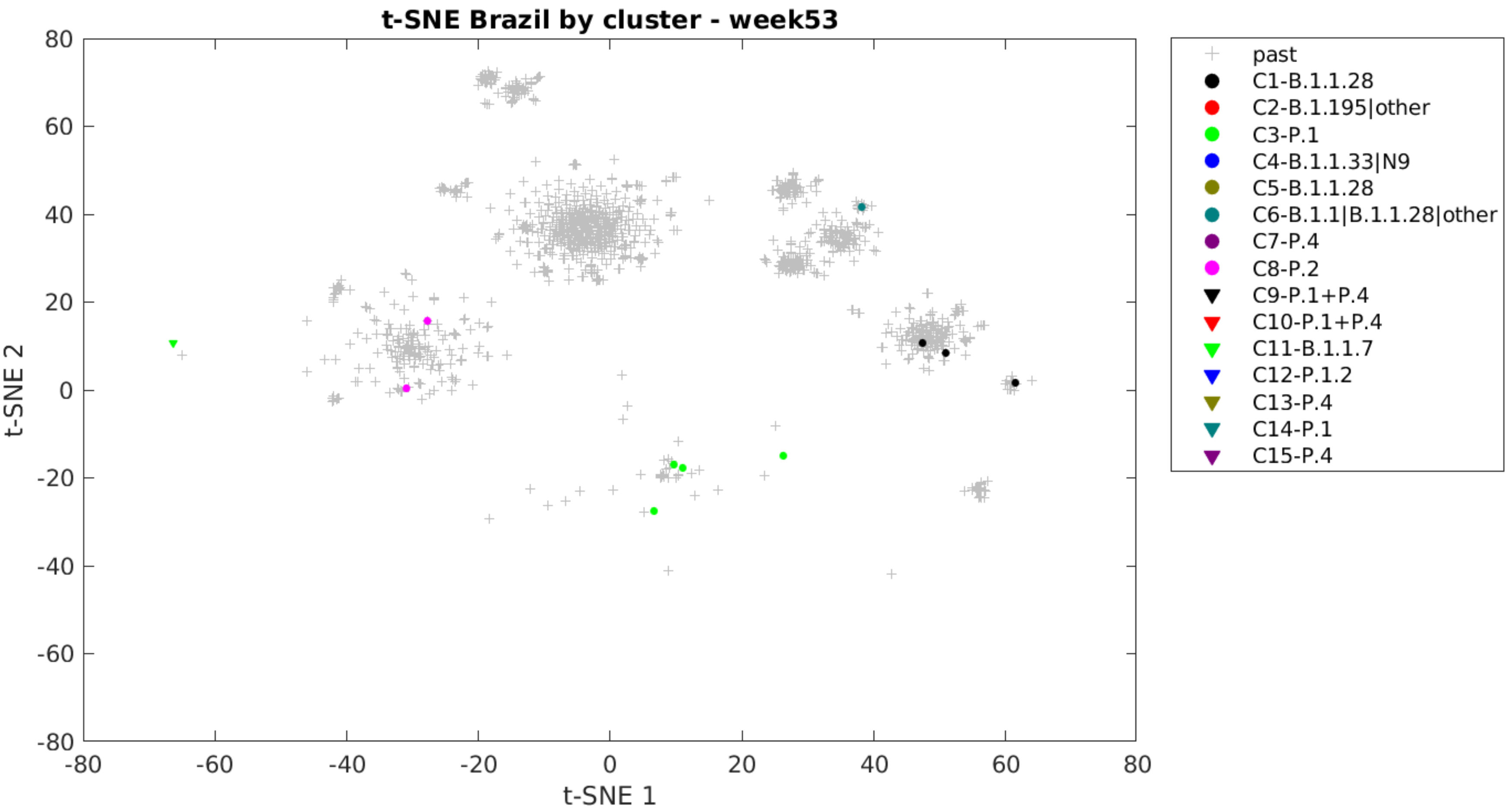

t-SNE Brazil by cluster - week53
t-SNE 2
t-SNE 1
80
60
40
20
0
-20
-40
-60
-80
-80
-60
-40
-20
0
20
40
60
80
past
C1-B.1.1.28
C2-B.1.195|other
C3-P.1
C4-B.1.1.33|N9
C5-B.1.1.28
C6-B.1.1|B.1.1.28|other
C7-P.4
C8-P.2
C9-P.1+P.4
C10-P.1+P.4
C11-B.1.1.7
C12-P.1.2
C13-P.4
C14-P.1
C15-P.4

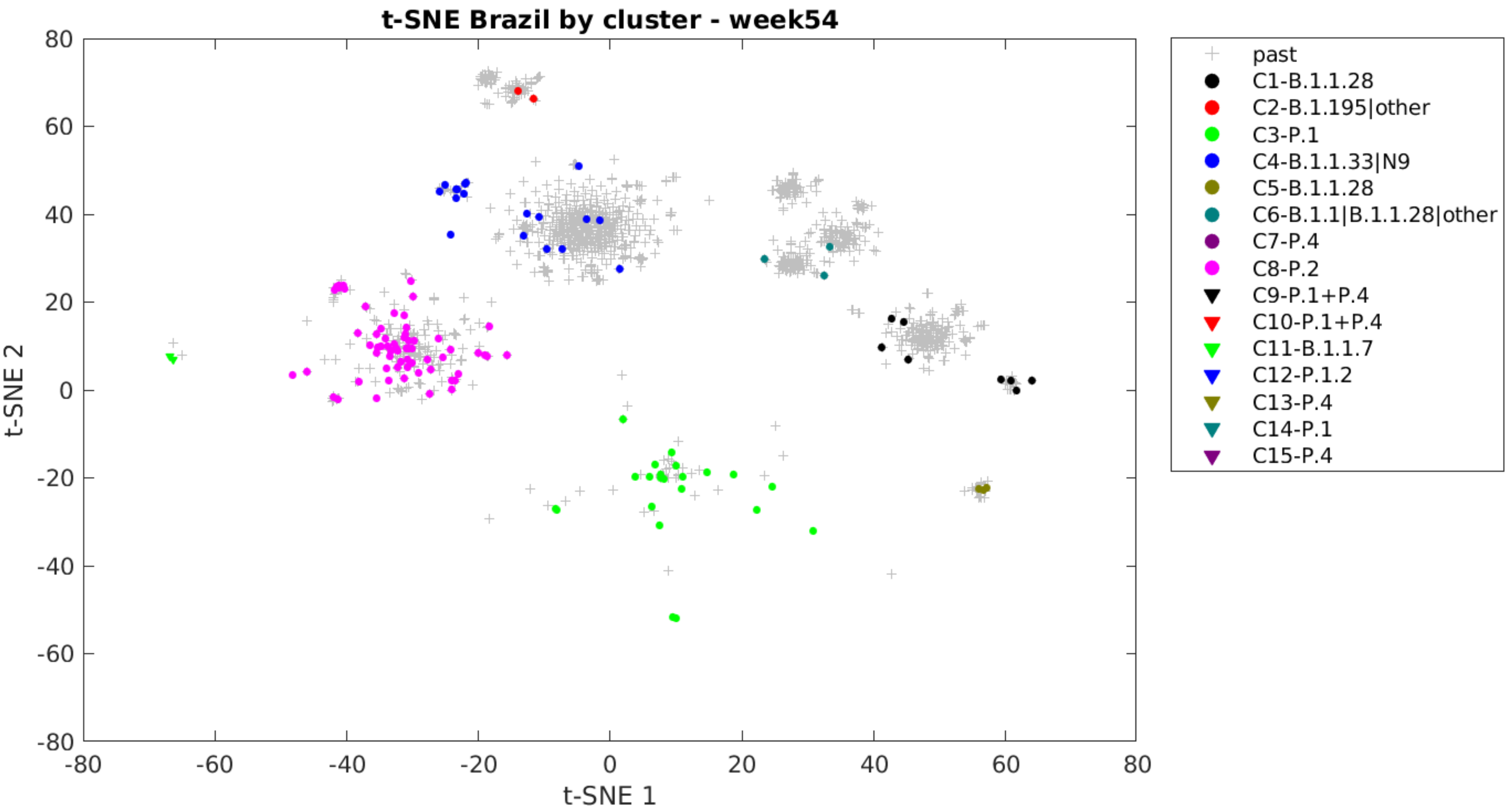

t-SNE Brazil by cluster - week54
t-SNE 2
t-SNE 1
80
60
40
20
0
-20
-40
-60
-80
-80
-60
-40
-20
0
20
40
60
80
past
C1-B.1.1.28
C2-B.1.195|other
C3-P.1
C4-B.1.1.33|N9
C5-B.1.1.28
C6-B.1.1|B.1.1.28|other
C7-P.4
C8-P.2
C9-P.1+P.4
C10-P.1+P.4
C11-B.1.1.7
C12-P.1.2
C13-P.4
C14-P.1
C15-P.4

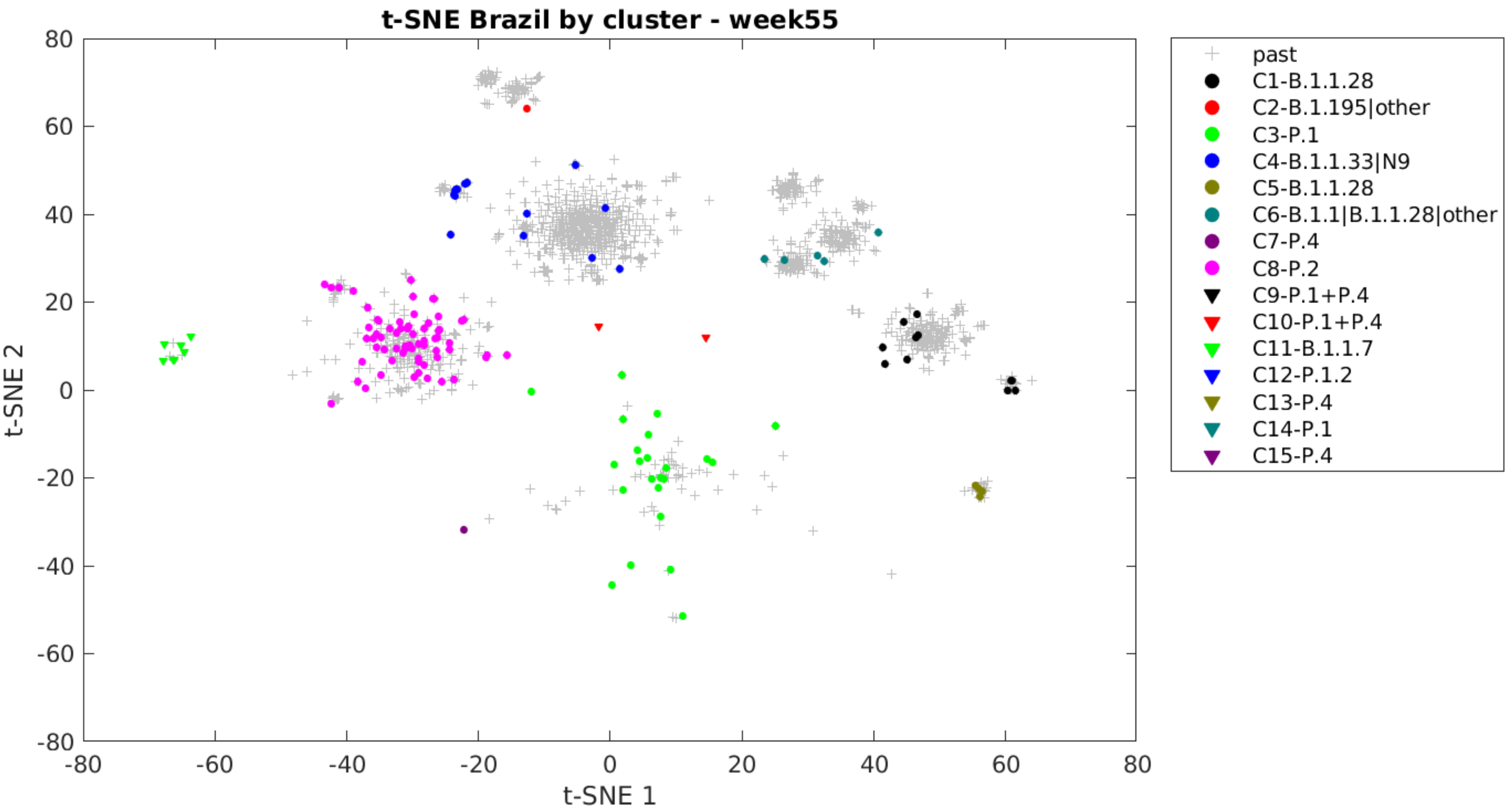
t-SNE Brazil by cluster - week55
t-SNE 1
t-SNE 2
80
60
40
20
0
-20
-40
-60
-80
-80
-60
-40
-20
0
20
40
60
80
past
C1-B.1.1.28
C2-B.1.195|other
C3-P.1
C4-B.1.1.33|N9
C5-B.1.1.28
C6-B.1.1|B.1.1.28|other
C7-P.4
C8-P.2
C9-P.1+P.4
C10-P.1+P.4
C11-B.1.1.7
C12-P.1.2
C13-P.4
C14-P.1
C15-P.4

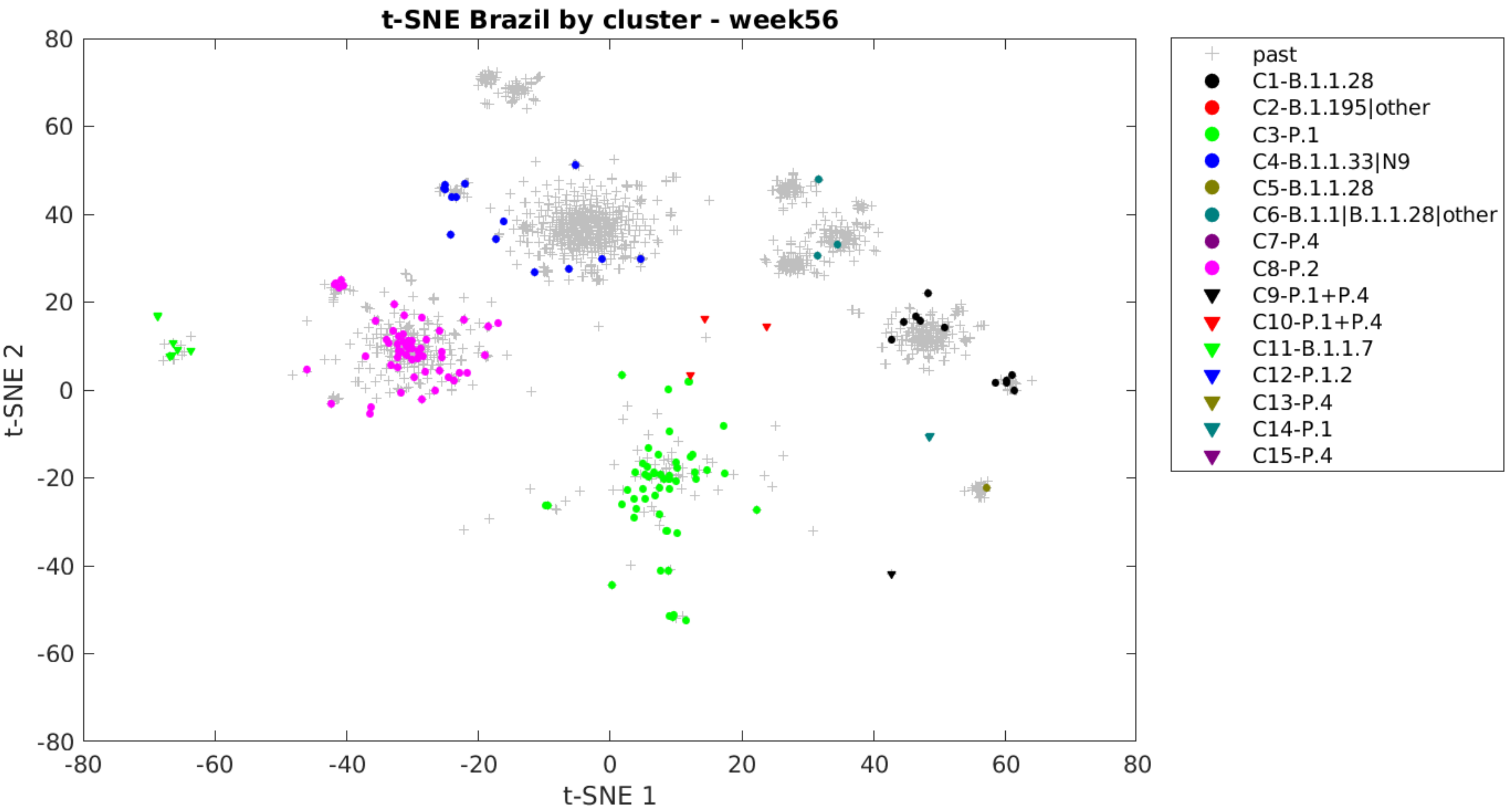

t-SNE Brazil by cluster - week56
t-SNE 2
t-SNE 1
80
60
40
20
0
-20
-40
-60
-80
-80
-60
-40
-20
0
20
40
60
80
past
C1-B.1.1.28
C2-B.1.195|other
C3-P.1
C4-B.1.1.33|N9
C5-B.1.1.28
C6-B.1.1|B.1.1.28|other
C7-P.4
C8-P.2
C9-P.1+P.4
C10-P.1+P.4
C11-B.1.1.7
C12-P.1.2
C13-P.4
C14-P.1
C15-P.4

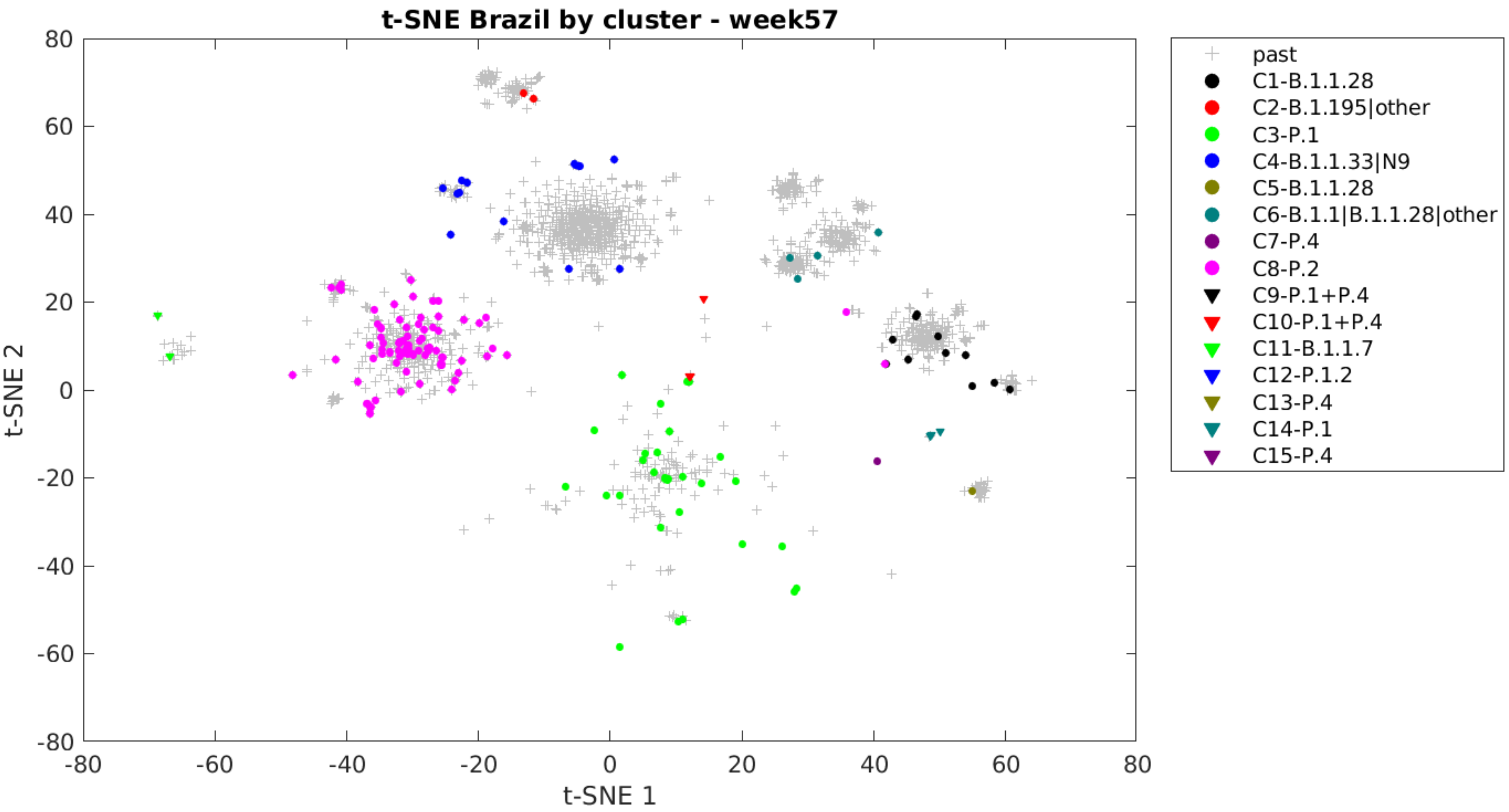

t-SNE Brazil by cluster - week57
t-SNE 2
t-SNE 1
80
60
40
20
0
-20
-40
-60
-80
-80
-60
-40
-20
0
20
40
60
80
past
C1-B.1.1.28
C2-B.1.195|other
C3-P.1
C4-B.1.1.33|N9
C5-B.1.1.28
C6-B.1.1|B.1.1.28|other
C7-P.4
C8-P.2
C9-P.1+P.4
C10-P.1+P.4
C11-B.1.1.7
C12-P.1.2
C13-P.4
C14-P.1
C15-P.4

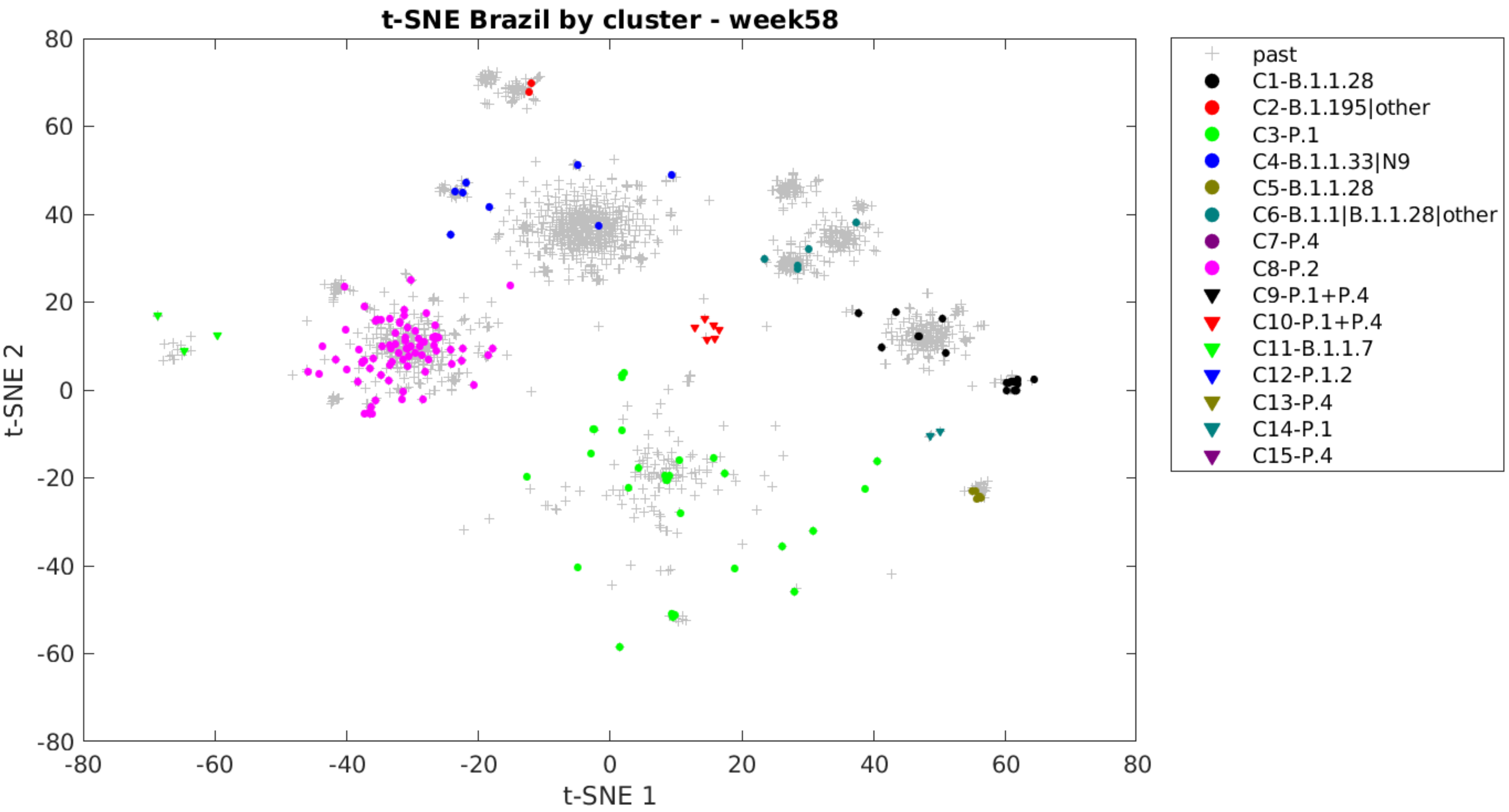
t-SNE Brazil by cluster - week58
t-SNE 1
t-SNE 2
80
60
40
20
0
-20
-40
-60
-80
-80
-60
-40
-20
0
20
40
60
80
past
C1-B.1.1.28
C2-B.1.195|other
C3-P.1
C4-B.1.1.33|N9
C5-B.1.1.28
C6-B.1.1|B.1.1.28|other
C7-P.4
C8-P.2
C9-P.1+P.4
C10-P.1+P.4
C11-B.1.1.7
C12-P.1.2
C13-P.4
C14-P.1
C15-P.4

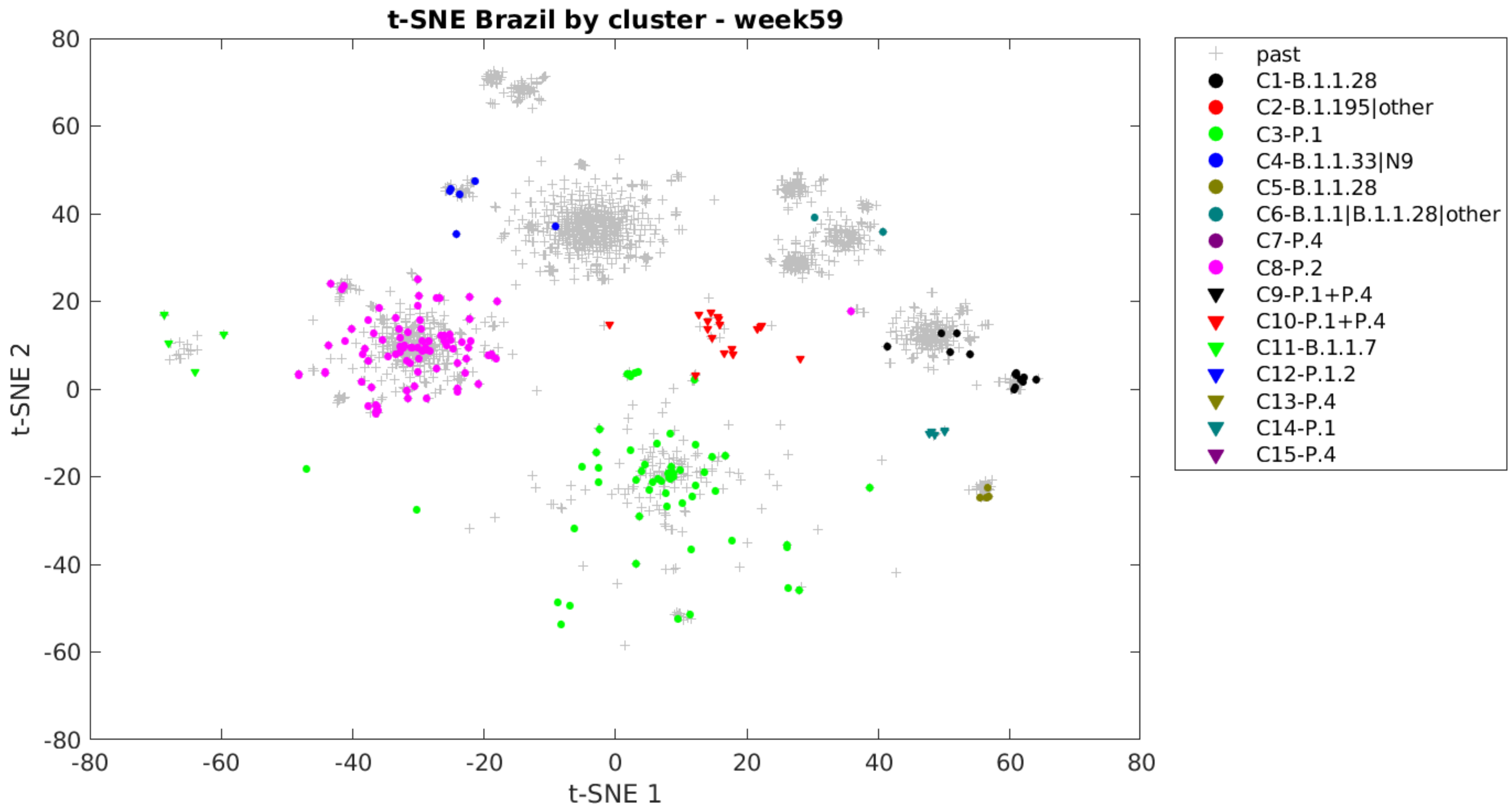
t-SNE Brazil by cluster - week59
t-SNE 1
t-SNE 2
80
60
40
20
0
-20
-40
-60
-80
past
C1-B.1.1.28
C2-B.1.195|other
C3-P.1
C4-B.1.1.33|N9
C5-B.1.1.28
C6-B.1.1|B.1.1.28|other
C7-P.4
C8-P.2
C9-P.1+P.4
C10-P.1+P.4
C11-B.1.1.7
C12-P.1.2
C13-P.4
C14-P.1
C15-P.4

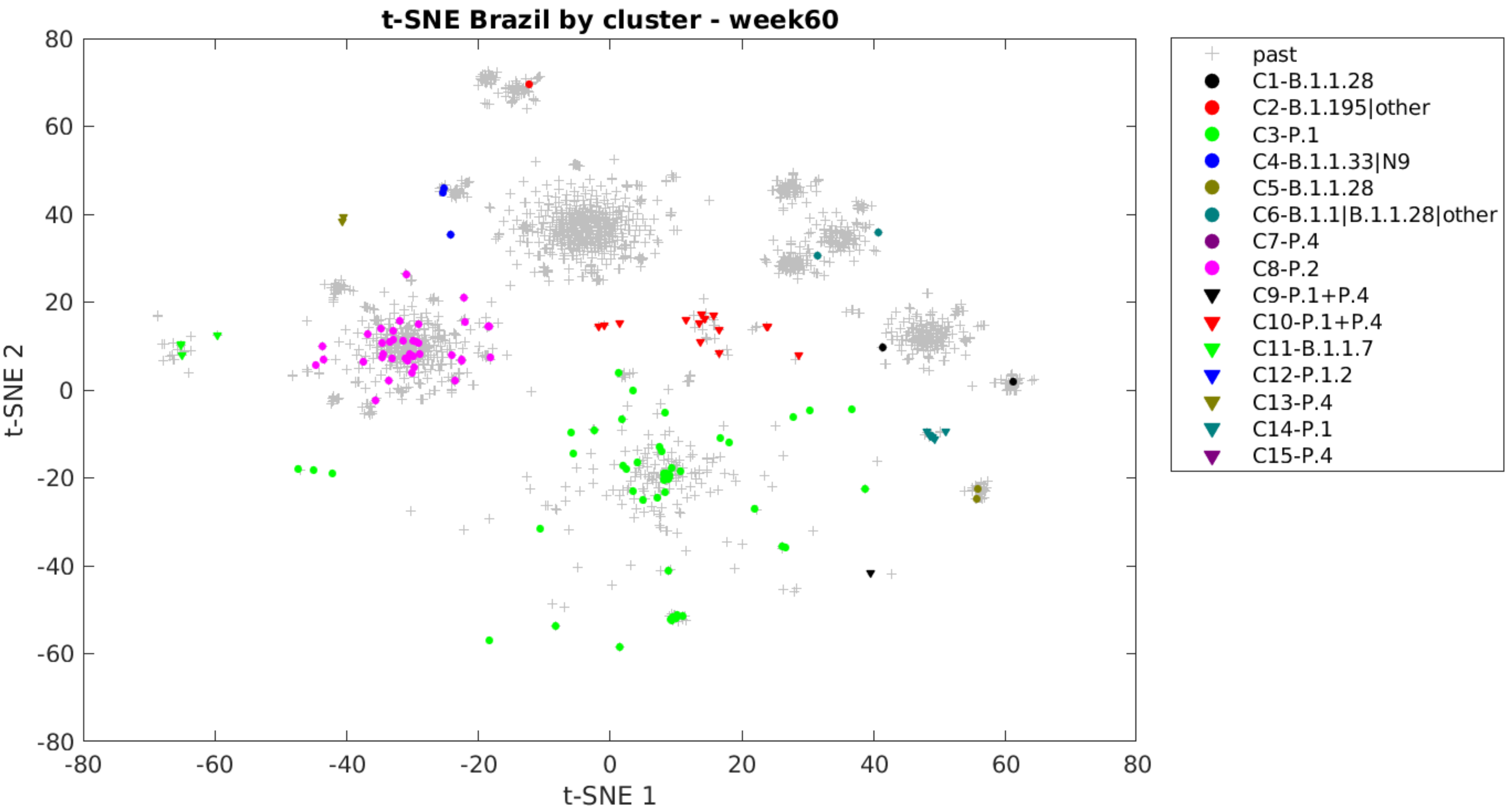

t-SNE Brazil by cluster - week60
t-SNE 2
t-SNE 1
80
60
40
20
0
-20
-40
-60
-80
-80
-60
-40
-20
0
20
40
60
80
past
C1-B.1.1.28
C2-B.1.195|other
C3-P.1
C4-B.1.1.33|N9
C5-B.1.1.28
C6-B.1.1|B.1.1.28|other
C7-P.4
C8-P.2
C9-P.1+P.4
C10-P.1+P.4
C11-B.1.1.7
C12-P.1.2
C13-P.4
C14-P.1
C15-P.4

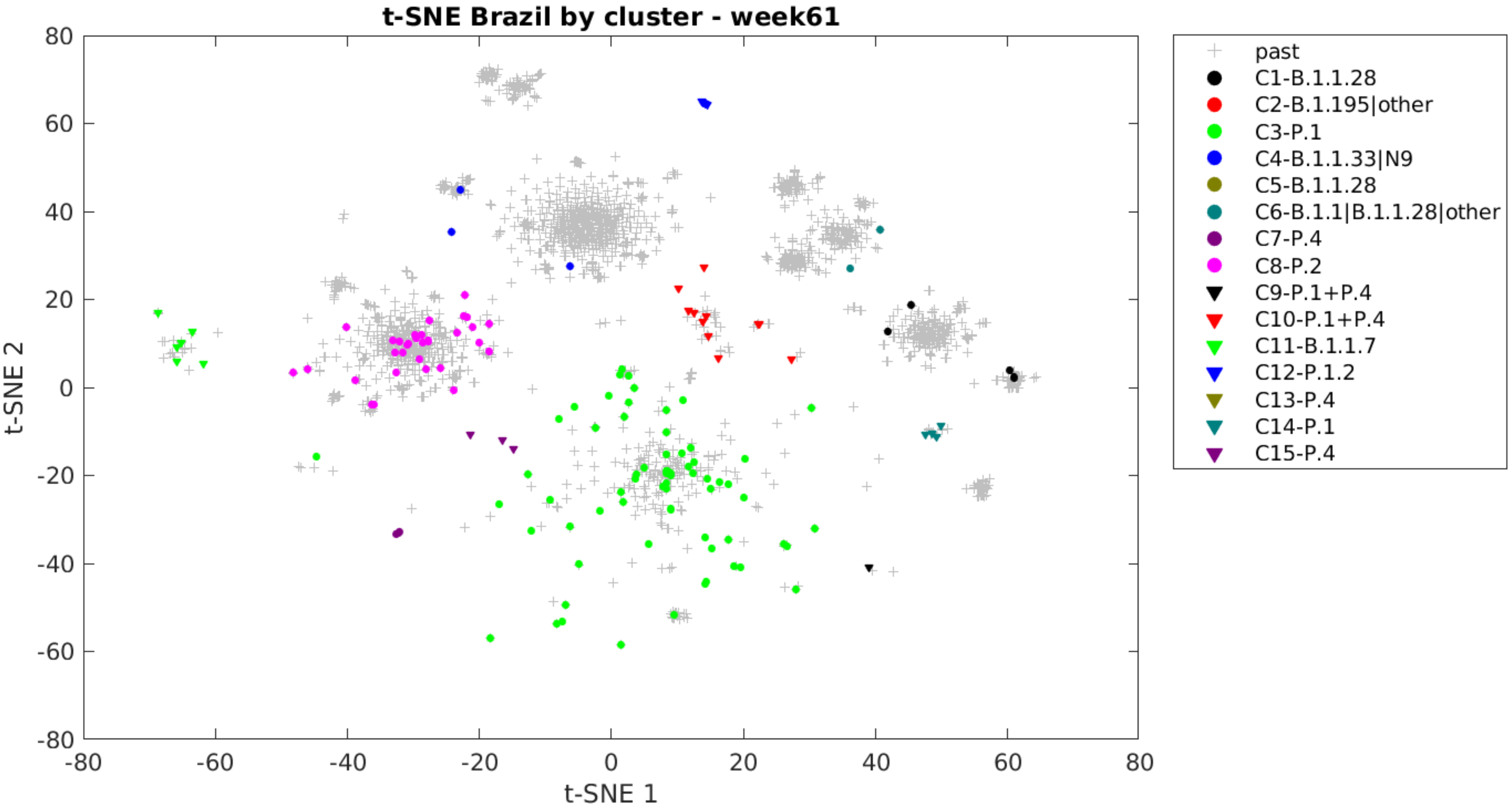

t-SNE Brazil by cluster - week61
t-SNE 2
t-SNE 1
80
60
40
20
0
-20
-40
-60
-80
-80
-60
-40
-20
0
20
40
60
80
past
C1-B.1.1.28
C2-B.1.195|other
C3-P.1
C4-B.1.1.33|N9
C5-B.1.1.28
C6-B.1.1|B.1.1.28|other
C7-P.4
C8-P.2
C9-P.1+P.4
C10-P.1+P.4
C11-B.1.1.7
C12-P.1.2
C13-P.4
C14-P.1
C15-P.4

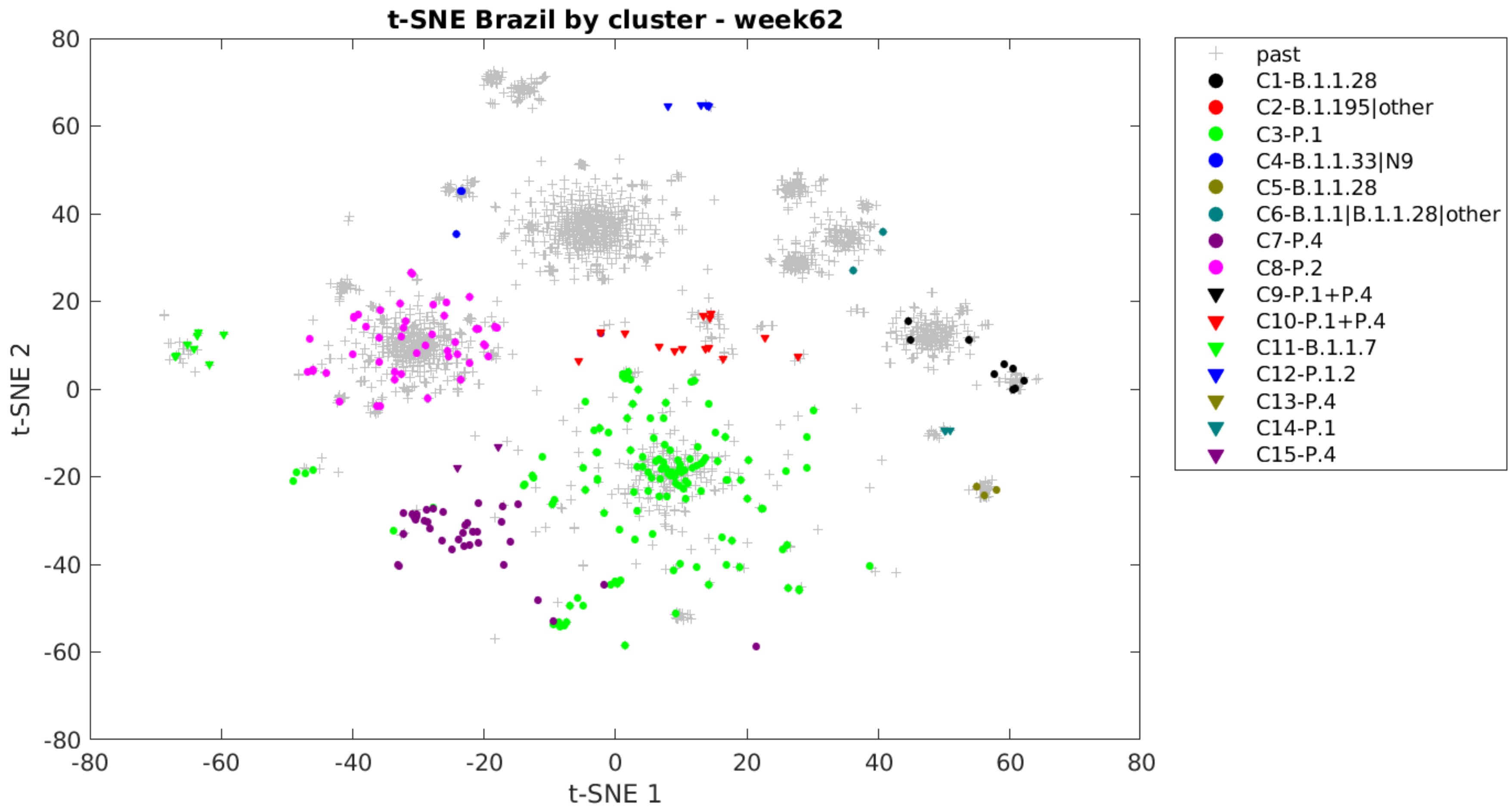
t-SNE Brazil by cluster - week62
t-SNE 1
t-SNE 2
80
60
40
20
0
-20
-40
-60
-80
-80
-60
-40
-20
0
20
40
60
80
past
C1-B.1.1.28
C2-B.1.195|other
C3-P.1
C4-B.1.1.33|N9
C5-B.1.1.28
C6-B.1.1|B.1.1.28|other
C7-P.4
C8-P.2
C9-P.1+P.4
C10-P.1+P.4
C11-B.1.1.7
C12-P.1.2
C13-P.4
C14-P.1
C15-P.4

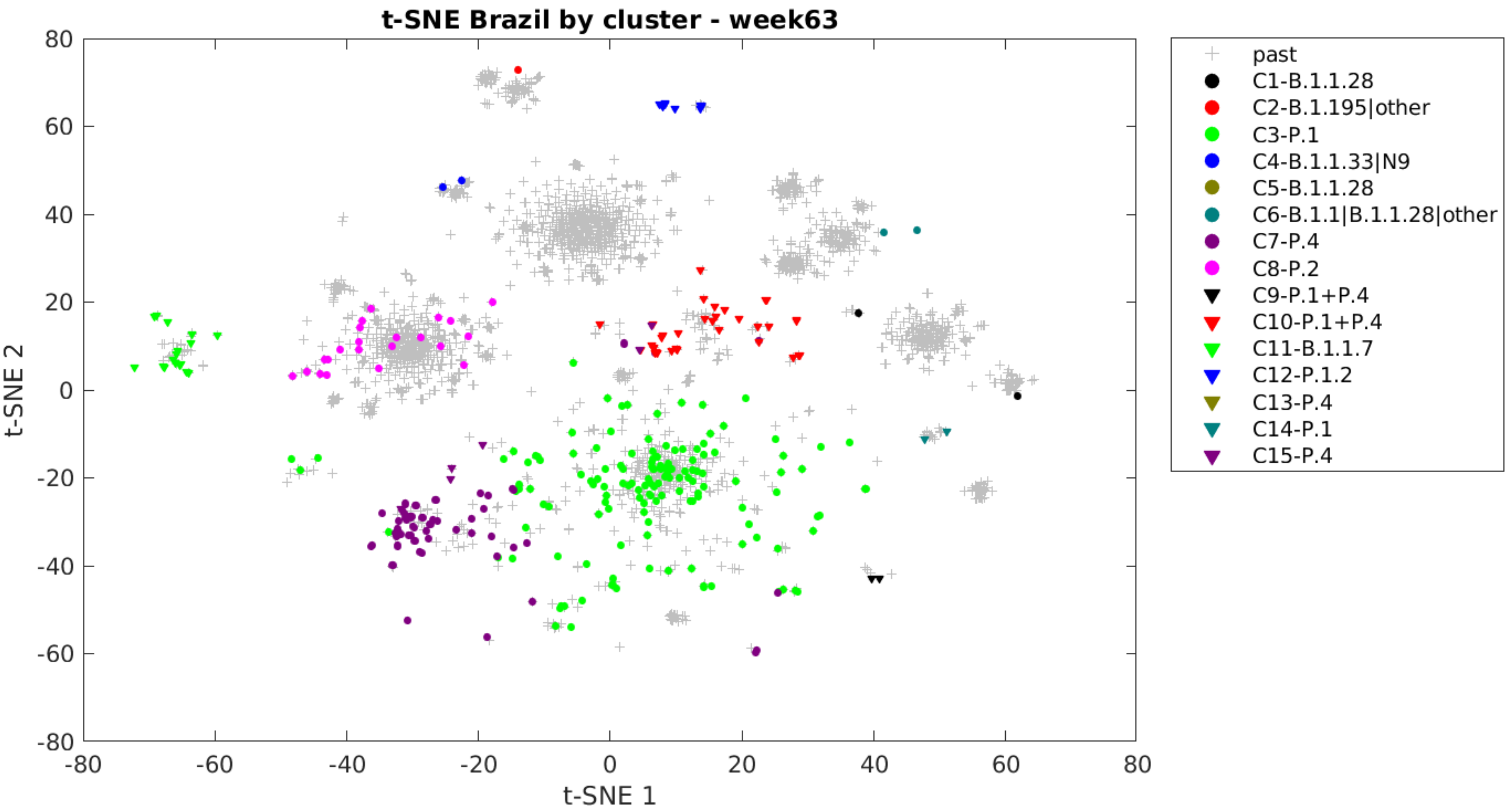

t-SNE Brazil by cluster - week63
t-SNE 1
t-SNE 2
-80
-60
-40
-20
0
20
40
60
80
past
C1-B.1.1.28
C2-B.1.195|other
C3-P.1
C4-B.1.1.33|N9
C5-B.1.1.28
C6-B.1.1|B.1.1.28|other
C7-P.4
C8-P.2
C9-P.1+P.4
C10-P.1+P.4
C11-B.1.1.7
C12-P.1.2
C13-P.4
C14-P.1
C15-P.4

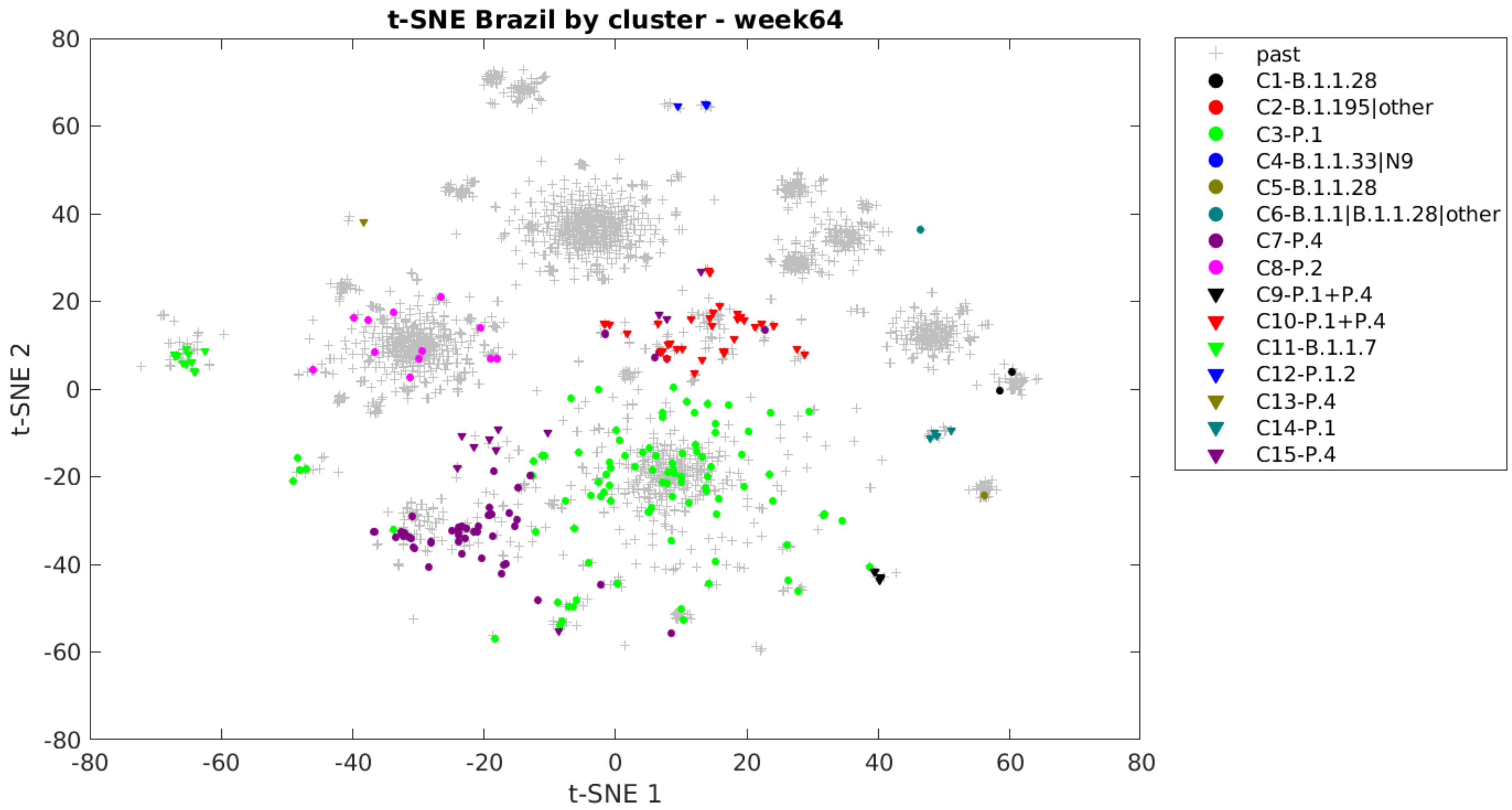

t-SNE Brazil by cluster - week64
t-SNE 1
t-SNE 2
80
60
40
20
0
-20
-40
-60
-80
past
C1-B.1.1.28
C2-B.1.195|other
C3-P.1
C4-B.1.1.33|N9
C5-B.1.1.28
C6-B.1.1|B.1.1.28|other
C7-P.4
C8-P.2
C9-P.1+P.4
C10-P.1+P.4
C11-B.1.1.7
C12-P.1.2
C13-P.4
C14-P.1
C15-P.4

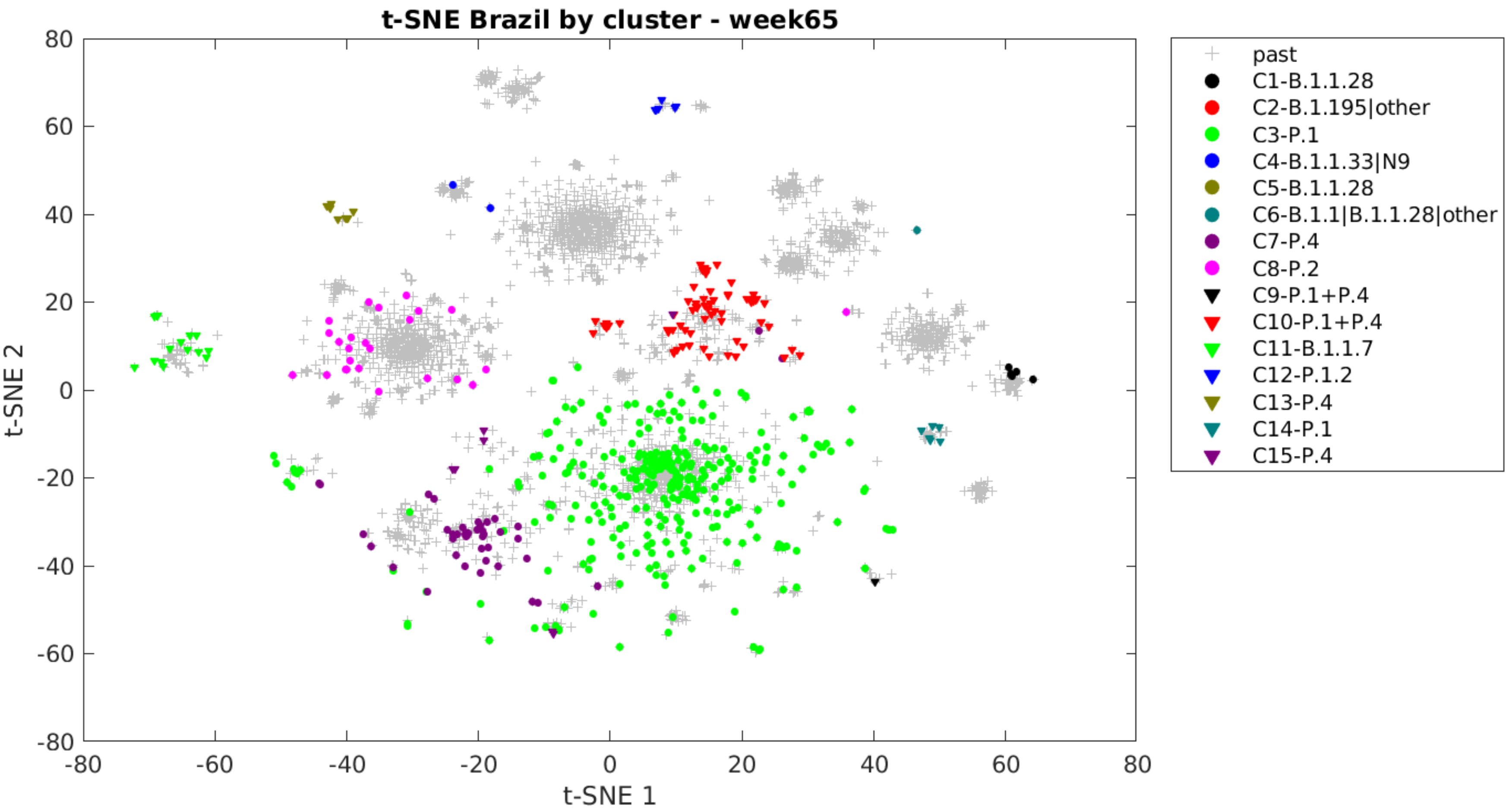

t-SNE Brazil by cluster - week65
80
60
40
20
0
-20
-40
-60
-80
-80
-60
-40
-20
0
20
40
60
80
t-SNE 1
t-SNE 2
past
C1-B.1.1.28
C2-B.1.195|other
C3-P.1
C4-B.1.1.33|N9
C5-B.1.1.28
C6-B.1.1|B.1.1.28|other
C7-P.4
C8-P.2
C9-P.1+P.4
C10-P.1+P.4
C11-B.1.1.7
C12-P.1.2
C13-P.4
C14-P.1
C15-P.4

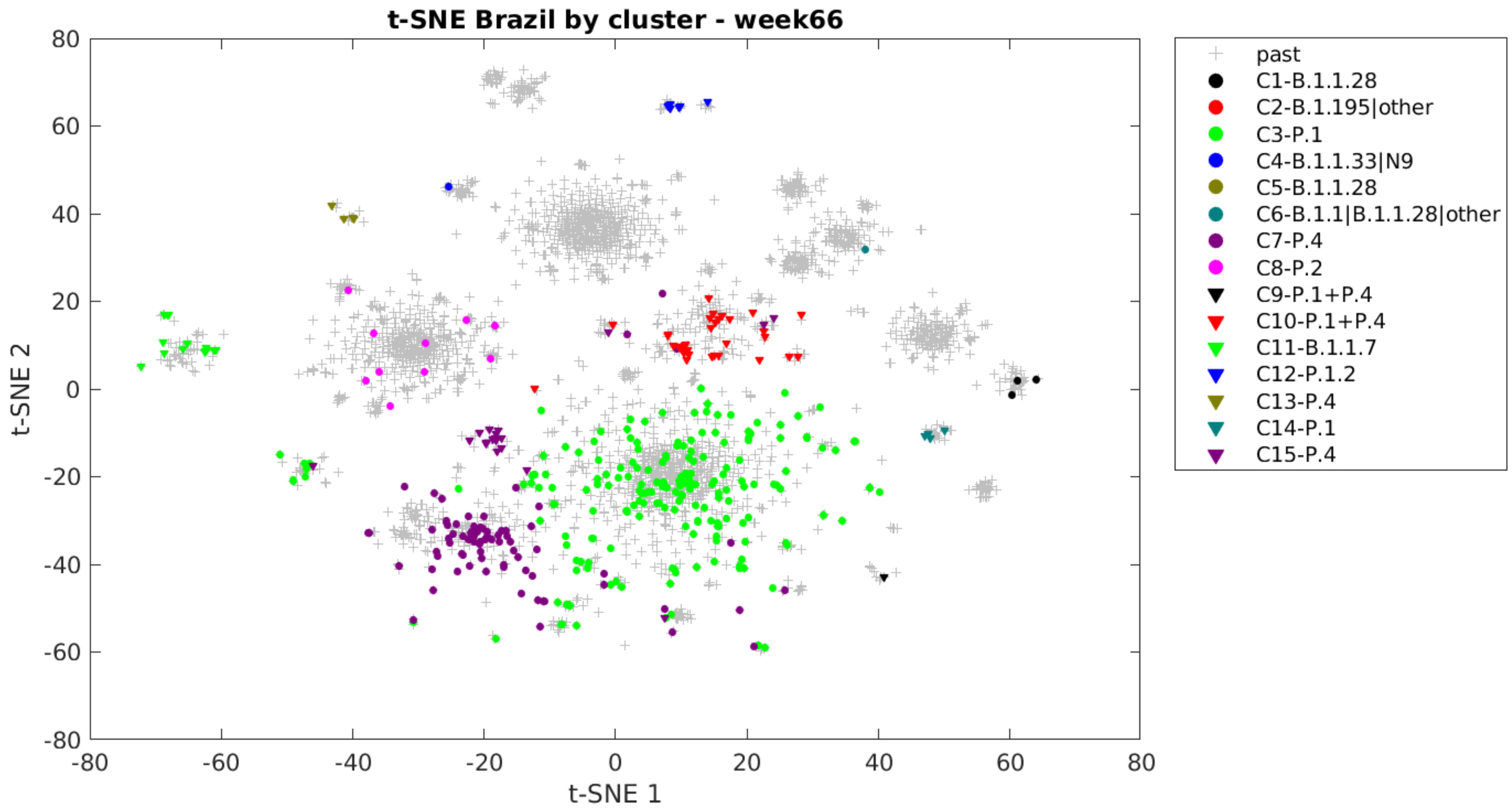

t-SNE Brazil by cluster - week66
t-SNE 2
t-SNE 1
80
60
40
20
0
-20
-40
-60
-80
-80
-60
-40
-20
0
20
40
60
80
past
C1-B.1.1.28
C2-B.1.195|other
C3-P.1
C4-B.1.1.33|N9
C5-B.1.1.28
C6-B.1.1|B.1.1.28|other
C7-P.4
C8-P.2
C9-P.1+P.4
C10-P.1+P.4
C11-B.1.1.7
C12-P.1.2
C13-P.4
C14-P.1
C15-P.4

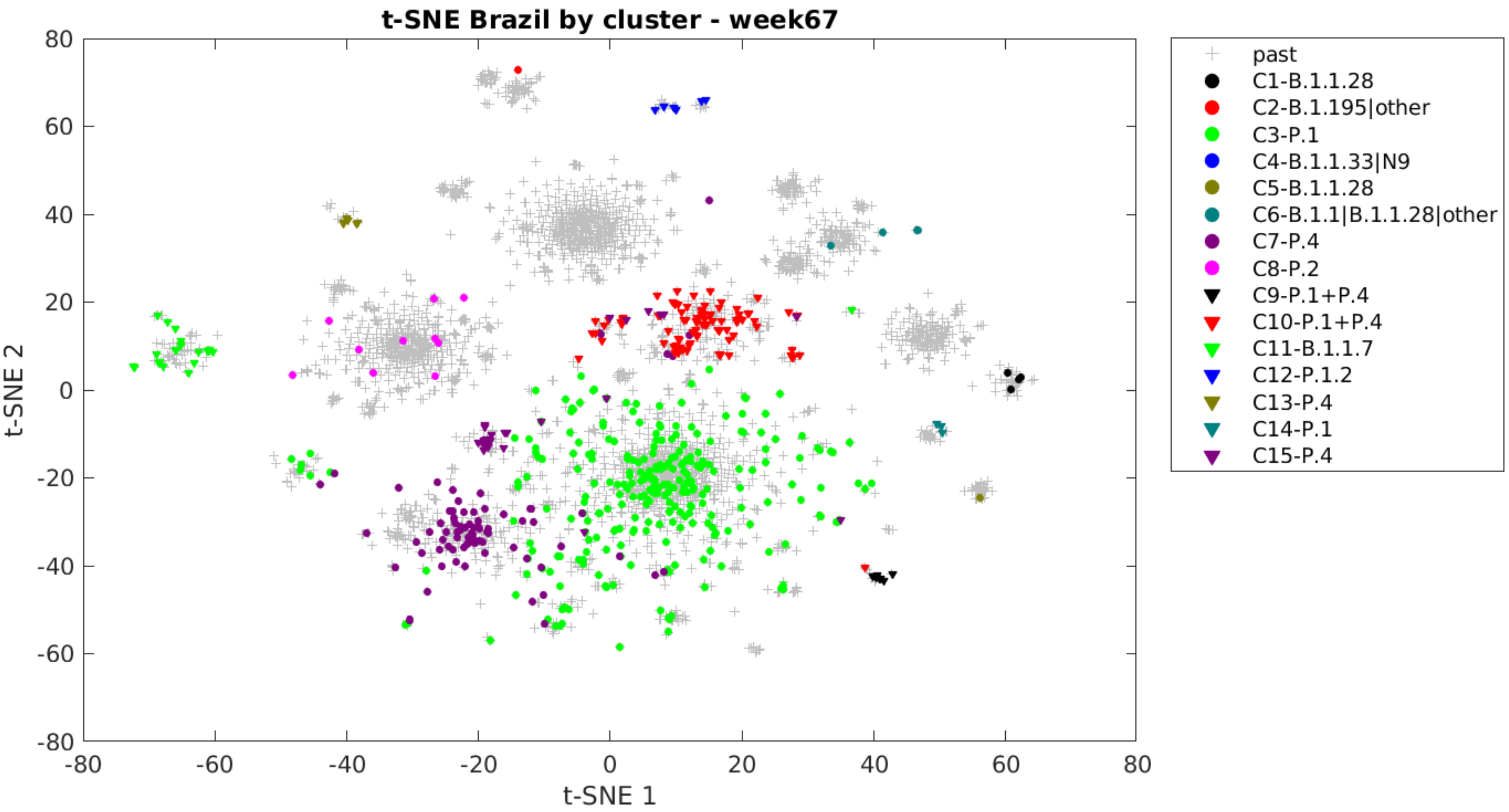

t-SNE Brazil by cluster - week67
t-SNE 1
t-SNE 2
past
C1-B.1.1.28
C2-B.1.195|other
C3-P.1
C4-B.1.1.33|N9
C5-B.1.1.28
C6-B.1.1|B.1.1.28|other
C7-P.4
C8-P.2
C9-P.1+P.4
C10-P.1+P.4
C11-B.1.1.7
C12-P.1.2
C13-P.4
C14-P.1
C15-P.4

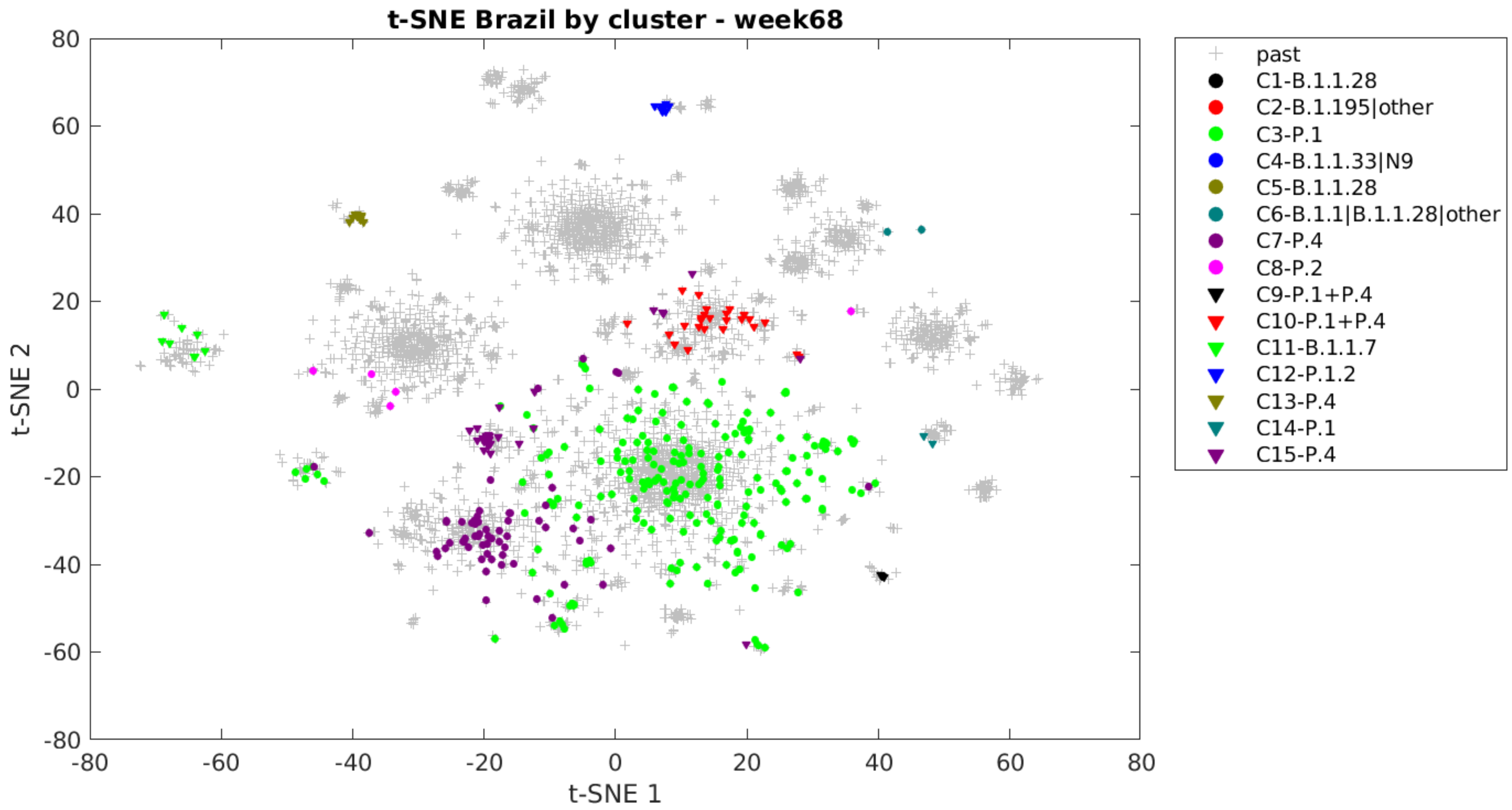

t-SNE Brazil by cluster - week68
t-SNE 2
t-SNE 1
80
60
40
20
0
-20
-40
-60
-80
-80
-60
-40
-20
0
20
40
60
80
past
C1-B.1.1.28
C2-B.1.195|other
C3-P.1
C4-B.1.1.33|N9
C5-B.1.1.28
C6-B.1.1|B.1.1.28|other
C7-P.4
C8-P.2
C9-P.1+P.4
C10-P.1+P.4
C11-B.1.1.7
C12-P.1.2
C13-P.4
C14-P.1
C15-P.4

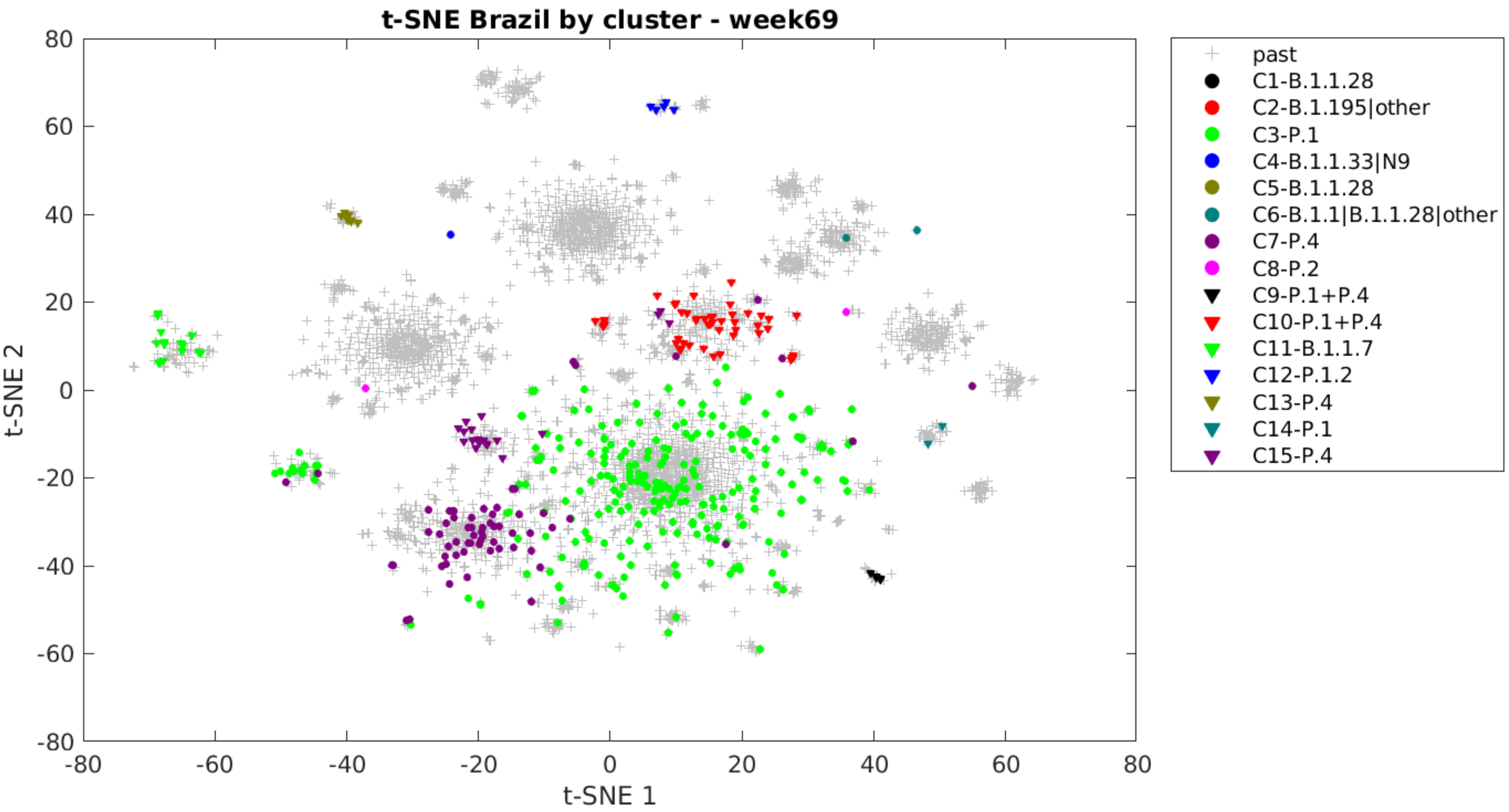

t-SNE Brazil by cluster - week69
80
60
40
20
0
-20
-40
-60
-80
-80
-60
-40
-20
0
20
40
60
80
t-SNE 1
t-SNE 2
past
C1-B.1.1.28
C2-B.1.195|other
C3-P.1
C4-B.1.1.33|N9
C5-B.1.1.28
C6-B.1.1|B.1.1.28|other
C7-P.4
C8-P.2
C9-P.1+P.4
C10-P.1+P.4
C11-B.1.1.7
C12-P.1.2
C13-P.4
C14-P.1
C15-P.4

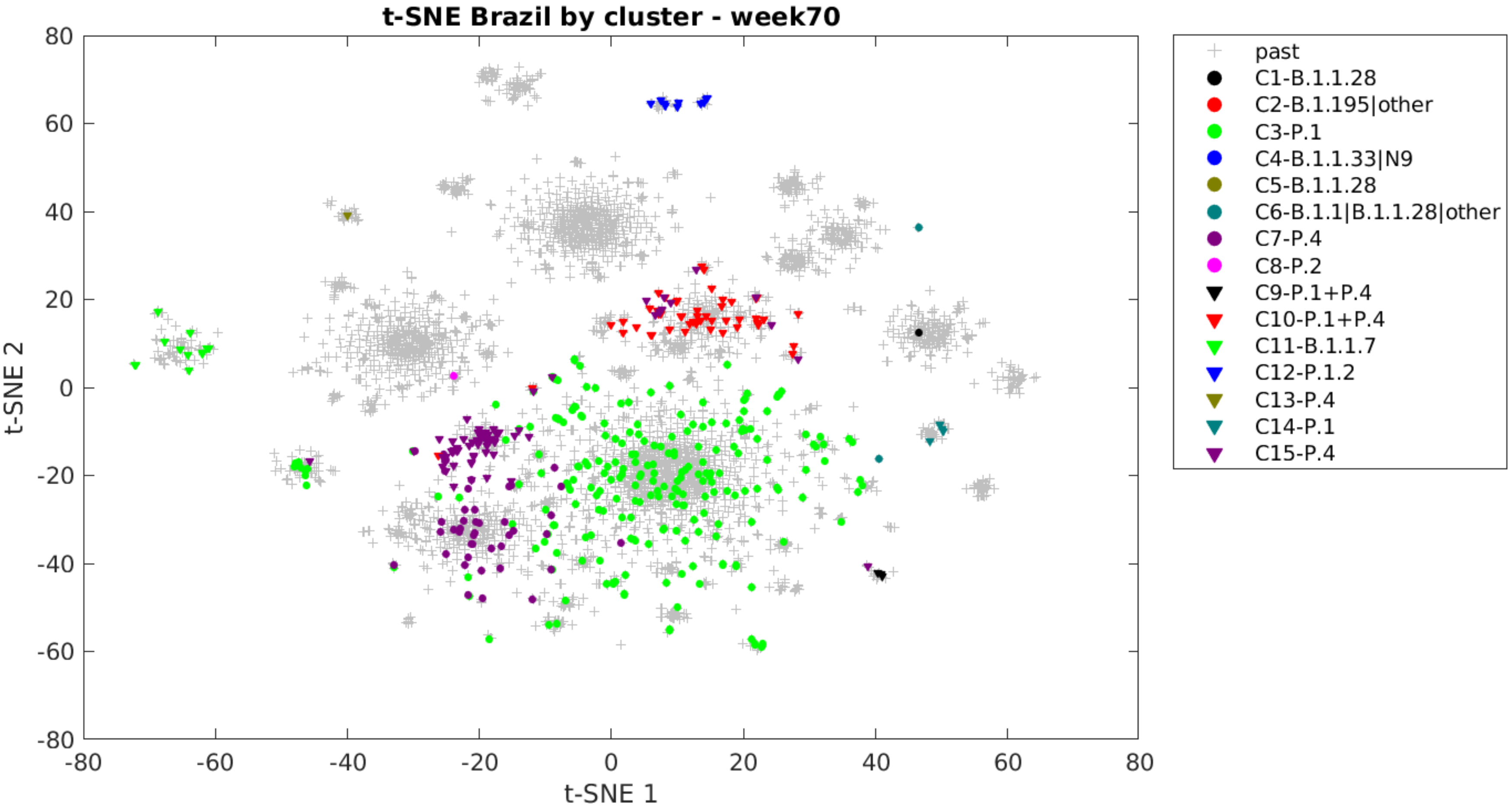

t-SNE Brazil by cluster - week70
80
60
40
20
0
-20
-40
-60
-80
-80
-60
-40
-20
0
20
40
60
80
t-SNE 1
t-SNE 2
past
C1-B.1.1.28
C2-B.1.195|other
C3-P.1
C4-B.1.1.33|N9
C5-B.1.1.28
C6-B.1.1|B.1.1.28|other
C7-P.4
C8-P.2
C9-P.1+P.4
C10-P.1+P.4
C11-B.1.1.7
C12-P.1.2
C13-P.4
C14-P.1
C15-P.4

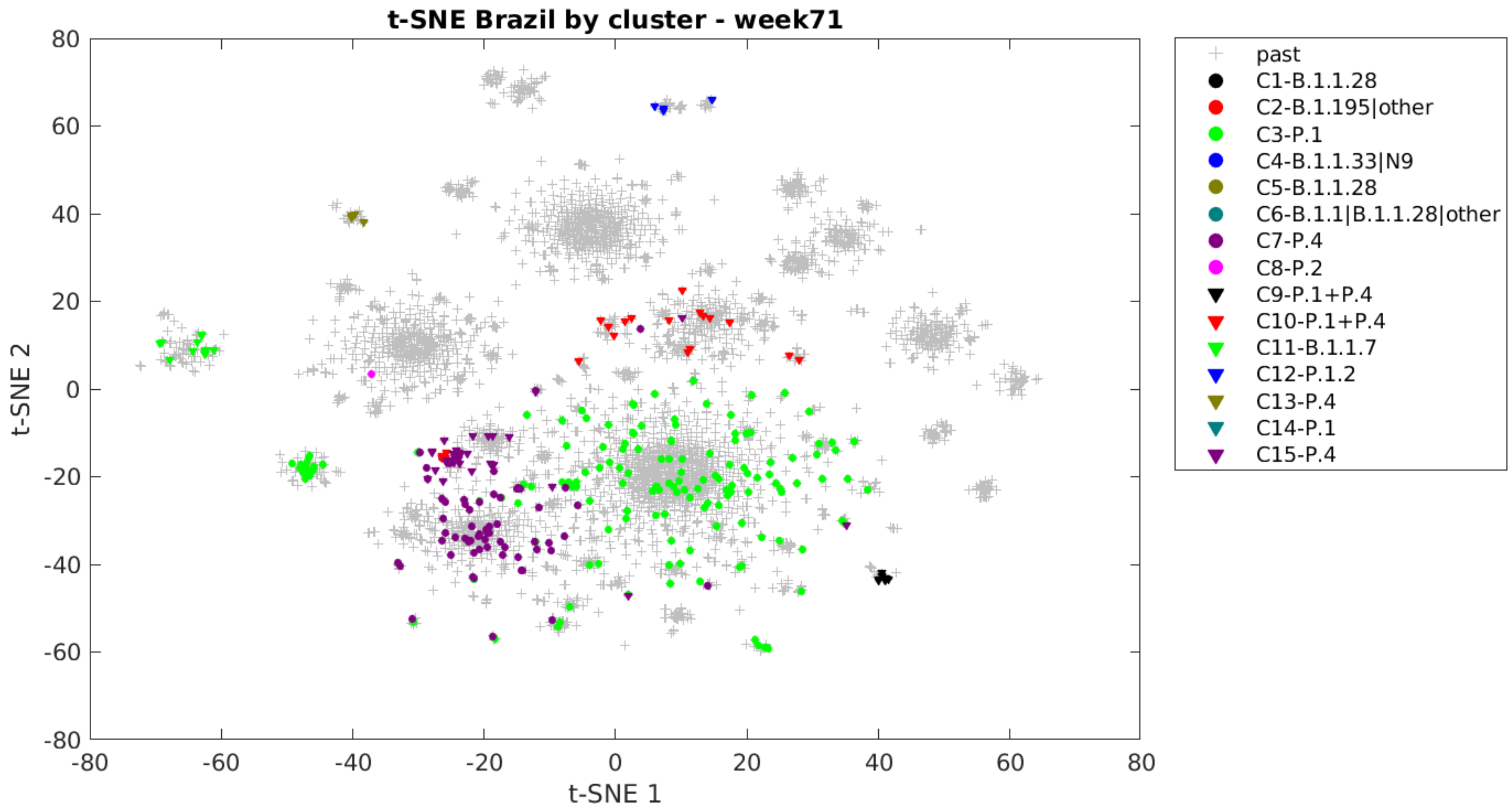
t-SNE Brazil by cluster - week71
80
60
40
20
0
-20
-40
-60
-80
-80
-60
-40
-20
0
20
40
60
80
t-SNE 2
t-SNE 1
past
C1-B.1.1.28
C2-B.1.195|other
C3-P.1
C4-B.1.1.33|N9
C5-B.1.1.28
C6-B.1.1|B.1.1.28|other
C7-P.4
C8-P.2
C9-P.1+P.4
C10-P.1+P.4
C11-B.1.1.7
C12-P.1.2
C13-P.4
C14-P.1
C15-P.4
